# Clonal relationship and alcohol consumption-associated mutational signature in synchronous hypopharyngeal tumours and oesophageal squamous cell carcinoma

**DOI:** 10.1038/s41416-022-01995-0

**Published:** 2022-10-19

**Authors:** Josephine Mun-Yee Ko, Chen Guo, Conghui Liu, Lvwen Ning, Wei Dai, Lihua Tao, Anthony Wing-Ip Lo, Carissa Wing-Yan Wong, Ian Yu-Hong Wong, Fion Siu-Yin Chan, Claudia Lai-Yin Wong, Kwan Kit Chan, Tsz Ting Law, Nikki Pui-Yue Lee, Zhichao Liu, Haoyao Jiang, Zhigang Li, Simon Law, Maria Li Lung

**Affiliations:** 1 Department of Clinical Oncology, University of Hong Kong, Hong Kong (Special Administrative Region), People’s Republic of China; 2 Division of Anatomical Pathology, Queen Mary Hospital, Hong Kong (Special Administrative Region), People’s Republic of China; 3 Department of Surgery, University of Hong Kong, Hong Kong (Special Administrative Region), People’s Republic of China; 4 Department of Thoracic Surgery, Section of Esophageal Surgery, Shanghai Chest Hospital, Shanghai Jiao Tong University, Shanghai, People’s Republic of China

**Keywords:** Oesophageal cancer, Cancer genomics

## Abstract

**Background:**

The patients with dual oesophageal squamous cell carcinoma (ESCC) and hypopharyngeal cancer (HPC) have poor prognosis; their underlying genetic pathogenesis is unclear. We hypothesise that development of synchronous ESCC/HPC depends on multicentricity or independent origin, rather than multifocality due to local or lateral spreading.

**Method:**

Multiple region whole-exome sequencing (M-WES) and clonality analysis were used to assess clonal relationship and spatial inter- or intra-tumour heterogeneity (ITH) in 62 tumour regions from eight dual ESCC/HPC and ten ESCC patients.

**Results:**

All synchronous ESCC/HPC patients had COSMIC 16 mutation signatures, compared to only 40% ESCC in the current study (*p* = 0.013) and public data set (*n* = 165, *p* = 0.003). This alcohol consumption-related mutation signature 16, commonly involved in multiple alcohol-related cancers, was significantly associated with drinking and alcohol metabolism-related *ADH1B* rs1229984. The mutational landscape and copy number profiles were completely distinct between the two primary tumours; clonality analysis further suggested the two primary tumours shared no or only one clone accompanying independent subclone evolution. M-WES strategy demonstrated higher sensitivity and accuracy for detection of mutational prevalence and the late branch mutations among different regions in the ESCC tumours, compared to traditional sequencing analysis based on single biopsy strategy. Patients with high ITH assessed by cancer cell fraction analysis after M-WES were significantly associated with both relapse and survival.

**Conclusions:**

Our hypothesis-generating M-WES ITH assessment data have implications for prognostication. Collectively, our findings support multicentric independent clonal evolution, the field cancerisation theory, and suggest novel insights implicating an aetiologic role of alcohol metabolism in dual ESCC/HPC carcinogenesis.

## Introduction

Oesophageal cancer (EC) is a deadly cancer worldwide [1]. Oesophageal squamous cell carcinoma (ESCC) is the prevalent histological subtype endemic in Asia, Africa, and Europe [1]. The occurrence of multiple primary cancers occurs in 8.3–12.6% of ESCC patients [2–7]. The treatment and prognosis of patients with dual ESCC/head and neck cancer (HNC) are primarily based on ESCC stage, which is usually advanced at diagnosis, contributing to its dismal five-year survival and management challenges [3]. Current treatment strategies, including chemoradiation therapy (CRT) and surgery, are often ineffective due to intra-tumoural heterogeneity (ITH) derived from the complex subclonal tumour evolution. The multiple primary HNCs and aerodigestive tract cancers may be driven by “field cancerization” [8]. This concept proposes an aetiologic role of epithelium exposure to common carcinogens associated with alcohol and tobacco consumption, leading to multifocal lesions. To date no comprehensive genomic studies have examined the genetic pathogenesis of multifocal tumours with regards to the ITH and mutational signatures in patients with dual synchronous ESCC/hypopharyngeal cancer (ESCC/HPC) [9, 10]. It is unclear whether synchronous tumours share similar genomic features arising from clonal expansion or if they independently evolve. To understand its underlying aetiology, we applied multiple region-sampling whole-exome sequencing (M-WES) strategy and compared the mutation signatures associated with primary ESCC and synchronous ESCC/HPC. WES analysis demonstrated the quantification of ITH and existence of more than two subclones could be a universal potentially useful survival biomarker across twelve cancer types, but this is unknown for ESCC [11]. We aimed to utilise M-WES analysis to evaluate if degree of ITH carries prognostic information.

## Methods

During 2018–2019, ten ESCC and five synchronous ESCC/HPC patients were recruited from Queen Mary Hospital (QMH), University of Hong Kong. Another three synchronous ESCC/HPC patients were recruited from 2020 at Shanghai Chest Hospital (SCH). Diagnosis of synchronous ESCC/HPC was based on the criteria of clear separation of tumours by histological examination and normal mucosa and the exclusion of the second tumour due to metastasis of the primary tumour [12]. A total of 60 tumour tissues, 6 matched normal tissues from oesophagus and hypopharynx and 15 matched fresh bloods from 10 ESCC and 8 ESCC/HPC patients with patient’s informed consent were obtained for M-WES analysis. The study was approved by the Ethics Committee of Shanghai Jiaotong University and Institutional Review Board of the University of Hong Kong/Hospital Authority Hong Kong West Cluster (HKU/HA HKW IRB number UW 17–187) and was performed according to the principles of the Declaration of Helsinki. All patients were treatment-naive, directly undergoing surgery. M-WES was utilised to understand spatial ITH by obtaining 4–6 ESCC regions from distant regions of each tumour specimen after upfront surgery of 10 Hong Kong ESCC patients. For dual cancer patients, at least one specimen from each primary site was collected, except there was no available HP tumour for HK5 (Table S1). Figure S1 shows good-quality sequencing data with mean target coverage for 81 samples from 18 patients.

### Whole-exome sequencing and bioinformatics analysis

WES libraries were prepared with KAPA HTP Library Preparation and SeqCap EZ Exome+UTR Kits (Roche). WES and bioinformatics analysis were performed, as previously described, and detailed in the supplementary methods [13, 14]. The multi-regional sample identities were confirmed using Identity-by-descent (IBD) analysis by PLINK v1.9. A mutational heatmap with the ComplexHeatmap R package showing the protein-altering mutations or mutated genes across regions was drawn for each ESCC patient [15]. Subclonal compositions of multi-regional samples were analysed using the SuperFreq R package considering somatic single nucleotide variants (SNVs) and CNVs [16]. Bamfiles containing the pre-filtered variants including both germline variants and somatic mutations were input into SuperFreq to generate patient river plots. Copy number variation (CNV) gains and losses were above 1 or below −1 after log 2 transformation. Cancer cell fractions (CCFs) were computed for each somatic mutation based on sample purity (predicted by ABSOLUTE v1.2) and local copy-number [17]. Sanger sequencing demonstrated an accuracy of 98.2% (110/112) for selected mutations (Table S2).

### Mutation signature analysis

Lists of exonic mutations were uploaded to the web-based tool, Mutational Signatures V3.2 (http://cancer.sanger.ac.uk/signatures/) for mutation signature calling. Reports of somatic mutation prevalence, mutational profiles, COSMIC signature contributions, comparison with cancer signatures, reconstructed mutational profiles, and principal component analysis were downloaded. Mutational signature calling was performed for 165 ESCC patients from previous publications with two data sets from Sequence Read Archive (accession number SRP033394) and European Genome–phenome Archive (accession number EGAS00001000932). Another in-house WES data set included our previous study [13].

### Statistical analysis

The differences between mutation signature occurrence and association of survival and relapse with ITH were examined with Fisher’s exact test. Pearson correlation coefficient was calculated for the correlation between mutation signature 16 and drinking, males, smoking, and dual primary cases. A *p* < 0.05 (2-sided) was considered statistically significant. Analyses were performed with SPSS v26 (SPSS Inc., IBM Corporation, Armonk, NY, USA).

## Results and discussion

### Synchronous ESCC/HPC and ESCC patients’ clinical parameters

Detailed clinical information of 18 synchronous ESCC/HPC and ESCC patients for unbiased M-WES analysis is summarised in Table 1. All ESCC/HPC and 70% ESCC patients are males; age ranges of ESCC/HPC (49–84, average = 62.9) and ESCC (48–85, average = 62.6) patients are similar. Five (62.5%) ESCC/HPC and one (10%) ESCC patient died, after a median 23.5 months follow-up.

**Table 1:** Clinical information of 15 Hong Kong and 3 Shanghai ESCC patients receiving surgery.

Patient Index	Tumour code^a^ (multiple regions)	Sex	Age	Stage ESCC	Stage hypopharynx cancer	Clonal relationship of dual primary cancers	Drinking status	Smoking status	Smoking cessation (years)	Survival status	Survival time (months)
SH1	E H	M	64	T2N0M0	T3N0M0	Not clonal	Drinker 35 yr	20 cig/d 35 yr	NA	Alive	18
SH2	E H	M	62	T3N1M0	T2N0M0	One subclone in common	Ex-drinker 35 yr	Ex-smoker 20 cig/d, 35 yr	5	Dead	12
SH3	E H	M	56	T2N3M0	T3N1M0	Not clonal	Drinker 30 yr	20 cig/d 30 yr	NA	Alive	18
HK1	E (A, B, C) H (A, C)	M	49	T2N0	T2N2	Not clonal	Non-drinker	Chronic smoker	NA	Alive	60
HK2	E (A) H (A, B, C)	M	67	T1a	T3N3b	One subclone in common	Ex-drinker	Ex-smoker 10 cig/d 52 yr	Unknown	Dead	7
HK3	E (B) H (A)	M	84	T3N3	T4	One subclone in common	Ex-drinker	Ex-smoker	30	Dead	2
HK4	E H	M	58	TisN0	T1N2b	Not clonal	Ex-drinker 30 yr	Ex-smoker >60 pack yr	Unknown	Dead	13
HK5^b^	E (A, B, C, D)	M	63	T2N2b	T4a	–	Ex-drinker	Ex-smoker 40 yr	Unknown	Dead	15
HK6	E (B, D, E)	M	70	T3N1	NA	–	Drinker	Non-smoker	NA	Alive	41
HK7	E (B, C, D)	F	61	T3N2	NA	–	Non-drinker	Non-smoker	NA	Alive	37
HK8	E (A, B, C, D, E)	M	53	T1bN0	NA	–	Ex-drinker	Ex-smoker	20	Alive	26
HK9	E (A, B, C, D)	F	71	T1bN0	NA	–	Non-drinker	Non-smoker	NA	Alive	37
HK10	E (A, B, C)	M	48	T1bN2	NA	–	Drinker	Ex-smoker 1/2 pack/d	Unknown	Alive	37
HK11	E (A, B, C, D)	M	61	T3N2Mx	NA	–	Non-drinker	Non-smoker	NA	Alive	25
HK12	E (A, B, C, D)	M	57	T2N0	NA	–	Drinker	Ex-smoker	Unknown	Alive	23
HK13	E (A, B, C, D)	M	51	T1bN0	NA	–	Ex-drinker	Non-smoker	NA	Dead	17
HK14	E (A, B, C, D)	F	85	T2N1	NA	–	Non-drinker	Non-smoker	NA	Alive	24
HK15	E (A, B, C, D)	M	69	T3N0	NA	–	Ex-drinker	Non-smoker	NA	Alive	25

^a^E and H stand for ESCC and hypopharynx tumours, respectively. Only regions with carcinoma content ≥30% were used for WES analysis.

^b^Cases HK1–5 and SH1–3 were patients with dual tumours, while HK6-15 were patients with primary ESCC tumour excised for multi-regional sampling. HK5 has matched synchronous tumour in hypopharynx, but the tissue was not available for WES.

### Multi-region sampling captures intra-tumour heterogeneity of SNVs and CNVs of synchronous ESCC/HPC patients

**Fig. 1:**
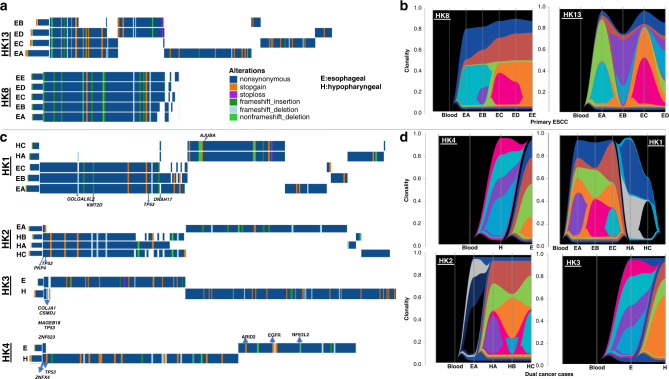
Inter- and intra-tumour heterogenity in dual cancer and ESCC patients. Heatmap showing the exonic non-silent mutations and clonality analysis by combining SNVs and CNVs in two primary ESCC (HK8 and HK13) (**a**, **b**) and four ESCC-related dual cancer patients (HK1-4) (**c**, **d**). HK8 and HK13 showed low and high degree of ITH for comparison of spatial and temporal ITH within single ESCC tumour and the ESCC/HP tumours from dual cancer patients. River plots show several subclones in different colours contributing to one regional sample.

**Fig. 2:**
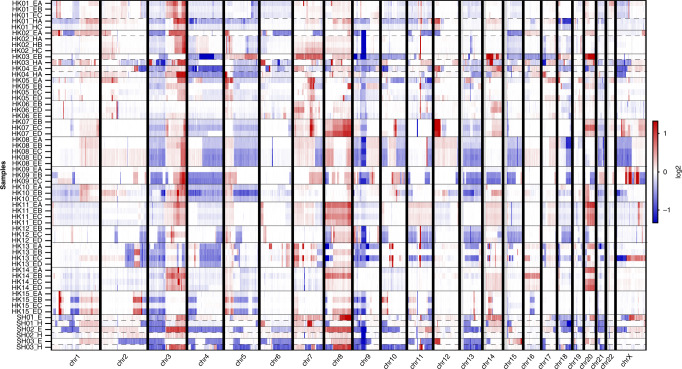
Inter- and intra-tumour heterogeneity reflected by M-WES CNV analysis. CNV plots showing distinctive inter-tumour heterogeneity pattern between hypopharygngeal and oesophageal regions of synchronous tumour cases (HK1-4) and ITH between multiple regions of ESCC (HK5-HK15).

The mean coverage of aligned reads was 52.6×, 72.4× and 88.8× for 15 bloods or 6 matched normal and 60 tumour samples from 18 patients, respectively (Fig. S1). The total numbers of exonic non-silent mutations (including frameshift and non-frameshift indels, stop gain, stop loss, and nonsynonymous) of 18 patients are summarised in Table S3. Similar somatic mutation prevalence was observed among patients with only primary ESCC and with dual cancers, with an average of 4.35 somatic exonic mutations/Mb (range 0.52–11.58) (Fig. S2). *TP53* remained the most frequently mutated gene in dual cancer (71.4%, 5/7) and ESCC (70%, 7/10) patients. Surprisingly, both the mutational landscape (Figs. 1c and S3A) and CNV patterns across chromosomes (Fig. 2) of oesophageal and hypopharyngeal tumours of synchronous ESCC/HPC patients are distinct. Two primary tumours from four dual cancer patients (4/7, 57.1%) shared no common exonic non-silent mutations, while a few mutations were shared in three patients (Table S4). The ESCC/HPC tumours showed high inter-tumour heterogeneity in terms of CNVs at ten genes frequently occurring in ESCC (Fig. S4). For the common CCND1 amplification at 11q13, high intra-tumour heterogeneity existed in three dual primary patients, HK3, HK4 and SH2, which was only amplified in either the ESCC or HP tumours, but not present in both. CCND1 amplification was present in both the ESCC and HP tumours in HK1, HK2, SH1 and SH3 although the three ESCC (EA, EB, and EC) and two HP tumours (HA and HC) of HK1, as well as the ESCC and HP tumours of SH1 were amplified to different levels. Further analysis of CNVs between the ESCC and HP tumours of the same patients identified 73 regions differentially amplified or deleted CNVs between the ESCC and HP tumours of the same patient (*p* < 0.01, fdr <0.15) (Table S5). These included cancer relevant genes involved in actin cytoskeleton remodelling, integrin activation, EMT-*Wnt* signalling pathway, and metastasis or with potential prognostic role such as *SPEN* on chromosome 1, *PLEKHG4B*, and *CCT5* on chromosome 5, *LRP5* on chromosome 11, *RFPL1*, *SLC5A1*, and *SHANK3* on chromosome 22 [18–22]. Our WES findings substantiate earlier reports of discordant patterns of inter-tumour allelic losses inferred by microsatellite typing or SNP profiling in multiple ESCC and HNCs patients [9, 10]. The clonality analysis tracking multiple clones from different tumour regions in the same individual further confirmed that no (HK1, HK4, SH1, SH3) or only one clone (HK2, HK3, SH2) was shared between the primary sites, consistent with the GATK-called mutations (Figs. 1d and S3B). Clonality analysis easily differentiates the ESCC/HPC dual cancers from ESCC patients with clonal relationships (Fig. 1). More detailed analyses with respect to the change of dominant subclones (defined as clonality>0.5) and somatic mutations revealed that, in general, the dominant subclones were mutually exclusive between ESCC and HP tumours from dual-cancer patients (Table S6). Among the multiple regions of ESCC, one dominant subclone was shared in at least two ESCC regions in HK5, two dominant subclones were shared in at least two regions in HK8. At least three or four dominant subclones were shared in at least two regions in the remaining 9 ESCC patients without dual cancer. Three (3/7, 42.9%) dual cancer patients had single shared clones containing both CNVs and mutations mostly occurring at intronic, promoter and untranslated regions (Table S7), suggesting the possibility of a minority of tumour cells migrating along the aerodigestive tract. To the best of our knowledge, this is the first WES study reporting the completely distinct genetic landscape, CNV profile and subclones between two patient’s tumours of synchronous dual cancers. The mechanism underlying unique origins of dual cancers is supported by WES mutation landscape and CNV profiling. Their entirely different mutational landscapes and CNV patterns suggest the multicentricity of HPC and ESCC tumours was not clonally related and arose independently. Future studies with larger sample cohorts are warranted, as we cannot rule out the possibility that the differences of copy number variation pattern and genetic mutation profile may result from the small size of the cohort in the study.

### Alcohol consumption-related mutational signature 16 is dominant in synchronous cancer patients

**Fig. 3:**
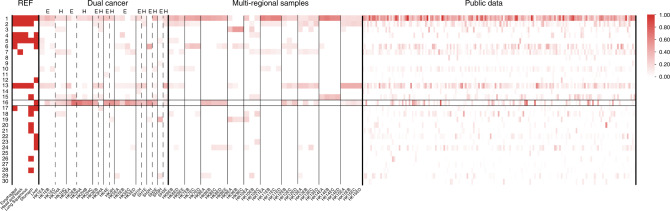
COSMIC mutation signature distribution of dual primary, ESCC and public data. The 165 WES samples were analysed by Musica to call COSMIC mutation signature contributions. The most common mutation signatures were signatures 1 (165/165), 13 (144/165), 2 (116/165), 7 (116/165), 10 (95/165), and 6 (91/165). The underlying aetiology of ESCC in both our cohorts (*n* = 10) and the public data set (*n* = 165) suggested by the seven dominating mutational signatures: ageing (signature 1), APOPEC enzyme family activity (signatures 2 and 13), mismatch repair (signature 6), POLE (ultra-hypermutation) (signature 10), UV exposure in squamous cancers (signature 7), and signature 16. Mutation signature 16 occurs predominately in all 8 dual ESCC/HPC, but only in 40% of 10 ESCC patients treated by upfront surgery in our cohorts and the WES public data set with 165 ESCC patients.

The mutagenic processes from defective DNA repair, replication and genotoxins exposure of various carcinogens in cancer genomes during tumourigenesis are reflected by the mutational signatures [23, 24]. The M-WES studies in 36 regions from ten primary ESCC identified seven frequently occurring signatures (1, 2, 6, 7, 10, 13, 16) from 30 COSMIC mutational signatures, similar to those observed from 165 single-region ESCC WES public data (Fig. 3) and are concordant to earlier studies [25–27]. All patients showed ITH by M-WES in the mutational signatures (1, 2, 6, 7, 13, and 16) detected in at least one-third of all regions (12/36). Significantly, mutation signature 16, which is prevalent in ESCC synchronous cases (100%, 8/8), has a significantly lower frequency in ESCC patients in the current study (40%, 4/10, *p* = 0.013) and in the publicly available data set (40%, 66/165, *p* = 0.003) (Fig. 3). Our current study is the first to report such a high prevalence of COSMIC mutation signature 16 in dual ESCC/HPC patients compared to the current study and literature reported frequency ranging from 16–40% in ESCC [26]. Mutation signature 16 correlates with drinking, males, smoking, dual primary cases (Pearson correlation 0.723, *p* = 0.001, 0.721, *p* = 0.001, 0.523, *p* = 0.026, and 0.555, *p* = 0.017, respectively). By Fisher’s exact test, mutation signature 16 associated significantly with drinking (*p* = 0.008), smoking (*p* = 0.047), male gender (*p* = 0.012) and a trend with relapse (*p* = 0.101) and overall survival (*p* = 0.114) (Table 2). The distinct occurrence of drinking-associated mutation signature 16 between ESCC-related dual cancers bridges the gap between field cancerization and alcohol exposure. Past epidemiological studies reported that alcohol consumption is a risk factor of multiple dysplasia lesions in the oesophagus, head and neck, and development of multiple primaries in the upper-GI tract [28]. The alcohol consumption-associated mutational signature 16 was previously identified as one of the common mutational signatures in several alcohol-related cancers including liver cancer, head and neck cancer, and ESCC, highlighting the aetiologic risk factor role of alcohol consumption in tumourigenesis [26, 29, 30]. In the current study, the presence of mutation signature 16 occurs in all dual ESCC/HPC patients and correlates to drinking history. We further verified the relationship between COSMIC signature 16 and alcohol-related SNP *ADH1B* rs1229984 (T>C) in the public ESCC cohort and further confirmed that this signature was associated with the patients carrying the C allele of rs1229984, especially those patients with homozygous CC (CC vs. TT, 44.4% vs. 26.5%, *p* = 0.047, Chi-Square test). Consistently, dual synchronous ESCC/HPC patients were more likely to carry genotype CC compared to other ESCC patients with marginal significance (dual cancer vs. ESCC, 50.0% vs. 23.0%, *p* = 0.0998, Fisher’s exact test). East Asian individuals carrying the TT/TC genotypes of the alcohol metabolism-related functional SNP *ADH1B* rs1229984 were more likely to experience drinking discomfort, as the T allele causes rapid accumulation of acetaldehyde after alcohol intake. Our association findings of rs1229984 with COSMIC signature 16 were in line with earlier observation of individual carriers with the CC genotype of *ADH1B* rs1229984 having a significantly higher risk of multiple alcohol-related cancers of the larynx, pharynx, oesophagus, and pancreas [31]. Thus, our study provides new evidence implicating the aetiologic role of alcohol consumption in dual ESCC/HPC tumourigenesis. The molecular pathogenesis of HNC is classified into nonkeratinized HPV-related and keratinised HPV-unrelated forms associated with elderly males, smoking and drinking [32]. HPV-16/18 DNAs were undetectable by PCR-based consensus primer analysis (Fig. S6) [33], suggesting dual primary cancer patients in our cohort belong to the HPV-unrelated subgroup, which was further supported by their high frequency *p53* mutations (Table S4) and *p16* deletions (Fig. S4). Given the limited clinical sample capacity due to the rarity of dual cancers in ESCC patients, findings in the current study are considered hypothesis-generating and the novel insights between alcohol consumption in dual ESCC/HPC tumourigenesis merit further validation with larger sample cohorts. Despite the limited number of cases analysed, our findings are clinically relevant. The power of our study was 97.52% with the detection of COSMIC 16 mutational signature in 100% of the 8 HP/ESCC cases and 40% of the 165 ESCC from public data set and the type I error rate of 0.05. Given that mutational signature 16 is significantly more prevalent in ESCC-related dual cancers than ESCC patients, the likelihood of developing multiple primaries may be assessed in part with this potential molecular parameter.

**Table 2:** Dual primary cancers, COSMIC mutational signature 16 and ITH association with relapse and overall survival.

	Smoking			Drinking			Relapse			Overall survival		
	Yes	No	*p*^a^	Yes	No	*p*^a^	Yes	No	*p*^a^	Dead	Alive	*p*^a^
Dual primary												
Yes	8	0	**0.004**	7	1	0.314	5	3	0.145	5	3	**0.043**
No	3	7		6	4		2	8		1	9	
Total	11	7		13	5		7	11		6	12	
COSMIC 16 mutational signature												
Yes	10	3	**0.047**	12	1	**0.008**	7	6	0.101	6	7	0.114
No	1	4		1	4		0	5		0	5	
Total	11	7		13	5		7	11		6	12	
ITH^b^												
Yes	1	2	1.000	3	0	0.205	3	0	**0.005**	2	0	**0.045**
No	4	5		4	5		0	9		1	9	
Total	5	7		7	5		3	9		2	10	

Bolded *p* values = statistically significant, <0.05.

^a^Fisher Exact test, 2-tailed.

^b^ITH defined by CCF analysis.

### M-WES sensitively tracks ITH by multiple sampling

**Fig. 4:**
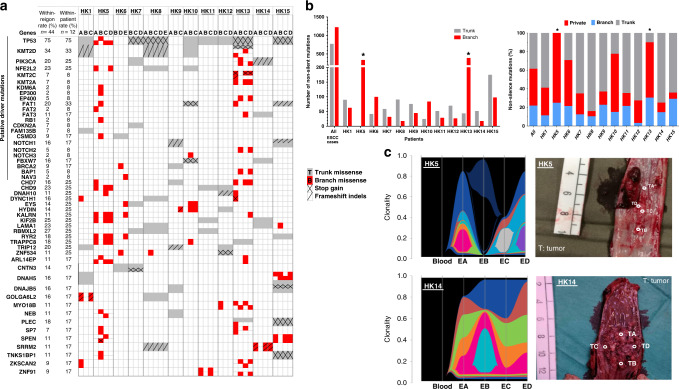
Intra-tumour heterogeneity reflected by M-WES SNV and clonality analysis. Clonal status of putative driver mutations defined by previous large-scale ESCC genomic studies [26, 48–51] and frequently mutated genes in different regions across patients by **a** heatmap. Tumour suppressor mutations predominately occurred early in the evolutionary process as trunk mutations (TP53, KMT2D, NOTCH1, FBXW7, CDKN2A, etc.), while most other driver gene mutations occurred as both trunk and branch mutations, including oncogenes (PIK3CA, NFE2L2) and other genes involved in WNT signalling (FAT1, FAT2, FAT3), DNA repair (BRCA2, BAP1), histone modification and chromatin remodelling (EP300, EP400, KMT2C, KMT2A, KDM6A). **b** The distribution of trunk, private or shared branch mutations in 12 patients. In a total of 1982 somatic non-silent mutations, 709 are clonal trunk and 1273 (64.2%) subclonal branch mutations. **c** Clonality analysis and the physical distance of multiple ESCC regions of HK5 (dual primary cancers) and HK14 (primary ESCC). Grey and red indicate trunk and branch mutations, respectively. ^∗^denotes the highest heterogeneity rate of 100 and 90% in two patients, HK5 and HK13.

M-WES analysis of 12 patients in multiple ESCC regions identified 1981 non-silent mutations in 1749 genes in 44 regions (Table S3). The median number of exonic mutations detected was 117 (non-silent = 88), similar to previous reports [24, 27]. Researchers attempted to investigate inter- and intra-tumour heterogeneity by multi-regional sampling underlying the phylogenetic branched evolution model, providing evidence that such spatial and temporal ITH occurs in various cancers including ESCC [27, 34–39]. Our findings of ESCC ITH recapitulate similar observations of high ITH in two earlier studies [25, 27]. The presence of mutations in all regions from an ESCC patient is defined as early “trunk”; otherwise, it is defined as late branch mutations during clonal evolution. The phylogenetic trees constructed based on the somatic mutations in all ten primary ESCC patients to track evolutionary patterns, indicated extensive spatial ITH variation of the somatic non-silent mutations (Fig. S5). The generalisation that driver tumour suppressors occurred early as trunk mutations did not apply to two patients with poor prognosis; two branch missense mutations at *TP53* in HK5 and *KMT2D* in HK13 were detected (Fig. 4a). The within-patient mutation rates of ESCC putative driver genes are higher than within-regions (Table S8). The strategy of M-WES analysis with multiple region sampling resulted in more sensitive detection of late branch mutations such as *KMT2A*, *EP300*, and *EP400*. ITH may impede precision medicine by affecting strategies on biopsy sampling and treatment decision through more in-depth characterisation of actionable drug targets [40, 41]. this study also identified an additional 28 frequently mutated genes, including 5 trunk and 23 branch mutations in 16.7–25% of 12 patients, suggesting the current single biopsy sequencing analysis will underestimate mutational prevalence and miss late branch mutations occurring in tumour evolution. Two of the five newly identified trunk early mutations previously not reported in ESCC, *RBMXL2* and *TRIP12*, occur in 25% (3/12) patients. *RBMXL* encodes an RNA-binding protein complex with heterogeneous nuclear RNA (hnRNA) involved in pre-mRNA processing and regulating splicing with unknown functions [42]. *TRIP12* (*thyroid hormone receptor interactor 12*) encodes an E3 ubiquitin-protein ligase regulating degradation of the critical ESCC tumour suppressors including FBXW7 and the *p19ARF*/*ARF*/*TP53* axis [43, 44]. Future M-WES studies are needed to evaluate whether any of these are potential ESCC driver mutations.

### High ITH associated with poor survival

**Fig. 5:**
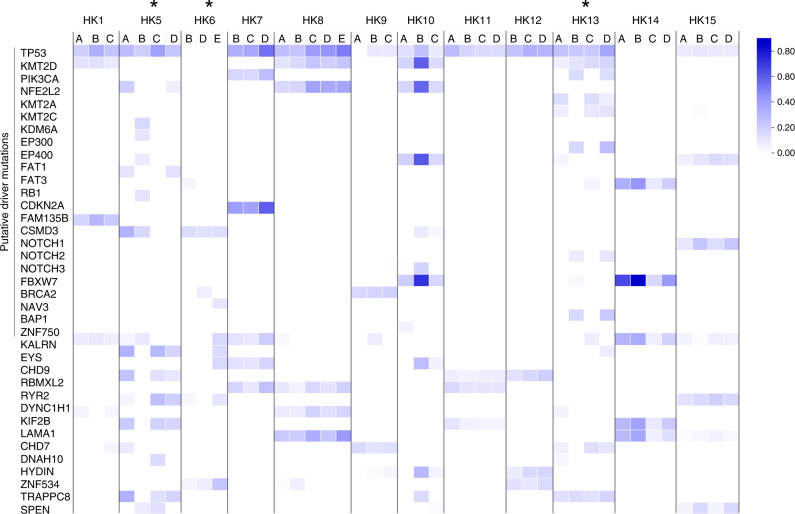
Cancer cell fraction (CCF) analysis. Clonal and subclonal status of putative ESCC driver and frequently mutated genes across patients. ^∗^denotes patients HK5, HK6, and HK13 showed the highest proportion of subclonal status among putative ESCC driver genes.

Various degrees of heterogeneity observed in 12 patients are summarised by numbers and distribution frequencies of non-silent trunk and branch mutations (Fig. 4b and Table S9). The heterogeneity frequency was calculated by dividing total non-silent branch mutations by patient-specific mutations. The median ITH of 12 patients with multiple ESCC regions was 37%, ranging from 15.9–100%, concordant with an earlier Japanese study with 13 patients with higher mean coverage depth of 150X [27]. The highest heterogeneity rate of 100 and 90% were observed in two patients, HK5 and HK13, followed by two other patients (HK6 and HK10) with 71 and 78%, while the majority of the remaining patients had <40% heterogeneity. Patients HK5 and HK13 had the highest number of branch (>300) and private (~250) mutations, compared to <100 branch and 70 private mutations observed in other patients (Fig. 4a, b). The clonal status of putative ESCC driver genes within each region is displayed as CCFs (Fig. 5), calculated after integration of CNVs, VAF and tumour cell purity [45, 46]. HK5, HK6, and HK13 showed the highest proportion of subclonal status among putative ESCC driver genes of 85% (11/13), 57% (4/7), 67% (10/15), respectively, concordant with results when only considering somatic non-silent mutations (Fig. 4a, b). For the eight dual primary cancer patients, both the recurrence and survival rates were 62.5% (5/8). For the ten ESCC patients, the recurrence and survival rates were 20% (2/10) and 10% (1/10), respectively. After a median follow-up of 25.5 months for twelve patients with multiple ESCC regions receiving surgery, two patients with the highest degree of ITH died. In addition to overall survival, the dual primary cancer patient, HK5, and two primary ESCC patients, HK6 and HK13, experienced the highest degree of heterogeneity based on the evidence from clonal status of putative driver mutations and frequently mutated genes in non-silent mutation heatmaps and CCF (Figs. 4a, b and 5) in this ESCC cohort of patients with relapse. HK5, HK6 and HK13 had the highest proportion of subclonal driver mutations associated with poorer overall survival (*p* = 0.045) and disease relapse (*p* = 0.005) (Table 2). To the best of our knowledge, earlier M-WES ESCC studies did not address the prognostic role of high tumoural ITH. The evidence presented here supports the hypothesis for the potential clinical usefulness of utilisation of high ITH as a prognostic indicator, but further larger cohort studies are needed to validate our findings.

## Conclusions

ESCC patients are at high risk to develop multiple primary cancers in the upper aerodigestive tract, especially at the hypopharynx and larynx [3, 47]. Our key findings of the alcohol exposure-related mutational signature present in all eight dual ESCC/HPC patients provide aetiologic insight implicating alcohol consumption being involved in dual ESCC/HPC carcinogenesis, as previously postulated by the field cancerization theory. Future verification studies are needed to substantiate this novel observation about alcohol-related mutational signature COSMIC 16 and analysis of dual synchronous ESCC/HPC cancers related to alcohol exposure. Current data from both clonality analysis and distinctive genomic profiles suggest multicentricity independent origin of clonal evolution rather than the clonal expansion of common neoplastic clones giving rise to dual primary ESCC/HPC. Our data indicate that M-WES assessment of ITH is needed to improve prognostication and lays the groundwork required to unravel the underlying genetic pathogenesis for the identification of novel therapeutic options and biomarkers for early screening, prediction and prognostication of treatment outcomes to improve survival for this deadly cancer.

## Supplementary information

Supplementary materials

Supplementary Table S3

Supplementory Table 6

Supplementory Table 7

Journal checklist summary

## Data Availability

All WES data are deposited at the European Genome-phenome Archive (EGA) with accession number (EGAS00001005966). All other data supporting the findings of this study are available from the corresponding author upon request.
