# Supplementary material for: Clonal relationship and alcohol consumption-associated mutational signature in synchronous hypopharyngeal tumours and oesophageal squamous cell carcinoma
Source: Br J Cancer. 2022 Oct 19;127(12):2166–74. doi: 10.1038/s41416-022-01995-0 (PMC9726980; doi:10.1038/s41416-022-01995-0)
Supplement: Supplementary file 1 — Supplementary materials [file 41416_2022_1995_MOESM1_ESM.pdf]

Supplementary methods

WES

DNA extraction using AllPrep DNA/RNA/miRNA Universal Kit (Qiagen) was performed only for the specimens with a cancer cell fraction (CCF) >30%. The matched blood samples of patients with multiple region tumor specimens were extracted with the QIAamp DNA Blood Mini Kit (Qiagen). The quality and quantity of extracted DNAs were evaluated on 0.7% agarose gels, with Nanodrop 1000 (Thermo Scientific) and Qubit dsDNA High Sensitivity Assay (Life Technologies). In brief, 500ng of genomic DNA was sheared into 300bp fragments with the M220 focused-ultrasonicator (Covaris) and then amplified with the KAPA HTP Library Preparation Kit (Roche). Amplified libraries were further captured with the SeqCap EZ Exome + UTR Kit (Roche) following the protocol. The libraries were quantified with KAPA Library Quantification Kit (Roche) through quantitative polymerase chain reaction (qPCR). The average fragment size of each library was examined with the High Sensitivity NGS Fragment Analysis Kit on the Fragment Analyzer Automated CE System (Advanced Analytical Technologies). The WES libraries were sequenced with the HiSeq sequencer (Illumina). The pre-processing and somatic short variant calling followed the Genome Analysis Toolkit (GATK) Best Practice using GATK4 (version 4.1.2). The raw reads were first aligned to the human reference genome version 19 (hg19) with Burrows-Wheeler Aligner (BWA) to generate a BAM file for each sample. This BAM file was further processed by Picard through sorting and marking duplicated reads. Then base quality recalibration was done through functions BaseRecalibrator and then ApplyBQSR to generate the recalibrated BAM file, which was ready for downstream variant calling. Germline variants were called from analysis-ready BAM files for each sample following the GATK Best Practice. The germline variant callset of each sample was evaluated regarding variant level concordance, genotype concordance, number of Indels & SNPs, the ratio of transition (Ti) to transversion (Tv) SNPs (TiTv ratio) and the ratio of insertions to deletions (INDEL Ratio).

The sequencing data of 165 ESCC patients from previous publications were downloaded for meta-analysis.[1-3] Two datasets were downloaded from Sequence Read Archive under accession number SRP033394 and from European Genome-phenome Archive under accession number EGAS00001000932. The first dataset included WES of 19 pairs of tumor/blood sets of and targeted sequencing of 119 tumor/blood sets captured using the SureSelect® Human All Exon 50M (Agilent Technologies) or customized beads and sequenced on HiSeq2000 (Illumina). The second dataset includes 113 pairs of tumor/blood sets captured using the SureSelect Human All Exon V4 kit (Agilent Technologies) and sequenced on the HiSeq 2000 platform (Illumina). Another inhouse dataset, previously published Hong Kong ESCC study includes 42 sets of tumor normal pairs captured using the Illumina TruSeq capture kit and sequenced with the HiSeq 1500 sequencer (Illumina, San Diego, CA, USA).[1] These samples were analyzed following the pipeline described above.

After removing duplicated reads, an average of 75% and 74% reads were left for blood and tumor, respectively, which met the acceptable duplication rate.

Identification of clonal and subclonal mutations

Single nucleotide variants (SNVs) and insertion and deletions (INDELs) were called simultaneously with Mutect2 following GATK Best Practice (v4.1.2) from matched tumor-normal pairs. The panel of normal (PON) file was generated from 24 blood samples, sequenced on the same platform to eliminate technical artifacts during somatic mutation calling. Somatic mutations were annotated by ANNOVAR (version 2017Jul16). The SNV PON file was generated from blood samples. A mutation was filtered out, if it had less than five reads supporting the alternative allele, or the variant allele frequency (VAF) was less than 10%. Any mutation reported to have minor allele frequency (MAF) greater than 1% in any of the public databases, including ESP6500 and in-house control (Hong Kong 895), was again filtered out. The mutations were manually examined by visualizing the BAM file reads in the Integrative Genomics Viewer (IGV). Mutations occurring only at the edge of reads, on low-quality reads, or in regions with an abnormally high load of mutations were excluded. Mutations recurring in all regions were considered clonal mutations. A gene level heatmap ranking the most frequent SNVs and CNVs across patients was generated, with each mutation labeled as trunk or branch mutations in different colors.

Phylogenetic tree analysis

The most likely phylogenetic trees of each patient were drawn with PHYLIP (PHYLogeny Inference Package). DrawTree package by counting the number of presence (1) and absence (0) of all exonic mutations. The trunk with clonal mutations was colored in red and the length is proportional to the number of mutations.

References:

1. Dai W, Ko JMY, Choi SSA et al. Whole-exome sequencing reveals critical genes underlying metastasis in oesophageal squamous cell carcinoma. J Pathol 2017; 242: 500-510.
2. Gao YB, Chen ZL, Li JG et al. Genetic landscape of esophageal squamous cell carcinoma. Nat Genet 2014; 46: 1097-1102.
3. Lin DC, Hao JJ, Nagata Y et al. Genomic and molecular characterization of esophageal squamous cell carcinoma. Nat Genet 2014; 46: 467-473.
4. Si HX, Tsao SW, Poon CS et al. Viral load of HPV in esophageal squamous cell carcinoma. Int J Cancer 2003; 103: 496-500.

List of supplementary tables

Supplementary Table 1 Carcinoma content of 56/68 regions of Hong Kong ESCC cohort with carcinoma content ≥30% without necrosis assessed by H&E staining selected for M-WES

Supplementary Table 2: Sanger sequencing validation of trunk and branch mutations

Supplementary Table 3: List of exonic non-silent mutations detected in all 18 patients (separate excel file)

Supplementary Table 4: Number of exonic, non-silent mutations detected in dual synchronous cancers

Supplementary Table 5: CNVs differentially expressed in ESCC and HP tumors in dual cancer patients.

Supplementary Table 6: Change of dominant subclones (separate excel file)

Supplementary Table 7: List of sharing mutations and CNVs in the shared clones (separate excel file)

Supplementary Table 8: Within-patient versus within-region non-silent mutation rates

Supplementary Table 9: Prevalence of subclonal mutations

List of supplementary figures

Figure S1 WES sequencing of multi-regional tumor, matched normal and blood samples from 18 patients

Figure S2 (A) Somatic mutation prevalence and (B) its distribution 8 dual synchronous HP/ESCC and 10 ESCC patients receiving surgical treatment analyzed by WES

Figure S3 (A) Heat maps and (B) river plots of Shanghai cohort of dual synchronous HP/ESCC patients

Figure S4 Copy number variations (CNVs) in ESCC typical regions shows high degree of inter- and intra-tumorigeneity

Figure S5 Phylogenetic trees and trunk/branch mutations in ten primary ESCC patients

Figure S6 HPV infection in dual primary and ESCC patients by PCR using consensus primer

Supplementary Table 1 Carcinoma content of 56/68 regions of 15 Hong Kong ESCC cohort with carcinoma content ≥30% without necrosis assessed by H&E staining selected for M-WES

**Patients**	**Multiple regions of esophageal tumors**						**Multiple regions of hypopharynx tumors**				
**Dual Primary**	**EA**	**EB**	**EC**	**ED**	**Regions selected for M-WES/Total regions assessed**		**HA**	**HB**	**HC**	**HD**	**Regions selected for M-WES/Total regions assessed**
HK1	60%	90%	90%	-	3/3		70%	60% Necrosis	50%	70% Necrosis	2/4
HK2	70%	10%	-	-	1/2		30%	40%	40%	-	3/3
HK3	30%	95%	-	-	2/2		60%	-	-	-	1/1
HK4	50% Necrosis	70%	0% Necrosis	-	1/3		70%	-	-	-	1/1
HK5	80%	667	50%	80%	4/4		-	-	-	-	-
**Primary ESCC**	**EA**	**EB**	**EC**	**ED**	**EE**	**EF**	**Regions selected for M-WES/Total regions assessed**				
HK6	0%	50%	10%	90%	30%	5%	3/6				
HK7	0%	60%	30%	30%	20%	5%	3/6				
HK8	80%	70%	90%	95%	95%	-	5/5				
HK9	30%	80%	95%	90%	-	-	4/4				
HK10	70%	80%	80%	20%	-	-	3/4				
HK11	50%	70%	50%	90%	-	-	4/4				
HK12	30%	70%	60%	50%	-	-	4/4				
HK13	70%	90%	50%	40%	-	-	4/4				
HK14	80%	70%	50%	80%	-	-	4/4				
HK15	30%	40%	95%	80%	-	-	4/4				

Supplementary Table 2: Sanger sequencing validation of trunk and branch mutations

Gene	Chr	Position	Ref	Alt	Sample ID	Mutation	NGS result	Validation
RBMXL2	chr11	7111484	G	A	HK11EA	trunk	present	Yes
					HK11EB	trunk	present	Yes
					HK11EC	trunk	present	Yes
					HK11ED	trunk	present	Yes
RBMXL2	chr11	7111415	A	G	HK8EA	trunk	present	Yes
					HK8EB	trunk	present	Yes
					HK8EC	trunk	present	Yes
					HK8ED	trunk	present	Yes
					HK8EE	trunk	present	Yes
TP53	chr17	7579415	C	T	HK8EA	trunk	present	Yes
					HK8EB	trunk	present	Yes
					HK8EC	trunk	present	Yes
					HK8ED	trunk	present	Yes
					HK8EE	trunk	present	Yes
TP53	chr17	7578265	A	T	HK10EA	trunk	present	Yes
					HK10EB	trunk	present	Yes
					HK10EC	trunk	present	Yes
TP53	chr17	7578253	C	A	HK11EA	trunk	present	Yes
					HK11EB	trunk	present	Yes
					HK11EC	trunk	present	Yes
					HK11ED	trunk	present	Yes
TP53	chr17	7578406	C	T	HK12EB	trunk	present	Yes
					HK12EC	trunk	present	Yes
					HK12ED	trunk	present	Yes
TP53	chr17	7578188	C	A	HK13EA	trunk	present	Yes
					HK13EB	trunk	present	Yes
					HK13EC	trunk	present	Yes
					HK13ED	trunk	present	Yes
KMT2D	chr12	49433646	G	C	HK13EA	trunk	present	Yes
					HK13EB	trunk	present	Yes
					HK13EC	trunk	present	Yes
					HK13ED	trunk	present	Yes
KMT2D	chr12	49444552	G	A	HK13EA	branch	present	Yes
					HK13EB		absent	
					HK13EC		absent	
					HK13ED		absent	
KMT2D	chr12	49431346	G	T	HK13EA		absent	
					HK13EB		absent	
					HK13EC	branch	present	Yes
					HK13ED		absent	
KMT2D	chr12	49422997	C	A	HK10TB	trunk	present	Yes
KMT2D	chr12	49433227	-	G	HK8EA	trunk	present	Yes
					HK8EB	trunk	present	Yes
					HK8EC	trunk	present	Yes
					HK8ED	trunk	present	Yes
					HK8EE	trunk	present	Yes
NFE2L2	chr2	178098944	C	A	HK8EA	trunk	present	Yes
					HK8EB	trunk	present	Yes
					HK8EC	trunk	present	Yes
					HK8ED	trunk	present	Yes
					HK8EE	trunk	present	Yes
EYS	chr6	65300683	A	C	HK6EB		absent	
					HK6ED		absent	
					HK6EE	branch	present	X

EYS	chr6	66204699	C	A	ESCC118TB	trunk	present	Yes
					ESCC118TC	trunk	present	Yes
PIK3CA	chr3	178936091	G	A	ESCC118TB	trunk	present	Yes
LAMA1	chr18	7012062	A	C	HK8EB	trunk	present	Yes
					HK8EC	trunk	present	Yes
					HK8ED	trunk	present	Yes
					HK8EE	trunk	present	Yes
FAT1	chr4	187629400	C	A	HK10EA	trunk	present	Yes
					HK10EB	trunk	present	Yes
					HK10EC	trunk	present	Yes
FBXW7	chr4	153245450	G	A	HK10EA	trunk	present	Yes
					HK10EB	trunk	present	Yes
					HK10EC	trunk	present	Yes
EYS	chr6	65300314	C	G	HK10EA		absent	
					HK10EB	branch	present	Yes
					HK10EC	branch	present	Yes
KIF2B	chr17	51902185	G	T	HK11EA	trunk	present	Yes
					HK11EB	trunk	present	Yes
					HK11EC	trunk	present	Yes
					HK11ED	trunk	present	Yes
PIK3CA	chr3	178936091	G	A	HK13EA		absent	
					HK13EB	branch	present	**X**
					HK13EC		absent	
					HK13ED		absent	
PIK3CA	chr3	178952074	G	A	HK13EA		absent	
					HK13EB	branch	present	Yes
					HK13EC		absent	
					HK13ED	branch	present	Yes
LAMA1	chr18	7037660	C	G	HK14EA	trunk	present	Yes
					HK14EB	trunk	present	Yes
					HK14EC	trunk	present	Yes
					HK14ED	trunk	present	Yes
FBXW7	chr4	153249393	G	A	HK14EA	trunk	present	Yes
					HK14EB	trunk	present	Yes
					HK14EC	trunk	present	Yes
					HK14ED	trunk	present	Yes
KIF2B	chr17	51901296	C	A	HK14EA	trunk	present	Yes
					HK14EB	trunk	present	Yes
					HK14EC	trunk	present	Yes
					HK14ED	trunk	present	Yes
TP53	chr17	7578212	G	A	HK15EA	trunk	present	Yes
					HK15EB	trunk	present	Yes
					HK15EC	trunk	present	Yes
					HK15ED	trunk	present	Yes
FAT1	chr4	187549845	−	A	HK15EA	trunk	present	Yes
					HK15EB	trunk	present	Yes
					HK15EC	trunk	present	Yes
					HK15ED	trunk	present	Yes
NOTCH1	chr9	139409055	G	A	HK15EA	trunk	present	Yes
					HK15EB	trunk	present	Yes
					HK15EC	trunk	present	Yes
					HK15ED	trunk	present	Yes
FAT1	chr4	187516980	G	T	HK13EA	branch	present	Yes
					HK13EB		absent	
					HK13EC		absent	
					HK13ED		absent	

Supplementary Table 4: Number of exonic, non-silent mutations detected in dual synchronous cancers

Sample ID	Esophageal tumor No. of exonic non-silent mutations	Hypopharyngeal tumor No. of exonic non-silent mutations	No. of shared non-silent mutations	No. of shared genes	List of shared genes
HK1	151	98	0	3	*DNAH17*<br>*GOLGA6L2*<br>*TTN*
HK2	87	95	0	2	*PKP4*<br>*TP53*
HK3	219	328	0	9	*ALG13*<br>*COL3A1*<br>*CSMD3*<br>*CTNND2*<br>*FSIP2*<br>*MAGEB18*<br>*RYR2*<br>*TP53*<br>*ZNF623*
HK4	92	155	0	3	*TP53*<br>*ZNFX4*<br>*TTN*
SH1	162	146	3	3	*FSIP2*<br>*CEO135*<br>*TP53*
SH2	74	13	4	4	*OPTN*<br>*RNF121*<br>*SEC23A*<br>*TP53*
SH3	219	62	1	1	*MYO1H*

Supplementary Table 5: CNVs differentially expressed in ESCC and HP tumors in dual cancer patients.

chromosome	start	end	genes	ESCC (log 2, >0 gain, <0 loss)	HP tumor (log 2, >0 gain, <0 loss)	*p*	fdr
chr1	7998187	7999934	AL034417.3	-0.020	0.237	0.001695	0.146008
chr1	15465909	15491400	CELA2B	-0.125	0.112	0.002519	0.171267
chr1	15847864	15940460	SPEN	-0.091	0.133	0.003741	0.198653
chr1	16460948	16468481	LINC01772	-0.088	0.204	0.00233	0.164229
chr1	20651767	20661544	DDOST	-0.169	0.063	0.001439	0.143016
chr1	153746851	153751227	AL513523.4	0.184	0.422	0.003411	0.197358
chr2	27253684	27275817	SLC30A3	-0.021	0.253	0.003411	0.197358
chr2	97372532	97372835	IGKV2OR2-7	0.017	0.209	0.001695	0.146008
chr2	99181079	99197626	MRPL30	0.024	0.349	0.001838	0.151547
chr2	143937073	143964156	AC016910.1	-0.114	0.143	0.000942	0.117764
chr3	140678039	140701150	TRIM42	0.327	0.647	0.003674	0.197774
chr3	150852484	151080726	CLRN1-AS1	0.394	0.730	0.000863	0.117764
chr3	164714095	164831480	LINC01324	0.246	0.556	0.001695	0.146008
chr3	169939353	169966734	AC008040.1	0.426	0.741	0.001562	0.146008
chr3	184097064	184106995	HTR3E	0.487	0.861	0.003166	0.191721
chr3	184399790	184457891	LINC02054	0.458	0.900	0.003166	0.191721
chr3	184741937	184742462	AC107294.3	0.469	0.879	0.000942	0.117764
chr4	122732702	122744943	BBS12	-0.358	-0.073	0.003411	0.197358
chr5	92151	189972	PLEKHG4B	0.244	-0.305	0.000942	0.117764
chr5	10249921	10266412	CCT5	0.324	0.058	0.00272	0.178694
chr5	13690331	13944543	DNAH5	0.377	0.005	0.000661	0.116747
chr5	17498231	17498827	AC106774.8	0.188	-0.238	0.00233	0.164229
chr5	33986178	34008108	AMACR	0.222	-0.065	0.001695	0.146008
chr5	34905264	34918989	RAD1	0.216	-0.066	0.000457	0.110624
chr5	135648584	135653935	LINC01959	-0.188	-0.477	0.003674	0.197774
chr11	68312609	68449275	LRP5	0.276	0.893	0.003166	0.191721
chr11	76860867	77026797	ACER3	-0.139	0.309	0.003674	0.197774
chr12	122687125	122715979	AC026333.3	-0.182	0.140	0.001991	0.154311
chr12	123262060	123262402	AC068768.2	-0.138	0.115	0.001324	0.142578
chr12	132603150	132610543	LRCOL1	-0.171	0.160	0.000281	0.08392
chr12	133181409	133214831	ZNF268	0.010	0.300	0.000661	0.116747
chr14	22070557	22071208	TRAV22	0.066	-0.349	0.003166	0.191721
chr14	22314490	22314919	TRAV40	0.097	-0.255	0.00011	0.052799
chr16	3365099	3479550	AC025283.3	-0.026	-0.338	0.000863	0.117764
chr16	4696510	4734378	ANKS3	0.070	-0.142	0.00055	0.116747
chr17	34142947	34147047	AC004147.3	0.098	-0.136	0.00272	0.178694

chr17	48646923	48707346	LINC02086	0.186	0.387	0.002155	0.163751
chr18	12407896	12432238	PRELID3A	0.032	-0.400	0.002519	0.171267
chr18	14104542	14105226	AC006557.3	-0.122	-0.533	0.001838	0.151547
chr19	8321158	8323340	RPS28	0.208	-0.131	0.001562	0.146008
chr19	46838136	46850992	AP2S1	-0.154	0.232	0.001324	0.142578
chr19	50480119	50483351	AC020909.3	-0.145	-0.375	0.001324	0.142578
chr22	17580157	17589192	AC007666.1	-0.035	-0.307	0.000723	0.116747
chr22	18110759	18131154	AC008079.2	0.045	-0.160	0.003674	0.197774
chr22	18605815	18611919	RIMBP3	0.044	-0.250	6.24E-05	0.034552
chr22	19130808	19132623	TSSK2	0.471	-0.133	3.63E-07	0.000704
chr22	19756703	19783593	TBX1	0.136	-0.120	0.00079	0.117764
chr22	20707691	20859417	PI4KA	0.236	-0.065	0.001439	0.143016
chr22	21114607	21124451	AP000550.1	0.192	-0.052	0.000942	0.117764
chr22	21354563	21355763	LINC01651	-0.009	-0.239	0.001027	0.124386
chr22	22380766	22381347	IGLV1-44	0.284	-0.185	0.003674	0.197774
chr22	22513736	22520270	ZNF280A	-0.036	-0.320	0.002935	0.189632
chr22	22697789	22698407	IGLV2-23	0.022	-0.160	0.00233	0.164229
chr22	22711689	22713203	IGLV3-21	0.038	-0.230	0.000123	0.052799
chr22	22887780	22896107	IGLL5	0.021	-0.214	0.000255	0.082269
chr22	22893692	22893818	IGLJ1	0.117	-0.124	0.000255	0.082269
chr22	22899481	22899655	IGLJ2	0.000	-0.238	3.42E-05	0.022081
chr22	23145326	23164350	RAB36	-0.021	-0.324	2.34E-05	0.018114
chr22	23957414	23961186	GSTT2B	0.003	-0.195	0.001991	0.154311
chr22	26858634	26865786	LINC01422	0.069	-0.149	0.000457	0.110624
chr22	29438583	29442455	RFPL1	0.094	-0.314	1.04E-05	0.010077
chr22	29720084	29731839	CABP7	0.026	-0.249	0.000661	0.116747
chr22	30652051	30667890	DUSP18	0.298	-0.056	0.000723	0.116747
chr22	32043032	32113029	SLC5A1	0.147	-0.159	5.82E-06	0.007516
chr22	37570246	37582616	LGALS2	0.183	-0.102	0.000723	0.116747
chr22	38219291	38273034	TMEM184B	0.052	-0.286	0.000377	0.104499
chr22	41245611	41286251	RANGAP1	0.175	-0.134	0.001439	0.143016
chr22	41909554	41914667	SHISA8	0.041	-0.215	0.00233	0.164229
chr22	41922023	41926818	TNFRSF13C	0.069	-0.164	0.000661	0.116747
chr22	41981304	41982217	Z99716.1	0.058	-0.222	0.001119	0.131396
chr22	48489460	48850912	FAM19A5	-0.033	-0.552	0.000187	0.07267
chr22	50316035	50317025	CR559946.2	0.169	-0.255	0.001991	0.154311
chr22	50674415	50733298	SHANK3	0.238	-0.310	1.24E-07	0.00048

Cancer relevant genes are highlighted in red.

Supplementary Table 8: Within-patient versus within-region non-silent mutation rates

Patients	Multiple regions of esophageal tumors (E)					Multiple regions of hypopharynx tumors (H)				
Dual Primary	EA	EB	EC	ED	Mutations in patient (E)	HA	HB	HC	HD	Mutations in patient (H)
HK1	135	93	115	-	151	97	-	74	-	98
HK2	87	-			87	69	66	84	-	94
HK5	111	170	94	98	329	-				-

Primary ESCC	EA	EB	EC	ED	EE	Mutations in patient (E)
HK6	-	59	-	88	105	140
HK7	-	72	74	71	-	89
HK8	93	96	99	98	100	107
HK9	77	112	107	21*	-	119
HK10	43	86	42	-	-	107
HK11	71	58	70	60	-	79
HK12	-	77	82	77	-	95
HK13	234	127	187	181	-	427
HK14	60	59	61	60	-	68
HK15	225	263	193	256	-	271

* Samples HK2-EB (0.5 mutations per Mb) and HK9-ED (0.7 mutations per Mb) were removed from downstream mutation analysis due to low mutations would lead to inaccurate, biased results.

Supplementary Table 9: Prevalence of subclonal mutations

**Patients**	**Intra-tumor prevalence of esophageal tumors (E) (%)**					**Intra-tumor prevalence of hypopharynx tumors (H) (%)**				
**Dual Primary**	**EA**	**EB**	**EC**	**ED**	**Within-patient prevalence (%)**	**HA**	**HB**	**HC**	**HD**	**Within-patient prevalence (%)**
HK1	30.5	2.6	19.2	-	41.1	9.6	-	0.4	-	70.7
HK2	-	-			-	8.8	8.2	17.0	-	70.9
HK5	33.7	51.7	28.6	29.8	**100.0**	-				-
**Primary ESCC**	**EA**	**EB**	**EC**	**ED**	**EE**	**Mutations$_E$ in patient (E)**				
HK6	-	12.9	-	33.6	45.7	70.7				
HK7	-	15.7	18.0	14.6	-	34.8				
HK8	2.8	5.6	8.4	7.5	9.3	15.9				
HK9	2.5	31.1	26.9	-	-	37.0				
HK10	17.8	57.9	16.8	-	-	77.6				
HK11	25.3	8.9	24.1	11.4	-	35.4				
HK12	-	8.4	13.7	8.4	-	27.4				
HK13	44.7	19.7	33.7	32.3	-	**89.9**				
HK14	13.2	11.8	14.7	13.2	-	25.0				
HK15	18.8	32.8	7.0	30.3	-	35.8				

Red font signifies the two patients with ~90-100% prevalence of subclonal mutations in the ESCC.

Figure S1 WES sequencing of multi-regional tumor, matched normal and blood samples from 18 patients. (A) Percentage of unique reads, unique reads aligned to human genome, and on the captured whole-exome regions. (B) The mean target coverage of matched blood (n=15), normal (n=6), and tumor samples (n=60)

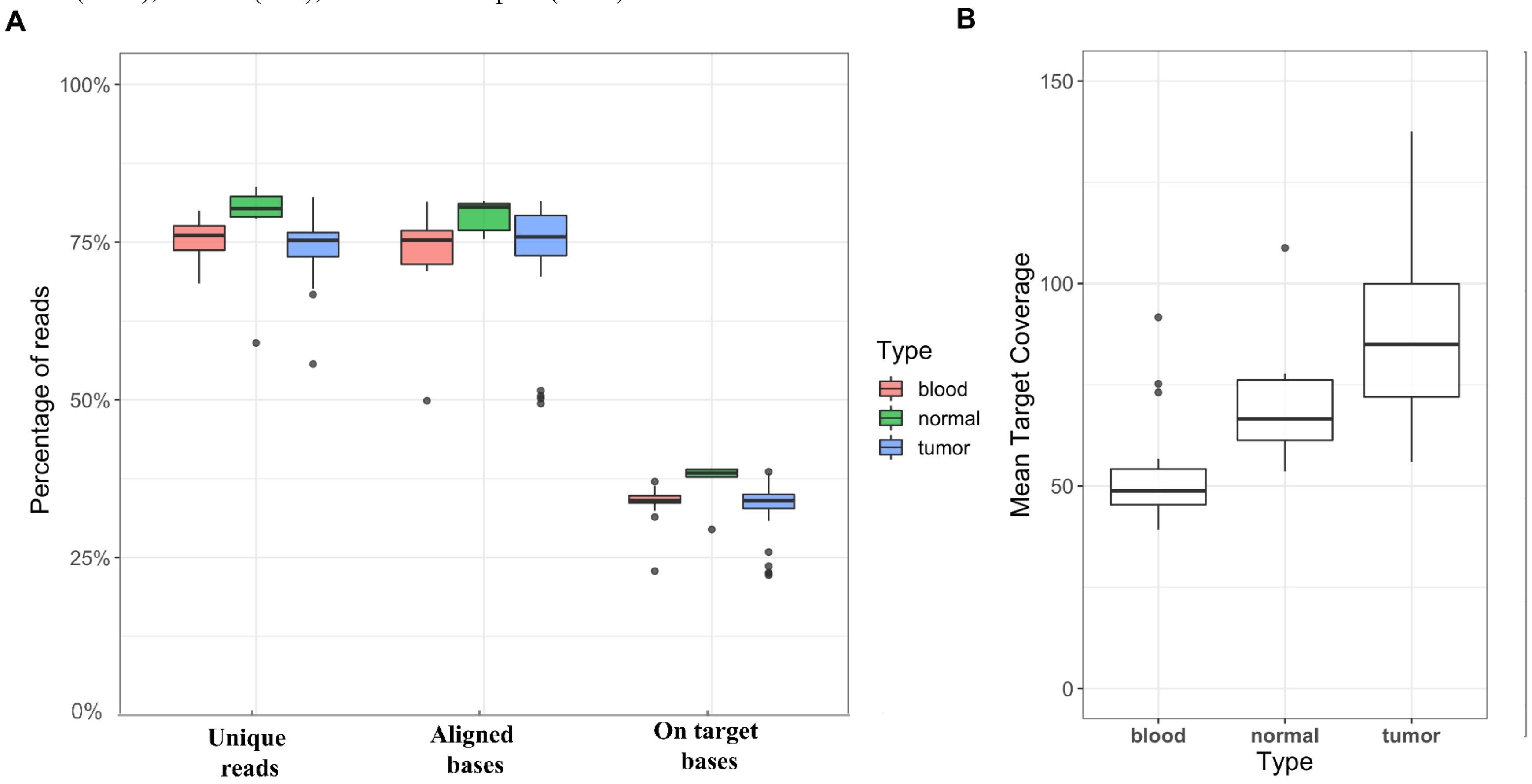

Figure S2 (A) Somatic mutation prevalence and (B) its distribution in 8 dual synchronous HP/ESCC and 10 ESCC patients receiving surgical treatment analyzed by WES

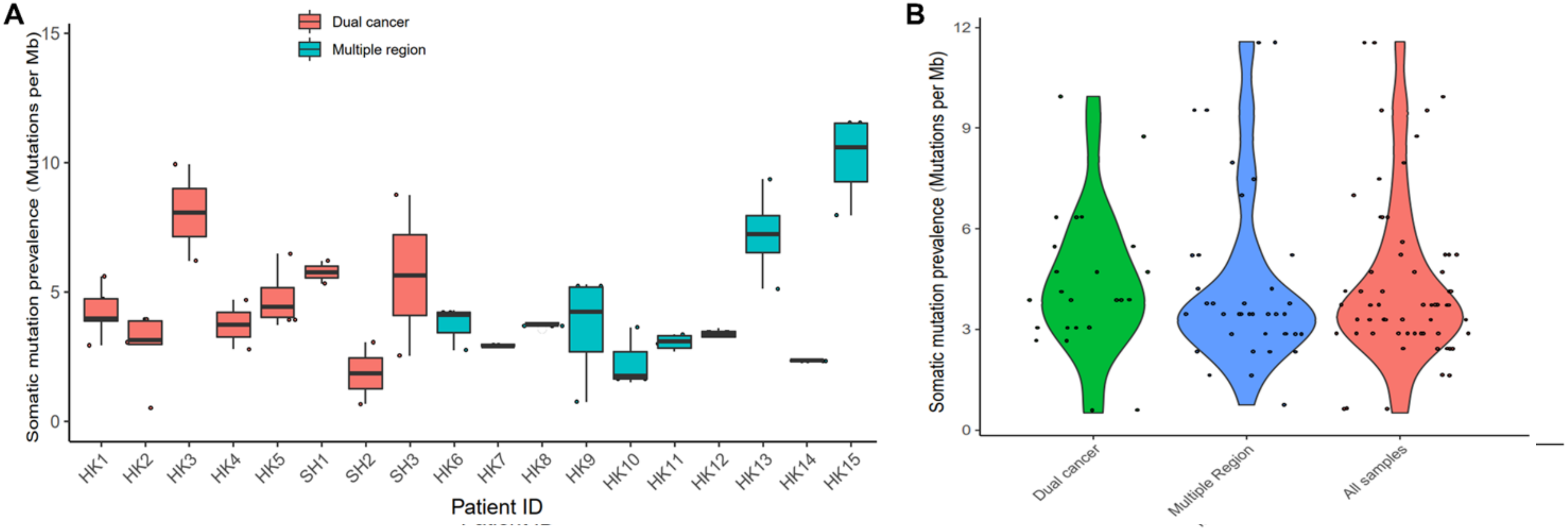

Figure S3 (A) Heat maps and (B) river plots of Shanghai cohort of dual synchronous HP/ESCC patients

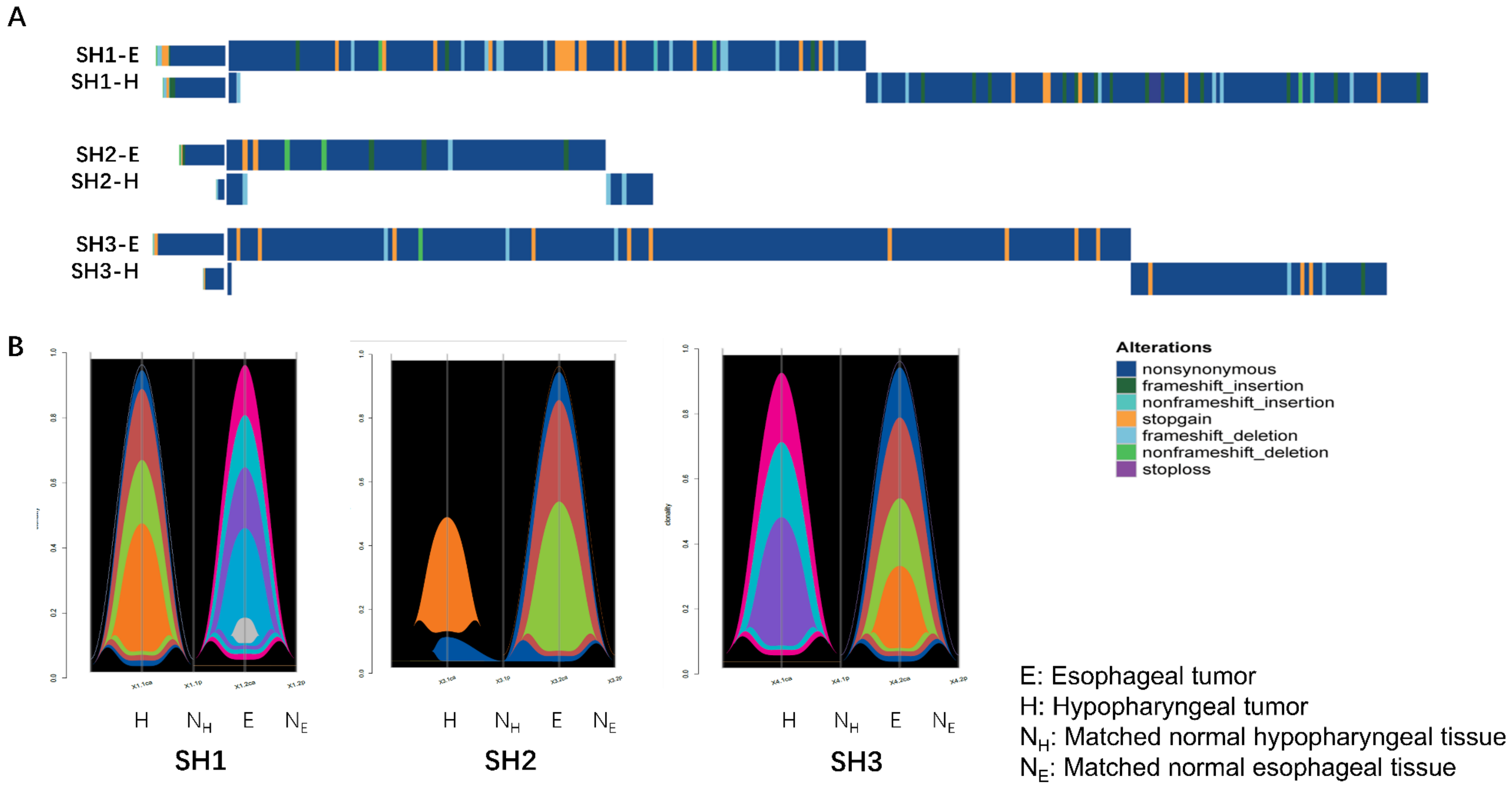

Figure S4 Copy number variations (CNVs) in ESCC typical regions show high degree of inter- and intra-tumor heterogeneity in ten genes

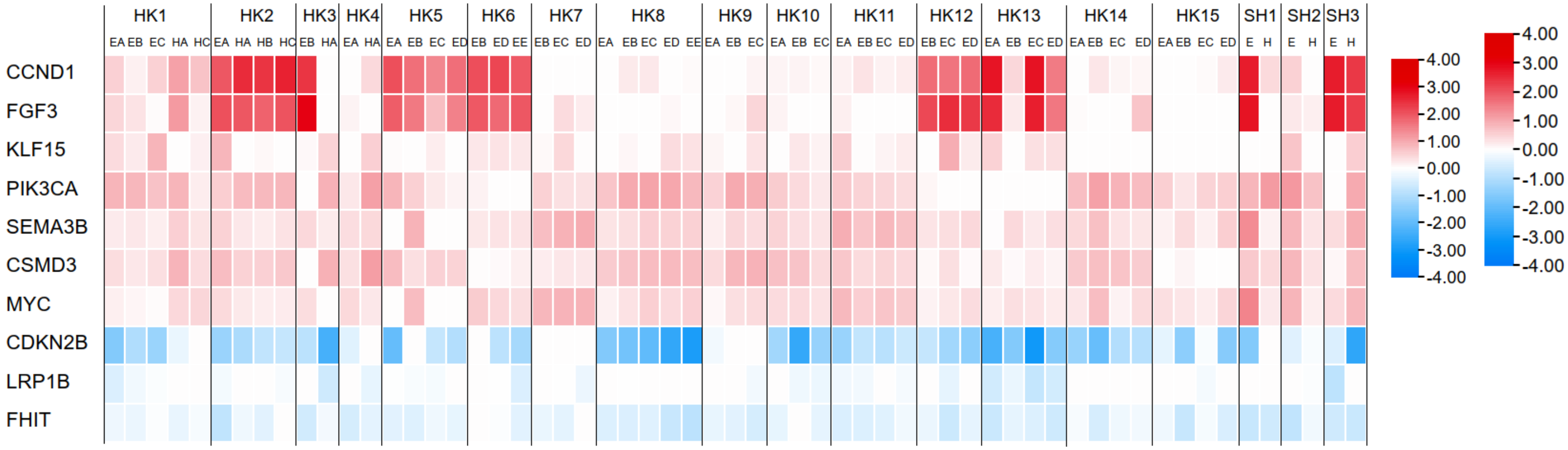

Figure S5 Phylogenetic tree, trunk, and branch mutations in ten primary ESCC patients

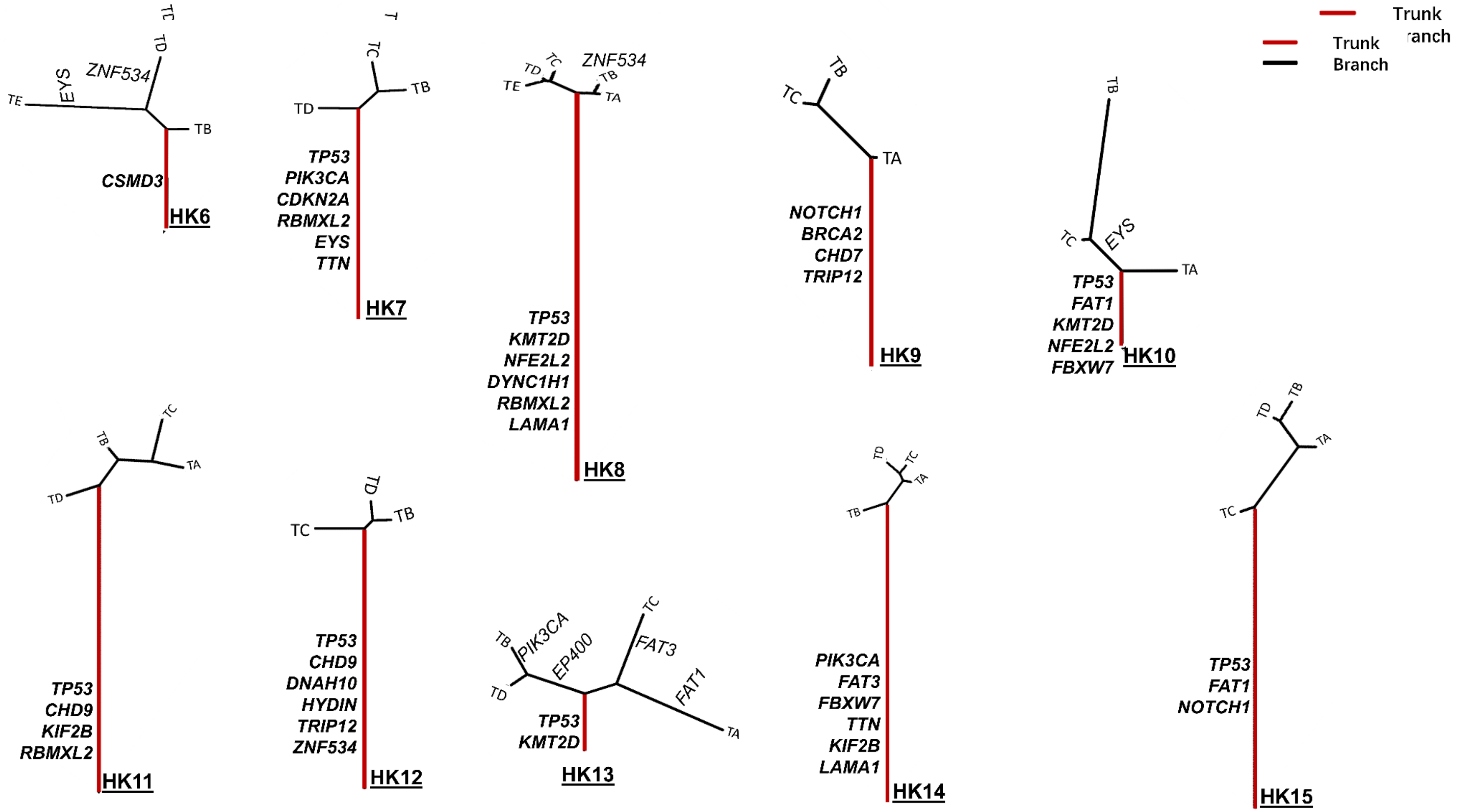

Figure S6 HPV infection in dual primary and ESCC patients by PCR using consensus primer

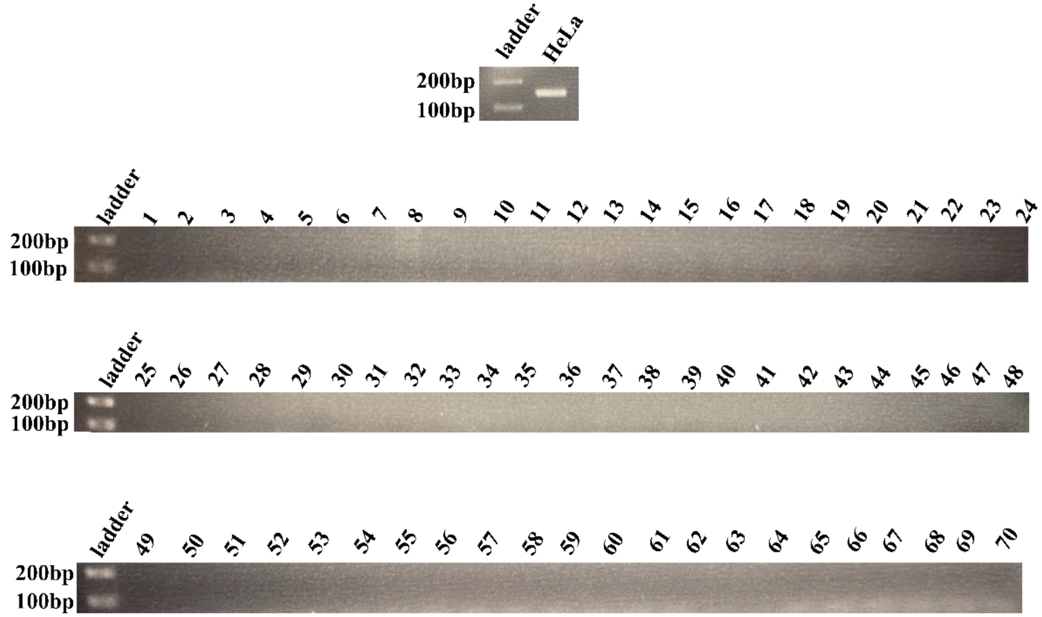

#	Sample name	#	Sample name	#	Sample name
1	HK1-EA	25	HK7-EB	49	HK13-EC
2	HK1-EB	26	HK7-EC	50	HK13-ED
3	HK1-EC	27	HK7-ED	51	HK14-EA
4	HK1-HA	28	HK8-EA	52	HK14-EB
5	HK1-HC	29	HK8-EB	53	HK14-EC
6	HK2-EA	30	HK8-EC	54	HK14-ED
7	HK2-EB	31	HK8-ED	55	HK15-EA
8	HK2-HA	32	HK8-EE	56	HK15-EB
9	HK2-HB	33	HK9-EA	57	HK15-EC
10	HK2-HC	34	HK9-EB	58	HK15-ED
11	HK3-EA	35	HK9-EC	59	SH1-H-Tumor
12	HK3-EB	36	HK9-ED	60	SH1-H-Normal
13	HK3-HA	37	HK10-EA	61	SH1-E-Tumor
14	HK3-HB	38	HK10-EB	62	SH1-E-Normal
15	HK3-HC	39	HK10-EC	63	SH2-H-Tumor
16	HK4-HA	40	HK11-EA	64	SH2-H-Normal
17	HK4-EA	41	HK11-EB	65	SH2-E-Tumor
18	HK5-EA	42	HK11-EC	66	SH2-E-Normal
19	HK5-EB	43	HK11-ED	67	SH3-H-Tumor
20	HK5-EC	44	HK12-EB	68	SH3-H-Normal
21	HK5-ED	45	HK12-EC	69	SH3-E-Tumor
22	HK6-EB	46	HK12-ED	70	SH3-E-Normal
23	HK6-ED	47	HK13-EA		
24	HK6-EE	48	HK13-EB		

PCR screening with GP5+/GP6+ primers as shown in the following:

GP5+: TTTGTTACTGTGGTAGATACTAC

GP6+: GAAAAATAAACTGTAAATCATATTC

The amplicon size is 150 bp and the details of PCR amplification was as previously described.[4] HeLa is the positive cell line with HPV infection. HPV DNA was not detected in all 18 patients.
