# Supplementary material for: Clonal relationship and alcohol consumption-associated mutational signature in synchronous hypopharyngeal tumours and oesophageal squamous cell carcinoma
Source: Br J Cancer. 2022 Oct 19;127(12):2166–74. doi: 10.1038/s41416-022-01995-0 (PMC9726980; doi:10.1038/s41416-022-01995-0)
Supplement: Supplementary file 2 — Supplementary Table S3 [file 41416_2022_1995_MOESM2_ESM.pdf]

Gene	Chromosome	Start	End	Ref	Alt	SH1-E	SH1-H
FSIP2	chr2	186669236	186669236	A	C	nonsynonymous	nonsynonymous
TP53	chr17	7577120	7577120	C	T	nonsynonymous	nonsynonymous
TTN	chr2	179584356	179584356	C	A	nonsynonymous	nonsynonymous
ABCG2	chr4	89061015	89061015	G	C	nonsynonymous	NA
ABHD11	chr7	73153054	73153054	C	T	nonsynonymous	NA
ALS2CR12	chr2	202216069	202216069	C	T	nonsynonymous	NA
ANKRD13A	chr12	110463620	110463620	G	C	nonsynonymous	NA
ANKRD36C	chr2	96533222	96533222	T	C	nonsynonymous	NA
ANO5	chr11	22279262	22279262	C	G	nonsynonymous	NA
AP1G2	chr14	24035141	24035141	G	A	nonsynonymous	NA
APAF1	chr12	99042410	99042410	C	G	nonsynonymous	NA
APC	chr5	112179693	112179693	G	C	nonsynonymous	NA
ARHGAP35	chr19	47425296	47425296	A	G	nonsynonymous	NA
ARID1A	chr1	27088799	27088799	A	G	nonsynonymous	NA
ARID4B	chr1	235397783	235397783	C	G	nonsynonymous	NA
ARL5B	chr10	18957493	18957493	A	G	nonsynonymous	NA
ARMC2	chr6	109232178	109232178	C	G	nonsynonymous	NA
ARPC1B	chr7	98988606	98988606	-	T	frameshift_insertion	NA
ASPH	chr8	62438625	62438625	A	G	nonsynonymous	NA
ATP5J2-PTCD1;PTCD1	chr7	99017735	99017735	C	T	nonsynonymous	NA
ATRX	chrX	76949371	76949371	T	A	nonsynonymous	NA
AXIN2	chr17	63531834	63531834	C	G	nonsynonymous	NA
BAZ2B	chr2	160206866	160206866	G	T	nonsynonymous	NA
BIRC2	chr11	102248743	102248743	G	C	nonsynonymous	NA
BRD3	chr9	136913401	136913401	T	A	nonsynonymous	NA
BRWD3	chrX	79945475	79945475	C	T	nonsynonymous	NA
CACNA1E	chr1	181701600	181701600	G	T	nonsynonymous	NA
CAMSAP3	chr19	7677084	7677084	G	T	stopgain	NA
CDC73	chr1	193094321	193094321	C	T	nonsynonymous	NA
CDT1	chr16	88871020	88871020	A	G	nonsynonymous	NA
CDX4	chrX	72667120	72667120	G	A	nonsynonymous	NA
CELSR1	chr22	46835108	46835118	AGCGCTGTCTC	-	frameshift_deletion	NA
CENPF	chr1	214819346	214819346	C	T	nonsynonymous	NA
CFAP221	chr2	120362255	120362255	G	C	nonsynonymous	NA
CNDP2	chr18	72185819	72185819	G	C	nonsynonymous	NA
COLGALT2	chr1	183920222	183920222	C	G	nonsynonymous	NA
CRYBB1	chr22	26997913	26997913	C	A	nonsynonymous	NA
CSMD3	chr8	113516135	113516135	C	A	nonsynonymous	NA
CT47B1	chrX	120009227	120009241	CCTCTTCCTCCTCCT	-	nonframeshift_deletion	NA
CTPS2	chrX	16720874	16720874	G	T	stopgain	NA
CUL9	chr6	43167083	43167083	C	G	nonsynonymous	NA
DCAF8L2	chrX	27765393	27765393	G	T	nonsynonymous	NA
DCHS2	chr4	155411978	155411978	G	C	nonsynonymous	NA
DDX31	chr9	135545434	135545434	G	C	nonsynonymous	NA
EID3	chr12	104698487	104698487	A	T	nonsynonymous	NA
ERBB4	chr2	212285243	212285243	C	T	nonsynonymous	NA
ERICH2	chr2	171627474	171627474	G	T	nonsynonymous	NA
ERV3-1	chr7	64452283	64452283	C	A	nonsynonymous	NA
FAM129A	chr1	184787899	184787899	G	C	nonsynonymous	NA
FAM155A	chr13	108518560	108518560	G	A	nonsynonymous	NA
FANCA	chr16	89838220	89838220	T	C	nonsynonymous	NA
FARSB	chr2	223489113	223489113	C	T	nonsynonymous	NA
FASTKD3	chr5	7861750	7861750	A	C	stopgain	NA
FBN2	chr5	127873289	127873289	C	T	nonsynonymous	NA
FBRS	chr16	30671262	30671262	C	A	nonsynonymous	NA
FMO4	chr1	171292243	171292243	-	T	frameshift_insertion	NA
FRMD1	chr6	168459832	168459832	C	G	nonsynonymous	NA
FSTL5	chr4	162463837	162463837	C	G	nonsynonymous	NA
GON4L	chr1	155747481	155747481	C	G	nonsynonymous	NA
GRAMD4	chr22	47068788	47068797	GCTGCCCGAG	-	frameshift_deletion	NA
GRHL2	chr8	102570742	102570742	A	G	nonsynonymous	NA
HEATR5A	chr14	31792782	31792782	C	G	nonsynonymous	NA
HIST1H2AB	chr6	26033433	26033433	C	G	nonsynonymous	NA
HIST1H2BB	chr6	26043793	26043793	C	G	nonsynonymous	NA
HIST1H4D	chr6	26189241	26189241	C	G	nonsynonymous	NA
IGF1R	chr15	99478275	99478282	ACCATGTG	-	frameshift_deletion	NA
IKZF2	chr2	213872794	213872794	G	A	stopgain	NA
IL12RB2	chr1	67833588	67833588	G	C	nonsynonymous	NA
IL21	chr4	123533881	123533881	C	-	frameshift_deletion	NA
IMPDH2	chr3	49062374	49062374	A	-	frameshift_deletion	NA
IRF2BP2	chr1	234744651	234744651	G	C	nonsynonymous	NA
IRF2BP2	chr1	234745209	234745209	G	A	nonsynonymous	NA
ITGAE	chr17	3659186	3659186	C	G	nonsynonymous	NA
KCNJ4	chr22	38822829	38822829	T	A	nonsynonymous	NA

KIF26B	chr1	245851952	245851952	C	G	nonsynonymous	NA
LDB1	chr10	103868922	103868922	C	T	nonsynonymous	NA
LRP2	chr2	169985578	169985578	A	G	nonsynonymous	NA
MADD	chr11	47330177	47330177	G	C	nonsynonymous	NA
MGAM2	chr7	141838366	141838366	C	G	nonsynonymous	NA
MIA3	chr1	222824021	222824021	A	G	nonsynonymous	NA
MRPS9	chr2	105665650	105665650	G	A	nonsynonymous	NA
MTSS1	chr8	125565820	125565820	T	-	frameshift_deletion	NA
MTUS1	chr8	17611401	17611401	C	A	nonsynonymous	NA
MUC16	chr19	8968922	8968922	C	T	nonsynonymous	NA
MXI1	chr10	112044584	112044584	G	T	stopgain	NA
MYDGF	chr19	4659970	4659970	C	A	stopgain	NA
MYH1	chr17	10399572	10399572	G	A	stopgain	NA
NCAM2	chr21	22746201	22746201	G	T	stopgain	NA
NELL1	chr11	21594916	21594916	G	A	stopgain	NA
NFATC1	chr18	77208917	77208917	G	A	nonsynonymous	NA
OAS1	chr12	113348969	113348969	A	T	stopgain	NA
OFD1	chrX	13767549	13767549	G	T	stopgain	NA
OPTN	chr10	13164392	13164392	G	T	nonsynonymous	NA
OR4N4	chr15	22382962	22382962	C	A	nonsynonymous	NA
OR5T2	chr11	56000061	56000061	C	A	nonsynonymous	NA
PAPPA2	chr1	176708858	176708858	C	T	nonsynonymous	NA
PCSK5	chr9	78804091	78804091	G	A	nonsynonymous	NA
PIGH	chr14	68056853	68056853	A	G	nonsynonymous	NA
PMEPA1	chr20	56234647	56234647	G	T	nonsynonymous	NA
PNN	chr14	39650342	39650342	C	T	stopgain	NA
PPFIA2	chr12	81768473	81768473	C	A	nonsynonymous	NA
PPP4R3A	chr14	91948033	91948033	G	A	stopgain	NA
PPP4R3B	chr2	55795466	55795466	A	C	nonsynonymous	NA
PRSS53	chr16	31097957	31097957	C	T	nonsynonymous	NA
PSMB5	chr14	23503963	23503963	G	A	nonsynonymous	NA
PTEN	chr10	89692883	89692883	C	G	nonsynonymous	NA
PTPN22	chr1	114377603	114377603	C	G	nonsynonymous	NA
RB1CC1	chr8	53573197	53573197	G	A	nonsynonymous	NA
REPS2	chrX	17065598	17065598	C	A	nonsynonymous	NA
RITA1	chr12	113629240	113629240	-	GCCACA	nonframeshift_insertion	NA
RLF	chr1	40703600	40703600	A	G	nonsynonymous	NA
RNF150	chr4	142053514	142053514	G	A	nonsynonymous	NA
RNF19A	chr8	101287213	101287213	G	C	nonsynonymous	NA
ROM1	chr11	62380795	62380808	CATCCGCCTGGCAC	-	frameshift_deletion	NA
ROS1	chr6	117681544	117681544	G	T	nonsynonymous	NA
RSRC2	chr12	123005966	123005966	C	T	nonsynonymous	NA
RTN4RL1	chr17	1840166	1840166	G	C	nonsynonymous	NA
SCARA5	chr8	27737230	27737230	C	T	nonsynonymous	NA
SCD5	chr4	83626480	83626480	G	C	nonsynonymous	NA
SCNM1;TNFAIP8L2-SCNM1	chr1	151141514	151141514	G	T	stopgain	NA
SH3PXD2A	chr10	105362656	105362656	C	A	nonsynonymous	NA
SLC22A24	chr11	62886363	62886363	G	T	nonsynonymous	NA
SLC28A2	chr15	45560516	45560516	C	A	nonsynonymous	NA
SLC6A14	chrX	115585510	115585510	C	G	nonsynonymous	NA
SLITRK4	chrX	142717429	142717434	GCTAAG	-	nonframeshift_deletion	NA
SPEG	chr2	220334043	220334043	C	G	nonsynonymous	NA
SPEG	chr2	220357387	220357387	G	A	nonsynonymous	NA
STIP1	chr11	63963107	63963148	TCAATACTAGGAAACTACAAG ATCCCCGGATCATGACCACTC	-	frameshift_deletion	NA
SUCO	chr1	172559029	172559030	TC	-	frameshift_deletion	NA
SVIL	chr10	29811351	29811351	G	C	nonsynonymous	NA
SWI5	chr9	131038615	131038615	C	T	nonsynonymous	NA
TACR1	chr2	75278440	75278440	G	C	nonsynonymous	NA
TAS2R50	chr12	11138637	11138637	C	G	nonsynonymous	NA
TCF7L2	chr10	114710693	114710693	T	A	nonsynonymous	NA
TECRL	chr4	65145841	65145841	T	A	nonsynonymous	NA
TENM2	chr5	167674849	167674849	A	G	nonsynonymous	NA
TMCO5A	chr15	38235597	38235597	G	A	nonsynonymous	NA
TMEM81	chr1	205052931	205052931	C	T	nonsynonymous	NA
TRIM22	chr11	5717588	5717588	G	T	nonsynonymous	NA
TRIM24	chr7	138255703	138255703	G	T	nonsynonymous	NA
TRIP11	chr14	92474162	92474162	T	C	nonsynonymous	NA
TSPAN6	chrX	99890202	99890208	GGCAGCG	-	frameshift_deletion	NA
TTYH1	chr19	54933391	54933391	G	A	nonsynonymous	NA
UBAP1	chr9	34250668	34250668	C	G	nonsynonymous	NA
UFL1	chr6	96971046	96971046	G	C	nonsynonymous	NA
UNC13C	chr15	54307858	54307858	G	A	nonsynonymous	NA
VPS33B	chr15	91543009	91543009	C	A	nonsynonymous	NA

XAB2	chr19	7685734	7685734	C	A	stopgain	NA
YWHAE	chr17	1264486	1264486	T	C	nonsynonymous	NA
ZBTB33	chrX	119387980	119387980	C	T	nonsynonymous	NA
ZDHHC15	chrX	74725656	74725656	-	T	frameshift_insertion	NA
ZFHX4	chr8	77616797	77616797	C	A	nonsynonymous	NA
ZNF106	chr15	42730828	42730828	C	A	stopgain	NA
ZNF226	chr19	44679765	44679765	C	G	nonsynonymous	NA
ZNF286A	chr17	15619842	15619842	G	C	nonsynonymous	NA
ZNF436	chr1	23689253	23689253	G	T	nonsynonymous	NA
ZNF479	chr7	57188741	57188741	T	-	frameshift_deletion	NA
ZNF480	chr19	52825015	52825015	C	G	nonsynonymous	NA
ZNF570	chr19	37966825	37966825	G	C	nonsynonymous	NA
ZNF600	chr19	53270014	53270014	C	T	nonsynonymous	NA
ZNF638	chr2	71592637	71592637	C	G	nonsynonymous	NA
ZNF770	chr15	35273865	35273865	T	A	nonsynonymous	NA
ZNF7	chr8	146068267	146068267	G	A	nonsynonymous	NA
ZNF823	chr19	11833315	11833315	C	G	nonsynonymous	NA
ACTN2	chr1	236881193	236881193	A	C	NA	nonsynonymous
ADAMTS18	chr16	77334184	77334184	C	G	NA	nonsynonymous
AIMP2	chr7	6049087	6049087	C	A	NA	nonsynonymous
AJUBA	chr14	23450954	23450955	CC	-	NA	frameshift_deletion
ALDH16A1	chr19	49967472	49967472	C	T	NA	nonsynonymous
ALDH1A3	chr15	101427862	101427862	G	C	NA	nonsynonymous
ANK3	chr10	61830900	61830900	T	G	NA	nonsynonymous
ANKRD13C	chr1	70779449	70779449	G	A	NA	nonsynonymous
AP4E1	chr15	51217313	51217313	C	G	NA	nonsynonymous
APOBEC3B	chr22	39382067	39382067	G	C	NA	nonsynonymous
ARHGEF10L	chr1	17914103	17914103	C	-	NA	frameshift_deletion
ARMCX5-GPRASP2;GPRASP2	chrX	101972146	101972146	G	A	NA	nonsynonymous
ASB10	chr7	150883666	150883666	G	A	NA	nonsynonymous
ASPM	chr1	197071008	197071008	C	T	NA	nonsynonymous
ATM	chr11	108218066	108218066	-	A	NA	frameshift_insertion
ATP6V0B	chr1	44442982	44442982	A	G	NA	nonsynonymous
BTN2A1	chr6	26468326	26468326	G	T	NA	nonsynonymous
CARD10	chr22	37888809	37888809	T	A	NA	nonsynonymous
CCSER1	chr4	91736958	91736958	G	A	NA	nonsynonymous
CDH12	chr5	21975260	21975260	T	A	NA	nonsynonymous
CEP135	chr4	56875993	56875993	G	C	NA	nonsynonymous
CES2	chr16	66973218	66973218	G	T	NA	nonsynonymous
CFHR4	chr1	196876137	196876137	G	C	NA	nonsynonymous
CHRM1	chr11	62678194	62678194	C	T	NA	nonsynonymous
CILP2	chr19	19655742	19655742	C	G	NA	nonsynonymous
CNST	chr1	246810713	246810713	C	G	NA	nonsynonymous
CTRC	chr1	15769005	15769005	G	A	NA	nonsynonymous
CUL7	chr6	43006406	43006406	-	CA	NA	frameshift_insertion
CYP17A1	chr10	104595015	104595015	C	A	NA	nonsynonymous
DCAF8L1	chrX	27998176	27998176	G	C	NA	nonsynonymous
DDR2	chr1	162748503	162748503	G	A	NA	nonsynonymous
DEXI	chr16	11035649	11035649	-	G	NA	frameshift_insertion
DHX34	chr19	47879212	47879212	C	T	NA	nonsynonymous
DIDO1	chr20	61512786	61512786	C	G	NA	nonsynonymous
DOHH	chr19	3491560	3491560	T	C	NA	nonsynonymous
DOHH	chr19	3492410	3492410	C	T	NA	nonsynonymous
DYNC1H1	chr14	102482236	102482236	C	T	NA	nonsynonymous
EEF1D	chr8	144671809	144671809	C	T	NA	nonsynonymous
EFEMP1	chr2	56104891	56104891	A	T	NA	stopgain
EFHC1	chr6	52329845	52329845	G	C	NA	nonsynonymous
ELAVL3	chr19	11569393	11569393	G	A	NA	nonsynonymous
EP300	chr22	41542794	41542794	T	A	NA	nonsynonymous
FAAH	chr1	46878832	46878832	G	T	NA	nonsynonymous
FAM135A	chr6	71233734	71233734	C	T	NA	nonsynonymous
FAM13B	chr5	137354198	137354198	G	T	NA	nonsynonymous
FAM193A	chr4	2664936	2664936	A	G	NA	nonsynonymous
FAM83G	chr17	18907093	18907093	G	A	NA	stopgain
FAN1	chr15	31229409	31229409	G	T	NA	stopgain
FARP2	chr2	242407780	242407780	G	A	NA	nonsynonymous
FASTKD1	chr2	170394637	170394637	G	A	NA	stopgain
FASTKD1	chr2	170417140	170417140	T	A	NA	nonsynonymous
FBN3	chr19	8155058	8155058	T	C	NA	nonsynonymous
FLNB	chr3	58111314	58111314	-	T	NA	frameshift_insertion
GHSR	chr3	172165995	172165995	C	A	NA	nonsynonymous
GNAL	chr18	11867195	11867195	G	A	NA	nonsynonymous
GNG12	chr1	68171233	68171233	-	A	NA	frameshift_insertion
GOPC	chr6	117923187	117923187	G	A	NA	stopgain
GPR146	chr7	1097840	1097840	C	T	NA	nonsynonymous

H6PD	chr1	9324222	9324222	C	T	NA	nonsynonymous
HHIP	chr4	145567964	145567964	A	C	NA	nonsynonymous
HIST2H2AB	chr1	149859411	149859411	-	A	NA	frameshift_insertion
HIVEP2	chr6	143093676	143093676	T	-	NA	frameshift_deletion
HMCN1	chr1	186026497	186026497	C	T	NA	nonsynonymous
HRG	chr3	186395268	186395268	C	G	NA	nonsynonymous
IL17RC	chr3	9974663	9974663	-	T	NA	frameshift_insertion
IQUB	chr7	123119966	123119966	T	A	NA	nonsynonymous
IRAK3	chr12	66641632	66641632	C	A	NA	nonsynonymous
KALRN	chr3	124369685	124369685	T	A	NA	nonsynonymous
KANK1	chr9	730156	730156	A	G	NA	nonsynonymous
KIAA1109	chr4	123170726	123170726	G	T	NA	nonsynonymous
KIF21A	chr12	39735859	39735859	T	C	NA	nonsynonymous
KIF23	chr15	69732770	69732770	C	T	NA	nonsynonymous
KIF5A	chr12	57969923	57969923	G	C	NA	nonsynonymous
KLHDC9	chr1	161068762	161068762	-	G	NA	frameshift_insertion
LHCGR	chr2	48936120	48936120	C	G	NA	nonsynonymous
LRP1B	chr2	141643707	141643707	C	A	NA	nonsynonymous
LRP6	chr12	12300306	12300306	G	C	NA	nonsynonymous
LRRIQ1	chr12	85449394	85449394	-	A	NA	frameshift_insertion
MAGEB10	chrX	27839647	27839647	C	G	NA	nonsynonymous
MAP3K1	chr5	56189399	56189399	G	T	NA	nonsynonymous
MED31	chr17	6547806	6547806	T	C	NA	nonsynonymous
MET	chr7	116411903	116411903	A	T	NA	nonsynonymous
MIOS	chr7	7645654	7645654	A	G	NA	nonsynonymous
MKI67	chr10	129906419	129906419	C	A	NA	stopgain
MPND	chr19	4357329	4357329	C	T	NA	nonsynonymous
MPND	chr19	4357339	4357339	C	G	NA	nonsynonymous
MUT	chr6	49421303	49421303	C	G	NA	nonsynonymous
MYBPC3	chr11	47371658	47371658	T	C	NA	nonsynonymous
NAV1	chr1	201750144	201750144	-	CA	NA	frameshift_insertion
NCKAP5	chr2	133541238	133541238	G	C	NA	nonsynonymous
NCOA3	chr20	46265315	46265315	C	A	NA	nonsynonymous
NCOA3	chr20	46268792	46268792	A	G	NA	nonsynonymous
NEXN	chr1	78407841	78407844	AGTT	-	NA	frameshift_deletion
NGFR	chr17	47590297	47590297	G	A	NA	nonsynonymous
NUP205	chr7	135261779	135261779	A	-	NA	frameshift_deletion
OR2L3	chr1	248224130	248224130	C	G	NA	nonsynonymous
OR9K2	chr12	55523613	55523613	T	A	NA	nonsynonymous
OS9	chr12	58089768	58089768	C	T	NA	nonsynonymous
PARP6	chr15	72534549	72534549	G	C	NA	nonsynonymous
PCDH17	chr13	58240955	58240955	C	T	NA	nonsynonymous
PCDHA6	chr5	140207992	140207992	C	A	NA	nonsynonymous
PCF11	chr11	82876649	82876649	C	G	NA	nonsynonymous
PCNX2	chr1	233397040	233397040	C	A	NA	nonsynonymous
PIEZO1	chr16	88807912	88807912	G	C	NA	nonsynonymous
PLCE1	chr10	96053358	96053358	C	A	NA	nonsynonymous
PSME2	chr14	24615757	24615757	C	T	NA	nonsynonymous
PTPN4	chr2	120635049	120635049	C	T	NA	nonsynonymous
RSPO1	chr1	38082222	38082222	C	T	NA	nonsynonymous
RYR2	chr1	237969486	237969486	A	G	NA	nonsynonymous
SBNO2	chr19	1112059	1112059	A	T	NA	nonsynonymous
SERPINB3	chr18	61323103	61323103	G	A	NA	nonsynonymous
SLCO2A1	chr3	133667535	133667535	-	A	NA	frameshift_insertion
SMNDC1	chr10	112053950	112053950	T	G	NA	nonsynonymous
SP100	chr2	231326126	231326126	A	G	NA	nonsynonymous
SUGP2	chr19	19136044	19136070	ACATTTGAATGAGGCAAGCATTTTAGC	-	NA	nonframeshift_deletion
SV2A	chr1	149885239	149885239	C	T	NA	nonsynonymous
SYDE2	chr1	85648361	85648361	T	A	NA	nonsynonymous
SYPL2	chr1	110022109	110022109	-	CCAGGG	NA	nonframeshift_insertion
SYTL2	chr11	85445546	85445546	G	A	NA	nonsynonymous
TBC1D8	chr2	101650167	101650167	C	G	NA	nonsynonymous
TCERG1L	chr10	132932663	132932663	T	C	NA	nonsynonymous
TESK2	chr1	45811014	45811014	C	A	NA	nonsynonymous
TMEM182	chr2	103414400	103414400	C	A	NA	nonsynonymous
TMEM98	chr17	31263382	31263382	-	A	NA	frameshift_insertion
TRIM36	chr5	114469652	114469652	T	C	NA	nonsynonymous
TRIM59	chr3	160156926	160156926	T	C	NA	nonsynonymous
TRIM64C	chr11	49080471	49080471	T	A	NA	nonsynonymous
TRPA1	chr8	72950289	72950289	T	-	NA	frameshift_deletion
TRPS1	chr8	116426528	116426528	T	C	NA	nonsynonymous
TTC26	chr7	138849958	138849958	G	C	NA	nonsynonymous
TUBB4A	chr19	6501331	6501331	C	T	NA	nonsynonymous
TUBGCP4	chr15	43693974	43693974	T	A	NA	nonsynonymous

USH2A	chr1	216251488	216251488	C	T	NA	nonsynonymous
VWDE	chr7	12376860	12376860	T	C	NA	nonsynonymous
WASHC4	chr12	105557906	105557906	A	T	NA	stopgain
WDFY3	chr4	85642602	85642602	T	A	NA	nonsynonymous
WDR19	chr4	39196244	39196244	A	G	NA	nonsynonymous
ZC3H12B	chrX	64719797	64719797	G	A	NA	nonsynonymous
ZC3H7A	chr16	11845241	11845241	C	G	NA	nonsynonymous
ZCCHC8	chr12	122977330	122977330	T	C	NA	nonsynonymous
ZFC3H1	chr12	72023391	72023391	T	C	NA	nonsynonymous
ZFHX2	chr14	24003212	24003212	C	T	NA	nonsynonymous
ZMPSTE24	chr1	40751612	40751612	T	C	NA	nonsynonymous
ZNF112	chr19	44833592	44833592	G	C	NA	nonsynonymous
ZNF335	chr20	44578160	44578160	-	TG	NA	frameshift_insertion
ZNF365	chr10	64415152	64415152	T	A	NA	nonsynonymous
ZNF789	chr7	99077401	99077401	G	A	NA	nonsynonymous

Gene	Chromosome	Start	End	Ref	Alt	SH2-E	SH2-H
OPTN	chr10	13152465	13152465	A	G	nonsynonymous	nonsynonymous
RNF121	chr11	71668279	71668279	A	G	nonsynonymous	nonsynonymous
SEC23A	chr14	39531020	39531020	T	C	nonsynonymous	nonsynonymous
TP53	chr17	7574021	7574021	C	A	stopgain	stopgain
ADRA2C	chr4	3768437	3768437	C	T	nonsynonymous	NA
AK9	chr6	109871385	109871385	G	A	stopgain	NA
BCO2	chr11	112084537	112084537	C	T	nonsynonymous	NA
C2CD5	chr12	22610023	22610023	T	C	nonsynonymous	NA
C2orf73	chr2	54570981	54570981	G	A	nonsynonymous	NA
CCDC181	chr1	169390720	169390720	A	C	nonsynonymous	NA
CDHR2	chr5	175992391	175992391	C	T	nonsynonymous	NA
CDKN2A	chr9	21974708	21974737	GCGTTGGGCAGCGCCCCCGCC TCCAGCAGC	-	nonframeshift_deletion	NA
CEP83	chr12	94794721	94794721	G	A	nonsynonymous	NA
CLK3	chr15	74919741	74919741	T	G	nonsynonymous	NA
ELOVL1	chr1	43830263	43830263	T	C	nonsynonymous	NA
EPHA1	chr7	143088841	143088841	A	T	nonsynonymous	NA
ERAP1	chr5	96119660	96119660	T	C	nonsynonymous	NA
FADS3	chr11	61646049	61646049	T	C	nonsynonymous	NA
FAT2	chr5	150923333	150923338	AAATTG	-	nonframeshift_deletion	NA
FBXL8	chr16	67197388	67197388	G	T	nonsynonymous	NA
FBXW7	chr4	153247226	153247226	A	G	nonsynonymous	NA
FCRL1	chr1	157773891	157773891	C	A	nonsynonymous	NA
GPSM1	chr9	139244094	139244094	C	T	nonsynonymous	NA
GRM6	chr5	178418456	178418456	T	G	nonsynonymous	NA
HHIP	chr4	145579946	145579946	C	A	nonsynonymous	NA
HNRNPCL1;HNRNPCL3;HNRNPCL4	chr1	12907418	12907418	C	G	nonsynonymous	NA
IDE	chr10	94214227	94214227	T	C	nonsynonymous	NA
IFT122	chr3	129225368	129225368	-	T	frameshift_insertion	NA
IGSF10	chr3	151163586	151163586	G	C	nonsynonymous	NA
ITGA4	chr2	182347148	182347148	A	T	nonsynonymous	NA
KCNH8	chr3	19554627	19554627	C	A	nonsynonymous	NA
KMT2C	chr7	151875048	151875048	C	A	nonsynonymous	NA
KNTC1	chr12	123075930	123075930	T	C	nonsynonymous	NA
KPNA4	chr3	160225913	160225913	G	C	nonsynonymous	NA
LMF2	chr22	50943110	50943110	G	C	nonsynonymous	NA
LMF2	chr22	50943916	50943916	C	T	nonsynonymous	NA
LPAR6	chr13	48985815	48985815	G	A	nonsynonymous	NA
MAN2A2	chr15	91461431	91461431	A	G	nonsynonymous	NA
METTL17	chr14	21464420	21464420	-	A	frameshift_insertion	NA
MSH3	chr5	80109480	80109480	G	C	nonsynonymous	NA
MTUS2	chr13	30054448	30054448	C	A	nonsynonymous	NA
MYH15	chr3	108229353	108229353	C	T	nonsynonymous	NA
MYO5B	chr18	47432937	47432937	G	C	nonsynonymous	NA
NUDT5	chr10	12228238	12228248	ATGATATACTG	-	frameshift_deletion	NA
NUDT5	chr10	12228250	12228250	T	C	nonsynonymous	NA
NYNRIN	chr14	24879218	24879218	C	A	nonsynonymous	NA
OR13A1	chr10	45799827	45799827	C	T	nonsynonymous	NA
OTUD4	chr4	146076780	146076780	G	A	nonsynonymous	NA
P2RY4	chrX	69479065	69479065	-	T	frameshift_insertion	NA
PCDH15	chr10	55587258	55587258	G	A	nonsynonymous	NA
PCDHA11	chr5	140249947	140249947	A	C	nonsynonymous	NA
PCDHB8	chr5	140558171	140558171	C	T	nonsynonymous	NA
PCNT	chr21	47860819	47860819	C	G	nonsynonymous	NA
PHOSPHO1	chr17	47301655	47301655	C	T	nonsynonymous	NA
PIK3CA	chr3	178952085	178952085	A	G	nonsynonymous	NA
PXDNL	chr8	52321351	52321351	C	T	nonsynonymous	NA
RUNX1T1	chr8	93027007	93027007	C	T	nonsynonymous	NA
SAMD14	chr17	48191418	48191418	A	G	nonsynonymous	NA
SIN3B	chr19	16952721	16952721	G	T	nonsynonymous	NA
SPERT	chr13	46288415	46288415	G	A	nonsynonymous	NA
SSPO	chr7	149484770	149484770	G	A	nonsynonymous	NA
TBC1D9B	chr5	179315305	179315305	A	G	nonsynonymous	NA
TET2	chr4	106182991	106182991	G	A	nonsynonymous	NA
TTC6	chr14	38256752	38256752	G	T	nonsynonymous	NA
UBA3	chr3	69105209	69105209	A	G	nonsynonymous	NA
UTP14A	chrX	129045091	129045091	G	T	nonsynonymous	NA
ZBTB17	chr1	16268467	16268467	-	T	frameshift_insertion	NA
ZDHHC17	chr12	77243171	77243171	A	G	nonsynonymous	NA
ZMYM2	chr13	20660134	20660134	C	G	nonsynonymous	NA
ZNF202	chr11	123600422	123600422	C	T	nonsynonymous	NA
ZNF433	chr19	12126096	12126096	G	A	nonsynonymous	NA
ZNF687	chr1	151259132	151259132	G	A	nonsynonymous	NA
ZNFX1	chr20	47865426	47865426	A	G	nonsynonymous	NA
ZWINT	chr10	58118406	58118406	G	A	nonsynonymous	NA

AKT1	chr14	105241340	105241411	CCTGTAAAGCAGGGCTGGGTGAGCTGCCACCCCGCACCCTCATCTCCACCCTGCCCCACCGCCCCGGCCCCA	-	NA	frameshift_deletion
CAMK2D	chr4	114386713	114386713	T	C	NA	nonsynonymous
CDC73	chr1	193218884	193218884	A	T	NA	nonsynonymous
FAT1	chr4	187540155	187540155	A	-	NA	frameshift_deletion
GRM3	chr7	86415811	86415811	C	T	NA	nonsynonymous
NCAM2	chr21	22804470	22804470	C	T	NA	nonsynonymous
SELE	chr1	169699704	169699704	T	C	NA	nonsynonymous
TSPAN17	chr5	176078761	176078761	C	T	NA	nonsynonymous
XYLB	chr3	38454497	38454497	C	T	NA	nonsynonymous

Gene	chromosome	Start	Stop	Ref	Alt	SH3-E	SH3-H
MYO1H	chr12	109882319	109882319	A	G	nonsynonymous	nonsynonymous
ACCS	chr11	44101082	44101082	C	T	nonsynonymous	NA
ACE2	chrX	15613012	15613012	G	A	stopgain	NA
ACOT2	chr14	74041894	74041894	G	C	nonsynonymous	NA
ACOX1	chr17	73956405	73956405	G	C	nonsynonymous	NA
ADAM17	chr2	9683362	9683362	G	C	nonsynonymous	NA
ADAMTS9	chr3	64608202	64608202	T	C	nonsynonymous	NA
AEBP2	chr12	19646822	19646822	C	G	stopgain	NA
AGBL5	chr2	27282216	27282216	C	T	nonsynonymous	NA
ALMS1	chr2	73784379	73784379	G	C	nonsynonymous	NA
ALX4	chr11	44331467	44331467	G	A	nonsynonymous	NA
AMPD3	chr11	10477934	10477934	A	G	nonsynonymous	NA
ANGEL1	chr14	77255678	77255678	G	C	nonsynonymous	NA
ANKS1B	chr12	100169386	100169386	C	T	nonsynonymous	NA
APEX2	chrX	55033852	55033852	G	T	nonsynonymous	NA
APOBR	chr16	28508310	28508310	G	A	nonsynonymous	NA
ARHGAP20	chr11	110451899	110451899	C	G	nonsynonymous	NA
ARHGAP22	chr10	49658786	49658786	C	T	nonsynonymous	NA
ASCL1	chr12	103352401	103352401	G	A	nonsynonymous	NA
BANF2	chr20	17705670	17705670	G	C	nonsynonymous	NA
BBS1	chr11	66282147	66282147	G	C	nonsynonymous	NA
BCL2L15	chr1	114429252	114429252	G	C	nonsynonymous	NA
BCO2	chr11	112064399	112064399	G	C	nonsynonymous	NA
C10orf71	chr10	50532160	50532160	G	C	nonsynonymous	NA
C19orf44	chr19	16620571	16620571	G	T	nonsynonymous	NA
C4orf19	chr4	37591756	37591756	G	A	nonsynonymous	NA
C5orf38	chr5	2752824	2752824	G	T	nonsynonymous	NA
CA11	chr19	49143359	49143359	G	C	nonsynonymous	NA
CACNA1A	chr19	13409455	13409455	C	T	nonsynonymous	NA
CACNA2D4	chr12	1919717	1919717	C	G	nonsynonymous	NA
CACNB2	chr10	18828444	18828444	G	C	nonsynonymous	NA
CANX	chr5	179136920	179136920	A	T	nonsynonymous	NA
CASP8AP2	chr6	90578530	90578530	G	C	nonsynonymous	NA
CDC45	chr22	19504359	19504359	G	C	nonsynonymous	NA
CENPN	chr16	81061821	81061821	G	A	nonsynonymous	NA
CENPT	chr16	67865128	67865128	C	T	nonsynonymous	NA
CFAP54	chr12	97017543	97017557	AGGATTCTTCTAAGA	-	frameshift_deletion	NA
CNOT8	chr5	154242878	154242878	G	C	nonsynonymous	NA
CNTN1	chr12	41312458	41312458	G	T	stopgain	NA
COBL	chr7	51096059	51096059	C	G	nonsynonymous	NA
COL14A1	chr8	121295933	121295933	C	T	nonsynonymous	NA
CPD	chr17	28778834	28778834	C	T	nonsynonymous	NA
CPNE4	chr3	131415451	131415451	C	T	nonsynonymous	NA
CSNK1A1	chr5	148889459	148889459	C	T	nonsynonymous	NA
CTNNB1	chr3	41275235	41275237	TCT	-	nonframeshift_deletion	NA
CUX1	chr7	101559413	101559413	G	A	nonsynonymous	NA
CYP4F11	chr19	16024597	16024597	C	T	nonsynonymous	NA
DCAF13	chr8	104427646	104427646	C	T	nonsynonymous	NA
DCHS2	chr4	155158252	155158252	G	A	nonsynonymous	NA
DIAPH3	chr13	60545138	60545138	G	A	nonsynonymous	NA
DIRAS1	chr19	2717405	2717405	C	G	nonsynonymous	NA
DMWD	chr19	46289538	46289538	C	T	nonsynonymous	NA
DNM1L	chr12	32866205	32866205	C	A	nonsynonymous	NA
DPP4	chr2	162875355	162875355	T	A	nonsynonymous	NA
ECM2	chr9	95277053	95277053	G	A	nonsynonymous	NA
EHD4	chr15	42193097	42193097	G	C	nonsynonymous	NA
ENO1	chr1	8934958	8934958	G	A	nonsynonymous	NA
EP300	chr22	41513494	41513494	C	T	nonsynonymous	NA
EPG5	chr18	43526654	43526654	G	A	nonsynonymous	NA
EPHA5	chr4	66467948	66467948	C	G	nonsynonymous	NA
EPRS	chr1	220157570	220157570	T	C	nonsynonymous	NA
ERMN	chr2	158181200	158181200	C	T	nonsynonymous	NA
FAM208B	chr10	5772875	5772875	C	G	nonsynonymous	NA
FAT3	chr11	92088117	92088117	G	A	nonsynonymous	NA
FBRS	chr16	30680436	30680451	CCCAAGGCCCGGGCTG	-	frameshift_deletion	NA
FBXO45	chr3	196304613	196304613	A	G	nonsynonymous	NA
FBXO47	chr17	37118184	37118184	C	G	nonsynonymous	NA
FGFR1	chr8	38271704	38271704	G	A	nonsynonymous	NA
FLCN	chr17	17131361	17131361	G	C	nonsynonymous	NA
FSTL5	chr4	162421168	162421168	C	A	nonsynonymous	NA
FTSJ3	chr17	61898859	61898859	C	A	stopgain	NA
GANC	chr15	42600429	42600429	C	G	nonsynonymous	NA
GFPT2	chr5	179740851	179740851	C	T	nonsynonymous	NA
GIPC1	chr19	14593566	14593566	C	G	nonsynonymous	NA
GIT2	chr12	110389058	110389058	C	T	nonsynonymous	NA
GLS2	chr12	56865335	56865335	G	C	nonsynonymous	NA
GPBP1L1	chr1	46099830	46099830	G	C	nonsynonymous	NA
GRID1	chr10	87373336	87373336	C	T	nonsynonymous	NA
GRIN3B	chr19	1005546	1005546	C	A	nonsynonymous	NA
GTPBP3	chr19	17448907	17448907	C	G	nonsynonymous	NA

HDGFL2	chr19	4491674	4491674	G	A	nonsynonymous	NA
HIPK3	chr11	33308906	33308906	C	G	nonsynonymous	NA
HIST1H4F	chr6	26240762	26240762	C	T	nonsynonymous	NA
HSP90B1	chr12	104332182	104332182	A	T	nonsynonymous	NA
HTR7	chr10	92616923	92616923	G	C	nonsynonymous	NA
IL7R	chr5	35876403	35876403	C	G	nonsynonymous	NA
IPO7	chr11	9441979	9441979	G	C	nonsynonymous	NA
IRF6	chr1	209964161	209964161	G	T	nonsynonymous	NA
ITIH2	chr10	7786167	7786167	G	C	nonsynonymous	NA
ITLN2	chr1	160924228	160924228	T	-	frameshift_deletion	NA
ITSN1	chr21	35186316	35186316	G	C	nonsynonymous	NA
IVL	chr1	152882554	152882554	A	G	nonsynonymous	NA
IVNS1ABP	chr1	185276656	185276656	C	A	stopgain	NA
KBTBD11	chr8	1950413	1950413	G	A	nonsynonymous	NA
KCNA10	chr1	111061340	111061340	C	T	nonsynonymous	NA
KIAA1210	chrX	118223136	118223136	G	A	nonsynonymous	NA
KIR3DL1	chr19	55331299	55331299	G	A	nonsynonymous	NA
KLHL32	chr6	97562253	97562253	G	T	stopgain	NA
KPNA4	chr3	160249326	160249326	G	C	nonsynonymous	NA
KRI1	chr19	10670070	10670070	C	G	nonsynonymous	NA
KRIT1	chr7	91852175	91852175	G	C	nonsynonymous	NA
KRTAP11-1	chr21	32253792	32253792	G	A	nonsynonymous	NA
LACTB	chr15	63433642	63433642	G	A	nonsynonymous	NA
LMAN2L	chr2	97377708	97377708	G	A	nonsynonymous	NA
MCMBP	chr10	121608963	121608963	C	T	nonsynonymous	NA
MEGF8	chr19	42830463	42830463	C	T	nonsynonymous	NA
MOV10	chr1	113234414	113234414	G	C	nonsynonymous	NA
MRPL1	chr4	78792940	78792940	A	G	nonsynonymous	NA
MYH14	chr19	50783370	50783370	G	A	nonsynonymous	NA
NAMPT	chr7	105903878	105903878	A	T	nonsynonymous	NA
NPTX2	chr7	98256566	98256566	C	G	nonsynonymous	NA
NUF2	chr1	163310166	163310166	G	C	nonsynonymous	NA
NXN	chr17	726946	726946	T	C	nonsynonymous	NA
OR1Q1	chr9	125377564	125377564	C	G	nonsynonymous	NA
OR2L3	chr1	248224365	248224365	C	T	nonsynonymous	NA
OR4S1	chr11	48328541	48328541	A	G	nonsynonymous	NA
OTOGL	chr12	80696506	80696506	C	G	nonsynonymous	NA
PAX4	chr7	127253526	127253526	G	T	nonsynonymous	NA
PCDHA9	chr5	140230217	140230217	C	G	nonsynonymous	NA
PCLO	chr7	82784776	82784776	G	T	nonsynonymous	NA
PDCD1	chr2	242794502	242794502	C	G	nonsynonymous	NA
PGLYRP2	chr19	15586406	15586406	C	T	nonsynonymous	NA
PHYKPL	chr5	177649516	177649516	C	T	nonsynonymous	NA
PIF1	chr15	65116218	65116218	G	A	nonsynonymous	NA
PITPNM1	chr11	67262399	67262399	G	A	nonsynonymous	NA
PJA2	chr5	108704293	108704293	C	T	nonsynonymous	NA
PLCB3	chr11	64026135	64026135	C	A	nonsynonymous	NA
PLCB3	chr11	64031029	64031029	C	G	nonsynonymous	NA
PLCB3	chr11	64031055	64031055	C	G	nonsynonymous	NA
PLEC	chr8	145002081	145002081	C	A	stopgain	NA
PLEC	chr8	145003431	145003431	C	G	nonsynonymous	NA
PLEC	chr8	145008204	145008204	C	T	nonsynonymous	NA
PLEKHA5	chr12	19522699	19522699	G	T	nonsynonymous	NA
PLK4	chr4	128806907	128806907	C	T	nonsynonymous	NA
PLXNA1	chr3	126749127	126749127	C	G	nonsynonymous	NA
PLXND1	chr3	129303062	129303062	C	G	nonsynonymous	NA
PRCC	chr1	156737685	156737685	C	G	nonsynonymous	NA
PRCC	chr1	156737729	156737729	C	T	stopgain	NA
PRUNE2	chr9	79320399	79320399	C	G	nonsynonymous	NA
PSD2	chr5	139193879	139193879	C	T	nonsynonymous	NA
PTER	chr10	16553208	16553208	G	C	nonsynonymous	NA
PTPN3	chr9	112182741	112182741	C	T	nonsynonymous	NA
PTPRB	chr12	71016286	71016286	C	T	nonsynonymous	NA
PYCR2	chr1	226111768	226111768	G	C	nonsynonymous	NA
PYM1	chr12	56295701	56295701	C	A	nonsynonymous	NA
RAB1A	chr2	65315689	65315689	C	G	nonsynonymous	NA
RAB3GAP2	chr1	220345314	220345314	C	G	nonsynonymous	NA
RGS11	chr16	321398	321398	G	A	nonsynonymous	NA
RHOB	chr2	20647365	20647365	G	C	nonsynonymous	NA
RIF1	chr2	152271359	152271359	C	T	nonsynonymous	NA
RNF10	chr12	120998568	120998568	C	G	nonsynonymous	NA
RNF113A	chrX	119004693	119004693	C	T	nonsynonymous	NA
RPGRIP1L	chr16	53720345	53720345	C	G	nonsynonymous	NA
RPTN	chr1	152127240	152127240	C	T	nonsynonymous	NA
RPUSD1	chr16	837134	837134	T	C	nonsynonymous	NA
SDHA	chr5	235272	235272	G	C	nonsynonymous	NA
SETD2	chr3	47129644	47129644	C	G	nonsynonymous	NA
SF3B3	chr16	70603939	70603939	G	T	stopgain	NA
SH3RF2	chr5	145393569	145393569	C	G	nonsynonymous	NA
SH3TC2	chr5	148407735	148407735	C	G	nonsynonymous	NA
SLC12A6	chr15	34530503	34530503	A	T	nonsynonymous	NA
SLC2A3	chr12	8083226	8083226	C	G	nonsynonymous	NA
SLC39A4	chr8	145638738	145638738	C	T	nonsynonymous	NA

SLC9C2	chr1	173494052	173494052	C	T	nonsynonymous	NA
SMC1A	chrX	53436187	53436187	C	T	nonsynonymous	NA
SMC1A	chrX	53439110	53439110	C	A	nonsynonymous	NA
SNTG2	chr2	1133487	1133487	G	A	nonsynonymous	NA
SPATA17	chr1	217856674	217856674	A	C	nonsynonymous	NA
SREBF2	chr22	42264779	42264779	C	G	nonsynonymous	NA
ST3GAL5	chr2	86075029	86075029	C	T	nonsynonymous	NA
STAT1	chr2	191848438	191848438	G	C	nonsynonymous	NA
STK11	chr19	1221270	1221270	G	C	nonsynonymous	NA
SYNE1	chr6	152501285	152501285	C	G	nonsynonymous	NA
SYNE1	chr6	152501351	152501351	C	G	nonsynonymous	NA
SYNE1	chr6	152737865	152737865	C	G	nonsynonymous	NA
TAAR5	chr6	132910460	132910460	G	C	nonsynonymous	NA
TBC1D2	chr9	100971155	100971155	G	C	nonsynonymous	NA
TBCEL	chr11	120930733	120930733	G	C	nonsynonymous	NA
TCF4	chr18	53131350	53131350	C	A	nonsynonymous	NA
TDRD15	chr2	21360771	21360771	C	G	nonsynonymous	NA
TDRD5	chr1	179623495	179623495	G	C	nonsynonymous	NA
THOC3	chr5	175395153	175395153	G	C	nonsynonymous	NA
THSD1	chr13	52952104	52952104	C	G	nonsynonymous	NA
TMC1	chr9	75407237	75407237	G	C	nonsynonymous	NA
TMEM132B	chr12	126138199	126138199	T	C	nonsynonymous	NA
TMEM246	chr9	104239222	104239222	G	C	nonsynonymous	NA
TP53	chr17	7577094	7577094	G	A	nonsynonymous	NA
TRAP1	chr16	3712953	3712953	G	C	stopgain	NA
TRAPPC8	chr18	29451051	29451051	C	G	nonsynonymous	NA
TRAT1	chr3	108572551	108572551	C	T	nonsynonymous	NA
TRIM2	chr4	154215492	154215492	C	G	nonsynonymous	NA
TRIM58	chr1	248039555	248039555	C	G	nonsynonymous	NA
TRPM1	chr15	31294128	31294128	G	C	nonsynonymous	NA
TRPM1	chr15	31320677	31320677	G	T	nonsynonymous	NA
TRRAP	chr7	98527741	98527741	A	G	nonsynonymous	NA
TSKS	chr19	50243401	50243401	C	T	nonsynonymous	NA
TSNARE1	chr8	143425356	143425356	G	C	nonsynonymous	NA
TSNAXIP1	chr16	67857557	67857557	T	C	nonsynonymous	NA
TTLL4	chr2	219617852	219617852	C	T	nonsynonymous	NA
TTN	chr2	179426326	179426326	G	T	nonsynonymous	NA
TTN	chr2	179441742	179441742	C	A	nonsynonymous	NA
UBAP2	chr9	33926989	33926989	G	A	nonsynonymous	NA
UQCRC2	chr16	21974083	21974083	G	A	nonsynonymous	NA
USH2A	chr1	216061960	216061960	C	G	nonsynonymous	NA
VPREB1	chr22	22599567	22599567	C	G	nonsynonymous	NA
VPS13C	chr15	62283886	62283886	G	C	stopgain	NA
XRN1	chr3	142089350	142089350	C	G	nonsynonymous	NA
YTHDC2	chr5	112888913	112888913	C	T	nonsynonymous	NA
ZDBF2	chr2	207172934	207172934	G	C	nonsynonymous	NA
ZIM2	chr19	57286090	57286090	G	A	nonsynonymous	NA
ZKSCAN2	chr16	25255532	25255532	C	A	stopgain	NA
ZNF343	chr20	2474513	2474513	C	G	nonsynonymous	NA
ZNF415	chr19	53612472	53612472	C	T	nonsynonymous	NA
ZNF469	chr16	88502811	88502811	G	T	nonsynonymous	NA
ZNF486	chr19	20296860	20296860	G	A	nonsynonymous	NA
ZNF597	chr16	3486840	3486840	C	G	nonsynonymous	NA
ZNF680	chr7	63986784	63986784	G	T	nonsynonymous	NA
ZNF790	chr19	37310131	37310131	C	G	nonsynonymous	NA
ZNF790	chr19	37310411	37310411	C	T	nonsynonymous	NA
ACSBG2	chr19	6187658	6187658	G	A	NA	nonsynonymous
ADRA1D	chr20	4229094	4229094	C	T	NA	nonsynonymous
ANO5	chr11	22261164	22261164	A	G	NA	nonsynonymous
ANO5	chr11	22296196	22296196	A	T	NA	nonsynonymous
B3GAT1	chr11	134253637	134253637	G	T	NA	nonsynonymous
C8orf37	chr8	96281387	96281387	C	A	NA	stopgain
CFAP77	chr9	135357713	135357713	C	T	NA	nonsynonymous
CLSTN2	chr3	140178466	140178466	C	A	NA	nonsynonymous
CLVS2	chr6	123384853	123384853	C	T	NA	nonsynonymous
CNBD1	chr8	87951885	87951885	G	A	NA	nonsynonymous
CPO	chr2	207823082	207823082	G	A	NA	nonsynonymous
CSMD2	chr1	34011753	34011753	A	C	NA	nonsynonymous
CSPP1	chr8	68107672	68107672	C	G	NA	nonsynonymous
CTSK	chr1	150776499	150776499	G	C	NA	nonsynonymous
CYP19A1	chr15	51514653	51514653	C	T	NA	nonsynonymous
DAAM2	chr6	39851767	39851767	G	T	NA	nonsynonymous
DGKB	chr7	14647132	14647132	T	C	NA	nonsynonymous
DNAJC13	chr3	132166257	132166257	G	C	NA	nonsynonymous
ERI3	chr1	44778855	44778855	G	T	NA	nonsynonymous
GPRASP1	chrX	101910739	101910739	C	G	NA	nonsynonymous
GRXCR1	chr4	42895652	42895652	G	T	NA	nonsynonymous
HLTF	chr3	148757915	148757915	T	A	NA	nonsynonymous
ITPR2	chr12	26731655	26731655	C	A	NA	nonsynonymous
IWS1	chr2	128246757	128246757	C	G	NA	nonsynonymous
KLRC2	chr12	10588512	10588512	T	G	NA	nonsynonymous
LDLRAP1	chr1	25893415	25893415	C	T	NA	nonsynonymous
LMTK3	chr19	49013310	49013310	G	C	NA	nonsynonymous

METTL25	chr12	82872742	82872742	T	G	NA	nonsynonymous
MMP2	chr16	55530960	55530960	C	T	NA	nonsynonymous
MMP9	chr20	44637617	44637617	T	C	NA	nonsynonymous
MNT	chr17	2290817	2290817	G	A	NA	nonsynonymous
MTMR8	chrX	63490886	63490886	G	C	NA	nonsynonymous
MZT2A	chr2	132241788	132241788	C	T	NA	nonsynonymous
NUP155	chr5	37310665	37310665	T	C	NA	nonsynonymous
OPRPN	chr4	71275670	71275670	G	A	NA	nonsynonymous
OR2T6	chr1	248551086	248551086	G	T	NA	nonsynonymous
OSBPL6	chr2	179247895	179247895	C	T	NA	nonsynonymous
PAQR8	chr6	52268504	52268507	TACA	-	NA	frameshift_deletion
PCDHGA2	chr5	140720952	140720952	C	T	NA	nonsynonymous
PCDHGA9	chr5	140783670	140783670	G	C	NA	nonsynonymous
PCDHGB7	chr5	140798033	140798033	G	T	NA	stopgain
PPP4R3A	chr14	91939445	91939445	T	C	NA	nonsynonymous
PTGER3	chr1	71512517	71512517	G	T	NA	stopgain
RBBP8NL	chr20	60989041	60989041	G	T	NA	nonsynonymous
SERPINE2	chr2	224866605	224866605	G	A	NA	nonsynonymous
SERPINI2	chr3	167185060	167185060	A	-	NA	frameshift_deletion
SERPINI2	chr3	167185065	167185065	A	T	NA	nonsynonymous
TAS2R46	chr12	11214548	11214548	G	A	NA	nonsynonymous
TIFA	chr4	113199142	113199142	G	A	NA	nonsynonymous
TMA16	chr4	164428277	164428277	C	G	NA	nonsynonymous
TMEM132C	chr12	129189840	129189840	C	G	NA	nonsynonymous
TNC	chr9	117826099	117826099	G	A	NA	nonsynonymous
TSPOAP1	chr17	56382906	56382906	C	A	NA	nonsynonymous
ULK1	chr12	132401012	132401012	G	C	NA	nonsynonymous
VPS13B	chr8	100026070	100026070	C	G	NA	nonsynonymous
ZMYM4	chr1	35846959	35846959	-	A	NA	frameshift_insertion
ZNF268	chr12	133779554	133779554	A	G	NA	nonsynonymous
ZNF430	chr19	21239568	21239568	G	T	NA	nonsynonymous
ZNF585A	chr19	37642728	37642728	C	A	NA	nonsynonymous
ZNF681	chr19	23927939	23927939	T	C	NA	nonsynonymous
ZSWIM6	chr5	60825902	60825902	T	A	NA	nonsynonymous

Gene	chromosome	Start	Stop	Ref	Alt	HK1-EA	HK1-EB	HK1-EC	HK1-HA	HK1-HC
ABCF3	chr3	183905767	183905767	G	A	nonsynonymous	NA	NA	NA	NA
ACTBL2	chr5	56778459	56778459	C	T	nonsynonymous	nonsynonymous	nonsynonymous	NA	NA
AMPD1	chr1	115215809	115215809	G	A	nonsynonymous	nonsynonymous	nonsynonymous	NA	NA
ANKRD50	chr4	125592411	125592411	T	A	nonsynonymous	nonsynonymous	nonsynonymous	NA	NA
AP1G1	chr16	71795419	71795419	T	C	nonsynonymous	nonsynonymous	nonsynonymous	NA	NA
ARHGEF18	chr19	7504933	7504933	A	G	nonsynonymous	NA	NA	NA	NA
B3GAT2	chr6	71603973	71603973	C	T	nonsynonymous	nonsynonymous	nonsynonymous	NA	NA
BAZ2B	chr2	160189181	160189181	C	T	nonsynonymous	nonsynonymous	nonsynonymous	NA	NA
BICD1	chr12	32480512	32480512	G	A	nonsynonymous	nonsynonymous	nonsynonymous	NA	NA
C1orf56	chr1	151021014	151021014	G	C	nonsynonymous	nonsynonymous	nonsynonymous	NA	NA
C22orf15	chr22	24107049	24107049	G	A	nonsynonymous	nonsynonymous	nonsynonymous	NA	NA
C3	chr19	6713289	6713289	T	C	nonsynonymous	nonsynonymous	nonsynonymous	NA	NA
CACNA1A	chr19	13563790	13563790	C	T	nonsynonymous	nonsynonymous	nonsynonymous	NA	NA
CCDC42	chr17	8638796	8638796	T	A	nonsynonymous	NA	nonsynonymous	NA	NA
CELF3	chr1	151688403	151688403	G	A	nonsynonymous	nonsynonymous	nonsynonymous	NA	NA
CGNL1	chr15	57730591	57730591	C	T	nonsynonymous	nonsynonymous	nonsynonymous	NA	NA
COCH	chr14	31355303	31355303	T	C	nonsynonymous	nonsynonymous	nonsynonymous	NA	NA
COL22A1	chr8	139601675	139601675	C	T	nonsynonymous	nonsynonymous	nonsynonymous	NA	NA
COL2A1	chr12	48374709	48374709	C	A	nonsynonymous	NA	NA	NA	NA
COL4A1	chr13	110817266	110817266	C	G	nonsynonymous	nonsynonymous	nonsynonymous	NA	NA
CPNE6	chr14	24546433	24546433	T	C	nonsynonymous	NA	NA	NA	NA
CTSB	chr8	11702657	11702657	C	T	nonsynonymous	NA	NA	NA	NA
CTSB	chr8	11702710	11702710	C	G	nonsynonymous	NA	NA	NA	NA
CXorf66	chrX	139038108	139038108	T	G	nonsynonymous	NA	NA	NA	NA
DDX50	chr10	70666680	70666680	A	G	nonsynonymous	nonsynonymous	nonsynonymous	NA	NA
DNAAF4	chr15	55759311	55759311	G	A	nonsynonymous	nonsynonymous	nonsynonymous	NA	NA
DNAH14	chr1	225521194	225521194	G	A	nonsynonymous	nonsynonymous	nonsynonymous	NA	NA
DNAH5	chr5	13810302	13810302	G	A	nonsynonymous	nonsynonymous	nonsynonymous	NA	NA
DPP4	chr2	162877158	162877158	C	T	nonsynonymous	NA	NA	NA	NA
DYNC1H1	chr14	102452238	102452238	G	C	nonsynonymous	NA	NA	NA	NA
EHD4	chr15	42211452	42211452	C	T	nonsynonymous	nonsynonymous	nonsynonymous	NA	NA
EVPL	chr17	74020139	74020139	T	A	nonsynonymous	NA	nonsynonymous	NA	NA
FABP9	chr8	82373696	82373696	C	G	nonsynonymous	nonsynonymous	nonsynonymous	NA	NA
FAM110B	chr8	59059714	59059714	C	T	nonsynonymous	nonsynonymous	nonsynonymous	NA	NA
FAM126B	chr2	201846438	201846438	G	A	nonsynonymous	nonsynonymous	nonsynonymous	NA	NA
FAM135B	chr8	139180232	139180232	C	T	nonsynonymous	nonsynonymous	nonsynonymous	NA	NA
FAM186A	chr12	50754614	50754614	C	T	nonsynonymous	NA	nonsynonymous	NA	NA
FAM71B	chr5	156589917	156589917	G	C	nonsynonymous	NA	nonsynonymous	NA	NA
FBXO43	chr8	101157293	101157293	G	C	stopgain	NA	NA	NA	NA
FGFR1OP	chr6	167446097	167446097	G	A	nonsynonymous	NA	NA	NA	NA
FOXP1	chr3	71027021	71027021	G	A	nonsynonymous	nonsynonymous	nonsynonymous	NA	NA
GCOM1;POLR2M	chr15	58001222	58001222	G	C	nonsynonymous	nonsynonymous	nonsynonymous	NA	NA
GOLGA1	chr9	127689013	127689013	G	C	stopgain	NA	NA	NA	NA
GOLGA6L2	chr15	23686672	23686673	CG	-	frameshift_deletion	NA	frameshift_deletion	NA	NA
GOLGA6L2	chr15	23686676	23686694	TCTTCTCCTCCTGCTCCCG	-	frameshift_deletion	NA	frameshift_deletion	NA	NA
GON4L	chr1	155722137	155722137	C	G	nonsynonymous	nonsynonymous	nonsynonymous	NA	NA
GRB14	chr2	165353778	165353778	G	A	nonsynonymous	NA	NA	NA	NA
GRIK3	chr1	37271790	37271790	G	C	nonsynonymous	nonsynonymous	nonsynonymous	NA	NA
HGF	chr7	81381472	81381472	G	T	nonsynonymous	nonsynonymous	nonsynonymous	NA	NA
HIST1H4I	chr6	27107093	27107093	-	G	frameshift_insertion	frameshift_insertion	frameshift_insertion	NA	NA
HS3ST4	chr16	26147270	26147270	G	C	nonsynonymous	NA	NA	NA	NA
IFNA5	chr9	21305033	21305033	G	C	nonsynonymous	NA	nonsynonymous	NA	NA
INPP5F	chr10	121586241	121586241	C	T	nonsynonymous	nonsynonymous	nonsynonymous	NA	NA
IQSEC3	chr12	284141	284141	C	T	nonsynonymous	nonsynonymous	nonsynonymous	NA	NA
ITGA4	chr2	182395266	182395266	T	C	nonsynonymous	nonsynonymous	nonsynonymous	NA	NA
KANSL1L	chr2	211019010	211019010	C	A	nonsynonymous	NA	nonsynonymous	NA	NA
KBTBD13	chr15	65370165	65370165	C	G	nonsynonymous	NA	nonsynonymous	NA	NA
KCNMB3	chr3	178960748	178960748	G	A	nonsynonymous	nonsynonymous	nonsynonymous	NA	NA
KERA	chr12	91449434	91449434	G	C	nonsynonymous	NA	NA	NA	NA
KIF26B	chr1	245530163	245530163	G	A	nonsynonymous	nonsynonymous	nonsynonymous	NA	NA
KLC3	chr19	45852172	45852172	G	C	nonsynonymous	nonsynonymous	nonsynonymous	NA	NA
KMT2D	chr12	49427283	49427283	-	TGCA	frameshift_insertion	frameshift_insertion	frameshift_insertion	NA	NA
KRTAP19-4	chr21	31869328	31869328	C	T	nonsynonymous	nonsynonymous	nonsynonymous	NA	NA
LAMA4	chr6	112575005	112575005	C	G	nonsynonymous	nonsynonymous	nonsynonymous	NA	NA
MED13	chr17	60062098	60062098	G	T	nonsynonymous	nonsynonymous	nonsynonymous	NA	NA
MKI67	chr10	129901961	129901961	C	G	nonsynonymous	nonsynonymous	nonsynonymous	NA	NA
MSH6	chr2	48027313	48027313	C	G	nonsynonymous	nonsynonymous	nonsynonymous	NA	NA
MTF1	chr1	38288254	38288254	G	T	nonsynonymous	nonsynonymous	NA	NA	NA
MYLK	chr3	123401128	123401128	G	A	nonsynonymous	NA	NA	NA	NA
MZF1	chr19	59074704	59074704	C	T	nonsynonymous	nonsynonymous	nonsynonymous	NA	NA
NAF1	chr4	164085538	164085538	G	C	nonsynonymous	nonsynonymous	nonsynonymous	NA	NA
NAV1	chr1	201752599	201752599	G	C	nonsynonymous	nonsynonymous	nonsynonymous	NA	NA
NFATC3	chr16	68156187	68156187	A	G	nonsynonymous	nonsynonymous	nonsynonymous	NA	NA
NFS1	chr20	34278406	34278406	G	A	nonsynonymous	nonsynonymous	nonsynonymous	NA	NA
NOB1	chr16	69782950	69782950	A	T	nonsynonymous	nonsynonymous	nonsynonymous	NA	NA
NPHP3	chr3	132437928	132437928	C	T	nonsynonymous	NA	NA	NA	NA
NPY2R	chr4	156135730	156135730	C	-	frameshift_deletion	NA	NA	NA	NA
NRDC	chr1	52256649	52256649	C	T	nonsynonymous	nonsynonymous	nonsynonymous	NA	NA
NREP	chr5	111066701	111066701	C	T	nonsynonymous	NA	nonsynonymous	NA	NA
NRXN1	chr2	50847162	50847162	C	G	nonsynonymous	nonsynonymous	nonsynonymous	NA	NA
NSD3	chr8	38205419	38205419	G	T	nonsynonymous	NA	NA	NA	NA
OR4K14	chr14	20482969	20482969	G	T	stopgain	NA	stopgain	NA	NA
OR8A1	chr11	124440202	124440202	C	G	nonsynonymous	nonsynonymous	nonsynonymous	NA	NA
PAN3	chr13	28851481	28851481	C	G	nonsynonymous	NA	NA	NA	NA
PAX1	chr20	21689262	21689262	G	A	nonsynonymous	NA	NA	NA	NA
PCDHA4	chr5	140188963	140188963	G	A	nonsynonymous	NA	NA	NA	NA
PCDHA6	chr5	140208958	140208958	C	T	nonsynonymous	nonsynonymous	nonsynonymous	NA	NA
PCDHA7	chr5	140215824	140215824	G	T	nonsynonymous	nonsynonymous	nonsynonymous	NA	NA
PCDHA8	chr5	140222773	140222773	C	T	nonsynonymous	NA	NA	NA	NA
PCDHGB5	chr5	140780062	140780062	T	A	nonsynonymous	nonsynonymous	nonsynonymous	NA	NA
PCLO	chr7	82584397	82584397	C	G	nonsynonymous	NA	nonsynonymous	NA	NA
PDE8B	chr5	76700554	76700554	C	T	nonsynonymous	nonsynonymous	nonsynonymous	NA	NA
PDIA5	chr3	122849442	122849442	C	T	nonsynonymous	nonsynonymous	NA	NA	NA
PHLPP2	chr16	71748644	71748644	G	C	nonsynonymous	nonsynonymous	nonsynonymous	NA	NA
PIK3R4	chr3	130403166	130403166	G	T	nonsynonymous	NA	nonsynonymous	NA	NA
PLLP	chr16	57290878	57290878	G	A	stopgain	NA	stopgain	NA	NA
PNN	chr14	39650572	39650572	G	C	nonsynonymous	nonsynonymous	nonsynonymous	NA	NA
POLR1B	chr2	113332777	113332777	A	G	nonsynonymous	nonsynonymous	nonsynonymous	NA	NA
PRKCI	chr3	169985778	169985778	G	A	nonsynonymous	nonsynonymous	NA	NA	NA
PTPN22	chr1	114372619	114372619	G	A	nonsynonymous	NA	nonsynonymous	NA	NA

PTPRT	chr20	40944523	40944523	G	T	nonsynonymous	NA	NA	NA	NA
RAB11A	chr15	66170108	66170108	G	A	nonsynonymous	nonsynonymous	nonsynonymous	NA	NA
SALL2	chr14	21991070	21991070	T	C	nonsynonymous	nonsynonymous	nonsynonymous	NA	NA
SCN5A	chr3	38647552	38647552	C	T	nonsynonymous	nonsynonymous	nonsynonymous	NA	NA
SEPT10	chr2	110332340	110332340	G	A	stopgain	stopgain	stopgain	NA	NA
SETD2	chr3	47084166	47084166	G	A	nonsynonymous	nonsynonymous	nonsynonymous	NA	NA
SLC13A4	chr7	135376361	135376361	A	G	nonsynonymous	nonsynonymous	nonsynonymous	NA	NA
SLC44A5	chr1	75684378	75684378	G	C	nonsynonymous	nonsynonymous	nonsynonymous	NA	NA
SNAP23	chr15	42821873	42821873	C	G	nonsynonymous	nonsynonymous	nonsynonymous	NA	NA
SOSTDC1	chr7	16502336	16502336	C	T	nonsynonymous	nonsynonymous	nonsynonymous	NA	NA
SRBD1	chr2	45704164	45704164	C	G	nonsynonymous	nonsynonymous	nonsynonymous	NA	NA
SRPK2	chr7	104766771	104766771	G	C	nonsynonymous	nonsynonymous	nonsynonymous	NA	NA
STOX2	chr4	184931745	184931745	A	G	nonsynonymous	nonsynonymous	nonsynonymous	NA	NA
STPG2	chr4	98633965	98633965	C	T	nonsynonymous	NA	NA	NA	NA
SYBU	chr8	110592166	110592166	G	A	nonsynonymous	nonsynonymous	nonsynonymous	NA	NA
TAF5	chr10	105147777	105147777	A	G	nonsynonymous	NA	NA	NA	NA
TBC1D20	chr20	419457	419457	C	T	nonsynonymous	nonsynonymous	nonsynonymous	NA	NA
TECPR1	chr7	97847039	97847039	C	T	nonsynonymous	nonsynonymous	nonsynonymous	NA	NA
TESK2	chr1	45811588	45811588	C	G	nonsynonymous	nonsynonymous	nonsynonymous	NA	NA
TMEM108	chr3	133114718	133114718	C	G	stopgain	stopgain	stopgain	NA	NA
TONSL	chr8	145663936	145663936	C	G	nonsynonymous	NA	NA	NA	NA
TP53	chr17	7578406	7578406	C	T	nonsynonymous	nonsynonymous	nonsynonymous	NA	NA
TPR	chr1	186329460	186329460	G	C	nonsynonymous	NA	NA	NA	NA
TRIP12	chr2	230655931	230655931	T	G	nonsynonymous	nonsynonymous	nonsynonymous	NA	NA
TSPYL6	chr2	54482264	54482264	C	T	nonsynonymous	nonsynonymous	nonsynonymous	NA	NA
TTC6	chr14	38208205	38208206	TC	-	frameshift_deletion	frameshift_deletion	frameshift_deletion	NA	NA
TTN	chr2	179610331	179610331	G	C	nonsynonymous	nonsynonymous	nonsynonymous	NA	NA
UAP1L1	chr9	139974585	139974585	G	A	nonsynonymous	nonsynonymous	nonsynonymous	NA	NA
VAPA	chr18	9936128	9936128	A	G	nonsynonymous	nonsynonymous	nonsynonymous	NA	NA
VCAM1	chr1	101194829	101194829	T	A	nonsynonymous	nonsynonymous	nonsynonymous	NA	NA
VILL	chr3	38048460	38048460	G	C	nonsynonymous	NA	NA	NA	NA
ZKSCAN2	chr16	25266553	25266553	C	T	nonsynonymous	NA	NA	NA	NA
ZNF638	chr2	71576926	71576926	A	T	nonsynonymous	nonsynonymous	nonsynonymous	NA	NA
ZNF790	chr19	37309724	37309724	-	T	frameshift_insertion	frameshift_insertion	frameshift_insertion	NA	NA
ZNF837	chr19	58879199	58879199	G	A	nonsynonymous	NA	NA	NA	NA
CCDC180	chr9	100109613	100109613	T	A	NA	nonsynonymous	nonsynonymous	NA	NA
OR14A16	chr1	247978203	247978203	T	G	NA	nonsynonymous	nonsynonymous	NA	NA
TOPAZ1	chr3	44283838	44283838	G	A	NA	nonsynonymous	NA	NA	NA
ZNF525	chr19	53884547	53884547	G	T	NA	stopgain	NA	NA	NA
ARMC5	chr16	31473784	31473784	G	A	NA	NA	nonsynonymous	NA	NA
BFSP2	chr3	133167367	133167367	G	C	NA	NA	nonsynonymous	NA	NA
DNAH17	chr17	76510894	76510894	G	A	NA	NA	nonsynonymous	NA	NA
DUSP8	chr11	1579404	1579404	G	A	NA	NA	nonsynonymous	NA	NA
FBN2	chr5	127670916	127670916	G	A	NA	NA	nonsynonymous	NA	NA
HIVEP3	chr1	42047626	42047626	G	A	NA	NA	nonsynonymous	NA	NA
KLHL12	chr1	202861735	202861735	C	A	NA	NA	nonsynonymous	NA	NA
MAPK1	chr22	22160227	22160227	C	T	NA	NA	nonsynonymous	NA	NA
POLR2E	chr19	1089953	1089953	C	T	NA	NA	nonsynonymous	NA	NA
SEC16A	chr9	139357548	139357548	G	A	NA	NA	stopgain	NA	NA
TSC22D2	chr3	150127682	150127682	G	A	NA	NA	nonsynonymous	NA	NA
ZMYND15	chr17	4649166	4649166	G	A	NA	NA	nonsynonymous	NA	NA
ADGRE2	chr19	14876548	14876548	A	C	NA	NA	NA	nonsynonymous	NA
ADGRE5	chr19	14508538	14508538	T	G	NA	NA	NA	nonsynonymous	nonsynonymous
AJUBA	chr14	23451169	23451169	G	A	NA	NA	NA	stopgain	stopgain
ANXA2	chr15	60653141	60653141	T	C	NA	NA	NA	nonsynonymous	nonsynonymous
ARHGAP20	chr11	110582906	110582906	G	A	NA	NA	NA	nonsynonymous	nonsynonymous
ATAD2B	chr2	24110799	24110799	T	C	NA	NA	NA	nonsynonymous	NA
BCL2L2-PABPN1;PABP	chr14	23791398	23791398	A	T	NA	NA	NA	nonsynonymous	nonsynonymous
BTN1A1	chr6	26508956	26508956	G	T	NA	NA	NA	nonsynonymous	nonsynonymous
C11orf68	chr11	65685309	65685309	G	A	NA	NA	NA	nonsynonymous	NA
CD101	chr1	117556375	117556375	C	A	NA	NA	NA	nonsynonymous	nonsynonymous
CD163	chr12	7639130	7639130	C	A	NA	NA	NA	nonsynonymous	nonsynonymous
CD69	chr12	9907191	9907193	AAA	-	NA	NA	NA	nonframeshift_deletio	nonframeshift_deletion
CD69	chr12	9907196	9907219	CTTTGCCATTTGACCACTTCCATG	-	NA	NA	NA	nonframeshift_deletio	nonframeshift_deletion
CDK5RAP2	chr9	123230172	123230192	AACCCATTTCCCTGGAAACCC	-	NA	NA	NA	nonframeshift_deletio	nonframeshift_deletion
CEP85L	chr6	118887021	118887021	T	A	NA	NA	NA	stopgain	stopgain
CHD2	chr15	93540581	93540581	A	G	NA	NA	NA	nonsynonymous	nonsynonymous
CIR1	chr2	175246487	175246487	T	A	NA	NA	NA	nonsynonymous	nonsynonymous
CNTNAP5	chr2	125521651	125521651	T	A	NA	NA	NA	nonsynonymous	nonsynonymous
COL5A3	chr19	10084618	10084618	G	A	NA	NA	NA	nonsynonymous	nonsynonymous
COLEC12	chr18	346856	346856	C	A	NA	NA	NA	nonsynonymous	nonsynonymous
CSMD1	chr8	4494966	4494966	C	T	NA	NA	NA	nonsynonymous	nonsynonymous
CSMD3	chr8	113395868	113395868	C	T	NA	NA	NA	nonsynonymous	nonsynonymous
DCSTAMP	chr8	105361477	105361477	C	G	NA	NA	NA	nonsynonymous	NA
DFFA	chr1	10532380	10532380	C	A	NA	NA	NA	nonsynonymous	nonsynonymous
DGKI	chr7	137257533	137257533	T	C	NA	NA	NA	nonsynonymous	nonsynonymous
DNAH17	chr17	76488772	76488772	C	T	NA	NA	NA	nonsynonymous	nonsynonymous
EML3	chr11	62371420	62371420	C	T	NA	NA	NA	nonsynonymous	nonsynonymous
EYS	chr6	64516194	64516194	G	A	NA	NA	NA	nonsynonymous	nonsynonymous
FAM160A2	chr11	6244127	6244127	C	T	NA	NA	NA	nonsynonymous	NA
FAM217A	chr6	4068979	4068979	G	A	NA	NA	NA	nonsynonymous	nonsynonymous
FANK1	chr10	127697994	127697994	C	A	NA	NA	NA	nonsynonymous	nonsynonymous
FCGBP	chr19	40376631	40376631	G	C	NA	NA	NA	nonsynonymous	NA
FZD10	chr12	130648647	130648647	G	A	NA	NA	NA	nonsynonymous	NA
GABRG3	chr15	27777817	27777817	G	T	NA	NA	NA	nonsynonymous	NA
GALNT8	chr12	4835889	4835889	G	T	NA	NA	NA	nonsynonymous	NA
GIPC2	chr1	78601320	78601320	G	A	NA	NA	NA	nonsynonymous	NA
GOLGA6L2	chr15	23685526	23685547	CTGCTCCCACATCTTCTTCTTC	-	NA	NA	NA	frameshift_deletion	NA
GRIK4	chr11	120769244	120769244	G	A	NA	NA	NA	nonsynonymous	nonsynonymous
GRIN2B	chr12	13720077	13720077	G	A	NA	NA	NA	nonsynonymous	nonsynonymous
HECTD1	chr14	31598609	31598609	C	A	NA	NA	NA	nonsynonymous	nonsynonymous
HENMT1	chr1	109191411	109191411	T	C	NA	NA	NA	nonsynonymous	nonsynonymous
HRH3	chr20	60791141	60791141	C	T	NA	NA	NA	nonsynonymous	nonsynonymous
HSPA13	chr21	15750562	15750562	G	C	NA	NA	NA	nonsynonymous	nonsynonymous
ILF2	chr1	153636580	153636580	T	C	NA	NA	NA	nonsynonymous	nonsynonymous
KIF18A	chr11	28119181	28119181	T	C	NA	NA	NA	nonsynonymous	nonsynonymous

KLHL25	chr15	86311930	86311930	G	A	NA	NA	NA	nonsynonymous	nonsynonymous
LAMA5	chr20	60885094	60885094	G	A	NA	NA	NA	nonsynonymous	NA
LRIG3	chr12	59281713	59281713	C	T	NA	NA	NA	nonsynonymous	nonsynonymous
LRRC70	chr5	61875626	61875626	A	C	NA	NA	NA	nonsynonymous	nonsynonymous
MED13L	chr12	116446719	116446719	T	A	NA	NA	NA	nonsynonymous	nonsynonymous
MNS1	chr15	56735897	56735897	T	G	NA	NA	NA	nonsynonymous	nonsynonymous
MROH2B	chr5	41061806	41061806	C	G	NA	NA	NA	nonsynonymous	nonsynonymous
MUC13	chr3	124646630	124646630	G	T	NA	NA	NA	nonsynonymous	nonsynonymous
MYH2	chr17	10443965	10443965	T	G	NA	NA	NA	nonsynonymous	nonsynonymous
MYH8	chr17	10317314	10317314	G	C	NA	NA	NA	nonsynonymous	nonsynonymous
NCLN	chr19	3204045	3204045	T	A	NA	NA	NA	nonsynonymous	NA
NDNF	chr4	121961120	121961120	T	A	NA	NA	NA	nonsynonymous	nonsynonymous
NF1	chr17	29670030	29670030	C	T	NA	NA	NA	nonsynonymous	nonsynonymous
NFE2L2	chr2	178098975	178098975	A	G	NA	NA	NA	nonsynonymous	nonsynonymous
OPN1LW	chrX	153420130	153420130	G	A	NA	NA	NA	nonsynonymous	nonsynonymous
OR5D18	chr11	55587304	55587304	C	A	NA	NA	NA	nonsynonymous	NA
PCDH8	chr13	53420005	53420005	C	A	NA	NA	NA	nonsynonymous	NA
PHLDB2	chr3	111659492	111659492	A	T	NA	NA	NA	nonsynonymous	nonsynonymous
PIBF1	chr13	73401956	73401956	G	A	NA	NA	NA	nonsynonymous	nonsynonymous
POM121L2	chr6	27279369	27279369	G	A	NA	NA	NA	nonsynonymous	NA
PROS1	chr3	93624735	93624735	G	C	NA	NA	NA	stopgain	stopgain
PRRC2C	chr1	171526539	171526583	CAGTTCCAGCCTCAACCTCAGCTCCACTTCCGGCAACCTTAACTC	-	NA	NA	NA	nonframeshift_deletic	nonframeshift_deletion
PTPN21	chr14	88946460	88946460	C	T	NA	NA	NA	nonsynonymous	nonsynonymous
PTPRH	chr19	55708741	55708741	C	G	NA	NA	NA	nonsynonymous	nonsynonymous
REG3G	chr2	79253842	79253842	A	G	NA	NA	NA	nonsynonymous	nonsynonymous
RFX1	chr19	14080828	14080828	G	A	NA	NA	NA	stopgain	NA
RUNDC3B	chr7	87329815	87329815	A	C	NA	NA	NA	nonsynonymous	nonsynonymous
RXYLT1	chr12	64202547	64202547	C	G	NA	NA	NA	nonsynonymous	nonsynonymous
SIPA1L1	chr14	72138338	72138338	-	GAGGA	NA	NA	NA	frameshift_insertion	frameshift_insertion
SLC26A5	chr7	103018963	103018963	C	A	NA	NA	NA	nonsynonymous	NA
SLC34A3	chr9	140130626	140130626	C	G	NA	NA	NA	nonsynonymous	nonsynonymous
SLCO1B7	chr12	21229400	21229400	C	T	NA	NA	NA	nonsynonymous	nonsynonymous
SON	chr21	34926440	34926440	C	G	NA	NA	NA	nonsynonymous	nonsynonymous
SOX9	chr17	70120069	70120089	GGCGCCCCCGCAGCCGCAGGC	-	NA	NA	NA	nonframeshift_deletic	NA
THBS1	chr15	39879605	39879605	C	T	NA	NA	NA	nonsynonymous	NA
TMED5	chr1	93620352	93620352	C	A	NA	NA	NA	stopgain	stopgain
TMEM231	chr16	75590049	75590049	C	T	NA	NA	NA	nonsynonymous	NA
TPH2	chr12	72338219	72338219	C	G	NA	NA	NA	nonsynonymous	nonsynonymous
TRAK2	chr2	202251126	202251126	G	C	NA	NA	NA	nonsynonymous	NA
TRPC5OS	chrX	111145413	111145413	A	C	NA	NA	NA	stoploss	stoploss
TTN	chr2	179463361	179463361	G	A	NA	NA	NA	nonsynonymous	nonsynonymous
USP28	chr11	113675469	113675469	T	A	NA	NA	NA	nonsynonymous	nonsynonymous
VIPR2	chr7	158824703	158824703	C	T	NA	NA	NA	nonsynonymous	nonsynonymous
WDHD1	chr14	55475077	55475077	G	C	NA	NA	NA	nonsynonymous	NA
WDR37	chr10	1170932	1170932	C	A	NA	NA	NA	nonsynonymous	nonsynonymous
WDR73	chr15	85188791	85188791	A	G	NA	NA	NA	nonsynonymous	nonsynonymous
WDR75	chr2	190333278	190333278	A	G	NA	NA	NA	nonsynonymous	nonsynonymous
WNK4	chr17	40937354	40937354	G	A	NA	NA	NA	nonsynonymous	nonsynonymous
ZNF215	chr11	6953717	6953717	C	A	NA	NA	NA	nonsynonymous	nonsynonymous
ZNF443	chr19	12541927	12541927	T	A	NA	NA	NA	nonsynonymous	NA
ZNF653	chr19	11596499	11596499	G	T	NA	NA	NA	nonsynonymous	nonsynonymous
ZNF862	chr7	149558506	149558506	G	A	NA	NA	NA	nonsynonymous	nonsynonymous
FAT3	chr11	92088483	92088483	G	A	NA	NA	NA	NA	nonsynonymous

Gene	chromosome	Start	Stop	Ref	Alt	HK2-EA	HK2-HA	HK2-HB	HK2-HC
AP1M1	chr19	16338499	16338499	C	A	nonsynonymous	NA	NA	NA
ASPHD2	chr22	26829925	26829925	G	A	nonsynonymous	NA	NA	NA
ATP7B	chr13	52539020	52539020	G	C	nonsynonymous	NA	NA	NA
BIRC6	chr2	32698206	32698206	A	T	nonsynonymous	NA	NA	NA
BRCA2	chr13	32911760	32911760	A	T	nonsynonymous	NA	NA	NA
CCDC138	chr2	109473300	109473300	G	C	nonsynonymous	NA	NA	NA
CDC42BPA	chr1	227288705	227288705	G	T	nonsynonymous	NA	NA	NA
CEP290	chr12	88444203	88444203	A	T	nonsynonymous	NA	NA	NA
CITED4	chr1	41327744	41327744	G	A	nonsynonymous	NA	NA	NA
CLK3	chr15	74918186	74918186	-	T	frameshift_insertion	NA	NA	NA
CREBBP	chr16	3788617	3788617	C	T	nonsynonymous	NA	NA	NA
CTU2	chr16	88780140	88780140	G	A	nonsynonymous	NA	NA	NA
DENND2C	chr1	115141992	115141992	G	A	nonsynonymous	NA	NA	NA
ETAA1	chr2	67631603	67631603	G	C	nonsynonymous	NA	NA	NA
FAM227A	chr22	39003421	39003421	T	-	frameshift_deletion	NA	NA	NA
FBXL22	chr15	63889922	63889922	T	C	nonsynonymous	NA	NA	NA
GAN	chr16	81391441	81391441	G	A	nonsynonymous	NA	NA	NA
GC	chr4	72620192	72620192	T	A	nonsynonymous	NA	NA	NA
GFRA1	chr10	117884998	117884998	C	G	nonsynonymous	NA	NA	NA
GINS4	chr8	41387773	41387773	G	C	nonsynonymous	NA	NA	NA
GMDS	chr6	1930368	1930368	C	G	nonsynonymous	NA	NA	NA
GNG3	chr11	62475800	62475800	G	C	nonsynonymous	NA	NA	NA
GRM6	chr5	178409981	178409981	-	T	frameshift_insertion	NA	NA	NA
H2AFV	chr7	44874119	44874119	C	T	nonsynonymous	NA	NA	NA
HAUS2	chr15	42858829	42858829	A	G	nonsynonymous	NA	NA	NA
HIRIP3	chr16	30004970	30004970	C	A	nonsynonymous	NA	NA	NA
ILK	chr11	6630184	6630184	C	T	nonsynonymous	NA	NA	NA
INHA	chr2	220440101	220440103	CTC	-	nonframeshift_deletion	NA	NA	NA
IRGC	chr19	44223138	44223138	G	A	nonsynonymous	NA	NA	NA
KDM6A	chrX	44929544	44929544	-	T	frameshift_insertion	NA	NA	NA
KIFC3	chr16	57805187	57805187	C	T	nonsynonymous	NA	NA	NA
KLHL1	chr13	70681819	70681819	C	A	nonsynonymous	NA	NA	NA
KRT76	chr12	53170560	53170560	G	C	nonsynonymous	NA	NA	NA
LAMA1	chr18	6985617	6985617	T	C	nonsynonymous	NA	NA	NA
LENG8	chr19	54969003	54969003	G	A	nonsynonymous	NA	NA	NA
LRRC1	chr6	53764611	53764611	G	A	nonsynonymous	NA	NA	NA
MB21D2	chr3	192516720	192516720	G	A	stopgain	NA	NA	NA
MSL2	chr3	135870794	135870794	G	C	nonsynonymous	NA	NA	NA
MUC4	chr3	195497190	195497190	G	C	nonsynonymous	NA	NA	NA
MYH10	chr17	8424595	8424595	T	C	nonsynonymous	NA	NA	NA
NAT16	chr7	100818013	100818013	C	G	nonsynonymous	NA	NA	NA
NEDD1	chr12	97334289	97334289	G	A	nonsynonymous	NA	NA	NA
NFATC4	chr14	24843071	24843071	G	T	nonsynonymous	NA	NA	NA
NKTR	chr3	42685469	42685469	-	GG	frameshift_insertion	NA	NA	NA
OR5A2	chr11	59189612	59189612	T	C	nonsynonymous	NA	NA	NA
OR6C3	chr12	55725849	55725849	C	G	nonsynonymous	NA	NA	NA
PCK1	chr20	56138099	56138099	A	G	nonsynonymous	NA	NA	NA
PDE1C	chr7	31887608	31887608	C	G	nonsynonymous	NA	NA	NA
PGBD1	chr6	28268666	28268666	A	T	nonsynonymous	NA	NA	NA
PHLPP1	chr18	60497276	60497276	C	G	nonsynonymous	NA	NA	NA
PKP4	chr2	159481532	159481532	C	T	nonsynonymous	NA	NA	NA
PLA2R1	chr2	160824054	160824054	T	C	nonsynonymous	NA	NA	NA
PRKAR2A	chr3	48789149	48789149	T	C	nonsynonymous	NA	NA	NA
PRPF4B	chr6	4044230	4044230	A	T	nonsynonymous	NA	NA	NA
PRR11	chr17	57262447	57262447	G	C	nonsynonymous	NA	NA	NA
PSMC1	chr14	90731465	90731465	A	G	nonsynonymous	NA	NA	NA
RAD17	chr5	68669849	68669849	G	C	nonsynonymous	NA	NA	NA
RBM14-RBM4;RBM4	chr11	66411453	66411459	AGCCCCA	-	frameshift_deletion	NA	NA	NA
RFWD3	chr16	74662559	74662559	G	A	nonsynonymous	NA	NA	NA
ROCK2	chr2	11332439	11332439	G	C	nonsynonymous	NA	NA	NA
SACS	chr13	23908096	23908096	T	C	nonsynonymous	NA	NA	NA
SCYL3	chr1	169847922	169847922	A	T	nonsynonymous	NA	NA	NA
SEMA3E	chr7	83032061	83032061	A	G	nonsynonymous	NA	NA	NA
SGO2	chr2	201438238	201438238	C	T	nonsynonymous	NA	NA	NA
SIGLEC14	chr19	52149575	52149575	C	T	nonsynonymous	NA	NA	NA
SLC12A3	chr16	56918054	56918054	C	T	nonsynonymous	NA	NA	NA
SLC24A1	chr15	65946389	65946389	G	A	nonsynonymous	NA	NA	NA
SLIT2	chr4	20570505	20570505	G	A	nonsynonymous	NA	NA	NA
SMAD7	chr18	46468987	46468987	C	G	nonsynonymous	NA	NA	NA
SRSF6	chr20	42089573	42089573	A	G	nonsynonymous	NA	NA	NA
TMEM81	chr1	205053338	205053338	T	A	nonsynonymous	NA	NA	NA
TP53	chr17	7578239	7578239	C	A	stopgain	NA	NA	NA
TRIM35	chr8	27145548	27145548	T	C	nonsynonymous	NA	NA	NA
TRIML1	chr4	189068224	189068224	C	T	nonsynonymous	NA	NA	NA
TSGA10	chr2	99722106	99722106	G	T	nonsynonymous	NA	NA	NA
TTN	chr2	179399307	179399307	A	T	nonsynonymous	NA	NA	NA
UNC5A	chr5	176295908	176295908	G	A	nonsynonymous	NA	NA	NA
USP20	chr9	132623300	132623300	C	G	nonsynonymous	NA	NA	NA
USP7	chr16	9009113	9009113	T	C	nonsynonymous	NA	NA	NA
VEPH1	chr3	157146149	157146149	G	A	nonsynonymous	NA	NA	NA
VSTM4	chr10	50285359	50285359	T	C	nonsynonymous	NA	NA	NA

WDR35	chr2	20180509	20180509	G	C	nonsynonymous	NA	NA	NA
ZCCHC4	chr4	25351123	25351123	G	C	nonsynonymous	NA	NA	NA
ZNF534	chr19	52942520	52942520	G	C	nonsynonymous	NA	NA	NA
ZNF559	chr19	9449218	9449218	A	T	nonsynonymous	NA	NA	NA
ZNF57	chr19	2917815	2917815	A	G	nonsynonymous	NA	NA	NA
ZNF806	chr2	133075782	133075782	C	G	nonsynonymous	NA	NA	NA
ADAM21	chr14	70925031	70925031	T	A	NA	nonsynonymous	nonsynonymous	nonsynonymous
ANKRD1	chr10	92675358	92675358	C	T	NA	nonsynonymous	nonsynonymous	nonsynonymous
AP4B1	chr1	114444400	114444400	T	C	NA	nonsynonymous	nonsynonymous	NA
ARHGAP24	chr4	86863350	86863350	C	T	NA	stopgain	stopgain	stopgain
ASAP2	chr2	9463302	9463302	C	T	NA	nonsynonymous	nonsynonymous	nonsynonymous
BMP2K	chr4	79786712	79786712	A	G	NA	nonsynonymous	nonsynonymous	nonsynonymous
CCDC158	chr4	77288456	77288456	C	G	NA	nonsynonymous	nonsynonymous	nonsynonymous
CD72	chr9	35616185	35616185	G	A	NA	nonsynonymous	nonsynonymous	nonsynonymous
CORO2A	chr9	100897167	100897167	C	A	NA	nonsynonymous	nonsynonymous	nonsynonymous
CPSF2	chr14	92627494	92627505	GCTGTCAGACTT	-	NA	nonframeshift_deletion	NA	nonframeshift_deletion
CRBN	chr3	3215795	3215795	T	C	NA	nonsynonymous	nonsynonymous	nonsynonymous
DCAF12L2	chrX	125298984	125298984	C	G	NA	nonsynonymous	nonsynonymous	nonsynonymous
DCT	chr13	95131431	95131431	G	A	NA	stopgain	stopgain	stopgain
DDX17	chr22	38891881	38891881	T	C	NA	nonsynonymous	nonsynonymous	nonsynonymous
DENND3	chr8	142204195	142204195	G	A	NA	nonsynonymous	nonsynonymous	nonsynonymous
DENND6A	chr3	57619039	57619039	G	T	NA	nonsynonymous	nonsynonymous	nonsynonymous
DPPA2	chr3	109031443	109031443	C	T	NA	nonsynonymous	NA	nonsynonymous
EYS	chr6	66042288	66042288	G	T	NA	nonsynonymous	nonsynonymous	nonsynonymous
FAM216A	chr12	110924172	110924172	A	G	NA	nonsynonymous	nonsynonymous	nonsynonymous
FREM2	chr13	39435627	39435627	C	G	NA	nonsynonymous	NA	nonsynonymous
FTSJ1	chrX	48340811	48340811	G	A	NA	nonsynonymous	nonsynonymous	nonsynonymous
FYB2	chr1	57216864	57216864	C	A	NA	stopgain	stopgain	stopgain
GALNT13	chr2	155158009	155158009	G	A	NA	nonsynonymous	nonsynonymous	nonsynonymous
GINS1	chr20	25426590	25426590	T	A	NA	nonsynonymous	nonsynonymous	nonsynonymous
GJD4	chr10	35896793	35896793	G	A	NA	nonsynonymous	NA	NA
GTF2I	chr7	74152437	74152437	A	G	NA	nonsynonymous	nonsynonymous	nonsynonymous
IL5RA	chr3	3139741	3139741	C	G	NA	nonsynonymous	nonsynonymous	nonsynonymous
KIAA0825	chr5	93739413	93739413	C	A	NA	nonsynonymous	nonsynonymous	nonsynonymous
KIF3A	chr5	132052611	132052611	T	C	NA	nonsynonymous	nonsynonymous	nonsynonymous
KLHDC2	chr14	50245136	50245136	G	C	NA	nonsynonymous	nonsynonymous	nonsynonymous
LDB2	chr4	16510245	16510245	G	-	NA	frameshift_deletion	frameshift_deletion	frameshift_deletion
LRP1B	chr2	141816575	141816575	T	A	NA	nonsynonymous	nonsynonymous	nonsynonymous
MAL	chr2	95719184	95719184	G	A	NA	nonsynonymous	nonsynonymous	NA
MEGF8	chr19	42867339	42867339	C	G	NA	nonsynonymous	NA	nonsynonymous
MGMT	chr10	131565167	131565167	G	A	NA	nonsynonymous	nonsynonymous	NA
MPDZ	chr9	13138060	13138060	C	T	NA	nonsynonymous	nonsynonymous	nonsynonymous
NCOA2	chr8	71068716	71068716	C	-	NA	frameshift_deletion	frameshift_deletion	frameshift_deletion
NCOA3	chr20	46264169	46264169	A	G	NA	nonsynonymous	NA	NA
NEMP1	chr12	57463047	57463047	A	C	NA	nonsynonymous	nonsynonymous	nonsynonymous
OSBPL5	chr11	3115007	3115007	T	C	NA	nonsynonymous	nonsynonymous	nonsynonymous
PCDHB6	chr5	140530151	140530151	T	A	NA	nonsynonymous	nonsynonymous	nonsynonymous
PKP4	chr2	159499019	159499019	A	C	NA	nonsynonymous	nonsynonymous	nonsynonymous
PLCB3	chr11	64030178	64030178	G	A	NA	nonsynonymous	nonsynonymous	nonsynonymous
PLIN4	chr19	4512911	4512911	G	C	NA	nonsynonymous	NA	nonsynonymous
PRDM9	chr5	23527470	23527470	G	A	NA	nonsynonymous	nonsynonymous	NA
PRR23A	chr3	138724931	138724931	T	C	NA	nonsynonymous	NA	nonsynonymous
PWWP2A	chr5	159520542	159520546	TCCAC	-	NA	frameshift_deletion	frameshift_deletion	frameshift_deletion
RSPH4A	chr6	116943950	116943950	A	G	NA	nonsynonymous	nonsynonymous	nonsynonymous
SBF2	chr11	9983519	9983519	C	A	NA	nonsynonymous	nonsynonymous	nonsynonymous
SCIN	chr7	12610462	12610462	C	A	NA	nonsynonymous	NA	NA
SCMH1	chr1	41579102	41579102	C	G	NA	nonsynonymous	nonsynonymous	nonsynonymous
SLC22A6	chr11	62747326	62747326	C	A	NA	nonsynonymous	NA	NA
SLC24A2	chr9	19786359	19786359	C	G	NA	nonsynonymous	nonsynonymous	nonsynonymous
SLC26A7	chr8	92346620	92346620	T	C	NA	nonsynonymous	nonsynonymous	nonsynonymous
SLC39A4	chr8	145639136	145639136	G	T	NA	nonsynonymous	nonsynonymous	NA
SMIM7	chr19	16770909	16770909	T	C	NA	nonsynonymous	NA	nonsynonymous
SQOR	chr15	45974779	45974779	T	A	NA	nonsynonymous	nonsynonymous	nonsynonymous
STAM	chr10	17750779	17750779	A	G	NA	nonsynonymous	nonsynonymous	nonsynonymous
TEX15	chr8	30699645	30699645	C	T	NA	nonsynonymous	nonsynonymous	nonsynonymous
TNS3	chr7	47463772	47463772	C	A	NA	nonsynonymous	nonsynonymous	nonsynonymous
TP53	chr17	7578413	7578413	C	A	NA	nonsynonymous	nonsynonymous	nonsynonymous
TRIM36	chr5	114462504	114462504	T	C	NA	nonsynonymous	nonsynonymous	nonsynonymous
TRPM6	chr9	77390856	77390856	G	A	NA	nonsynonymous	NA	nonsynonymous
USP29	chr19	57640127	57640127	A	T	NA	nonsynonymous	nonsynonymous	nonsynonymous
USP4	chr3	49316261	49316261	C	T	NA	nonsynonymous	nonsynonymous	nonsynonymous
ZFP42	chr4	188924527	188924527	C	A	NA	nonsynonymous	nonsynonymous	nonsynonymous
ZNF267	chr16	31927209	31927209	C	G	NA	nonsynonymous	NA	nonsynonymous
ZNF384	chr12	6788217	6788217	T	C	NA	nonsynonymous	nonsynonymous	nonsynonymous
ZNF780A	chr19	40582020	40582020	G	T	NA	nonsynonymous	nonsynonymous	nonsynonymous
ANO5	chr11	22248887	22248887	G	A	NA	NA	nonsynonymous	nonsynonymous
ARHGAP10	chr4	148653470	148653472	GGA	-	NA	NA	nonframeshift_deletion	nonframeshift_deletion
ARSD	chrX	2836031	2836031	G	T	NA	NA	nonsynonymous	nonsynonymous
FASN	chr17	80041952	80041952	C	T	NA	NA	nonsynonymous	NA
LIMCH1	chr4	41686453	41686466	ATCAAAAGGAGTCC	-	NA	NA	frameshift_deletion	frameshift_deletion
LRBA	chr4	151835364	151835364	A	T	NA	NA	nonsynonymous	nonsynonymous

MUC5B	chr11	1262224	1262224	T	A	NA	NA	nonsynonymous	NA
OR52B6	chr11	5602773	5602773	C	A	NA	NA	nonsynonymous	nonsynonymous
SBF2	chr11	9985369	9985369	C	G	NA	NA	nonsynonymous	nonsynonymous
SP5	chr2	171573750	171573750	C	T	NA	NA	nonsynonymous	nonsynonymous
CDKN1A	chr6	36652068	36652068	G	A	NA	NA	NA	nonsynonymous
DEPDC4	chr12	100657332	100657332	G	C	NA	NA	NA	nonsynonymous
DNHD1	chr11	6561188	6561188	G	A	NA	NA	NA	nonsynonymous
DUSP11	chr2	73994332	73994332	T	C	NA	NA	NA	nonsynonymous
ERCC6	chr10	50680981	50680981	C	T	NA	NA	NA	nonsynonymous
FAT2	chr5	150900941	150900941	T	C	NA	NA	NA	nonsynonymous
FNDC5	chr1	33333829	33333829	G	T	NA	NA	NA	nonsynonymous
GAS2L1	chr22	29704315	29704315	C	G	NA	NA	NA	nonsynonymous
LRP10	chr14	23344314	23344314	C	T	NA	NA	NA	nonsynonymous
LRP10	chr14	23345286	23345286	C	A	NA	NA	NA	nonsynonymous
LRP10	chr14	23345300	23345300	C	A	NA	NA	NA	stopgain
OR10R2	chr1	158450611	158450611	A	T	NA	NA	NA	nonsynonymous
PIFO	chr1	111889277	111889277	G	C	NA	NA	NA	nonsynonymous
RNF217	chr6	125397873	125397873	G	A	NA	NA	NA	nonsynonymous
SBF1	chr22	50900701	50900701	G	A	NA	NA	NA	nonsynonymous
TMPRSS11A	chr4	68789884	68789884	G	A	NA	NA	NA	nonsynonymous

Gene	chromosome	Start	Stop	Ref	Alt	HK3-EB	HK3-HA
MAGI3	chr1	114128110	114128110	C	G	nonsynonymous	NA
CLCNKB	chr1	16377498	16377498	G	A	synonymous	NA
GPATCH2	chr1	217783695	217783695	T	G	nonsynonymous	NA
RYR2	chr1	237957188	237957188	C	T	stopgain	NA
SH3BP5L	chr1	249106146	249106146	C	T	nonsynonymous	NA
CFAP57	chr1	43685064	43685064	G	T	nonsynonymous	NA
C1orf141	chr1	67591529	67591529	G	A	stopgain	NA
ZNF644	chr1	91404033	91404033	G	C	nonsynonymous	NA
SLC9A4	chr2	103142744	103142744	C	T	synonymous	NA
ANAPC1	chr2	112620065	112620065	T	C	nonsynonymous	NA
FOXD4L1	chr2	114257805	114257805	C	G	nonsynonymous	NA
NUP35	chr2	184025797	184025797	C	T	stopgain	NA
FSIP2	chr2	186661001	186661001	G	A	synonymous	NA
COL3A1	chr2	189861196	189861196	G	C	nonsynonymous	NA
WDR75	chr2	190323579	190323579	G	A	nonsynonymous	NA
STAT4	chr2	191904012	191904012	C	T	synonymous	NA
NABP1	chr2	192550373	192550373	-	T	frameshift_insertion	NA
ADAM23	chr2	207310188	207310188	C	A	nonsynonymous	NA
IKZF2	chr2	213872586	213872586	T	A	nonsynonymous	NA
FBXO36	chr2	230875432	230875432	G	T	synonymous	NA
NEU2	chr2	233899696	233899696	G	C	nonsynonymous	NA
ALK	chr2	29456534	29456534	A	G	nonsynonymous	NA
CAPN13	chr2	30976021	30976021	T	C	nonsynonymous	NA
LTBP1	chr2	33335683	33335683	C	T	nonsynonymous	NA
EXOC6B	chr2	72411307	72411307	G	A	nonsynonymous	NA
REG3G	chr2	79254940	79254940	A	C	nonsynonymous	NA
KANSL3	chr2	97274297	97274297	C	T	synonymous	NA
RPL24	chr3	101405353	101405353	C	G	synonymous	NA
ZBTB20	chr3	114069199	114069199	T	C	nonsynonymous	NA
COL6A5	chr3	130098816	130098816	A	G	nonsynonymous	NA
DNAJC13	chr3	132172999	132172999	G	C	nonsynonymous	NA
DNAJC13	chr3	132233457	132233457	A	G	nonsynonymous	NA
SERPINI1	chr3	167508301	167508301	A	G	nonsynonymous	NA
B3GNT5	chr3	182988448	182988448	C	T	nonsynonymous	NA
BSN	chr3	49690695	49690695	G	C	nonsynonymous	NA
C3orf14	chr3	62317031	62317031	C	G	nonsynonymous	NA
TMF1	chr3	69101202	69101202	G	T	nonsynonymous	NA
EPHA6	chr3	97167450	97167450	A	T	nonsynonymous	NA
FREM3	chr4	144618199	144618199	G	C	synonymous	NA
RPS3A	chr4	152020822	152020822	C	T	synonymous	NA
FAM53A	chr4	1646036	1646036	C	A	nonsynonymous	NA
FRYL	chr4	48591879	48591879	T	C	nonsynonymous	NA
ENOPH1	chr4	83381252	83381252	A	G	synonymous	NA
HSD17B13	chr4	88243985	88243985	G	A	synonymous	NA
CTNND2	chr5	10988326	10988326	C	G	nonsynonymous	NA
SLC6A19	chr5	1212515	1212515	C	G	nonsynonymous	NA
SLC22A5	chr5	131705907	131705907	C	A	stopgain	NA
CXXC5	chr5	139060997	139060997	G	C	nonsynonymous	NA
NDUFA2	chr5	140025188	140025188	A	T	nonsynonymous	NA
PCDHA7	chr5	140214708	140214708	A	G	nonsynonymous	NA
PCDHB1	chr5	140433472	140433472	G	C	nonsynonymous	NA
PCDHGA10	chr5	140793367	140793367	G	A	nonsynonymous	NA
RBM22	chr5	150073798	150073798	G	T	nonsynonymous	NA
DHX29	chr5	54567970	54567970	T	C	nonsynonymous	NA
PIK3R1	chr5	67592100	67592100	G	A	nonsynonymous	NA
REV3L	chr6	111632401	111632401	T	C	nonsynonymous	NA
RSPH4A	chr6	116953503	116953503	G	T	stopgain	NA
PEX7	chr6	137234653	137234653	A	G	nonsynonymous	NA
SHPRH	chr6	146209163	146209163	C	T	nonsynonymous	NA
ARID1B	chr6	157454254	157454254	G	A	nonsynonymous	NA
MTCH1	chr6	36949391	36949391	G	A	nonsynonymous	NA
CUL7	chr6	43017845	43017845	A	G	synonymous	NA
CAPN11	chr6	44150747	44150747	G	A	nonsynonymous	NA
PHIP	chr6	79679876	79679876	C	T	nonsynonymous	NA
SERPINE1	chr7	100777096	100777096	C	T	nonsynonymous	NA
TMEM168	chr7	112424780	112424780	C	T	nonsynonymous	NA
PLXNA4	chr7	132192732	132192732	C	T	nonsynonymous	NA
UBN2	chr7	138969091	138969091	C	G	stopgain	NA
BRAF	chr7	140482830	140482830	C	A	nonsynonymous	NA
IGF2BP3	chr7	23391129	23391129	C	T	nonsynonymous	NA
PLEKHA8	chr7	30092476	30092476	A	G	nonsynonymous	NA
GRB10	chr7	50685833	50685833	A	T	nonsynonymous	NA
GRB10	chr7	50860157	50860157	C	A	nonsynonymous	NA
AKAP9	chr7	91727442	91727442	G	C	nonsynonymous	NA
ADAP1	chr7	944770	944770	T	C	nonsynonymous	NA
PDK4	chr7	95225541	95225541	C	T	nonsynonymous	NA
CSMD3	chr8	113314187	113314187	A	T	nonsynonymous	NA
COLEC10	chr8	120101975	120101975	C	A	nonsynonymous	NA
ZNF623	chr8	144733142	144733142	G	A	nonsynonymous	NA
SCRIB	chr8	144891764	144891764	G	A	nonsynonymous	NA
EPPK1	chr8	144944859	144944859	G	A	nonsynonymous	NA
SCARA3	chr8	27509077	27509077	A	G	synonymous	NA

LEPROTL1	chr8	29994866	29994866	C	G	nonsynonymous	NA
KAT6A	chr8	41905980	41905980	T	A	nonsynonymous	NA
GAPVD1	chr9	128122921	128122921	A	G	nonsynonymous	NA
NUP214	chr9	134072701	134072701	C	T	nonsynonymous	NA
ADAMTSL2	chr9	136405848	136405848	G	A	nonsynonymous	NA
CDC37L1	chr9	4701881	4701881	T	A	stopgain	NA
TRPM6	chr9	77377568	77377568	T	G	nonsynonymous	NA
SORCS1	chr10	108338876	108338876	C	T	nonsynonymous	NA
PLEKHA1	chr10	124175485	124175485	C	G	nonsynonymous	NA
MKI67	chr10	129913710	129913710	C	G	nonsynonymous	NA
WAC	chr10	28900717	28900717	C	T	stopgain	NA
WDFY4	chr10	49934004	49934004	G	A	nonsynonymous	NA
PCDH15	chr10	55568879	55568879	T	C	nonsynonymous	NA
CEP126	chr11	101833684	101833684	G	C	nonsynonymous	NA
CEP126	chr11	101833689	101833689	C	A	nonsynonymous	NA
ARHGEF12	chr11	120350901	120350901	A	G	synonymous	NA
DDX25	chr11	125776025	125776025	T	C	nonsynonymous	NA
ST14	chr11	130064142	130064142	C	T	nonsynonymous	NA
E2F8	chr11	19259629	19259629	C	G	synonymous	NA
DBX1	chr11	20180811	20180811	A	T	nonsynonymous	NA
SLC17A6	chr11	22363158	22363158	G	A	synonymous	NA
C11orf49	chr11	47183164	47183164	A	T	nonsynonymous	NA
OR51G2	chr11	4936359	4936359	G	A	nonsynonymous	NA
OR9G1;OR9G9	chr11	56468707	56468707	C	T	nonsynonymous	NA
TRIM34;TRIM6-T	chr11	5653641	5653641	G	T	nonsynonymous	NA
PHRF1	chr11	582033	582033	G	A	nonsynonymous	NA
ZP1	chr11	60638772	60638772	G	A	nonsynonymous	NA
SLC15A3	chr11	60711264	60711264	T	A	nonsynonymous	NA
ARFIP2	chr11	6498404	6498404	T	A	nonsynonymous	NA
ARHGEF17	chr11	73022366	73022366	C	A	nonsynonymous	NA
SYT9	chr11	7334976	7334976	C	G	nonsynonymous	NA
PRCP	chr11	82550304	82550304	T	A	nonsynonymous	NA
FAT3	chr11	92534953	92534953	C	T	nonsynonymous	NA
SLC36A4	chr11	92914005	92914005	G	C	nonsynonymous	NA
CEP295	chr11	93431741	93431741	A	T	synonymous	NA
UHRF1BP1L	chr12	100441946	100441946	G	A	synonymous	NA
NCOR2	chr12	124812086	124812086	T	C	nonsynonymous	NA
TMEM132C	chr12	129190388	129190388	C	A	nonsynonymous	NA
KLHL42	chr12	27950874	27950874	C	G	synonymous	NA
ALG10B	chr12	38714654	38714654	A	G	nonsynonymous	NA
LRRK2	chr12	40714970	40714970	C	G	nonsynonymous	NA
PRICKLE1	chr12	42858523	42858523	C	T	nonsynonymous	NA
ANO6	chr12	45741925	45741925	C	A	synonymous	NA
OR10AD1	chr12	48596599	48596599	A	T	nonsynonymous	NA
FAM186A	chr12	50749989	50749989	A	G	nonsynonymous	NA
SLC11A2	chr12	51393174	51393174	G	T	synonymous	NA
MBD6	chr12	57920994	57920994	C	T	nonsynonymous	NA
EEF1AKMT3	chr12	58174179	58174179	A	G	nonsynonymous	NA
USP15	chr12	62784747	62784747	G	C	nonsynonymous	NA
OTOGL	chr12	80699417	80699417	C	G	nonsynonymous	NA
NEDD1	chr12	97331040	97331040	A	G	nonsynonymous	NA
ZIC2	chr13	100635243	100635243	C	T	nonsynonymous	NA
COL4A2-AS2	chr13	111110045	111110045	G	A	nonsynonymous	NA
EEF1AKMT1	chr13	21331662	21331662	C	A	stopgain	NA
ATP12A	chr13	25272865	25272865	C	T	nonsynonymous	NA
FREM2	chr13	39263065	39263065	G	A	synonymous	NA
FREM2	chr13	39265046	39265046	G	T	stopgain	NA
RNF219	chr13	79190135	79190135	A	G	synonymous	NA
CEP170B	chr14	105352782	105352782	C	T	nonsynonymous	NA
CHD8	chr14	21873568	21873568	T	A	nonsynonymous	NA
STRN3	chr14	31495300	31495300	G	C	nonsynonymous	NA
NPAS3	chr14	34269677	34269677	G	A	nonsynonymous	NA
LRFN5	chr14	42356378	42356378	G	T	nonsynonymous	NA
PAPLN	chr14	73733512	73733513	GA	-	frameshift_deletion	NA
ACOT2	chr14	74036305	74036305	G	A	nonsynonymous	NA
NRXN3	chr14	80328256	80328256	G	C	synonymous	NA
UNC79	chr14	94109963	94109963	C	T	synonymous	NA
MKRN3	chr15	23810961	23810961	A	G	nonsynonymous	NA
GOLGA8Q	chr15	30846076	30846076	G	C	nonsynonymous	NA
CEP152	chr15	49031102	49031102	A	G	nonsynonymous	NA
FAM227B	chr15	49800534	49800534	G	C	nonsynonymous	NA
PRKCB	chr16	24135185	24135185	C	T	synonymous	NA
QPRT	chr16	29706404	29706404	C	T	nonsynonymous	NA
CAPN15	chr16	602924	602924	-	G	frameshift_insertion	NA
CMIP	chr16	81725432	81725432	C	A	nonsynonymous	NA
RAB11FIP4	chr17	29849026	29849026	C	G	synonymous	NA
OR1A1	chr17	3118939	3118939	A	G	nonsynonymous	NA
FAM187A	chr17	42982413	42982413	C	T	nonsynonymous	NA
NLRP1	chr17	5405135	5405135	T	G	stoploss	NA
NOG	chr17	54672004	54672004	G	T	nonsynonymous	NA
ABCA9	chr17	66982342	66982342	C	T	nonsynonymous	NA
COG1	chr17	71204502	71204502	C	A	nonsynonymous	NA
RPL38	chr17	72205418	72205418	G	A	nonsynonymous	NA

EXOC7	chr17	74097942	74097942	C	A	synonymous	NA
TP53	chr17	7577098	7577098	T	G	nonsynonymous	NA
DSC1	chr18	28720036	28720036	C	G	nonsynonymous	NA
SKOR2	chr18	44771305	44771305	C	T	nonsynonymous	NA
MYO5B	chr18	47369683	47369683	G	C	nonsynonymous	NA
LAMA1	chr18	6986218	6986218	G	A	nonsynonymous	NA
ZNF236	chr18	74637297	74637297	-	T	frameshift_insertion	NA
RBFA	chr18	77794531	77794531	C	T	synonymous	NA
MIDN	chr19	1251839	1251839	C	G	nonsynonymous	NA
AKAP8L	chr19	15491149	15491149	C	G	synonymous	NA
CPAMD8	chr19	17036151	17036151	G	C	synonymous	NA
IQCN	chr19	18376000	18376000	C	T	nonsynonymous	NA
PBX4	chr19	19675769	19675769	C	T	nonsynonymous	NA
MOB3A	chr19	2078319	2078319	C	T	nonsynonymous	NA
ZNF730	chr19	23329315	23329315	G	T	nonsynonymous	NA
OVOL3	chr19	36602108	36602108	C	G	nonsynonymous	NA
ETHE1	chr19	44030441	44030441	G	A	nonsynonymous	NA
TMEM143	chr19	48845964	48845964	C	T	synonymous	NA
ZNF836	chr19	52659649	52659649	G	C	synonymous	NA
VN1R2	chr19	53762292	53762292	C	G	nonsynonymous	NA
KIR3DL1	chr19	55341636	55341636	C	T	nonsynonymous	NA
DHX35	chr20	37634942	37634942	C	T	stopgain	NA
CDC25B	chr20	3784098	3784098	G	A	synonymous	NA
SEMG1	chr20	43836239	43836239	G	C	nonsynonymous	NA
FAM217B	chr20	58519846	58519846	G	C	nonsynonymous	NA
TPTE	chr21	10951337	10951337	C	T	synonymous	NA
TMPRSS15	chr21	19653536	19653536	C	G	nonsynonymous	NA
NF2	chr22	30090782	30090782	A	G	synonymous	NA
CHKB	chr22	51019915	51019915	G	A	nonsynonymous	NA
SHANK3	chr22	51144524	51144524	C	T	nonsynonymous	NA
WWC3	chrX	10058834	10058834	C	T	nonsynonymous	NA
NRK	chrX	105190322	105190322	G	C	nonsynonymous	NA
ALG13	chrX	110951474	110951474	G	T	synonymous	NA
ADGRG4	chrX	135405050	135405050	G	T	nonsynonymous	NA
MAGEB18	chrX	26157453	26157453	C	G	nonsynonymous	NA
VSIG4	chrX	65253398	65253398	G	C	nonsynonymous	NA
NLGN3	chrX	70367881	70367881	G	A	synonymous	NA
DACH2	chrX	85403936	85403936	A	G	synonymous	NA
FAM133A	chrX	92964914	92964914	G	A	nonsynonymous	NA
FAM133A	chrX	92964957	92964957	G	-	frameshift_deletion	NA
CDC14A	chr1	100949885	100949885	C	T	NA	stopgain
TAS1R3	chr1	1269443	1269443	G	C	NA	nonsynonymous
TARS2	chr1	150460385	150460385	G	C	NA	nonsynonymous
SETDB1	chr1	150936182	150936182	G	T	NA	nonsynonymous
FLG	chr1	152275863	152275863	A	C	NA	synonymous
ADAM15	chr1	155030138	155030138	C	G	NA	nonsynonymous
OR10J1	chr1	159409819	159409819	A	G	NA	nonsynonymous
PRRX1	chr1	170695386	170695386	A	C	NA	nonsynonymous
RGS16	chr1	182573355	182573355	C	T	NA	nonsynonymous
TPR	chr1	186322894	186322894	G	C	NA	nonsynonymous
PTGS2	chr1	186643529	186643529	T	C	NA	nonsynonymous
KCNT2	chr1	196300359	196300359	T	A	NA	nonsynonymous
F13B	chr1	197025003	197025003	G	-	NA	frameshift_deletion
PROX1	chr1	214170964	214170964	A	C	NA	nonsynonymous
CENPF	chr1	214814901	214814901	C	T	NA	synonymous
EPRS	chr1	220152760	220152760	C	T	NA	synonymous
RAB3GAP2	chr1	220445620	220445620	G	C	NA	nonsynonymous
OBSCN	chr1	228522538	228522538	G	A	NA	synonymous
RYR2	chr1	237608763	237608763	A	G	NA	synonymous
ZNF669	chr1	247264425	247264425	G	T	NA	nonsynonymous
OR6F1	chr1	247875632	247875632	C	T	NA	synonymous
OR2M4	chr1	248402688	248402688	C	T	NA	nonsynonymous
KPNA6	chr1	32620299	32620299	C	T	NA	nonsynonymous
CSMD2	chr1	34080172	34080172	G	T	NA	nonsynonymous
RLF	chr1	40697215	40697215	T	C	NA	nonsynonymous
RIMKLA	chr1	42880529	42880529	G	C	NA	nonsynonymous
CYP4Z1	chr1	47533222	47533222	C	G	NA	synonymous
NRDC	chr1	52274980	52274991	TACTGA CCTATG	-	NA	frameshift_deletion
FYB2	chr1	57254791	57254791	G	C	NA	synonymous
C8B	chr1	57415343	57415343	C	T	NA	nonsynonymous
CHD5	chr1	6185614	6185614	C	T	NA	synonymous
NOL9	chr1	6605202	6605202	C	G	NA	nonsynonymous
LRRIQ3	chr1	74507274	74507274	T	C	NA	synonymous
MRPS9	chr2	105708918	105708918	G	A	NA	synonymous
ST6GAL2	chr2	107423243	107423243	C	A	NA	nonsynonymous
GREB1	chr2	11778816	11778816	G	A	NA	nonsynonymous
DDX18	chr2	118572389	118572389	G	C	NA	nonsynonymous
RIF1	chr2	152321131	152321131	A	C	NA	nonsynonymous
SCN9A	chr2	167055310	167055310	C	T	NA	nonsynonymous
PXDN	chr2	1687431	1687431	C	G	NA	nonsynonymous
TTN	chr2	179455952	179455952	G	A	NA	nonsynonymous

FSIP2	chr2	186654000	186654000	C	T	NA	stopgain
COL3A1	chr2	189868141	189868141	C	T	NA	nonsynonymous
PARD3B	chr2	205989075	205989075	T	C	NA	nonsynonymous
PARD3B	chr2	206480377	206480377	G	A	NA	nonsynonymous
DYTN	chr2	207527804	207527804	C	T	NA	nonsynonymous
TDRD15	chr2	21365751	21365751	A	C	NA	nonsynonymous
FN1	chr2	216262403	216262403	G	A	NA	nonsynonymous
COL4A3	chr2	228175592	228175592	G	C	NA	nonsynonymous
AGFG1	chr2	228398325	228398325	C	G	NA	nonsynonymous
ARMC9	chr2	232156120	232156120	C	T	NA	nonsynonymous
MROH2A	chr2	234726672	234726672	G	T	NA	nonsynonymous
PRLH	chr2	238475761	238475761	A	C	NA	synonymous
OR6B2	chr2	240969261	240969261	C	T	NA	nonsynonymous
POMC	chr2	25384506	25384506	C	T	NA	nonsynonymous
EHD3	chr2	31483747	31483747	C	T	NA	nonsynonymous
GALM	chr2	38908463	38908463	C	G	NA	nonsynonymous
SLC8A1	chr2	40342434	40342434	A	C	NA	nonsynonymous
FSHR	chr2	49381455	49381455	C	A	NA	nonsynonymous
ALMS1	chr2	73786256	73786256	G	A	NA	synonymous
ZNF514	chr2	95815568	95815568	C	T	NA	nonsynonymous
TRIM43	chr2	96262091	96262091	G	A	NA	nonsynonymous
FER1L5	chr2	97335893	97335893	G	A	NA	nonsynonymous
DPPA4	chr3	109056341	109056343	AGA	-	NA	nonframeshift_deletion
CFAP44	chr3	113120477	113120477	G	C	NA	nonsynonymous
FBXO40	chr3	121340427	121340427	G	C	NA	nonsynonymous
COL6A6	chr3	130290200	130290200	A	G	NA	synonymous
GRK7	chr3	141497227	141497227	G	A	NA	nonsynonymous
U2SURP	chr3	142720500	142720500	G	C	NA	nonsynonymous
FGD5	chr3	14905697	14905697	C	T	NA	nonsynonymous
MED12L	chr3	151097965	151097965	G	C	NA	nonsynonymous
IQCJ-SCHIP1;SCH	chr3	159482423	159482423	G	A	NA	synonymous
PDCD10	chr3	167414911	167414911	C	T	NA	nonsynonymous
PIK3CA	chr3	178936082	178936082	G	A	NA	nonsynonymous
KNG1	chr3	186459435	186459435	G	A	NA	nonsynonymous
TP63	chr3	189587173	189587173	A	G	NA	nonsynonymous
IL1RAP	chr3	190366181	190366181	T	C	NA	nonsynonymous
CCDC50	chr3	191107357	191107357	A	T	NA	synonymous
KCNH8	chr3	19384145	19384145	T	C	NA	nonsynonymous
MUC4	chr3	195512942	195512942	C	G	NA	nonsynonymous
KAT2B	chr3	20156417	20156417	C	A	NA	nonsynonymous
XYLB	chr3	38417700	38417700	A	G	NA	nonsynonymous
SCN10A	chr3	38739228	38739228	T	G	NA	nonsynonymous
MYRIP	chr3	40293482	40293482	G	T	NA	nonsynonymous
ITPR1	chr3	4681083	4681083	G	C	NA	nonsynonymous
ZGRF1	chr4	113540360	113540360	G	C	NA	nonsynonymous
SEC24D	chr4	119673940	119673940	T	C	NA	nonsynonymous
TMEM131L	chr4	154544075	154544075	G	T	NA	nonsynonymous
CC2D2A	chr4	15572034	15572034	G	C	NA	nonsynonymous
NOP14	chr4	2944050	2944050	C	A	NA	synonymous
ADGRL3	chr4	62936529	62936529	G	A	NA	nonsynonymous
GNRHR	chr4	68619922	68619922	G	T	NA	nonsynonymous
PRR27	chr4	71024388	71024388	C	A	NA	nonsynonymous
PTPN13	chr4	87692681	87692681	G	A	NA	nonsynonymous
MMRN1	chr4	90874345	90874345	A	G	NA	nonsynonymous
CTNND2	chr5	10973724	10973724	C	T	NA	synonymous
APC	chr5	112174170	112174170	C	T	NA	nonsynonymous
DCANP1	chr5	134782502	134782502	C	T	NA	synonymous
SLC4A9	chr5	139739905	139739905	C	T	NA	synonymous
GEMIN5	chr5	154311731	154311731	C	T	NA	nonsynonymous
GEMIN5	chr5	154311737	154311737	C	T	NA	nonsynonymous
GABRA1	chr5	161322857	161322857	A	C	NA	nonsynonymous
MROH2B	chr5	41015544	41015544	T	C	NA	nonsynonymous
C6	chr5	41176671	41176671	G	C	NA	nonsynonymous
TMEM267	chr5	43454041	43454041	A	C	NA	nonsynonymous
MAST4	chr5	66461296	66461296	-	T	NA	frameshift_insertion
HMGCR	chr5	74655325	74655325	G	A	NA	nonsynonymous
IQGAP2	chr5	75969847	75969847	G	C	NA	nonsynonymous
SEMA5A	chr5	9154792	9154792	T	C	NA	nonsynonymous
GRIK2	chr6	102337730	102337730	C	T	NA	synonymous
MAP7	chr6	136681842	136681842	C	G	NA	nonsynonymous
TXLNB	chr6	139581478	139581478	G	A	NA	stopgain
PPIL4	chr6	149826604	149826604	T	A	NA	nonsynonymous
NOX3	chr6	155750181	155750181	C	G	NA	nonsynonymous
TAGAP	chr6	159457263	159457263	C	T	NA	nonsynonymous
PRPF4B	chr6	4052275	4052275	C	G	NA	synonymous
TFAP2B	chr6	50805768	50805768	A	G	NA	nonsynonymous
PHF3	chr6	64416090	64416090	A	C	NA	nonsynonymous
EYS	chr6	66205147	66205147	A	C	NA	nonsynonymous
DSP	chr6	7578086	7578086	G	A	NA	nonsynonymous
ZNF292	chr6	87967387	87967387	G	T	NA	nonsynonymous
ZNF292	chr6	87971225	87971225	T	A	NA	nonsynonymous
MUC17	chr7	100676033	100676033	A	C	NA	nonsynonymous
DNAJC2	chr7	102962963	102962963	C	G	NA	nonsynonymous

LAMB1	chr7	107642122	107642122	C	T	NA	nonsynonymous
NRCAM	chr7	107790568	107790568	C	G	NA	nonsynonymous
NDUFA4	chr7	10979653	10979653	T	-	NA	frameshift_deletion
ST7	chr7	116810970	116810970	G	T	NA	stopgain
ASZ1	chr7	117067469	117067469	C	T	NA	nonsynonymous
SSMEM1	chr7	129856137	129856137	C	T	NA	synonymous
CALD1	chr7	134642826	134642826	G	T	NA	stopgain
CHRM2	chr7	136699825	136699825	C	A	NA	synonymous
MGAM	chr7	141720850	141720850	A	G	NA	synonymous
TRPV5	chr7	142612707	142612707	C	A	NA	nonsynonymous
EPHA1	chr7	143096432	143096432	C	T	NA	nonsynonymous
ZNF282	chr7	148903799	148903799	T	A	NA	synonymous
KMT2C	chr7	151873365	151873365	A	T	NA	nonsynonymous
HDAC9	chr7	18705992	18705992	C	G	NA	nonsynonymous
MPP6	chr7	24727162	24727162	G	C	NA	nonsynonymous
BBS9	chr7	33185884	33185884	G	A	NA	nonsynonymous
AOAH	chr7	36571760	36571760	G	A	NA	nonsynonymous
TNS3	chr7	47384360	47384360	T	C	NA	synonymous
PKD1L1	chr7	47840429	47840429	G	C	NA	nonsynonymous
COBL	chr7	51111379	51111379	G	T	NA	synonymous
ZNF479	chr7	57199999	57199999	T	C	NA	synonymous
TRIM50;TRIM50	chr7	72732707	72732707	T	A	NA	nonsynonymous
GTF2IRD1	chr7	74015500	74015500	C	A	NA	stopgain
CD36	chr7	80290419	80290419	G	A	NA	nonsynonymous
TEX47	chr7	88423640	88423640	T	C	NA	nonsynonymous
ZNF804B	chr7	88963109	88963109	A	C	NA	nonsynonymous
STEAP1	chr7	89791276	89791276	A	G	NA	nonsynonymous
RBM48	chr7	92158910	92158910	C	G	NA	nonsynonymous
MCM7	chr7	99693569	99693569	G	C	NA	nonsynonymous
DCAF13	chr8	104427572	104427572	T	G	NA	synonymous
CSMD3	chr8	113402992	113402992	C	G	NA	nonsynonymous
TBC1D31	chr8	124138916	124138916	G	A	NA	nonsynonymous
DLC1	chr8	12943905	12943905	C	T	NA	nonsynonymous
ZNF623	chr8	144732964	144732964	G	T	NA	stopgain
DLGAP2	chr8	1649441	1649441	G	C	NA	nonsynonymous
CHMP7	chr8	23106816	23106816	C	G	NA	synonymous
RP1	chr8	55542568	55542568	A	G	NA	synonymous
CHD7	chr8	61732644	61732644	G	C	NA	nonsynonymous
CLVS1	chr8	62212402	62212402	C	G	NA	nonsynonymous
RPL7	chr8	74203792	74203792	G	A	NA	nonsynonymous
RALYL	chr8	85785556	85785556	A	-	NA	frameshift_deletion
MTERF3	chr8	97251843	97251843	T	C	NA	nonsynonymous
CPQ	chr8	97797224	97797224	T	C	NA	synonymous
OR13C3	chr9	107298340	107298340	A	G	NA	nonsynonymous
OLFML2A	chr9	127572288	127572288	C	T	NA	nonsynonymous
MVB12B	chr9	129102907	129102907	G	A	NA	nonsynonymous
SET	chr9	131446232	131446232	C	T	NA	nonsynonymous
PTGES	chr9	132502088	132502088	G	A	NA	synonymous
CEL	chr9	135946429	135946429	G	A	NA	nonsynonymous
CEL	chr9	135946449	135946449	G	C	NA	nonsynonymous
ANAPC2	chr9	140074829	140074829	G	A	NA	nonsynonymous
FAM166A	chr9	140140289	140140289	G	A	NA	nonsynonymous
ELAVL2	chr9	23731044	23731044	C	A	NA	nonsynonymous
CNTNAP3B	chr9	43844301	43844301	C	T	NA	synonymous
GNAQ	chr9	80343494	80343494	C	G	NA	nonsynonymous
SPATA31D1	chr9	84609013	84609013	T	G	NA	nonsynonymous
DAPK1	chr9	90256895	90256895	A	G	NA	synonymous
WNT8B	chr10	102242209	102242209	G	A	NA	nonsynonymous
HPS6	chr10	103825954	103825954	G	C	NA	synonymous
CFAP43	chr10	105944885	105944885	C	T	NA	nonsynonymous
MXI1	chr10	112039753	112039753	G	T	NA	stopgain
DOCK1	chr10	129055676	129055676	G	C	NA	nonsynonymous
FOXI2	chr10	129535879	129535879	C	T	NA	synonymous
NKX6-2	chr10	134598501	134598501	G	T	NA	synonymous
FAM171A1	chr10	15256023	15256023	C	T	NA	nonsynonymous
CUBN	chr10	17083111	17083111	G	C	NA	nonsynonymous
NSUN6	chr10	18940106	18940106	C	G	NA	nonsynonymous
GPR158	chr10	25887905	25887905	G	A	NA	nonsynonymous
SVIL	chr10	29754580	29754580	G	T	NA	nonsynonymous
SVIL	chr10	29839964	29839964	G	C	NA	nonsynonymous
CCDC7	chr10	33140848	33140848	C	T	NA	stopgain
LRIT1	chr10	85992543	85992543	C	T	NA	nonsynonymous
KIF20B	chr10	91497554	91497554	C	T	NA	nonsynonymous
PLCE1	chr10	95791379	95791379	A	C	NA	nonsynonymous
GUCY1A2	chr11	106579278	106579278	T	A	NA	nonsynonymous
NPAT	chr11	108040542	108040542	C	A	NA	nonsynonymous
C11orf88	chr11	111386252	111386252	G	C	NA	synonymous
KMT2A	chr11	118368740	118368740	C	T	NA	synonymous
OR10G8	chr11	123901119	123901119	A	G	NA	nonsynonymous
NFRKB	chr11	129746690	129746690	G	T	NA	nonsynonymous
SPTY2D1	chr11	18637527	18637527	C	G	NA	synonymous
PTPN5	chr11	18755142	18755142	G	A	NA	synonymous
RCN1	chr11	32125013	32125013	C	G	NA	stopgain

KIAA1549L	chr11	33596419	33596419	C	T	NA	nonsynonymous
FNBP4	chr11	47765532	47765532	G	A	NA	stopgain
FOLH1	chr11	49168365	49168365	A	C	NA	nonsynonymous
OR5AN1	chr11	59132083	59132083	G	A	NA	nonsynonymous
OR5AN1	chr11	59132195	59132195	G	A	NA	synonymous
LGALS12	chr11	63276445	63276445	C	T	NA	synonymous
PLCB3	chr11	64033616	64033616	G	A	NA	nonsynonymous
SLC22A11	chr11	64323728	64323728	G	A	NA	nonsynonymous
ATG2A	chr11	64669505	64669505	C	G	NA	nonsynonymous
KLHL35	chr11	75134846	75134846	C	A	NA	nonsynonymous
DLG2	chr11	83770507	83770507	C	T	NA	nonsynonymous
AMOTL1	chr11	94587122	94587122	G	T	NA	stopgain
AMOTL1	chr11	94587124	94587124	G	-	NA	frameshift_deletion
SBF2	chr11	9868609	9868609	C	T	NA	nonsynonymous
SETD1B	chr12	122242666	122242666	A	C	NA	nonsynonymous
SETD1B	chr12	122242666	122242666	A	-	NA	frameshift_deletion
MPHOSPH9	chr12	123648535	123648535	G	C	NA	stopgain
POLE	chr12	133226323	133226323	G	A	NA	synonymous
GOLGA3	chr12	133363283	133363283	G	C	NA	nonsynonymous
ERC1	chr12	1372211	1372211	T	G	NA	synonymous
FGFR1OP2	chr12	27109509	27109509	G	A	NA	nonsynonymous
KRT2	chr12	53045468	53045468	G	C	NA	synonymous
PPP1R1A	chr12	54975799	54975799	C	A	NA	stopgain
ERBB3	chr12	56495457	56495457	C	T	NA	nonsynonymous
LRP1	chr12	57587038	57587038	C	T	NA	synonymous
VWF	chr12	6182807	6182807	G	T	NA	stopgain
PTPRR	chr12	71050586	71050586	C	T	NA	nonsynonymous
PLXNC1	chr12	94634320	94634320	G	A	NA	nonsynonymous
COL4A1	chr13	110845223	110845223	G	A	NA	synonymous
SACS	chr13	23911781	23911781	G	C	NA	nonsynonymous
SLC7A1	chr13	30088640	30088640	C	G	NA	nonsynonymous
AHNAK2	chr14	105410651	105410651	C	T	NA	nonsynonymous
APEX1	chr14	20925283	20925283	C	G	NA	synonymous
STXBP6	chr14	25288344	25288344	T	G	NA	nonsynonymous
FOXG1	chr14	29237390	29237390	G	A	NA	nonsynonymous
PRKD1	chr14	30046530	30046530	T	A	NA	nonsynonymous
KIAA0391	chr14	35592551	35592551	G	A	NA	nonsynonymous
FANCM	chr14	45669110	45669110	C	T	NA	nonsynonymous
MIS18BP1	chr14	45686321	45686321	A	T	NA	synonymous
MDGA2	chr14	47342791	47342791	-	AACCAACCTTAA	NA	nonframeshift_insertion
SAV1	chr14	51132284	51132284	T	C	NA	nonsynonymous
CATSPERB	chr14	92139281	92139281	A	T	NA	nonsynonymous
CYFIP1	chr15	22980132	22980132	G	C	NA	nonsynonymous
HERC2	chr15	28441711	28441711	G	A	NA	synonymous
MTMR10	chr15	31260130	31260130	G	A	NA	synonymous
TTBK2	chr15	43045371	43045371	C	A	NA	nonsynonymous
UBR1	chr15	43290399	43290399	T	C	NA	nonsynonymous
DUOX1	chr15	45454140	45454140	C	T	NA	nonsynonymous
DMXL2	chr15	51750713	51750713	C	T	NA	nonsynonymous
ADPGK	chr15	73044930	73044930	C	T	NA	nonsynonymous
AKAP13	chr15	86124818	86124818	C	T	NA	synonymous
KLHL25	chr15	86311526	86311526	C	T	NA	nonsynonymous
PDILT	chr16	20396147	20396147	T	C	NA	nonsynonymous
ACSM2A	chr16	20481025	20481025	T	G	NA	nonsynonymous
CHP2	chr16	23767751	23767751	C	G	NA	nonsynonymous
HS3ST4	chr16	26147533	26147533	A	T	NA	nonsynonymous
ITGAM	chr16	31284746	31284746	C	T	NA	synonymous
CHD9	chr16	53279508	53279508	T	G	NA	nonsynonymous
CES4A	chr16	67040349	67040349	C	T	NA	synonymous
KCTD19	chr16	67325565	67325565	G	T	NA	synonymous
WWP2	chr16	69905755	69905755	C	T	NA	synonymous
PKD1L2	chr16	81204346	81204346	C	T	NA	synonymous
PLCG2	chr16	81979799	81979799	G	A	NA	synonymous
CHMP1A	chr16	89712429	89712429	C	G	NA	nonsynonymous
MNT	chr17	2298225	2298225	C	T	NA	synonymous
LOC107984974	chr17	29349047	29349047	C	T	NA	stopgain
STAT3	chr17	40489811	40489811	C	G	NA	nonsynonymous
ZFP3	chr17	4995260	4995260	C	G	NA	stopgain
HLF	chr17	53345181	53345181	C	T	NA	nonsynonymous
ANKFN1	chr17	54428142	54428142	A	C	NA	nonsynonymous
TP53	chr17	7578475	7578475	G	-	NA	frameshift_deletion
DNAH17	chr17	76433889	76433889	G	A	NA	nonsynonymous
CCDC40	chr17	78032699	78032699	G	A	NA	nonsynonymous
ZNF750	chr17	80789425	80789425	G	T	NA	stopgain
SPIRE1	chr18	12496059	12496059	C	T	NA	nonsynonymous
KIAA1328	chr18	34802146	34802146	G	A	NA	nonsynonymous
MALT1	chr18	56376717	56376717	A	T	NA	nonsynonymous
CCBE1	chr18	57103339	57103339	A	G	NA	nonsynonymous
DSEL	chr18	65179400	65179400	G	A	NA	nonsynonymous
ZNF441	chr19	11892044	11892044	C	G	NA	nonsynonymous
ZNF700	chr19	12060499	12060499	C	A	NA	nonsynonymous
MED26	chr19	16687420	16687420	G	C	NA	nonsynonymous
UNC13A	chr19	17743617	17743617	G	T	NA	nonsynonymous

SUGP1	chr19	19416662	19416662	G	C	NA	nonsynonymous
ZNF14	chr19	19822882	19822882	G	C	NA	stopgain
DPY19L3	chr19	32927454	32927454	A	G	NA	nonsynonymous
KMT2B	chr19	36221628	36221628	G	A	NA	nonsynonymous
ACTN4	chr19	39205163	39205163	G	C	NA	nonsynonymous
CCDC8	chr19	46915188	46915188	C	T	NA	nonsynonymous
BCL2L12	chr19	50172355	50172355	G	A	NA	nonsynonymous
ZNF613	chr19	52447818	52447818	G	C	NA	nonsynonymous
CHMP2A	chr19	59063048	59063048	C	T	NA	nonsynonymous
SEL1L2	chr20	13847479	13847479	T	C	NA	nonsynonymous
DZANK1	chr20	18407756	18407756	C	G	NA	synonymous
NKX2-4	chr20	21376965	21376965	G	C	NA	nonsynonymous
CST2	chr20	23807222	23807222	C	G	NA	nonsynonymous
MYH7B	chr20	33568534	33568534	C	T	NA	nonsynonymous
GDF5OS	chr20	34022687	34022687	C	G	NA	nonsynonymous
PTPRT	chr20	40713385	40713385	C	T	NA	nonsynonymous
WFDC3	chr20	44404070	44404070	G	C	NA	nonsynonymous
CABLES2	chr20	60966148	60966148	C	A	NA	nonsynonymous
SCAF4	chr21	33065693	33065693	G	C	NA	nonsynonymous
CASTOR1	chr22	30683226	30683226	G	C	NA	nonsynonymous
MYH9	chr22	36682853	36682853	G	-	NA	frameshift_deletion
EFCAB6	chr22	43996061	43996061	G	A	NA	nonsynonymous
EFCAB6	chr22	44168889	44168889	G	A	NA	synonymous
TRMU	chr22	46746238	46746238	C	G	NA	nonsynonymous
SBF1	chr22	50904396	50904396	C	T	NA	synonymous
CSTF2	chrX	100087732	100087732	G	T	NA	nonsynonymous
ALG13	chrX	110987926	110987926	C	G	NA	nonsynonymous
RBMXL3	chrX	114426992	114426992	C	T	NA	synonymous
GEMIN8	chrX	14027038	14027038	C	A	NA	nonsynonymous
DNASE1L1	chrX	153631675	153631675	C	T	NA	nonsynonymous
SMS	chrX	21995323	21995323	T	C	NA	synonymous
MAGEB18	chrX	26157248	26157248	C	G	NA	nonsynonymous
FTHL17	chrX	31089667	31089667	T	A	NA	nonsynonymous
SRPX	chrX	38037581	38037581	T	A	NA	synonymous
AKAP4	chrX	49958628	49958628	A	G	NA	nonsynonymous
MTMR8	chrX	63488633	63488633	G	A	NA	synonymous
ERCC6L	chrX	71426530	71426530	T	C	NA	nonsynonymous
PCDH11X	chrX	91133848	91133848	A	C	NA	nonsynonymous

Gene	chromosome	Start	Stop	Ref	Alt	HK4-HA	HK4-EA
KCNA10	chr1	111060126	111060126	G	T	stopgain	NA
DVL1	chr1	1277781	1277781	C	T	synonymous	NA
DUSP12	chr1	161719816	161719816	A	G	synonymous	NA
OBSCN	chr1	228412385	228412385	A	G	nonsynonymous	NA
RYR2	chr1	237729980	237729980	G	T	nonsynonymous	NA
EPB41	chr1	29314352	29314352	C	T	nonsynonymous	NA
SMAP2	chr1	40872524	40872524	A	T	nonsynonymous	NA
SPATA6	chr1	48825268	48825268	G	A	nonsynonymous	NA
RNF11	chr1	51735794	51735794	G	A	nonsynonymous	NA
BTF3L4	chr1	52549021	52549021	A	T	nonsynonymous	NA
LRRIQ3	chr1	74507442	74507442	C	A	nonsynonymous	NA
ERICH3	chr1	75102019	75102019	G	T	stopgain	NA
PTGFR	chr1	79002163	79002163	C	T	stopgain	NA
CLCA2	chr1	86913275	86913275	T	C	nonsynonymous	NA
DNTTIP2	chr1	94342122	94342122	C	A	stopgain	NA
GCC2	chr2	109098813	109098813	T	A	stopgain	NA
TTL	chr2	113260624	113260624	A	G	synonymous	NA
POTEE	chr2	132021105	132021105	G	T	nonsynonymous	NA
ZRANB3	chr2	136111063	136111063	T	A	nonsynonymous	NA
LRP1B	chr2	140992403	140992403	C	T	synonymous	NA
COBLL1	chr2	165552182	165552182	T	C	nonsynonymous	NA
PPIG	chr2	170488430	170488430	A	G	nonsynonymous	NA
TTN	chr2	179419634	179419634	C	A	nonsynonymous	NA
UNC80	chr2	210640665	210640665	T	C	nonsynonymous	NA
ABCA12	chr2	215896601	215896601	C	T	synonymous	NA
NCL	chr2	232320126	232320126	C	A	nonsynonymous	NA
RBM44	chr2	238727035	238727035	A	G	synonymous	NA
SOS1	chr2	39250200	39250200	G	C	nonsynonymous	NA
PSME4	chr2	54125478	54125478	A	C	nonsynonymous	NA
USP34	chr2	61463031	61463031	C	A	nonsynonymous	NA
XPO1	chr2	61729155	61729155	T	C	synonymous	NA
DCTN1	chr2	74598750	74598750	C	T	nonsynonymous	NA
PTCD3	chr2	86364170	86364170	A	-	frameshift_deletion	NA
ITPRIPL1	chr2	96993154	96993154	G	A	nonsynonymous	NA
EEFSEC	chr3	128060188	128060188	G	A	nonsynonymous	NA
WDR48	chr3	39116244	39116244	A	G	nonsynonymous	NA
TLR9	chr3	52255531	52255531	A	C	nonsynonymous	NA
OR5H1	chr3	97852218	97852218	-	A	frameshift_insertion	NA
TET2	chr4	106182983	106182994	CACCTGATG CAT	-	nonframeshift_deletion	NA
TBCK	chr4	107037523	107037523	T	C	nonsynonymous	NA
USP53	chr4	120214103	120214103	C	A	nonsynonymous	NA
LRBA	chr4	151827539	151827539	A	T	nonsynonymous	NA
ARAP2	chr4	36230470	36230470	G	A	synonymous	NA
FBN2	chr5	127599226	127599226	C	T	nonsynonymous	NA
IRGM	chr5	150227846	150227846	G	A	nonsynonymous	NA
ZNF879	chr5	178459227	178459227	C	G	nonsynonymous	NA
OXCT1	chr5	41861434	41861434	G	A	nonsynonymous	NA
COX7C	chr5	85913930	85913930	G	A	nonsynonymous	NA
SLF1	chr5	94030587	94030587	A	G	nonsynonymous	NA
NKAIN2	chr6	124443079	124443080	TC	-	frameshift_deletion	NA
NKAIN2	chr6	124443082	124443082	T	A	stopgain	NA
ADGB	chr6	147012388	147012388	G	A	nonsynonymous	NA
SCAF8	chr6	155123254	155123254	C	T	synonymous	NA
HIST1H4C	chr6	26104191	26104191	A	C	nonsynonymous	NA
HIST1H4C	chr6	26104196	26104196	C	G	synonymous	NA
DSP	chr6	7584761	7584773	AAATCTTAC CTAT	-	frameshift_deletion	NA
PCOLCE	chr7	100204081	100204081	C	G	synonymous	NA
SSPO	chr7	149496995	149496995	C	T	synonymous	NA
DNAJB6	chr7	157151280	157151280	A	G	nonsynonymous	NA
SDK1	chr7	4172030	4172030	G	A	synonymous	NA
GUSB	chr7	65426002	65426002	C	A	nonsynonymous	NA
PEG10	chr7	94292895	94292895	C	T	synonymous	NA
PON1	chr7	94931627	94931627	G	A	nonsynonymous	NA
CSMD3	chr8	113563045	113563045	G	C	nonsynonymous	NA
C8orf48	chr8	13424915	13424915	T	C	nonsynonymous	NA
ZFP41	chr8	144332184	144332184	A	G	synonymous	NA
ZNF517	chr8	146033750	146033750	G	A	synonymous	NA
MYOM2	chr8	1998950	1998950	C	G	nonsynonymous	NA
PNOC	chr8	28196829	28196829	C	T	synonymous	NA
INTS9	chr8	28695204	28695204	G	A	synonymous	NA
RB1CC1	chr8	53573502	53573503	GG	-	frameshift_deletion	NA
VCPIP1	chr8	67547461	67547461	C	A	nonsynonymous	NA
SLCO5A1	chr8	70744813	70744813	G	T	synonymous	NA
ZFHX4	chr8	77762586	77762586	G	T	nonsynonymous	NA
VIRMA	chr8	95523562	95523562	T	C	nonsynonymous	NA
SURF6	chr9	136201348	136201348	C	T	nonsynonymous	NA
SURF6	chr9	136201385	136201385	C	A	nonsynonymous	NA
DOCK8	chr9	376275	376275	A	T	nonsynonymous	NA
PRUNE2	chr9	79324618	79324618	T	C	nonsynonymous	NA

SEMA4G	chr10	102740975	102740975	G	T	nonsynonymous	NA
PLPP4	chr10	122348843	122348855	CATTTTTGC ATAC	-	frameshift_deletion	NA
PCDH15	chr10	55782929	55782929	C	T	nonsynonymous	NA
C10orf62	chr10	99349802	99349802	G	A	nonsynonymous	NA
ROBO4	chr11	124761230	124761230	G	A	nonsynonymous	NA
OTOG	chr11	17627547	17627547	C	T	nonsynonymous	NA
CD44	chr11	35219783	35219783	C	T	synonymous	NA
MTMR2	chr11	95595464	95595464	T	A	nonsynonymous	NA
YBX3	chr12	10854611	10854611	T	C	nonsynonymous	NA
BICDL1	chr12	120510351	120510351	C	T	nonsynonymous	NA
TMEM132I	chr12	129566364	129566364	G	A	synonymous	NA
C2CD5	chr12	22625707	22625707	G	A	nonsynonymous	NA
ALG10	chr12	34177070	34177070	C	T	synonymous	NA
DNAJC14	chr12	56217265	56217265	G	A	nonsynonymous	NA
PA2G4	chr12	56504224	56504224	A	G	nonsynonymous	NA
CDK17	chr12	96674649	96674649	T	A	nonsynonymous	NA
LRRC63	chr13	46801821	46801821	C	A	stopgain	NA
SLITRK5	chr13	88328520	88328520	G	A	nonsynonymous	NA
EXOC3L4	chr14	103571364	103571364	G	A	nonsynonymous	NA
OR11G2	chr14	20665817	20665817	A	G	nonsynonymous	NA
ARG2	chr14	68113378	68113378	-	T	frameshift_insertion	NA
GALK2	chr15	49620171	49620171	C	T	stopgain	NA
TMEM202	chr15	72690707	72690707	C	G	nonsynonymous	NA
IGF1R	chr15	99478076	99478076	G	A	nonsynonymous	NA
VWA3A	chr16	22109007	22109007	G	A	synonymous	NA
DNAJA2	chr16	46993289	46993289	T	C	nonsynonymous	NA
ANKS3	chr16	4752229	4752229	G	A	nonsynonymous	NA
SALL1	chr16	51174820	51174820	G	A	nonsynonymous	NA
FOXL1	chr16	86612860	86612860	G	A	synonymous	NA
ZNF469	chr16	88495027	88495027	A	T	nonsynonymous	NA
MPRIP	chr17	17046921	17046921	C	T	nonsynonymous	NA
SLC47A2	chr17	19618038	19618038	G	A	nonsynonymous	NA
SUPT6H	chr17	27009858	27009858	A	G	nonsynonymous	NA
RNF135	chr17	29326011	29326011	C	T	synonymous	NA
HNF1B	chr17	36099589	36099589	A	C	nonsynonymous	NA
THRA	chr17	38245694	38245694	G	A	synonymous	NA
VPS53	chr17	424905	424905	T	C	nonsynonymous	NA
MTMR4	chr17	56572445	56572445	C	T	nonsynonymous	NA
MED13	chr17	60028344	60028344	G	T	synonymous	NA
SLC16A6	chr17	66267607	66267607	G	A	stopgain	NA
TEKT1	chr17	6716368	6716368	C	T	nonsynonymous	NA
NEURL4	chr17	7231105	7231105	C	T	synonymous	NA
CD300LF	chr17	72692356	72692356	G	C	nonsynonymous	NA
SMIM6	chr17	73643572	73643572	T	C	nonsynonymous	NA
TP53	chr17	7578190	7578190	T	C	nonsynonymous	NA
TP53	chr17	7579313	7579313	G	A	nonsynonymous	NA
ESCO1	chr18	19110450	19110450	T	A	nonsynonymous	NA
GAREM1	chr18	29848674	29848674	T	C	synonymous	NA
DSEL	chr18	65181360	65181360	A	G	synonymous	NA
MRPL4	chr19	10367421	10367421	G	T	nonsynonymous	NA
TYK2	chr19	10472457	10472457	-	A	stopgain	NA
UPF1	chr19	18974321	18974321	A	G	nonsynonymous	NA
ZNF98	chr19	22574524	22574524	G	A	nonsynonymous	NA
LMNB2	chr19	2438405	2438405	C	G	nonsynonymous	NA
SLC7A10	chr19	33700267	33700267	A	-	frameshift_deletion	NA
ALKBH6	chr19	36502318	36502318	A	G	synonymous	NA
WDR87	chr19	38385290	38385290	C	A	synonymous	NA
MAP4K1	chr19	39086364	39086364	C	T	nonsynonymous	NA
FCGBP	chr19	40376857	40376857	C	A	nonsynonymous	NA
ZNF780B	chr19	40541132	40541132	G	C	nonsynonymous	NA
B9D2	chr19	41869353	41869353	C	T	synonymous	NA
SIGLEC9	chr19	51630343	51630343	C	T	nonsynonymous	NA
ZNF416	chr19	58083560	58083560	G	T	nonsynonymous	NA
MUC16	chr19	9071269	9071269	G	A	nonsynonymous	NA
FKBP1A	chr20	1350711	1350711	G	A	nonsynonymous	NA
KIF16B	chr20	16387072	16387072	T	C	nonsynonymous	NA
REM1	chr20	30064566	30064566	G	A	synonymous	NA
ZDHHC8	chr22	20127097	20127097	C	T	nonsynonymous	NA
PISD	chr22	32017458	32017458	T	C	nonsynonymous	NA
SUN2	chr22	39138494	39138494	-	AAT	nonframeshift_insertion	NA
XRCC6	chr22	42018073	42018073	A	T	stopgain	NA
IRS4	chrX	107978230	107978230	C	T	nonsynonymous	NA
CT47B1	chrX	120009290	120009290	C	T	nonsynonymous	NA
ZNF41	chrX	47307614	47307614	C	A	nonsynonymous	NA
ERCC6L	chrX	71425908	71425908	A	G	synonymous	NA
SATL1	chrX	84363376	84363376	T	A	nonsynonymous	NA
NOTCH2	chr1	120471687	120471687	G	C	NA	nonsynonymous
PFDN2	chr1	161070536	161070536	T	A	NA	synonymous
SPEN	chr1	16259304	16259304	A	G	NA	nonsynonymous
IGFN1	chr1	201181975	201181975	G	A	NA	nonsynonymous
CCNT2	chr2	135711757	135711757	T	C	NA	nonsynonymous
CDCA7	chr2	174224206	174224206	T	A	NA	nonsynonymous

NFE2L2	chr2	178098957	178098957	-	ATCTAT	NA	nonframeshift_insertion
TTN	chr2	179584926	179584926	G	A	NA	nonsynonymous
CERKL	chr2	182468785	182468785	A	T	NA	nonsynonymous
KLHL29	chr2	23926082	23926082	A	T	NA	nonsynonymous
TET3	chr2	74230273	74230273	G	A	NA	nonsynonymous
STXBP5L	chr3	120760577	120760577	C	-	NA	frameshift_deletion
SLC15A2	chr3	121615326	121615326	A	G	NA	nonsynonymous
SI	chr3	164750367	164750367	C	T	NA	synonymous
LINC02054	chr3	184157056	184157056	G	A	NA	nonsynonymous
PLXNB1	chr3	48456717	48456717	G	A	NA	synonymous
CACNA2D3	chr3	54537669	54537669	A	G	NA	nonsynonymous
ARHGEF3	chr3	56807774	56807774	T	C	NA	nonsynonymous
PRICKLE2	chr3	64085377	64085377	C	A	NA	nonsynonymous
ANXA5	chr4	122607475	122607475	G	A	NA	nonsynonymous
SLIT2	chr4	20530671	20530671	A	T	NA	nonsynonymous
RICTOR	chr5	38996922	38996922	T	C	NA	nonsynonymous
CD180	chr5	66480118	66480118	T	C	NA	nonsynonymous
MFSD4B	chr6	111588213	111588213	A	G	NA	nonsynonymous
HIST1H4E	chr6	26204990	26204990	C	A	NA	nonsynonymous
HIST1H2BL	chr6	27775646	27775646	C	G	NA	nonsynonymous
LMBRD1	chr6	70428931	70428931	T	C	NA	nonsynonymous
SRRT	chr7	100485935	100485935	-	TG	NA	frameshift_insertion
THSD7A	chr7	11675984	11675984	C	T	NA	nonsynonymous
TAS2R16	chr7	122635295	122635295	C	A	NA	nonsynonymous
GRM8	chr7	126173504	126173504	A	G	NA	synonymous
HOXA1	chr7	27134257	27134257	C	T	NA	synonymous
TNS3	chr7	47343109	47343109	G	C	NA	nonsynonymous
POM121L1	chr7	53103649	53103649	G	A	NA	synonymous
EGFR	chr7	55224288	55224288	A	T	NA	stopgain
DBF4	chr7	87536936	87536936	T	A	NA	nonsynonymous
OXR1	chr8	107715173	107715173	A	G	NA	nonsynonymous
EIF3E	chr8	109215282	109215289	CTTTTGGT	-	NA	frameshift_deletion
PRDM14	chr8	70981631	70981631	C	G	NA	synonymous
ZFHX4	chr8	77762568	77762568	G	A	NA	nonsynonymous
C9orf3	chr9	97843023	97843023	A	G	NA	synonymous
LOXL4	chr10	100017801	100017801	C	T	NA	nonsynonymous
HPSE2	chr10	100249846	100249846	G	T	NA	nonsynonymous
BCCIP	chr10	127512141	127512141	T	C	NA	synonymous
RAB18	chr10	27826968	27826968	C	T	NA	nonsynonymous
NRP1	chr10	33486629	33486629	T	G	NA	nonsynonymous
CISD1	chr10	60036932	60036932	A	G	NA	synonymous
ANK3	chr10	61828572	61828572	A	G	NA	nonsynonymous
ZNF365	chr10	64159352	64159352	G	A	NA	nonsynonymous
CHST1	chr11	45671806	45671806	G	A	NA	nonsynonymous
ESRRA	chr11	64083342	64083342	G	A	NA	synonymous
DEPDC4	chr12	100656159	100656159	G	C	NA	nonsynonymous
CLEC1A	chr12	10224035	10224035	T	C	NA	nonsynonymous
TMEM233	chr12	120067645	120067645	C	T	NA	synonymous
NCOR2	chr12	124812114	124812114	T	C	NA	nonsynonymous
NCOR2	chr12	124968198	124968198	G	C	NA	nonsynonymous
ARID2	chr12	46123683	46123683	G	T	NA	stopgain
OSBPL8	chr12	76763055	76763055	T	A	NA	nonsynonymous
FRY	chr13	32841326	32841326	G	T	NA	stopgain
C14orf93	chr14	23456456	23456456	A	T	NA	nonsynonymous
SGPP1	chr14	64153297	64153297	G	-	NA	frameshift_deletion
SYNE2	chr14	64467427	64467427	C	G	NA	nonsynonymous
VRTN	chr14	74823886	74823886	C	A	NA	nonsynonymous
YLPM1	chr14	75248572	75248572	C	G	NA	nonsynonymous
NRXN3	chr14	79181227	79181227	G	A	NA	nonsynonymous
MAGEL2	chr15	23890519	23890519	G	A	NA	nonsynonymous
OCA2	chr15	28263653	28263653	C	A	NA	nonsynonymous
SLC12A6	chr15	34532885	34532885	C	A	NA	nonsynonymous
ISLR2	chr15	74425125	74425125	C	T	NA	synonymous
ADAMTSL3	chr15	84651190	84651190	G	T	NA	nonsynonymous
VPS33B	chr15	91553040	91553040	T	C	NA	nonsynonymous
MPG	chr16	129453	129453	G	A	NA	synonymous
NQO1	chr16	69745031	69745031	G	A	NA	nonsynonymous
PDPR	chr16	70177485	70177485	A	G	NA	nonsynonymous
ATAD5	chr17	29220736	29220736	C	A	NA	nonsynonymous
UBTF	chr17	42288274	42288274	G	T	NA	nonsynonymous
TP53	chr17	7578536	7578536	T	C	NA	nonsynonymous
ZNF750	chr17	80790264	80790264	A	G	NA	nonsynonymous
CEP192	chr18	13100428	13100428	C	G	NA	nonsynonymous
ANKRD30B	chr18	14852410	14852410	A	T	NA	synonymous
CDH7	chr18	63511064	63511064	C	A	NA	nonsynonymous
CD22	chr19	35829173	35829175	ATT	-	NA	nonframeshift_deletion
GGN	chr19	38876381	38876381	G	A	NA	synonymous
ZNF841	chr19	52569133	52569133	G	C	NA	nonsynonymous
KIR2DL1	chr19	55284785	55284785	G	T	NA	nonsynonymous
ZNF557	chr19	7075688	7075688	C	G	NA	synonymous
EPB41L1	chr20	34797571	34797571	C	T	NA	synonymous
STAU1	chr20	47774992	47774992	T	C	NA	nonsynonymous
SCAF4	chr21	33044073	33044073	C	T	NA	nonsynonymous

GGT5	chr22	24628983	24628983	G	A	NA	nonsynonymous
CENPI	chrX	100383786	100383786	C	T	NA	synonymous
ATP2B3	chrX	152801886	152801886	C	T	NA	nonsynonymous

Gene	chromosome	Start	Stop	Ref	Alt	HK5-EA	HK5-EB	HK5-EC	HK5-ED
AKAP13	chr15	86284304	86284304	A	G	nonsynonymous	NA	nonsynonymous	nonsynonymous
ANGPTL2	chr9	129856058	129856058	C	A	nonsynonymous	NA	nonsynonymous	nonsynonymous
ANKRD65	chr1	1354681	1354681	C	A	nonsynonymous	NA	NA	NA
ARHGAP24	chr4	86916800	86916800	A	G	nonsynonymous	NA	nonsynonymous	nonsynonymous
ARHGAP45	chr19	1077811	1077811	C	A	nonsynonymous	NA	NA	nonsynonymous
ARL14EP	chr11	30358143	30358143	A	G	nonsynonymous	NA	nonsynonymous	nonsynonymous
ASNS	chr7	97493720	97493720	G	T	nonsynonymous	NA	NA	NA
ATG2A	chr11	64665764	64665764	G	A	nonsynonymous	NA	NA	nonsynonymous
ATN1	chr12	7047770	7047770	G	A	nonsynonymous	NA	NA	NA
ATN1	chr12	7050121	7050121	C	G	nonsynonymous	NA	nonsynonymous	nonsynonymous
ATP9B	chr18	76973962	76973962	G	T	nonsynonymous	NA	nonsynonymous	nonsynonymous
C19orf54	chr19	41255440	41255440	G	A	nonsynonymous	NA	nonsynonymous	nonsynonymous
CACNA1G	chr17	48696040	48696040	G	A	nonsynonymous	NA	NA	nonsynonymous
CC2D1B	chr1	52819295	52819295	C	T	nonsynonymous	NA	NA	NA
CCDC80	chr3	112358569	112358569	C	T	nonsynonymous	NA	NA	NA
CD1A	chr1	158225119	158225119	G	A	nonsynonymous	NA	NA	NA
CHD7	chr8	61769272	61769272	T	G	nonsynonymous	NA	NA	NA
CHD9	chr16	53190285	53190285	C	G	nonsynonymous	NA	nonsynonymous	nonsynonymous
CLVS1	chr8	62212702	62212702	G	C	nonsynonymous	NA	nonsynonymous	nonsynonymous
CNTNAP2	chr7	147600716	147600716	G	T	nonsynonymous	NA	NA	NA
COL8A2	chr1	36563449	36563449	G	C	nonsynonymous	NA	NA	NA
CORO1A	chr16	30199303	30199303	C	T	nonsynonymous	NA	NA	nonsynonymous
COX20	chr1	245006410	245006412	AAG	-	nonframeshift_deletion	NA	nonframeshift_deletion	nonframeshift_deletion
DAPK1	chr9	90255217	90255217	A	G	nonsynonymous	NA	NA	NA
DCLRE1A	chr10	115596922	115596922	T	C	nonsynonymous	NA	nonsynonymous	NA
DDX23	chr12	49227221	49227221	G	T	nonsynonymous	NA	nonsynonymous	nonsynonymous
DDX51	chr12	132625384	132625384	C	A	nonsynonymous	NA	NA	NA
DDX59	chr1	200628236	200628236	-	A	frameshift_insertion	NA	frameshift_insertion	NA
E2F7	chr12	77419544	77419544	C	T	nonsynonymous	NA	nonsynonymous	nonsynonymous
ELP4	chr11	31653842	31653842	G	T	stopgain	NA	stopgain	stopgain
EPB41L3	chr18	5438096	5438096	G	C	nonsynonymous	NA	nonsynonymous	nonsynonymous
ERG	chr21	39775497	39775497	C	T	nonsynonymous	NA	nonsynonymous	nonsynonymous
FAM200B	chr4	15689013	15689013	G	-	frameshift_deletion	NA	frameshift_deletion	frameshift_deletion
FAT2	chr5	150908926	150908926	T	A	nonsynonymous	NA	NA	NA
FITM2	chr20	42935608	42935608	C	T	nonsynonymous	NA	nonsynonymous	nonsynonymous
FZD2	chr17	42635502	42635502	T	C	nonsynonymous	NA	nonsynonymous	nonsynonymous
GABRB3	chr15	26806239	26806239	T	C	nonsynonymous	NA	nonsynonymous	nonsynonymous
GRIA1	chr5	153174193	153174193	C	A	nonsynonymous	NA	NA	NA
GSTM1	chr1	110231897	110231897	A	G	nonsynonymous	NA	NA	NA
HNRNPAB	chr5	177632991	177632991	-	A	frameshift_insertion	NA	frameshift_insertion	frameshift_insertion
ITGB5	chr3	124578107	124578107	C	A	nonsynonymous	NA	nonsynonymous	nonsynonymous
ITGB8	chr7	20406803	20406803	C	T	nonsynonymous	NA	nonsynonymous	nonsynonymous
KALRN	chr3	124181405	124181405	A	T	nonsynonymous	NA	nonsynonymous	nonsynonymous
KANK3	chr19	8398974	8398974	G	A	nonsynonymous	NA	nonsynonymous	nonsynonymous
KDM5D	chrY	21871569	21871569	C	T	nonsynonymous	NA	NA	NA
KIF2B	chr17	51901985	51901985	C	G	nonsynonymous	NA	nonsynonymous	nonsynonymous
KREMEN1	chr22	29536331	29536331	G	C	nonsynonymous	NA	NA	NA
KRT37	chr17	39580622	39580622	C	T	nonsynonymous	NA	nonsynonymous	nonsynonymous
LCAT	chr16	67976419	67976419	-	A	frameshift_insertion	NA	frameshift_insertion	frameshift_insertion
MACF1	chr1	39750988	39750988	A	G	nonsynonymous	NA	nonsynonymous	nonsynonymous
MDGA2	chr14	47770694	47770694	A	G	nonsynonymous	NA	nonsynonymous	nonsynonymous
MIS18BP1	chr14	45693530	45693530	T	C	nonsynonymous	NA	nonsynonymous	nonsynonymous
N4BP1	chr16	48577002	48577002	G	C	nonsynonymous	NA	NA	nonsynonymous
NFE2L2	chr2	178096202	178096202	C	G	nonsynonymous	NA	NA	nonsynonymous
NLGN4X	chrX	5811049	5811049	G	C	nonsynonymous	NA	NA	NA
NRAP	chr10	115422488	115422488	G	A	nonsynonymous	NA	NA	NA
NUMB	chr14	73743590	73743590	T	C	nonsynonymous	NA	nonsynonymous	nonsynonymous
NUP210L	chr1	153998056	153998056	G	T	nonsynonymous	NA	NA	NA
OR4D10	chr11	59245425	59245425	G	C	nonsynonymous	NA	NA	NA
OR5AN1	chr11	59132603	59132603	C	G	nonsynonymous	NA	nonsynonymous	nonsynonymous
OR5D16	chr11	55606277	55606277	T	A	stopgain	NA	stopgain	stopgain
OR5H14	chr3	97868798	97868798	C	T	nonsynonymous	NA	NA	NA
OR8B2	chr11	124252330	124252330	G	C	nonsynonymous	NA	nonsynonymous	nonsynonymous
ORC3	chr6	88331102	88331102	C	A	nonsynonymous	NA	NA	NA
PAX3	chr2	223163288	223163288	G	A	nonsynonymous	NA	NA	NA
PCDH9	chr13	67802561	67802561	C	A	nonsynonymous	NA	nonsynonymous	nonsynonymous
PCSK5	chr9	78953182	78953182	G	T	nonsynonymous	NA	nonsynonymous	nonsynonymous
PHIP	chr6	79725484	79725484	T	C	nonsynonymous	NA	nonsynonymous	nonsynonymous
PHYHD1	chr9	131689326	131689326	G	A	nonsynonymous	NA	NA	nonsynonymous
PIEZO1	chr16	88791836	88791836	C	T	nonsynonymous	NA	nonsynonymous	nonsynonymous
PLEKHG3	chr14	65198436	65198436	C	T	nonsynonymous	NA	nonsynonymous	nonsynonymous
PLRG1	chr4	155461768	155461768	C	G	nonsynonymous	NA	NA	NA
PLXNA3	chrX	153699953	153699953	A	G	nonsynonymous	NA	nonsynonymous	nonsynonymous
POLR1A	chr2	86265944	86265944	T	C	nonsynonymous	NA	nonsynonymous	nonsynonymous
POSTN	chr13	38158232	38158232	C	T	nonsynonymous	NA	NA	nonsynonymous
PTH1R	chr3	46944241	46944241	-	T	frameshift_insertion	NA	NA	frameshift_insertion
RAB3B	chr1	52403066	52403066	G	A	nonsynonymous	NA	nonsynonymous	nonsynonymous
RAPGEF4	chr2	173891392	173891392	T	G	nonsynonymous	NA	NA	nonsynonymous
RASGRF1	chr15	79382678	79382678	C	T	nonsynonymous	NA	NA	nonsynonymous
RBM41	chrX	106331784	106331784	T	A	nonsynonymous	NA	nonsynonymous	nonsynonymous
RIC1	chr9	5747368	5747368	C	T	stopgain	NA	stopgain	stopgain
RNF25	chr2	219532672	219532672	-	CCTTGGC CACGTGG CCCAGCA	nonframeshift_insertion	NA	nonframeshift_insertion	nonframeshift_insertion

RPL10L	chr14	47120901	47120901	C	G	nonsynonymous	NA	NA	NA
RYR2	chr1	237540641	237540641	C	T	nonsynonymous	NA	nonsynonymous	nonsynonymous
SLC12A6	chr15	34528328	34528328	C	A	stopgain	NA	stopgain	stopgain
SLC6A15	chr12	85264404	85264404	T	A	nonsynonymous	NA	nonsynonymous	nonsynonymous
SLC7A5	chr16	87902822	87902822	G	T	nonsynonymous	NA	NA	NA
SLIT2	chr4	20599945	20599945	G	A	nonsynonymous	NA	nonsynonymous	nonsynonymous
SRPK1	chr6	35842086	35842086	T	C	nonsynonymous	NA	nonsynonymous	nonsynonymous
TAOK2	chr16	29997904	29997904	A	G	nonsynonymous	NA	nonsynonymous	nonsynonymous
TCERG1L	chr10	132965084	132965084	G	T	nonsynonymous	NA	NA	NA
TFB2M	chr1	246714570	246714570	T	C	nonsynonymous	NA	NA	NA
TNRC6B	chr22	40661209	40661209	G	T	nonsynonymous	NA	NA	nonsynonymous
TNS3	chr7	47384421	47384421	C	T	nonsynonymous	NA	nonsynonymous	nonsynonymous
TP53	chr17	7577518	7577518	T	A	nonsynonymous	NA	nonsynonymous	nonsynonymous
TRA2A	chr7	23552697	23552697	T	C	nonsynonymous	NA	nonsynonymous	nonsynonymous
TRAPPC8	chr18	29446844	29446844	T	C	nonsynonymous	NA	nonsynonymous	nonsynonymous
TRMT112	chr11	64084761	64084761	G	A	nonsynonymous	NA	NA	nonsynonymous
UBE2O	chr17	74387542	74387542	-	G	frameshift_insertion	NA	NA	NA
URGCP	chr7	43916595	43916595	G	A	nonsynonymous	NA	nonsynonymous	nonsynonymous
URI1	chr19	30498379	30498379	A	G	nonsynonymous	NA	nonsynonymous	nonsynonymous
WDFY4	chr10	50038885	50038885	C	A	nonsynonymous	NA	NA	NA
WDR49	chr3	167223191	167223191	C	T	nonsynonymous	NA	NA	NA
WDR75	chr2	190340063	190340063	A	G	nonsynonymous	NA	NA	nonsynonymous
XIRP2	chr2	168106979	168106979	A	T	nonsynonymous	NA	NA	NA
ZBTB1	chr14	64988415	64988415	C	A	nonsynonymous	NA	nonsynonymous	nonsynonymous
ZC3H15	chr2	187368911	187368911	A	C	nonsynonymous	NA	nonsynonymous	nonsynonymous
ZCCHC14	chr16	87448037	87448037	C	T	nonsynonymous	NA	NA	NA
ZIC4	chr3	147113965	147113965	C	A	nonsynonymous	NA	nonsynonymous	nonsynonymous
ZNF254	chr19	24309267	24309267	A	T	nonsynonymous	NA	nonsynonymous	nonsynonymous
ZNF460	chr19	57802550	57802550	A	G	nonsynonymous	NA	nonsynonymous	nonsynonymous
ACACA	chr17	35583309	35583309	G	T	NA	nonsynonymous	NA	NA
ACTG1	chr17	79478363	79478363	T	C	NA	nonsynonymous	NA	NA
ADCYAP1R1	chr7	31139765	31139765	C	A	NA	stopgain	NA	NA
ALG12	chr22	50301507	50301507	T	A	NA	nonsynonymous	NA	NA
ANKRD36	chr2	97792811	97792811	G	T	NA	nonsynonymous	NA	NA
ARHGAP36	chrX	130220349	130220349	C	T	NA	nonsynonymous	NA	NA
ARL14EP	chr11	30354503	30354503	A	G	NA	nonsynonymous	NA	NA
ATP13A3	chr3	194180573	194180573	G	T	NA	nonsynonymous	NA	NA
BRINP2	chr1	177249922	177249922	G	A	NA	nonsynonymous	NA	NA
C14orf132	chr14	96505846	96505846	C	-	NA	frameshift_deletion	NA	NA
C2CD5	chr12	22635628	22635628	T	C	NA	nonsynonymous	NA	NA
C2orf71	chr2	29295922	29295922	G	C	NA	nonsynonymous	NA	NA
CA2	chr8	86377560	86377560	G	T	NA	nonsynonymous	NA	NA
CABIN1	chr22	24459425	24459425	C	A	NA	nonsynonymous	NA	NA
CAMKK2	chr12	121686497	121686497	G	A	NA	nonsynonymous	NA	NA
CCDC39	chr3	180337101	180337101	A	T	NA	nonsynonymous	NA	NA
CCSER2	chr10	86273764	86273764	A	T	NA	nonsynonymous	NA	NA
CCT5	chr5	10254797	10254797	A	G	NA	nonsynonymous	NA	NA
CDC6	chr17	38447534	38447534	G	C	NA	nonsynonymous	NA	NA
CDC73	chr1	193218944	193218944	A	G	NA	nonsynonymous	NA	NA
CFAP54	chr12	96974430	96974430	G	T	NA	nonsynonymous	NA	NA
CHPF	chr2	220405192	220405192	A	T	NA	nonsynonymous	NA	NA
CKAP5	chr11	46829610	46829610	A	-	NA	frameshift_deletion	NA	NA
COBLL1	chr2	165551317	165551317	G	C	NA	nonsynonymous	NA	NA
COX10	chr17	14110417	14110417	A	T	NA	nonsynonymous	NA	NA
CRB1	chr1	197390684	197390684	G	C	NA	nonsynonymous	NA	NA
CSMD3	chr8	113569117	113569117	C	T	NA	nonsynonymous	NA	NA
CTNNA2	chr2	80773083	80773083	C	A	NA	nonsynonymous	NA	NA
DAAM1	chr14	59730299	59730299	A	G	NA	nonsynonymous	NA	NA
DENND3	chr8	142151379	142151379	C	T	NA	nonsynonymous	NA	NA
DNAJC13	chr3	132231896	132231896	C	G	NA	nonsynonymous	NA	NA
DSEL	chr18	65180185	65180185	C	A	NA	nonsynonymous	NA	NA
DYNC2H1	chr11	103191890	103191890	T	C	NA	nonsynonymous	NA	NA
EAF2	chr3	121575872	121575872	G	C	NA	nonsynonymous	NA	NA
ENPP1	chr6	132171189	132171189	G	A	NA	nonsynonymous	NA	NA
ENPP7	chr17	77705055	77705055	C	A	NA	nonsynonymous	NA	NA
EP300	chr22	41565575	41565575	A	G	NA	nonsynonymous	NA	NA
EPS15L1	chr19	16552734	16552734	T	C	NA	nonsynonymous	NA	NA
ERCC6L	chrX	71427436	71427436	A	T	NA	nonsynonymous	NA	NA
ERLIN1	chr10	101943556	101943556	T	C	NA	nonsynonymous	NA	NA
ETV1	chr7	13946177	13946177	G	T	NA	nonsynonymous	NA	NA
FAHD2B	chr2	97756061	97756061	C	T	NA	nonsynonymous	NA	NA
FAT1	chr4	187532929	187532929	C	A	NA	nonsynonymous	NA	NA
FSHB	chr11	30255301	30255301	G	A	NA	nonsynonymous	NA	NA
GDPD1	chr17	57351134	57351134	G	C	NA	stoploss	NA	NA
GEMIN5	chr5	154287307	154287307	G	C	NA	nonsynonymous	NA	NA
GLCCI1	chr7	8110706	8110706	A	C	NA	nonsynonymous	NA	NA
GLG1	chr16	74640688	74640688	C	A	NA	nonsynonymous	NA	NA
GPR34	chrX	41555513	41555513	T	A	NA	nonsynonymous	NA	NA
GTPBP3	chr19	17452487	17452530	TCCAGGAC	-	NA	frameshift_deletion	NA	NA
GTSF1L	chr20	42355275	42355275	G	T	NA	nonsynonymous	NA	NA
HECTD2	chr10	93221069	93221069	C	G	NA	nonsynonymous	NA	NA
HIST2H2AC	chr1	149858562	149858562	C	G	NA	nonsynonymous	NA	NA
HMCN1	chr1	186031720	186031720	T	A	NA	nonsynonymous	NA	NA
HPCAL1	chr2	10563148	10563148	G	A	NA	nonsynonymous	NA	NA
IGF2BP1	chr17	47118744	47118744	G	A	NA	nonsynonymous	NA	NA
IMPG1	chr6	76744450	76744450	T	C	NA	nonsynonymous	NA	NA
IPO8	chr12	30829843	30829843	A	C	NA	nonsynonymous	NA	NA

IQCH	chr15	67692631	67692631	G	C	NA	nonsynonymous	NA	NA
ITPR2	chr12	26553182	26553182	T	A	NA	nonsynonymous	NA	NA
KCNJ13	chr2	233633071	233633071	G	A	NA	stopgain	NA	NA
KCNK9	chr8	140630973	140630973	G	A	NA	nonsynonymous	NA	NA
KCNMB4	chr12	70760602	70760602	G	C	NA	nonsynonymous	NA	NA
KCNQ2	chr20	62039804	62039804	G	A	NA	nonsynonymous	NA	NA
KDM6A	chrX	44922716	44922716	G	A	NA	nonsynonymous	NA	NA
KIF1B	chr1	10332348	10332348	C	A	NA	stopgain	NA	NA
KIR3DL2	chr19	55377290	55377290	G	T	NA	nonsynonymous	NA	NA
KRT6C	chr12	52865280	52865280	-	A	NA	frameshift_insertion	NA	NA
KSR1	chr17	25924349	25924351	CCT	-	NA	nonframeshift_deletion	NA	NA
KY	chr3	134322857	134322857	C	T	NA	nonsynonymous	NA	NA
LRP1	chr12	57550054	57550054	C	T	NA	nonsynonymous	NA	NA
LRP1B	chr2	141946110	141946110	T	A	NA	nonsynonymous	NA	NA
LRRC2	chr3	46563093	46563093	C	A	NA	stopgain	NA	NA
LRRC8E	chr19	7964590	7964590	C	A	NA	nonsynonymous	NA	NA
MALRD1	chr10	19498571	19498571	A	T	NA	nonsynonymous	NA	NA
MAMDC2	chr9	72741095	72741095	T	A	NA	nonsynonymous	NA	NA
MAP3K3	chr17	61744420	61744423	AGTG	-	NA	frameshift_deletion	NA	NA
MED24	chr17	38179577	38179578	TT	-	NA	frameshift_deletion	NA	NA
METTL3	chr14	21971323	21971323	C	T	NA	nonsynonymous	NA	NA
MIB1	chr18	19379811	19379811	C	T	NA	nonsynonymous	NA	NA
MNDA	chr1	158811985	158811985	T	G	NA	nonsynonymous	NA	NA
MSI1	chr12	120805866	120805866	T	A	NA	nonsynonymous	NA	NA
MUC17	chr7	100679075	100679075	A	T	NA	nonsynonymous	NA	NA
MUC5B	chr11	1276349	1276349	A	T	NA	nonsynonymous	NA	NA
MYLK2	chr20	30408157	30408157	C	T	NA	nonsynonymous	NA	NA
MYO19	chr17	34869324	34869324	T	A	NA	nonsynonymous	NA	NA
NEB	chr2	152511888	152511888	G	A	NA	nonsynonymous	NA	NA
NEDD4	chr15	56208640	56208679	ATTATCGA	-	NA	frameshift_deletion	NA	NA
NFKBIB	chr19	39390789	39390789	G	T	NA	nonsynonymous	NA	NA
NKX2-8	chr14	37050484	37050484	C	A	NA	stopgain	NA	NA
NOA1	chr4	57834617	57834617	C	T	NA	nonsynonymous	NA	NA
NSD1	chr5	176721001	176721001	A	G	NA	nonsynonymous	NA	NA
OR4C3	chr11	48346689	48346689	G	A	NA	nonsynonymous	NA	NA
ORC3	chr6	88376829	88376829	G	C	NA	nonsynonymous	NA	NA
PCDHA13	chr5	140263477	140263477	G	A	NA	nonsynonymous	NA	NA
PCDHB7	chr5	140554672	140554672	C	G	NA	nonsynonymous	NA	NA
PCSK5	chr9	78804630	78804630	G	C	NA	nonsynonymous	NA	NA
PEAK1	chr15	77474047	77474047	C	G	NA	nonsynonymous	NA	NA
PFKFB3	chr10	6262731	6262731	A	C	NA	nonsynonymous	NA	NA
PKHD1	chr6	51892648	51892648	G	T	NA	nonsynonymous	NA	NA
PLEKHG1	chr6	151152814	151152814	G	A	NA	nonsynonymous	NA	NA
PNLIP	chr10	118314991	118314991	G	C	NA	nonsynonymous	NA	NA
PPP3R1	chr2	68415791	68415791	C	A	NA	nonsynonymous	NA	NA
PPTC7	chr12	110983763	110983763	T	A	NA	nonsynonymous	NA	NA
PRDM9	chr5	23522903	23522903	C	-	NA	frameshift_deletion	NA	NA
PTER	chr10	16553049	16553049	C	T	NA	nonsynonymous	NA	NA
PTPRE	chr10	129864393	129864393	C	T	NA	nonsynonymous	NA	NA
RAB11FIP3	chr16	570486	570486	G	A	NA	nonsynonymous	NA	NA
RALGAPA2	chr20	20582482	20582482	G	C	NA	nonsynonymous	NA	NA
RB1	chr13	48923105	48923105	A	G	NA	nonsynonymous	NA	NA
RCAN2	chr6	46190965	46190965	T	C	NA	nonsynonymous	NA	NA
RILPL2	chr12	123920915	123920915	T	A	NA	nonsynonymous	NA	NA
ROM1	chr11	62381951	62381951	T	G	NA	nonsynonymous	NA	NA
RORA	chr15	60803539	60803539	T	C	NA	nonsynonymous	NA	NA
RPS6KA1	chr1	26900645	26900645	-	T	NA	frameshift_insertion	NA	NA
RTN4RL1	chr17	1840535	1840535	C	T	NA	nonsynonymous	NA	NA
RYR1	chr19	38945908	38945908	C	T	NA	nonsynonymous	NA	NA
SCARB2	chr4	77097638	77097638	-	C	NA	frameshift_insertion	NA	NA
SEL1L3	chr4	25792129	25792129	T	A	NA	nonsynonymous	NA	NA
SEMA6D	chr15	48062894	48062894	G	C	NA	nonsynonymous	NA	NA
SEMG2	chr20	43850396	43850419	TGGACAAA	-	NA	nonframeshift_deletion	NA	NA
SETD5	chr3	9495552	9495552	G	C	NA	nonsynonymous	NA	NA
SFI1	chr22	32014114	32014114	-	A	NA	frameshift_insertion	NA	NA
SI	chr3	164755782	164755782	C	G	NA	nonsynonymous	NA	NA
SLAMF1	chr1	160604554	160604554	G	T	NA	nonsynonymous	NA	NA
SLC19A3	chr2	228563579	228563579	C	G	NA	nonsynonymous	NA	NA
SMARCAD1	chr4	95173787	95173787	A	G	NA	nonsynonymous	NA	NA
SNTB1	chr8	121551187	121551187	G	A	NA	nonsynonymous	NA	NA
SORCS2	chr4	7668879	7668879	A	G	NA	nonsynonymous	NA	NA
SP7	chr12	53722793	53722793	C	A	NA	nonsynonymous	NA	NA
SPACA1	chr6	88769192	88769192	G	T	NA	nonsynonymous	NA	NA
SPEN	chr1	16256755	16256755	G	A	NA	stopgain	NA	NA
SPTB	chr14	65239488	65239488	G	A	NA	nonsynonymous	NA	NA
STAC2	chr17	37370506	37370506	C	-	NA	frameshift_deletion	NA	NA
TAF5	chr10	105138291	105138291	A	T	NA	nonsynonymous	NA	NA
TCERG1	chr5	145858200	145858200	G	T	NA	nonsynonymous	NA	NA
TF	chr3	133496071	133496071	G	T	NA	nonsynonymous	NA	NA
THSD7A	chr7	11630198	11630198	G	A	NA	nonsynonymous	NA	NA
THUMPD3	chr3	9408596	9408596	A	C	NA	nonsynonymous	NA	NA
TMC5	chr16	19471628	19471628	A	T	NA	nonsynonymous	NA	NA
TMEM151A	chr11	66062303	66062303	T	A	NA	nonsynonymous	NA	NA
TNKS1BP1	chr11	57068457	57068457	G	A	NA	nonsynonymous	NA	NA
TNKS1BP1	chr11	57070159	57070159	C	A	NA	nonsynonymous	NA	NA
TOX2	chr20	42680184	42680184	A	T	NA	nonsynonymous	NA	NA
TP53	chr17	7578535	7578535	T	C	NA	nonsynonymous	NA	NA

TP63	chr3	189526215	189526233	CTCCATCA(	-	NA	frameshift_deletion	NA	NA
TRIM23	chr5	64907632	64907632	G	T	NA	nonsynonymous	NA	NA
TTN	chr2	179517809	179517809	G	A	NA	nonsynonymous	NA	NA
TUBA3D	chr2	132236889	132236889	C	T	NA	nonsynonymous	NA	NA
TXNDC9	chr2	99949506	99949506	C	-	NA	frameshift_deletion	NA	NA
UBR1	chr15	43347090	43347090	T	A	NA	nonsynonymous	NA	NA
UBXN4	chr2	136527355	136527355	G	C	NA	nonsynonymous	NA	NA
UGP2	chr2	64112985	64112985	-	T	NA	frameshift_insertion	NA	NA
URGCP	chr7	43918114	43918114	C	A	NA	nonsynonymous	NA	NA
UTP20	chr12	101732648	101732648	A	G	NA	nonsynonymous	NA	NA
VWA8	chr13	42144650	42144650	T	G	NA	nonsynonymous	NA	NA
WARS2	chr1	119576778	119576778	G	C	NA	nonsynonymous	NA	NA
ZDBF2	chr2	207170049	207170049	C	T	NA	nonsynonymous	NA	NA
ZFC3H1	chr12	72029249	72029249	T	C	NA	nonsynonymous	NA	NA
ZFYVE1	chr14	73490751	73490751	C	T	NA	nonsynonymous	NA	NA
ZNF148	chr3	124952602	124952602	T	G	NA	nonsynonymous	NA	NA
ZNF229	chr19	44933208	44933208	C	T	NA	nonsynonymous	NA	NA
ZNF414	chr19	8576780	8576780	C	T	NA	nonsynonymous	NA	NA
ZNF480	chr19	52817414	52817420	AACATTC	-	NA	frameshift_deletion	NA	NA
ZNF521	chr18	22806134	22806134	T	A	NA	nonsynonymous	NA	NA
ZNF560	chr19	9579835	9579835	C	-	NA	frameshift_deletion	NA	NA
ZNF669	chr1	247263743	247263743	T	A	NA	nonsynonymous	NA	NA
ZNF699	chr19	9413132	9413132	A	G	NA	nonsynonymous	NA	NA
ZNFX1	chr20	47892327	47892327	G	T	NA	nonsynonymous	NA	NA
ZSCAN5A	chr19	56733415	56733415	G	C	NA	nonsynonymous	NA	NA
ACADM	chr1	76200503	76200503	G	C	NA	NA	nonsynonymous	nonsynonymous
ANK2	chr4	114276955	114276955	G	A	NA	NA	nonsynonymous	NA
ANKRD30A	chr10	37414905	37414905	A	G	NA	NA	nonsynonymous	NA
AP3D1	chr19	2118752	2118752	C	T	NA	NA	nonsynonymous	NA
ARMH1	chr1	45191065	45191065	C	T	NA	NA	nonsynonymous	nonsynonymous
CDC73	chr1	193121519	193121519	G	A	NA	NA	nonsynonymous	NA
CFAP46	chr10	134659596	134659596	C	T	NA	NA	nonsynonymous	NA
DNAH10	chr12	124362283	124362283	A	T	NA	NA	nonsynonymous	NA
EPHA5	chr4	66467410	66467410	C	G	NA	NA	nonsynonymous	NA
GABRB3	chr15	26793043	26793043	G	A	NA	NA	nonsynonymous	nonsynonymous
GRIPAP1	chrX	48847495	48847495	G	A	NA	NA	nonsynonymous	NA
HCN4	chr15	73636081	73636081	G	C	NA	NA	nonsynonymous	NA
HOXB8	chr17	46692064	46692064	C	G	NA	NA	nonsynonymous	NA
KCNH7	chr2	163253451	163253451	T	G	NA	NA	nonsynonymous	NA
KCNMB1	chr5	169812398	169812398	G	T	NA	NA	stopgain	NA
KIF1B	chr1	10327522	10327522	C	T	NA	NA	nonsynonymous	NA
MAP3K19	chr2	135782215	135782215	C	T	NA	NA	nonsynonymous	NA
NCOR2	chr12	124821557	124821557	C	A	NA	NA	nonsynonymous	nonsynonymous
NEB	chr2	152512702	152512702	C	G	NA	NA	nonsynonymous	NA
OR5B3	chr11	58170054	58170054	T	C	NA	NA	nonsynonymous	NA
PEX12	chr17	33904182	33904182	T	G	NA	NA	nonsynonymous	NA
SP3	chr2	174777909	174777909	C	T	NA	NA	nonsynonymous	NA
SPEN	chr1	16245903	16245903	G	A	NA	NA	nonsynonymous	NA
TELO2	chr16	1551493	1551493	G	A	NA	NA	nonsynonymous	NA
TMEM259	chr19	1014285	1014285	G	A	NA	NA	nonsynonymous	NA
USP53	chr4	120192457	120192457	T	G	NA	NA	nonsynonymous	NA
VPS13C	chr15	62182467	62182467	T	C	NA	NA	nonsynonymous	NA
XPO7	chr8	21842203	21842203	C	T	NA	NA	stopgain	NA
YIPF6	chrX	67731790	67731790	A	G	NA	NA	nonsynonymous	NA
ZNRF4	chr19	5456775	5456775	G	A	NA	NA	nonsynonymous	NA
ATXN3	chr14	92549555	92549555	C	T	NA	NA	NA	nonsynonymous
C3orf33	chr3	155520326	155520326	G	A	NA	NA	NA	stopgain
CKMT1B	chr15	43891115	43891115	G	C	NA	NA	NA	nonsynonymous
CLEC16A	chr16	11065040	11065040	G	A	NA	NA	NA	nonsynonymous
DLC1	chr8	12957404	12957404	C	-	NA	NA	NA	frameshift_deletion
DLC1	chr8	12957405	12957405	T	A	NA	NA	NA	nonsynonymous
EDEM3	chr1	184679626	184679626	C	A	NA	NA	NA	nonsynonymous
HERC1	chr15	63908008	63908008	C	A	NA	NA	NA	stopgain
HKR1	chr19	37853655	37853655	C	T	NA	NA	NA	stopgain
HOOK2	chr19	12875822	12875822	C	T	NA	NA	NA	nonsynonymous
KCNB1	chr20	48098549	48098549	C	A	NA	NA	NA	stopgain
LRRTM4	chr2	77745572	77745572	T	A	NA	NA	NA	stopgain
ROBO2	chr3	77645803	77645803	C	T	NA	NA	NA	nonsynonymous
SF1	chr11	64535043	64535043	C	T	NA	NA	NA	nonsynonymous
SKOR1	chr15	68114365	68114365	G	A	NA	NA	NA	nonsynonymous
SLC39A11	chr17	70643772	70643772	G	C	NA	NA	NA	nonsynonymous
TAS2R46	chr12	11214290	11214290	G	C	NA	NA	NA	nonsynonymous
ZNF469	chr16	88498497	88498497	C	T	NA	NA	NA	nonsynonymous

Gene	chromosome	Start	Stop	Ref	Alt	HK6-TB	HK6-TD	HK6-TE
ADAMTS13	chr9	136295066	136295066	C	T	nonsynonymous	nonsynonymous	NA
ADAMTS20	chr12	43925941	43925941	C	A	nonsynonymous	nonsynonymous	nonsynonymous
ADGRB1	chr8	143603395	143603395	G	A	nonsynonymous	nonsynonymous	nonsynonymous
AHSG	chr3	186330968	186330968	T	C	nonsynonymous	nonsynonymous	nonsynonymous
ATP8B2	chr1	154317146	154317146	C	T	nonsynonymous	nonsynonymous	nonsynonymous
BRD7	chr16	50357538	50357538	G	A	nonsynonymous	nonsynonymous	nonsynonymous
CABLES2	chr20	60968556	60968556	G	C	nonsynonymous	nonsynonymous	nonsynonymous
CASS4	chr20	55027965	55027965	C	T	nonsynonymous	nonsynonymous	nonsynonymous
CCDC39	chr3	180381700	180381700	C	T	nonsynonymous	nonsynonymous	nonsynonymous
CHL1	chr3	423871	423871	A	T	nonsynonymous	nonsynonymous	nonsynonymous
CHSY3	chr5	129243853	129243853	G	A	nonsynonymous	nonsynonymous	nonsynonymous
CNTN3	chr3	74383974	74383974	G	A	nonsynonymous	nonsynonymous	nonsynonymous
CSMD3	chr8	113484900	113484900	G	T	nonsynonymous	nonsynonymous	nonsynonymous
DAO	chr12	109283279	109283279	G	A	nonsynonymous	nonsynonymous	nonsynonymous
DCLK1	chr13	36428636	36428636	C	A	nonsynonymous	nonsynonymous	NA
DOPEY2	chr21	37665759	37665759	G	C	nonsynonymous	nonsynonymous	nonsynonymous
ELF4	chrX	129200832	129200832	G	A	nonsynonymous	nonsynonymous	nonsynonymous
GRID2IP	chr7	6548677	6548677	T	A	nonsynonymous	nonsynonymous	nonsynonymous
ITGBL1	chr13	102227873	102227873	G	C	nonsynonymous	nonsynonymous	nonsynonymous
JAG2	chr14	105618019	105618019	G	A	nonsynonymous	nonsynonymous	NA
KRI1	chr19	10670073	10670073	C	T	nonsynonymous	nonsynonymous	nonsynonymous
LRFN5	chr14	42360861	42360861	G	T	nonsynonymous	nonsynonymous	nonsynonymous
MAP3K4	chr6	161501971	161501971	C	T	nonsynonymous	NA	nonsynonymous
MEPCE	chr7	100027978	100027978	G	A	nonsynonymous	NA	NA
MTHFR	chr1	11854865	11854865	G	A	nonsynonymous	nonsynonymous	NA
N4BP2	chr4	40122261	40122261	C	G	nonsynonymous	nonsynonymous	nonsynonymous
NAP1L4	chr11	2985942	2985942	T	A	stopgain	stopgain	stopgain
NCOA6	chr20	33331002	33331002	G	T	nonsynonymous	nonsynonymous	nonsynonymous
NID2	chr14	52520812	52520812	G	A	nonsynonymous	nonsynonymous	nonsynonymous
NIF3L1	chr2	201756994	201756994	G	C	nonsynonymous	nonsynonymous	nonsynonymous
NOVA2	chr19	46457123	46457123	C	T	nonsynonymous	nonsynonymous	nonsynonymous
PABPC5	chrX	90690855	90690855	G	T	nonsynonymous	nonsynonymous	NA
PCDH19	chrX	99661705	99661705	C	T	nonsynonymous	nonsynonymous	nonsynonymous
PCDHB10	chr5	140572972	140572972	G	T	nonsynonymous	nonsynonymous	nonsynonymous
PCED1B	chr12	47629097	47629097	C	T	nonsynonymous	nonsynonymous	nonsynonymous
PER1	chr17	8049359	8049359	G	A	nonsynonymous	nonsynonymous	NA
PHF3	chr6	64422841	64422841	G	C	nonsynonymous	nonsynonymous	nonsynonymous
PLCB1	chr20	8721060	8721060	T	C	nonsynonymous	nonsynonymous	nonsynonymous
PPP1R9A	chr7	94919567	94919567	G	C	nonsynonymous	nonsynonymous	nonsynonymous
PRCC	chr1	156737648	156737648	C	A	nonsynonymous	nonsynonymous	nonsynonymous
PTPRC	chr1	198687390	198687390	C	T	nonsynonymous	nonsynonymous	nonsynonymous
PWP1	chr12	108086647	108086647	C	G	nonsynonymous	nonsynonymous	nonsynonymous
PYGL	chr14	51387704	51387704	G	A	nonsynonymous	nonsynonymous	NA
RAB1B	chr11	66043570	66043570	C	G	nonsynonymous	nonsynonymous	NA
SCFD1	chr14	31103227	31103227	T	C	nonsynonymous	nonsynonymous	NA
SCNN1A	chr12	6457341	6457341	C	A	nonsynonymous	nonsynonymous	nonsynonymous
SLC23A1	chr5	138707763	138707763	C	A	nonsynonymous	nonsynonymous	nonsynonymous
SLC35A1	chr6	88221168	88221168	A	G	nonsynonymous	nonsynonymous	nonsynonymous
SLC6A15	chr12	85270295	85270295	C	T	nonsynonymous	nonsynonymous	nonsynonymous
SPEF2	chr5	35814615	35814615	G	T	nonsynonymous	nonsynonymous	nonsynonymous
STK17B	chr2	197004435	197004435	G	C	nonsynonymous	NA	nonsynonymous
TGM7	chr15	43571424	43571424	G	A	nonsynonymous	NA	NA
TRMT61B	chr2	29092949	29092950	CT	-	frameshift_deletion	frameshift_deletion	NA
TTC23	chr15	99702064	99702064	C	T	nonsynonymous	nonsynonymous	NA
VCAN	chr5	82808017	82808017	G	A	nonsynonymous	nonsynonymous	NA
WDR6	chr3	49050334	49050334	C	G	nonsynonymous	nonsynonymous	nonsynonymous
WRN	chr8	30924586	30924586	T	C	nonsynonymous	nonsynonymous	NA
ZNF519	chr18	14105371	14105371	A	G	nonsynonymous	nonsynonymous	NA
ZNF829	chr19	37406800	37406800	C	A	nonsynonymous	nonsynonymous	nonsynonymous
A2ML1	chr12	8988110	8988110	A	G	NA	nonsynonymous	NA
ADAR	chr1	154573970	154573970	G	T	NA	nonsynonymous	NA
AGBL1	chr15	86702242	86702242	G	C	NA	nonsynonymous	nonsynonymous
BIRC6	chr2	32756467	32756467	C	G	NA	nonsynonymous	NA
BRCA2	chr13	32912843	32912843	G	C	NA	nonsynonymous	NA
CDH7	chr18	63526966	63526966	T	A	NA	nonsynonymous	NA
FGF7	chr15	49716709	49716709	G	A	NA	nonsynonymous	nonsynonymous
GMIP	chr19	19746265	19746265	C	T	NA	nonsynonymous	nonsynonymous
HIST2H2BE	chr1	149858138	149858138	G	A	NA	nonsynonymous	nonsynonymous
HSP90AB1	chr6	44219271	44219271	G	C	NA	nonsynonymous	NA
ITIH3	chr3	52831203	52831203	G	A	NA	nonsynonymous	NA
ITPR2	chr12	26816716	26816716	C	G	NA	nonsynonymous	nonsynonymous
KPNA1	chr3	122170381	122170381	C	A	NA	nonsynonymous	NA
MAD1L1	chr7	2188800	2188800	C	T	NA	nonsynonymous	NA
MARK1	chr1	220835180	220835180	G	A	NA	nonsynonymous	nonsynonymous
MMS19	chr10	99219863	99219863	G	A	NA	stopgain	stopgain

OR10AC1	chr7	143208667	143208667	C	T	NA	nonsynonymous	NA
PARP3	chr3	51982363	51982363	A	G	NA	nonsynonymous	NA
PHACTR3	chr20	58318194	58318194	G	A	NA	nonsynonymous	nonsynonymous
PISD	chr22	32019807	32019807	G	A	NA	nonsynonymous	nonsynonymous
PLD5	chr1	242264082	242264082	T	A	NA	nonsynonymous	nonsynonymous
PSCA	chr8	143763501	143763501	C	G	NA	nonsynonymous	nonsynonymous
PSD2	chr5	139218274	139218274	G	T	NA	nonsynonymous	NA
RAVER1	chr19	10444057	10444057	C	A	NA	stopgain	stopgain
RBM11	chr21	15599521	15599521	G	T	NA	nonsynonymous	NA
RC3H1	chr1	173930313	173930313	G	A	NA	stopgain	NA
RNH1	chr11	498082	498082	T	C	NA	nonsynonymous	NA
STAT1	chr2	191841611	191841611	C	T	NA	nonsynonymous	nonsynonymous
SUCO	chr1	172547503	172547503	G	A	NA	nonsynonymous	nonsynonymous
TFAP2B	chr6	50807907	50807907	T	C	NA	nonsynonymous	NA
TGM6	chr20	2377175	2377175	G	A	NA	nonsynonymous	NA
ZNF469	chr16	88495085	88495085	C	A	NA	nonsynonymous	NA
ZNF534	chr19	52941870	52941870	A	G	NA	nonsynonymous	NA
ABCA8	chr17	66903932	66903932	C	T	NA	NA	nonsynonymous
ACVR2B	chr3	38519650	38519650	C	T	NA	NA	nonsynonymous
ARHGAP31	chr3	119128455	119128455	G	C	NA	NA	nonsynonymous
ARMC6	chr19	19166165	19166165	C	T	NA	NA	nonsynonymous
C3orf36	chr3	133647421	133647421	G	A	NA	NA	nonsynonymous
CD8B2	chr2	107120750	107120750	C	T	NA	NA	nonsynonymous
CHGB	chr20	5903724	5903724	G	A	NA	NA	nonsynonymous
COL4A4	chr2	227985764	227985764	C	-	NA	NA	frameshift_deletion
CORO1C	chr12	109041223	109041223	G	A	NA	NA	nonsynonymous
DCBLD1	chr6	117859921	117859921	C	G	NA	NA	nonsynonymous
DLG1	chr3	196865221	196865221	C	T	NA	NA	nonsynonymous
DNAH11	chr7	21678636	21678636	T	C	NA	NA	nonsynonymous
ERBB4	chr2	212652843	212652843	G	T	NA	NA	nonsynonymous
EYS	chr6	65300683	65300683	A	C	NA	NA	nonsynonymous
F7	chr13	113770018	113770018	G	C	NA	NA	nonsynonymous
FAM98A	chr2	33810492	33810492	G	A	NA	NA	nonsynonymous
FRYL	chr4	48533315	48533315	G	T	NA	NA	nonsynonymous
GALNT17	chr7	71130430	71130430	C	T	NA	NA	nonsynonymous
GPR180	chr13	95273388	95273388	G	T	NA	NA	nonsynonymous
GPR45	chr2	105859312	105859312	G	A	NA	NA	nonsynonymous
GPRASP1	chrX	101910789	101910789	G	C	NA	NA	nonsynonymous
KALRN	chr3	124153158	124153158	T	C	NA	NA	nonsynonymous
KLHL1	chr13	70549897	70549897	C	A	NA	NA	nonsynonymous
LAMA5	chr20	60889466	60889466	G	A	NA	NA	nonsynonymous
MAB21L2	chr4	151505028	151505028	G	A	NA	NA	nonsynonymous
MEFV	chr16	3304640	3304640	C	T	NA	NA	nonsynonymous
MUC16	chr19	9061897	9061897	T	C	NA	NA	nonsynonymous
NAV3	chr12	78444770	78444770	C	G	NA	NA	nonsynonymous
NECTIN3	chr3	110837742	110837742	G	A	NA	NA	nonsynonymous
OR6N2	chr1	158746549	158746549	G	A	NA	NA	nonsynonymous
PIK3C2G	chr12	18544074	18544074	G	A	NA	NA	nonsynonymous
RUNX1T1	chr8	92982935	92982935	G	A	NA	NA	nonsynonymous
RWDD1	chr6	116914166	116914166	G	A	NA	NA	nonsynonymous
RYR2	chr1	237947592	237947592	G	A	NA	NA	nonsynonymous
S1PR5	chr19	10624628	10624628	G	A	NA	NA	nonsynonymous
SCN9A	chr2	167168234	167168234	G	T	NA	NA	nonsynonymous
SDK2	chr17	71346397	71346397	C	T	NA	NA	nonsynonymous
SLC27A1	chr19	17599902	17599902	C	G	NA	NA	nonsynonymous
STAB2	chr12	104046341	104046341	A	G	NA	NA	nonsynonymous
STOX1	chr10	70646110	70646110	G	T	NA	NA	nonsynonymous
STOX2	chr4	184930889	184930889	G	A	NA	NA	nonsynonymous
TTC3	chr21	38570258	38570258	C	T	NA	NA	nonsynonymous
TTN	chr2	179614364	179614364	C	G	NA	NA	nonsynonymous
TXLNA	chr1	32646843	32646843	G	A	NA	NA	nonsynonymous
UTP20	chr12	101723210	101723210	C	T	NA	NA	nonsynonymous
ZNF287	chr17	16456635	16456635	T	C	NA	NA	nonsynonymous
ZNF727	chr7	63538552	63538552	A	T	NA	NA	nonsynonymous
ZNF844	chr19	12186669	12186669	G	A	NA	NA	nonsynonymous

Gene	chromosome	Start	Stop	Ref	Alt	HK7-TB	HK7-TC	HK7-TD
ACACB	chr12	109661650	109661650	G	A	nonsynonymous	nonsynonymous	nonsynonymous
ADAMTSL3	chr15	84558921	84558921	A	G	nonsynonymous	nonsynonymous	nonsynonymous
AGBL2	chr11	47689224	47689224	T	G	nonsynonymous	nonsynonymous	nonsynonymous
AMER1	chrX	63409829	63409829	C	T	nonsynonymous	nonsynonymous	nonsynonymous
APOBEC3D	chr22	39418996	39418996	C	T	nonsynonymous	nonsynonymous	nonsynonymous
ARMCX4	chrX	100747361	100747361	T	C	nonsynonymous	nonsynonymous	nonsynonymous
BTG3	chr21	18981461	18981461	-	T	frameshift_insertion	frameshift_insertion	frameshift_insertion
C14orf39	chr14	60932738	60932738	G	A	nonsynonymous	nonsynonymous	nonsynonymous
C3orf30	chr3	118865425	118865425	G	A	nonsynonymous	nonsynonymous	nonsynonymous
C8orf87	chr8	94146542	94146542	T	C	nonsynonymous	NA	NA
CADM4	chr19	44131419	44131419	C	T	nonsynonymous	nonsynonymous	nonsynonymous
CDKN2A	chr9	21971170	21971170	A	T	nonsynonymous	nonsynonymous	nonsynonymous
CIT	chr12	120156522	120156522	G	A	nonsynonymous	nonsynonymous	nonsynonymous
CNTN3	chr3	74414746	74414746	G	T	nonsynonymous	nonsynonymous	nonsynonymous
CNTN3	chr3	74414752	74414752	G	A	stopgain	stopgain	stopgain
CPB2	chr13	46641513	46641513	G	A	nonsynonymous	nonsynonymous	nonsynonymous
CPXM1	chr20	2776020	2776020	A	G	nonsynonymous	nonsynonymous	nonsynonymous
CREB3L2	chr7	137565272	137565272	C	T	nonsynonymous	nonsynonymous	nonsynonymous
CTNND2	chr5	11397249	11397249	G	A	nonsynonymous	nonsynonymous	nonsynonymous
DAB1	chr1	57489219	57489219	C	G	nonsynonymous	nonsynonymous	nonsynonymous
ELMSAN1	chr14	74205279	74205279	G	C	stopgain	NA	NA
EPHA7	chr6	93955090	93955090	C	A	nonsynonymous	nonsynonymous	nonsynonymous
ERCC6L	chrX	71426238	71426238	C	A	nonsynonymous	nonsynonymous	nonsynonymous
EYA4	chr6	133834118	133834118	G	T	nonsynonymous	nonsynonymous	nonsynonymous
EYS	chr6	66204699	66204699	C	A	nonsynonymous	nonsynonymous	nonsynonymous
FCRL5	chr1	157516735	157516735	G	A	nonsynonymous	NA	NA
FMNL2	chr2	153405575	153405575	G	C	nonsynonymous	nonsynonymous	nonsynonymous
GAB3	chrX	153906564	153906564	A	G	nonsynonymous	nonsynonymous	nonsynonymous
GPR27	chr3	71804030	71804030	A	G	nonsynonymous	nonsynonymous	nonsynonymous
GSTM5	chr1	110260005	110260005	G	T	nonsynonymous	nonsynonymous	nonsynonymous
HELB	chr12	66717845	66717845	G	A	nonsynonymous	nonsynonymous	nonsynonymous
JCAD	chr10	30317540	30317540	G	C	nonsynonymous	nonsynonymous	nonsynonymous
KAT14	chr20	18143362	18143362	G	A	nonsynonymous	nonsynonymous	nonsynonymous
KBTBD11	chr8	1950798	1950798	G	A	stopgain	stopgain	NA
KIAA0825	chr5	93820562	93820593	GAGCTGCGATAAGAATTCGACTTTGTCTAAAG	-	frameshift_deletion	frameshift_deletion	frameshift_deletion
LILRA5	chr19	54822903	54822903	G	A	nonsynonymous	nonsynonymous	nonsynonymous
MB21D2	chr3	192516740	192516740	C	T	nonsynonymous	nonsynonymous	nonsynonymous
MCM9	chr6	119177591	119177591	T	C	nonsynonymous	nonsynonymous	nonsynonymous
NLRP3	chr1	247582370	247582370	C	A	nonsynonymous	nonsynonymous	nonsynonymous
NNT	chr5	43677838	43677838	T	A	nonsynonymous	nonsynonymous	nonsynonymous
NR1I2	chr3	119528973	119528973	G	A	nonsynonymous	nonsynonymous	NA
NR6A1	chr9	127287048	127287048	G	A	nonsynonymous	nonsynonymous	nonsynonymous
OR1L3	chr9	125438359	125438359	G	T	nonsynonymous	nonsynonymous	NA
OR51J1	chr11	5424631	5424631	C	G	nonsynonymous	nonsynonymous	NA
OSBPL9	chr1	52246848	52246848	G	C	nonsynonymous	NA	NA
PCF11	chr11	82874902	82874902	A	G	nonsynonymous	nonsynonymous	nonsynonymous
PIK3CA	chr3	178936091	178936091	G	A	nonsynonymous	nonsynonymous	nonsynonymous
PLEKHG4B	chr5	140118	140118	G	A	nonsynonymous	nonsynonymous	NA
PLEKHH1	chr14	68038878	68038878	G	A	nonsynonymous	nonsynonymous	NA
PPP1R3A	chr7	113518730	113518730	C	T	nonsynonymous	nonsynonymous	nonsynonymous
PSG8	chr19	43259315	43259315	G	T	stopgain	stopgain	stopgain
RBMXL2	chr11	7110956	7110956	G	A	nonsynonymous	nonsynonymous	nonsynonymous
RHPN2	chr19	33517516	33517516	G	A	nonsynonymous	nonsynonymous	nonsynonymous
RNPC3	chr1	104078058	104078058	G	A	nonsynonymous	nonsynonymous	nonsynonymous
SHPK	chr17	3533637	3533637	G	A	nonsynonymous	NA	nonsynonymous
SLC2A14	chr12	7966944	7966944	C	T	nonsynonymous	nonsynonymous	nonsynonymous
SLC6A8	chrX	152959830	152959830	-	CTACTC	nonframeshift_insertion	nonframeshift_insertion	nonframeshift_insertion
TAF1	chrX	70613265	70613265	G	A	nonsynonymous	nonsynonymous	nonsynonymous
TP53	chr17	7577022	7577022	G	A	stopgain	stopgain	stopgain
TRIP11	chr14	92488103	92488103	G	A	stopgain	NA	NA
TTN	chr2	179497134	179497134	C	T	nonsynonymous	nonsynonymous	nonsynonymous
TTN	chr2	179644099	179644099	C	A	nonsynonymous	nonsynonymous	nonsynonymous
UBA1	chrX	47062160	47062189	CTGGCACCCATAAACGCCTTCATTGGGGGC	-	nonframeshift_deletion	nonframeshift_deletion	nonframeshift_deletion
UBQLNL	chr11	5536268	5536268	C	A	nonsynonymous	nonsynonymous	nonsynonymous
USP48	chr1	22032998	22032998	G	A	stopgain	stopgain	stopgain
WFS1	chr4	6303116	6303116	G	C	nonsynonymous	NA	nonsynonymous
WNK2	chr9	96002102	96002114	GCTCGCGGAGGAG	-	frameshift_deletion	frameshift_deletion	frameshift_deletion
ZDHHC13	chr11	19197397	19197397	T	G	nonsynonymous	nonsynonymous	nonsynonymous

ZFHX3	chr16	72827390	72827390	T	G	nonsynonymous	nonsynonymous	nonsynonymous
ZNF536	chr19	30934738	30934738	G	A	nonsynonymous	nonsynonymous	nonsynonymous
ZNF718	chr4	155899	155899	G	A	nonsynonymous	nonsynonymous	nonsynonymous
ZNF784	chr19	56133709	56133709	C	T	nonsynonymous	nonsynonymous	NA
ACVRL1	chr12	52309928	52309928	G	A	NA	nonsynonymous	NA
DOPEY1	chr6	83848686	83848686	G	A	NA	nonsynonymous	NA
ELOVL5	chr6	53159151	53159151	G	A	NA	nonsynonymous	NA
FREM1	chr9	14859271	14859271	G	A	NA	nonsynonymous	nonsynonymous
MAB21L2	chr4	151504866	151504866	G	A	NA	nonsynonymous	nonsynonymous
OSGIN1	chr16	83999056	83999056	C	T	NA	nonsynonymous	NA
SEC24B	chr4	110447494	110447494	A	T	NA	nonsynonymous	nonsynonymous
TGS1	chr8	56698836	56698836	A	G	NA	nonsynonymous	NA
UBA3	chr3	69129487	69129487	C	A	NA	nonsynonymous	NA
ADGRE1	chr19	6924788	6924788	C	T	NA	NA	stopgain
F2RL3	chr19	17000929	17000929	C	T	NA	NA	nonsynonymous
FLRT2	chr14	86088979	86088979	C	A	NA	NA	nonsynonymous
JAG1	chr20	10639170	10639170	G	A	NA	NA	stopgain
KCNJ6	chr21	39086864	39086864	C	T	NA	NA	nonsynonymous
PSMF1	chr20	1099488	1099490	CTT	-	NA	NA	nonframeshift_deletion
REPS1	chr6	139266502	139266502	G	A	NA	NA	nonsynonymous
ZFHX4	chr8	77765127	77765127	C	A	NA	NA	nonsynonymous

Gene	chromosome	Start	Stop	Ref	Alt	HK8-TA	HK8-TB	HK8-TC	HK8-TD	HK8-TE
ABCA1	chr9	107593322	107593322	-	C	frameshift_insertion	frameshift_insertion	frameshift_insertion	frameshift_insertion	frameshift_insertion
ABCC5	chr3	183703153	183703153	C	T	nonsynonymous	nonsynonymous	nonsynonymous	nonsynonymous	nonsynonymous
ALDH9A1	chr1	165634297	165634297	C	T	nonsynonymous	nonsynonymous	nonsynonymous	nonsynonymous	nonsynonymous
AMD1	chr6	111213570	111213570	T	G	nonsynonymous	nonsynonymous	nonsynonymous	nonsynonymous	nonsynonymous
ANKRD20A	chr9	69403183	69403183	A	C	nonsynonymous	nonsynonymous	nonsynonymous	nonsynonymous	nonsynonymous
ANO8	chr19	17443910	17443910	C	A	nonsynonymous	nonsynonymous	nonsynonymous	nonsynonymous	nonsynonymous
AP1G1	chr16	71784159	71784159	T	C	nonsynonymous	nonsynonymous	nonsynonymous	nonsynonymous	nonsynonymous
AQP9	chr15	58467155	58467155	G	A	nonsynonymous	nonsynonymous	nonsynonymous	nonsynonymous	nonsynonymous
ATR	chr3	142297544	142297544	-	A	frameshift_insertion	frameshift_insertion	frameshift_insertion	frameshift_insertion	frameshift_insertion
BMPER	chr7	34006163	34006163	G	A	nonsynonymous	nonsynonymous	nonsynonymous	nonsynonymous	nonsynonymous
CAND1	chr12	67686531	67686531	T	G	nonsynonymous	nonsynonymous	nonsynonymous	nonsynonymous	nonsynonymous
CCDC172	chr10	118137980	118137980	T	A	nonsynonymous	nonsynonymous	nonsynonymous	nonsynonymous	nonsynonymous
CCDC181	chr1	169390777	169390777	G	A	nonsynonymous	nonsynonymous	nonsynonymous	nonsynonymous	nonsynonymous
CD1E	chr1	158325745	158325745	C	T	nonsynonymous	nonsynonymous	nonsynonymous	nonsynonymous	nonsynonymous
CDC14A	chr1	100970426	100970426	-	T	frameshift_insertion	frameshift_insertion	frameshift_insertion	frameshift_insertion	frameshift_insertion
CDH9	chr5	26890584	26890584	C	T	nonsynonymous	nonsynonymous	nonsynonymous	nonsynonymous	nonsynonymous
CHD5	chr1	6166802	6166802	C	G	nonsynonymous	nonsynonymous	nonsynonymous	nonsynonymous	nonsynonymous
CLK4	chr5	178039434	178039434	G	T	nonsynonymous	nonsynonymous	nonsynonymous	nonsynonymous	nonsynonymous
CRYBB1	chr22	27003867	27003867	G	A	nonsynonymous	nonsynonymous	nonsynonymous	nonsynonymous	nonsynonymous
CYFIP1	chr15	22962302	22962302	A	G	nonsynonymous	nonsynonymous	nonsynonymous	nonsynonymous	nonsynonymous
DEFB110	chr6	49976895	49976895	C	T	nonsynonymous	nonsynonymous	nonsynonymous	nonsynonymous	nonsynonymous
DLEC1	chr3	38129799	38129799	G	T	nonsynonymous	nonsynonymous	nonsynonymous	nonsynonymous	nonsynonymous
DST	chr6	56434662	56434662	C	G	nonsynonymous	nonsynonymous	nonsynonymous	nonsynonymous	nonsynonymous
DYNC1H1	chr14	102470860	102470860	A	G	nonsynonymous	nonsynonymous	nonsynonymous	nonsynonymous	nonsynonymous
EFCAB2	chr1	245250605	245250605	A	C	nonsynonymous	nonsynonymous	nonsynonymous	nonsynonymous	nonsynonymous
EGR4	chr2	73519217	73519217	C	G	nonsynonymous	nonsynonymous	nonsynonymous	nonsynonymous	nonsynonymous
EPDR1	chr7	37960782	37960782	G	A	nonsynonymous	nonsynonymous	nonsynonymous	nonsynonymous	nonsynonymous
FAM24B	chr10	124609980	124609980	C	A	nonsynonymous	nonsynonymous	nonsynonymous	nonsynonymous	nonsynonymous
FAM57A	chr17	643836	643836	T	A	nonsynonymous	nonsynonymous	nonsynonymous	nonsynonymous	nonsynonymous
FAM83H	chr8	144808381	144808381	T	C	nonsynonymous	nonsynonymous	nonsynonymous	nonsynonymous	nonsynonymous
FLNC	chr7	128478821	128478821	A	G	nonsynonymous	nonsynonymous	NA	NA	NA
GOLGA6L2	chr15	23686164	23686164	C	G	nonsynonymous	nonsynonymous	nonsynonymous	nonsynonymous	nonsynonymous
GRIN2A	chr16	9916134	9916134	-	GAC	nonframeshift_insertion	nonframeshift_insertion	nonframeshift_insertion	nonframeshift_insertion	nonframeshift_insertion
GRM1	chr6	146708066	146708066	C	T	nonsynonymous	nonsynonymous	nonsynonymous	nonsynonymous	nonsynonymous
GZMA	chr5	54398519	54398519	C	A	nonsynonymous	nonsynonymous	nonsynonymous	nonsynonymous	nonsynonymous
IGF2R	chr6	160454138	160454138	C	T	stopgain	stopgain	stopgain	stopgain	stopgain
INSL5	chr1	67263868	67263868	G	A	nonsynonymous	nonsynonymous	nonsynonymous	nonsynonymous	nonsynonymous
INTS9	chr8	28625791	28625791	C	T	nonsynonymous	nonsynonymous	nonsynonymous	nonsynonymous	nonsynonymous
KDM4C	chr9	6984356	6984356	A	G	nonsynonymous	nonsynonymous	nonsynonymous	nonsynonymous	nonsynonymous
KMT2D	chr12	49433227	49433227	-	G	frameshift_insertion	frameshift_insertion	frameshift_insertion	frameshift_insertion	frameshift_insertion
LAMA1	chr18	7012062	7012062	A	C	nonsynonymous	nonsynonymous	nonsynonymous	nonsynonymous	nonsynonymous
LMF2	chr22	50943392	50943392	G	C	nonsynonymous	nonsynonymous	nonsynonymous	nonsynonymous	nonsynonymous
LMNB2	chr19	2433885	2433885	A	G	nonsynonymous	nonsynonymous	nonsynonymous	nonsynonymous	nonsynonymous
LRIF1	chr1	111493928	111493928	G	T	nonsynonymous	nonsynonymous	nonsynonymous	nonsynonymous	nonsynonymous
MBOAT4	chr8	29990136	29990136	G	T	nonsynonymous	nonsynonymous	nonsynonymous	nonsynonymous	nonsynonymous
MEGF11	chr15	66207841	66207841	T	C	nonsynonymous	nonsynonymous	nonsynonymous	nonsynonymous	nonsynonymous
MORC1	chr3	108776242	108776242	G	T	nonsynonymous	nonsynonymous	nonsynonymous	nonsynonymous	nonsynonymous
MPEG1	chr11	58979426	58979426	G	A	nonsynonymous	nonsynonymous	nonsynonymous	nonsynonymous	nonsynonymous
MRRF	chr9	125054088	125054090	GAA	-	nonframeshift_deletion	nonframeshift_deletion	nonframeshift_deletion	nonframeshift_deletion	nonframeshift_deletion
MRRF	chr9	125054092	125054092	-	TTCA	frameshift_insertion	frameshift_insertion	frameshift_insertion	frameshift_insertion	frameshift_insertion
MRTO4	chr1	19584362	19584362	G	A	nonsynonymous	nonsynonymous	nonsynonymous	nonsynonymous	nonsynonymous
NAPG	chr18	10540338	10540338	T	G	nonsynonymous	nonsynonymous	nonsynonymous	nonsynonymous	nonsynonymous
NFE2L2	chr2	178098944	178098944	C	A	nonsynonymous	nonsynonymous	nonsynonymous	nonsynonymous	nonsynonymous
NPVF	chr7	25266292	25266292	C	T	nonsynonymous	nonsynonymous	nonsynonymous	nonsynonymous	nonsynonymous
NR1I2	chr3	119534605	119534605	G	A	nonsynonymous	nonsynonymous	nonsynonymous	nonsynonymous	nonsynonymous
OR5M10	chr11	56344287	56344287	A	G	nonsynonymous	nonsynonymous	nonsynonymous	nonsynonymous	nonsynonymous
OR5M11	chr11	56310662	56310662	C	A	nonsynonymous	nonsynonymous	nonsynonymous	nonsynonymous	nonsynonymous
OSBPL7	chr17	45890706	45890706	G	T	nonsynonymous	nonsynonymous	nonsynonymous	nonsynonymous	nonsynonymous
PAK3	chrX	110459699	110459699	G	C	nonsynonymous	nonsynonymous	nonsynonymous	nonsynonymous	nonsynonymous
PDSS2	chr6	107531761	107531761	A	G	nonsynonymous	nonsynonymous	nonsynonymous	nonsynonymous	nonsynonymous
PENK	chr8	57353917	57353917	C	A	nonsynonymous	nonsynonymous	nonsynonymous	nonsynonymous	nonsynonymous
PEX6	chr6	42932838	42932838	G	A	nonsynonymous	nonsynonymous	nonsynonymous	nonsynonymous	nonsynonymous
POP7	chr7	100304755	100304755	A	T	nonsynonymous	nonsynonymous	NA	NA	NA
PPFIA2	chr12	81719622	81719622	G	T	nonsynonymous	nonsynonymous	nonsynonymous	nonsynonymous	nonsynonymous
PPL	chr16	4935038	4935038	C	G	nonsynonymous	nonsynonymous	nonsynonymous	nonsynonymous	nonsynonymous
PXDNL	chr8	52366210	52366210	C	T	nonsynonymous	nonsynonymous	nonsynonymous	nonsynonymous	nonsynonymous
RARG	chr12	53609115	53609115	C	T	nonsynonymous	nonsynonymous	nonsynonymous	nonsynonymous	nonsynonymous
RBL1	chr20	35675523	35675523	T	C	nonsynonymous	nonsynonymous	nonsynonymous	nonsynonymous	nonsynonymous
RBMXL2	chr11	7111415	7111415	A	G	nonsynonymous	nonsynonymous	nonsynonymous	nonsynonymous	nonsynonymous
RELN	chr7	103194167	103194167	C	A	nonsynonymous	nonsynonymous	nonsynonymous	nonsynonymous	nonsynonymous
RPAP3	chr12	48091457	48091457	A	C	nonsynonymous	nonsynonymous	nonsynonymous	nonsynonymous	nonsynonymous
SFN	chr1	27190388	27190388	C	A	nonsynonymous	nonsynonymous	nonsynonymous	nonsynonymous	nonsynonymous
SLC2A13	chr12	40422282	40422282	G	A	nonsynonymous	nonsynonymous	nonsynonymous	nonsynonymous	nonsynonymous
SLC30A1	chr1	211749086	211749086	A	-	frameshift_deletion	frameshift_deletion	frameshift_deletion	frameshift_deletion	frameshift_deletion
SLC4A4	chr4	72363321	72363321	C	A	nonsynonymous	nonsynonymous	nonsynonymous	nonsynonymous	nonsynonymous
SPATA17	chr1	217947827	217947827	G	A	nonsynonymous	nonsynonymous	nonsynonymous	nonsynonymous	nonsynonymous
SPATA32	chr17	43333097	43333097	G	A	nonsynonymous	nonsynonymous	nonsynonymous	nonsynonymous	nonsynonymous
SRCIN1	chr17	36708701	36708701	G	A	nonsynonymous	nonsynonymous	nonsynonymous	nonsynonymous	nonsynonymous
SRRM2	chr16	2816406	2816406	-	TT	frameshift_insertion	frameshift_insertion	frameshift_insertion	frameshift_insertion	frameshift_insertion
STK24	chr13	99174101	99174101	G	T	nonsynonymous	nonsynonymous	nonsynonymous	nonsynonymous	nonsynonymous
TAB3	chrX	30872791	30872791	G	A	nonsynonymous	nonsynonymous	nonsynonymous	nonsynonymous	nonsynonymous
TBC1D1	chr4	38020013	38020013	-	A	frameshift_insertion	frameshift_insertion	frameshift_insertion	frameshift_insertion	frameshift_insertion
TEX13C	chrX	124455323	124455323	T	G	nonsynonymous	nonsynonymous	nonsynonymous	nonsynonymous	nonsynonymous
TP53	chr17	7579415	7579415	C	T	stopgain	stopgain	stopgain	stopgain	stopgain
UGT2B7	chr4	69973976	69973976	G	C	nonsynonymous	nonsynonymous	nonsynonymous	nonsynonymous	nonsynonymous
UQCC2	chr6	33665444	33665444	C	A	nonsynonymous	nonsynonymous	nonsynonymous	nonsynonymous	nonsynonymous
VPS13A	chr9	79981673	79981686	AGAGAAG AAGCTAA	-	frameshift_deletion	frameshift_deletion	frameshift_deletion	frameshift_deletion	frameshift_deletion
VPS13D	chr1	12516114	12516114	C	T	nonsynonymous	nonsynonymous	nonsynonymous	nonsynonymous	nonsynonymous
VWDE	chr7	12376623	12376623	A	G	nonsynonymous	nonsynonymous	nonsynonymous	nonsynonymous	nonsynonymous
ZBED5	chr11	10874458	10874458	T	C	nonsynonymous	nonsynonymous	nonsynonymous	nonsynonymous	nonsynonymous
ZNF138	chr7	64275318	64275318	T	G	nonsynonymous	nonsynonymous	NA	NA	NA
ZNF490	chr19	12721478	12721478	C	A	nonsynonymous	nonsynonymous	nonsynonymous	nonsynonymous	nonsynonymous
ZNF646	chr16	31089611	31089611	A	G	nonsynonymous	nonsynonymous	nonsynonymous	nonsynonymous	nonsynonymous

ADAP2	chr17	29271968	29271968	C	T	NA	nonsynonymous	nonsynonymous	nonsynonymous	nonsynonymous
DUOXA1	chr15	45415160	45415160	A	T	NA	nonsynonymous	nonsynonymous	nonsynonymous	nonsynonymous
ZNF534	chr19	52941990	52941990	C	T	NA	nonsynonymous	NA	NA	NA
ALOX12	chr17	6913704	6913704	C	A	NA	NA	nonsynonymous	NA	NA
ITIH5	chr10	7605051	7605051	C	A	NA	NA	nonsynonymous	NA	NA
PCDHA2	chr5	140175815	140175815	G	T	NA	NA	nonsynonymous	nonsynonymous	NA
PDZRN3	chr3	73440201	73440201	C	T	NA	NA	nonsynonymous	nonsynonymous	nonsynonymous
PRMT7	chr16	68389738	68389738	C	T	NA	NA	nonsynonymous	nonsynonymous	nonsynonymous
SPRR2D	chr1	153012727	153012727	G	C	NA	NA	nonsynonymous	nonsynonymous	nonsynonymous
ZNF766	chr19	52794177	52794177	A	T	NA	NA	nonsynonymous	nonsynonymous	nonsynonymous
CTIF	chr18	46287771	46287771	C	T	NA	NA	NA	nonsynonymous	nonsynonymous
BAGE;BAGI	chr21	11097622	11097622	G	T	NA	NA	NA	NA	nonsynonymous
NEUROG3	chr10	71332555	71332555	C	A	NA	NA	NA	NA	nonsynonymous
SOCS5	chr2	46986895	46986895	C	T	NA	NA	NA	NA	nonsynonymous

Gene	chromosome	Start	Stop	Ref	Alt	HK9-TA	HK9-TB	HK9-TC
ABCC8	chr11	17428469	17428469	A	T	nonsynonymous	nonsynonymous	nonsynonymous
ABL1	chr9	133729524	133729524	G	-	frameshift_deletion	frameshift_deletion	frameshift_deletion
ACOX3	chr4	8418139	8418139	A	C	nonsynonymous	nonsynonymous	nonsynonymous
AGBL5	chr2	27280181	27280181	G	C	nonsynonymous	nonsynonymous	nonsynonymous
AGBL5	chr2	27280299	27280299	T	A	nonsynonymous	nonsynonymous	nonsynonymous
BRCA2	chr13	32900640	32900640	G	T	nonsynonymous	nonsynonymous	nonsynonymous
C1orf116	chr1	207196039	207196039	G	A	nonsynonymous	nonsynonymous	nonsynonymous
C2CD6	chr2	202412328	202412328	T	C	nonsynonymous	nonsynonymous	nonsynonymous
C8B	chr1	57411510	57411510	C	A	nonsynonymous	nonsynonymous	nonsynonymous
C8B	chr1	57411545	57411545	C	G	nonsynonymous	nonsynonymous	nonsynonymous
CAMTA2	chr17	4872566	4872566	C	T	nonsynonymous	nonsynonymous	nonsynonymous
CAPN9	chr1	230925909	230925909	A	C	nonsynonymous	NA	NA
CASC4	chr15	44673075	44673075	A	C	nonsynonymous	nonsynonymous	nonsynonymous
CDC14C	chr7	48964771	48964771	A	G	nonsynonymous	nonsynonymous	nonsynonymous
CHD7	chr8	61773587	61773587	C	A	nonsynonymous	nonsynonymous	nonsynonymous
CPT1B	chr22	51011356	51011356	C	A	nonsynonymous	nonsynonymous	nonsynonymous
CXorf40A;C	chrX	149101888	149101888	G	C	nonsynonymous	NA	NA
DAAM1	chr14	59798057	59798057	C	G	nonsynonymous	nonsynonymous	nonsynonymous
DLEC1	chr3	38139055	38139055	C	T	nonsynonymous	nonsynonymous	nonsynonymous
DNAH11	chr7	21747369	21747369	G	A	stopgain	stopgain	stopgain
DNAJB5	chr9	34997110	34997110	G	A	nonsynonymous	nonsynonymous	nonsynonymous
E2F8	chr11	19256048	19256048	C	G	nonsynonymous	nonsynonymous	nonsynonymous
FAM120C	chrX	54160420	54160420	C	A	nonsynonymous	nonsynonymous	nonsynonymous
HEPACAM2	chr7	92837902	92837904	TGA	-	nonframeshift_deletion	nonframeshift_deletion	nonframeshift_deletion
HIST1H1D	chr6	26234759	26234759	C	T	nonsynonymous	nonsynonymous	nonsynonymous
HK3	chr5	176309059	176309059	C	T	nonsynonymous	nonsynonymous	nonsynonymous
HOXD4	chr2	177017389	177017389	C	T	nonsynonymous	nonsynonymous	nonsynonymous
HTR6	chr1	19992775	19992800	CCTGGCC AGTGCCG CCTGCTG GCCAG	-	frameshift_deletion	frameshift_deletion	frameshift_deletion
KCNH1	chr1	211280716	211280716	G	C	nonsynonymous	nonsynonymous	nonsynonymous
KHDRBS2	chr6	62887183	62887183	-	T	frameshift_insertion	frameshift_insertion	frameshift_insertion
KIAA0895	chr7	36374760	36374760	G	T	nonsynonymous	nonsynonymous	nonsynonymous
LCE5A	chr1	152484231	152484231	C	T	nonsynonymous	nonsynonymous	nonsynonymous
LRRC3B	chr3	26751807	26751807	C	G	nonsynonymous	nonsynonymous	nonsynonymous
MAST4	chr5	66459100	66459100	T	G	nonsynonymous	nonsynonymous	nonsynonymous
MAT2A	chr2	85770153	85770153	G	T	nonsynonymous	nonsynonymous	nonsynonymous
MIA2	chr14	39721969	39721969	G	A	nonsynonymous	nonsynonymous	nonsynonymous
MKL2	chr16	14346348	14346353	CCTCTG	-	nonframeshift_deletion	nonframeshift_deletion	nonframeshift_deletion
MYO1D	chr17	30821785	30821785	C	T	nonsynonymous	nonsynonymous	nonsynonymous
NEB	chr2	152426678	152426678	G	A	nonsynonymous	nonsynonymous	nonsynonymous
NEUROD4	chr12	55420336	55420336	C	T	nonsynonymous	nonsynonymous	nonsynonymous
NOTCH1	chr9	139413086	139413127	GTCATGG CAGGTGG CGCCGTG GAAGCAG GCGGCGC TGGCACA	-	nonframeshift_deletion	nonframeshift_deletion	nonframeshift_deletion
NRXN3	chr14	79432709	79432715	GGTGATG	-	frameshift_deletion	frameshift_deletion	frameshift_deletion
OCRL	chrX	128709198	128709198	C	T	nonsynonymous	nonsynonymous	nonsynonymous
OR10G7	chr11	123908946	123908946	G	A	nonsynonymous	nonsynonymous	nonsynonymous
P2RY2	chr11	72945623	72945623	G	A	nonsynonymous	nonsynonymous	nonsynonymous
PEAK1	chr15	77407508	77407508	G	C	nonsynonymous	nonsynonymous	nonsynonymous
PEX1	chr7	92146975	92146975	T	G	nonsynonymous	nonsynonymous	nonsynonymous
PHKB	chr16	47622911	47622916	AAAATT	-	nonframeshift_deletion	nonframeshift_deletion	nonframeshift_deletion
PRKAR2A	chr3	48831441	48831441	T	C	nonsynonymous	nonsynonymous	nonsynonymous
PTPN9	chr15	75819526	75819526	G	C	nonsynonymous	nonsynonymous	nonsynonymous
RETREG3	chr17	40734240	40734240	G	T	nonsynonymous	nonsynonymous	nonsynonymous
REXO1	chr19	1817259	1817259	C	T	nonsynonymous	nonsynonymous	nonsynonymous
RUFY1	chr5	179021870	179021870	G	A	nonsynonymous	nonsynonymous	nonsynonymous
SBF2	chr11	9810813	9810813	G	A	nonsynonymous	nonsynonymous	nonsynonymous
SCAPER	chr15	77046244	77046244	G	T	nonsynonymous	nonsynonymous	nonsynonymous
SERPINF1	chr17	1674356	1674356	G	C	nonsynonymous	nonsynonymous	nonsynonymous
SNX19	chr11	130784931	130784931	C	G	nonsynonymous	nonsynonymous	nonsynonymous
SPEF2	chr5	35646817	35646817	G	A	nonsynonymous	nonsynonymous	nonsynonymous
STON1;STC	chr2	48808989	48808989	C	G	nonsynonymous	nonsynonymous	nonsynonymous
SYNJ1	chr21	34003783	34003783	T	A	nonsynonymous	nonsynonymous	nonsynonymous
SYTL3	chr6	159086494	159086494	G	C	nonsynonymous	nonsynonymous	nonsynonymous
TAF1C	chr16	84213814	84213814	C	T	nonsynonymous	nonsynonymous	nonsynonymous

TIGD1	chr2	233413025	233413025	A	C	nonsynonymous	nonsynonymous	nonsynonymous
TRERF1	chr6	42236368	42236368	T	A	nonsynonymous	nonsynonymous	nonsynonymous
TRIM36	chr5	114499301	114499301	A	C	nonsynonymous	nonsynonymous	nonsynonymous
TRIP12	chr2	230668824	230668824	A	-	frameshift_deletion	frameshift_deletion	frameshift_deletion
TTC28	chr22	28559416	28559416	C	G	nonsynonymous	nonsynonymous	nonsynonymous
UBTF	chr17	42287051	42287051	C	G	nonsynonymous	nonsynonymous	nonsynonymous
UMODL1	chr21	43496229	43496229	G	T	nonsynonymous	nonsynonymous	nonsynonymous
VPS13B	chr8	100654265	100654265	T	G	nonsynonymous	nonsynonymous	nonsynonymous
VPS29	chr12	110933983	110933983	T	A	nonsynonymous	nonsynonymous	nonsynonymous
WDR6	chr3	49044811	49044811	C	T	stopgain	stopgain	stopgain
WNK3	chrX	54337697	54337697	G	T	nonsynonymous	nonsynonymous	nonsynonymous
ZNF169	chr9	97062477	97062477	A	G	nonsynonymous	nonsynonymous	nonsynonymous
ZNF175	chr19	52090408	52090408	A	G	nonsynonymous	nonsynonymous	nonsynonymous
ZNF318	chr6	43316313	43316313	G	A	nonsynonymous	nonsynonymous	nonsynonymous
ZNF445	chr3	44496669	44496669	C	A	stopgain	stopgain	stopgain
ABCB6	chr2	220079701	220079701	A	T	NA	nonsynonymous	nonsynonymous
ADGRB1	chr8	143556954	143556954	G	A	NA	nonsynonymous	nonsynonymous
BACH1	chr21	30693724	30693724	G	T	NA	nonsynonymous	NA
CD200R1	chr3	112647906	112647906	G	T	NA	nonsynonymous	nonsynonymous
CD6	chr11	60775052	60775052	C	G	NA	nonsynonymous	NA
CRB1	chr1	197403848	197403848	C	G	NA	nonsynonymous	nonsynonymous
CUX1	chr7	101917578	101917578	T	C	NA	nonsynonymous	NA
ENPP2	chr8	120629751	120629751	A	G	NA	nonsynonymous	nonsynonymous
EPHA10	chr1	38197086	38197086	C	-	NA	frameshift_deletion	NA
EPHA10	chr1	38197096	38197120	CTGTACTT CAATGCT GGGGTTA AAA	-	NA	frameshift_deletion	NA
FNDC1	chr6	159688904	159688904	C	G	NA	nonsynonymous	NA
GJD2	chr15	35045325	35045325	C	T	NA	nonsynonymous	NA
IGF1R	chr15	99472876	99472876	T	C	NA	nonsynonymous	nonsynonymous
ITGB4	chr17	73752889	73752889	A	G	NA	nonsynonymous	nonsynonymous
KRT5	chr12	52909004	52909004	A	C	NA	nonsynonymous	nonsynonymous
LRCOL1	chr12	133182733	133182733	C	A	NA	nonsynonymous	nonsynonymous
MYO7A	chr11	76905549	76905549	G	C	NA	nonsynonymous	nonsynonymous
NEK2	chr1	211836783	211836783	G	C	NA	nonsynonymous	nonsynonymous
NHSL2	chrX	71351940	71351940	T	C	NA	nonsynonymous	nonsynonymous
NLRP3	chr1	247582365	247582365	A	T	NA	nonsynonymous	nonsynonymous
NR0B1	chrX	30327273	30327273	C	T	NA	nonsynonymous	nonsynonymous
P2RY11;PP/	chr19	10224796	10224796	C	G	NA	nonsynonymous	nonsynonymous
PLEKHG3	chr14	65199528	65199528	T	G	NA	nonsynonymous	nonsynonymous
PRPF6	chr20	62655957	62655957	G	C	NA	nonsynonymous	nonsynonymous
RBBP6	chr16	24580624	24580624	G	T	NA	nonsynonymous	nonsynonymous
SACS	chr13	23908830	23908830	A	G	NA	nonsynonymous	nonsynonymous
SACS	chr13	23908926	23908926	A	-	NA	frameshift_deletion	frameshift_deletion
SIPA1	chr11	65416905	65416905	G	C	NA	nonsynonymous	nonsynonymous
TAS2R1	chr5	9629885	9629885	A	G	NA	nonsynonymous	nonsynonymous
TBR1	chr2	162280028	162280051	CCGGGCG CGGGCGC GGGCCCC GGG	-	NA	nonframeshift_deletion	nonframeshift_deletion
TG	chr8	133880466	133880466	C	G	NA	nonsynonymous	nonsynonymous
TNFRSF12A	chr16	3070406	3070406	G	-	NA	frameshift_deletion	frameshift_deletion
TNFRSF12A	chr16	3070409	3070409	G	C	NA	nonsynonymous	nonsynonymous
TTN	chr2	179485350	179485350	C	T	NA	nonsynonymous	NA
UBASH3A	chr21	43852272	43852272	T	C	NA	nonsynonymous	nonsynonymous
UBE2U	chr1	64680498	64680498	G	C	NA	nonsynonymous	NA
ZXDA	chrX	57936047	57936047	G	A	NA	nonsynonymous	NA
AKIP1	chr11	8938835	8938835	G	A	NA	NA	nonsynonymous
ATP13A4	chr3	193182773	193182773	T	C	NA	NA	nonsynonymous
HYDIN	chr16	70935089	70935101	CTACCCTG GATTC	-	NA	NA	frameshift_deletion
TIGD5	chr8	144681142	144681142	G	A	NA	NA	nonsynonymous
VENTX	chr10	135053557	135053557	G	T	NA	NA	nonsynonymous

Gene	chromosome	Start	Stop	Ref	Alt	HK10-TA	HK10-TB	HK10-TC
ADGRL2	chr1	82436164	82436164	C	A	nonsynonymous	NA	NA
AMZ2	chr17	66247299	66247299	G	A	nonsynonymous	nonsynonymous	nonsynonymous
ARPP21	chr3	35763230	35763230	G	A	nonsynonymous	nonsynonymous	nonsynonymous
ATG10	chr5	81548430	81548430	C	A	nonsynonymous	NA	NA
CABP4	chr11	67223163	67223163	C	T	nonsynonymous	NA	NA
CCZ1	chr7	5938495	5938495	C	T	nonsynonymous	NA	NA
CNTNAP1	chr17	40843995	40843995	G	A	nonsynonymous	NA	NA
DDX25	chr11	125791223	125791223	A	G	nonsynonymous	NA	NA
DEPDC5	chr22	32156688	32156688	G	A	nonsynonymous	nonsynonymous	nonsynonymous
DHX36	chr3	154042147	154042147	C	T	nonsynonymous	NA	NA
DIDO1	chr20	61524261	61524261	C	A	nonsynonymous	nonsynonymous	nonsynonymous
DSE	chr6	116720655	116720655	G	A	nonsynonymous	nonsynonymous	nonsynonymous
DSPP	chr4	88536173	88536173	G	A	nonsynonymous	NA	NA
EXO5	chr1	40981271	40981271	G	T	nonsynonymous	nonsynonymous	nonsynonymous
F13A1	chr6	6224982	6224982	G	A	nonsynonymous	NA	nonsynonymous
FAM20C	chr7	299939	299939	C	T	nonsynonymous	NA	NA
FAT1	chr4	187629400	187629400	C	A	stopgain	stopgain	stopgain
FBXW7	chr4	153245450	153245450	G	A	stopgain	stopgain	stopgain
FOXP1	chr3	71179663	71179669	GTTTGGC	-	frameshift_deletion	NA	NA
GUCY1A2	chr11	106856821	106856821	G	T	nonsynonymous	NA	NA
HSD17B2	chr16	82124578	82124578	A	T	nonsynonymous	nonsynonymous	nonsynonymous
KCNMA1	chr10	78673887	78673887	C	A	nonsynonymous	nonsynonymous	nonsynonymous
KIF16B	chr20	16337054	16337054	T	C	nonsynonymous	nonsynonymous	nonsynonymous
KMT2D	chr12	49422997	49422997	C	A	nonsynonymous	nonsynonymous	nonsynonymous
LHX9	chr1	197896805	197896805	C	A	nonsynonymous	nonsynonymous	nonsynonymous
MYOD1	chr11	17741464	17741464	C	G	nonsynonymous	nonsynonymous	nonsynonymous
NAT14	chr19	55998057	55998057	G	A	nonsynonymous	NA	NA
NFE2L2	chr2	178098975	178098975	A	C	nonsynonymous	nonsynonymous	nonsynonymous
OR6C2	chr12	55846787	55846787	G	T	nonsynonymous	NA	NA
OR8D1	chr11	124180179	124180179	C	A	nonsynonymous	nonsynonymous	nonsynonymous
PSMD1	chr2	231934761	231934761	C	T	nonsynonymous	nonsynonymous	nonsynonymous
RPN1	chr3	128356713	128356713	G	C	nonsynonymous	NA	NA
RTL1	chr14	101350701	101350701	T	C	nonsynonymous	NA	NA
SCN1A	chr2	166868749	166868749	G	A	nonsynonymous	nonsynonymous	nonsynonymous
SH3BP1	chr22	38040689	38040689	T	A	nonsynonymous	NA	NA
SNCG	chr10	88719398	88719398	A	T	nonsynonymous	NA	NA
SOGA3	chr6	127797251	127797251	T	A	nonsynonymous	NA	NA
SOX5	chr12	23998997	23998997	C	T	nonsynonymous	nonsynonymous	nonsynonymous
SRRT	chr7	100483980	100483980	C	A	nonsynonymous	nonsynonymous	nonsynonymous
TBL3	chr16	2027067	2027067	G	A	nonsynonymous	nonsynonymous	nonsynonymous
TLN1	chr9	35720865	35720865	C	T	nonsynonymous	nonsynonymous	nonsynonymous
TP53	chr17	7578265	7578265	A	T	nonsynonymous	nonsynonymous	nonsynonymous
TRIOBP	chr22	38168635	38168635	C	T	stopgain	stopgain	stopgain
AFF1	chr4	87968124	87968124	G	T	NA	nonsynonymous	nonsynonymous
APLP2	chr11	130003614	130003614	G	A	NA	nonsynonymous	nonsynonymous
APOB	chr2	21225584	21225584	T	A	NA	nonsynonymous	NA
ARHGAP35	chr19	47422414	47422414	T	C	NA	nonsynonymous	NA
ARMCX5-G	chrX	101971190	101971190	C	A	NA	nonsynonymous	NA
ASIC5	chr4	156784712	156784712	G	T	NA	nonsynonymous	NA
AURKC	chr19	57744843	57744843	G	A	NA	nonsynonymous	nonsynonymous
BBS9	chr7	33312651	33312651	C	G	NA	nonsynonymous	nonsynonymous
CACNG6	chr19	54496146	54496146	C	A	NA	nonsynonymous	NA
CCNE1	chr19	30313229	30313229	C	G	NA	nonsynonymous	NA
CCR8	chr3	39374789	39374789	T	G	NA	nonsynonymous	NA
CMYA5	chr5	79026842	79026842	C	T	NA	nonsynonymous	nonsynonymous
CNOT10	chr3	32761703	32761703	A	G	NA	nonsynonymous	NA
CRELD2	chr22	50313391	50313391	C	A	NA	nonsynonymous	NA
CYP3A43	chr7	99459458	99459458	G	A	NA	nonsynonymous	NA
DCAF1	chr3	51475078	51475078	T	C	NA	nonsynonymous	nonsynonymous
DHX8	chr17	41582074	41582074	G	A	NA	nonsynonymous	NA
DNAH8	chr6	38883097	38883097	G	A	NA	nonsynonymous	NA
DNHD1	chr11	6567471	6567471	C	G	NA	nonsynonymous	nonsynonymous
DYM	chr18	46690155	46690155	A	G	NA	nonsynonymous	nonsynonymous

DYNC2H1	chr11	103055661	103055661	G	T	NA	nonsynonymous	NA
EWSR1	chr22	29695312	29695312	C	T	NA	nonsynonymous	NA
EXOC3L2	chr19	45716520	45716520	G	A	NA	nonsynonymous	NA
EYS	chr6	65300314	65300314	C	G	NA	nonsynonymous	nonsynonymous
FRMD4B	chr3	69351545	69351545	T	C	NA	nonsynonymous	NA
GRID1	chr10	87615877	87615877	C	A	NA	nonsynonymous	NA
GSTM5	chr1	110255267	110255267	G	A	NA	nonsynonymous	NA
HELB	chr12	66717855	66717855	C	T	NA	nonsynonymous	NA
HYDIN	chr16	71218862	71218862	G	T	NA	nonsynonymous	nonsynonymous
IMMP2L	chr7	111161407	111161407	C	A	NA	nonsynonymous	NA
ITGB6	chr2	161030537	161030537	A	C	NA	nonsynonymous	nonsynonymous
KIR2DS4	chr19	55349192	55349192	G	C	NA	nonsynonymous	NA
KNDC1	chr10	135015062	135015062	C	A	NA	stopgain	NA
LMBR1	chr7	156529250	156529250	G	C	NA	nonsynonymous	NA
LRP1B	chr2	141079582	141079582	G	T	NA	nonsynonymous	NA
MAN2B1	chr19	12758408	12758408	G	C	NA	stopgain	stopgain
MAP3K15	chrX	19482389	19482389	A	G	NA	nonsynonymous	NA
MTA1	chr14	105927222	105927222	G	A	NA	nonsynonymous	nonsynonymous
MTERF2	chr12	107371635	107371635	C	G	NA	nonsynonymous	NA
NOTCH3	chr19	15289995	15289995	C	A	NA	nonsynonymous	NA
NOXRED1	chr14	77872314	77872314	T	A	NA	stopgain	NA
NTRK3	chr15	88420229	88420229	C	A	NA	nonsynonymous	NA
OR5AP2	chr11	56409822	56409822	C	A	NA	nonsynonymous	NA
PCF11	chr11	82894101	82894101	G	A	NA	nonsynonymous	NA
PLD2	chr17	4726052	4726052	G	T	NA	nonsynonymous	NA
PNPLA3	chr22	44328825	44328825	C	T	NA	nonsynonymous	NA
PSMD7	chr16	74335474	74335481	GGTACCAC	-	NA	frameshift_deletion	NA
RAD1	chr5	34911810	34911810	G	A	NA	nonsynonymous	NA
RPP30	chr10	92631825	92631825	C	G	NA	nonsynonymous	NA
SARDH	chr9	136584094	136584094	C	A	NA	nonsynonymous	NA
SLC22A23	chr6	3273583	3273583	G	C	NA	nonsynonymous	NA
SNX13	chr7	17930061	17930061	G	T	NA	nonsynonymous	NA
SPECC1	chr17	20108206	20108206	G	T	NA	stopgain	stopgain
SPHKAP	chr2	228881906	228881906	C	T	NA	nonsynonymous	nonsynonymous
TMEM72	chr10	45423380	45423380	G	A	NA	nonsynonymous	NA
TRAPPC8	chr18	29450952	29450952	G	C	NA	nonsynonymous	NA
WDR12	chr2	203760873	203760873	T	C	NA	nonsynonymous	NA
ZNF479	chr7	57188365	57188365	A	G	NA	nonsynonymous	NA
ZNF492	chr19	22836776	22836776	C	A	NA	nonsynonymous	NA
ZNF549	chr19	58049504	58049504	C	T	NA	stopgain	NA
ZNF746	chr7	149174834	149174834	G	T	NA	nonsynonymous	NA
ZNF816	chr19	53453754	53453754	T	C	NA	nonsynonymous	NA
CXCR1	chr2	219029422	219029422	G	T	NA	NA	nonsynonymous
IGSF1	chrX	130415224	130415224	C	T	NA	NA	stopgain

Gene	chromosome	Start	Stop	Ref	Alt	HK11-TA	HK11-TB	HK11-TC	HK11-TD
AAMP	chr2	219131292	219131292	G	A	nonsynonymous	nonsynonymous	nonsynonymous	nonsynonymous
AFF4	chr5	132270096	132270096	C	T	nonsynonymous	nonsynonymous	nonsynonymous	nonsynonymous
AGTPBP1	chr9	88190300	88190300	C	T	nonsynonymous	nonsynonymous	nonsynonymous	nonsynonymous
ALDH3A2	chr17	19564460	19564461	AA	-	frameshift_deletion	frameshift_deletion	frameshift_deletion	frameshift_deletion
AMER2	chr13	25743762	25743762	C	G	nonsynonymous	nonsynonymous	nonsynonymous	nonsynonymous
ATP13A2	chr1	17328804	17328804	C	T	nonsynonymous	NA	nonsynonymous	NA
BDP1	chr5	70818734	70818734	C	T	nonsynonymous	nonsynonymous	nonsynonymous	nonsynonymous
C8orf48	chr8	13425333	13425333	G	C	nonsynonymous	nonsynonymous	nonsynonymous	nonsynonymous
CASKIN2	chr17	73498447	73498447	C	T	nonsynonymous	nonsynonymous	nonsynonymous	nonsynonymous
CCDC170	chr6	151859321	151859321	G	T	stopgain	stopgain	stopgain	stopgain
CCDC178	chr18	30795533	30795533	G	C	nonsynonymous	nonsynonymous	nonsynonymous	nonsynonymous
CCP110	chr16	19548168	19548168	C	T	nonsynonymous	nonsynonymous	nonsynonymous	nonsynonymous
CDH9	chr5	26881451	26881451	C	A	stopgain	stopgain	stopgain	stopgain
CHD9	chr16	53301384	53301384	A	G	nonsynonymous	nonsynonymous	nonsynonymous	nonsynonymous
COL17A1	chr10	105811970	105811970	C	T	nonsynonymous	nonsynonymous	nonsynonymous	nonsynonymous
CTNNA2	chr2	80136889	80136889	C	T	nonsynonymous	nonsynonymous	nonsynonymous	nonsynonymous
CUZD1	chr10	124597044	124597044	C	A	nonsynonymous	nonsynonymous	nonsynonymous	nonsynonymous
ECE1	chr1	21584058	21584058	G	C	nonsynonymous	nonsynonymous	nonsynonymous	NA
EEF1D	chr8	144672119	144672119	C	T	nonsynonymous	nonsynonymous	NA	NA
ENPP2	chr8	120569870	120569870	C	T	nonsynonymous	NA	NA	nonsynonymous
ERCC4	chr16	14029219	14029219	G	A	nonsynonymous	nonsynonymous	nonsynonymous	nonsynonymous
FRYL	chr4	48501456	48501456	C	T	nonsynonymous	nonsynonymous	nonsynonymous	nonsynonymous
GREM2	chr1	240656456	240656456	G	C	nonsynonymous	nonsynonymous	nonsynonymous	nonsynonymous
INO80	chr15	41280020	41280020	C	G	nonsynonymous	nonsynonymous	nonsynonymous	nonsynonymous
KIF2B	chr17	51902185	51902185	G	T	nonsynonymous	nonsynonymous	nonsynonymous	nonsynonymous
KLHDC7B	chr22	50988111	50988111	G	A	nonsynonymous	NA	nonsynonymous	nonsynonymous
LCE3B	chr1	152586542	152586542	G	A	nonsynonymous	nonsynonymous	nonsynonymous	nonsynonymous
LRRTM1	chr2	80529702	80529702	C	A	nonsynonymous	nonsynonymous	nonsynonymous	nonsynonymous
ME2	chr18	48447052	48447052	C	T	nonsynonymous	nonsynonymous	nonsynonymous	nonsynonymous
MMP15	chr16	58075679	58075679	C	T	stopgain	stopgain	stopgain	stopgain
MUC12	chr7	100636117	100636117	C	A	nonsynonymous	NA	NA	NA
MUC12	chr7	100636278	100636278	G	A	nonsynonymous	NA	NA	NA
NLGN4X	chrX	5811699	5811699	T	A	nonsynonymous	nonsynonymous	nonsynonymous	nonsynonymous
NOS2	chr17	26084327	26084327	G	A	nonsynonymous	nonsynonymous	nonsynonymous	nonsynonymous
OR10G3	chr14	22038169	22038169	C	T	nonsynonymous	nonsynonymous	NA	nonsynonymous
OR13A1	chr10	45799503	45799503	G	A	nonsynonymous	nonsynonymous	nonsynonymous	nonsynonymous
OR2T4	chr1	248525912	248525912	C	T	stopgain	stopgain	stopgain	stopgain
OR5R1	chr11	56185215	56185215	C	T	nonsynonymous	nonsynonymous	nonsynonymous	nonsynonymous
OR8H3	chr11	55890059	55890059	G	A	nonsynonymous	nonsynonymous	nonsynonymous	nonsynonymous
PEX16	chr11	45935455	45935455	G	A	nonsynonymous	nonsynonymous	nonsynonymous	nonsynonymous
PHACTR3	chr20	58342333	58342333	G	A	nonsynonymous	nonsynonymous	nonsynonymous	NA
PINX1	chr8	10623155	10623155	C	T	nonsynonymous	NA	nonsynonymous	nonsynonymous
PLG	chr6	161174008	161174008	G	T	nonsynonymous	nonsynonymous	nonsynonymous	nonsynonymous
PRDM9	chr5	23522810	23522810	G	A	nonsynonymous	nonsynonymous	nonsynonymous	nonsynonymous
PTPRM	chr18	8244187	8244187	C	T	nonsynonymous	NA	NA	NA
RBM10	chrX	47030464	47030464	G	A	nonsynonymous	nonsynonymous	nonsynonymous	nonsynonymous
RBMXL2	chr11	7111484	7111484	G	A	nonsynonymous	nonsynonymous	nonsynonymous	nonsynonymous
RSPH4A	chr6	116951691	116951691	G	A	nonsynonymous	nonsynonymous	nonsynonymous	NA
2-Sep	chr2	242289503	242289503	G	A	nonsynonymous	nonsynonymous	nonsynonymous	nonsynonymous
7-Sep	chr7	35913267	35913267	G	A	nonsynonymous	NA	NA	NA
SETBP1	chr18	42532264	42532264	C	T	nonsynonymous	nonsynonymous	nonsynonymous	nonsynonymous
SKP2	chr5	36181969	36181969	G	A	nonsynonymous	nonsynonymous	nonsynonymous	nonsynonymous
SLC39A4	chr8	145641439	145641439	G	A	nonsynonymous	NA	nonsynonymous	nonsynonymous
SLITRK5	chr13	88328572	88328572	C	T	nonsynonymous	nonsynonymous	nonsynonymous	nonsynonymous
SPAG17	chr1	118548054	118548054	C	T	nonsynonymous	nonsynonymous	nonsynonymous	nonsynonymous
SRCAP	chr16	30732549	30732549	G	A	nonsynonymous	nonsynonymous	nonsynonymous	nonsynonymous
SRSF5	chr14	70238125	70238125	C	T	nonsynonymous	nonsynonymous	nonsynonymous	nonsynonymous
SSFA2	chr2	182780778	182780780	CAT	-	nonframeshift_deletion	NA	nonframeshift_deletion	NA
ST18	chr8	53092756	53092756	G	T	nonsynonymous	nonsynonymous	nonsynonymous	NA
TBX20	chr7	35242265	35242265	G	A	nonsynonymous	nonsynonymous	nonsynonymous	nonsynonymous
TBX22	chrX	79278649	79278649	A	G	nonsynonymous	nonsynonymous	nonsynonymous	nonsynonymous
TG	chr8	133910458	133910458	T	G	nonsynonymous	nonsynonymous	nonsynonymous	nonsynonymous
TMCO4	chr1	20082139	20082139	T	C	nonsynonymous	NA	nonsynonymous	nonsynonymous
TP53	chr17	7578253	7578253	C	A	nonsynonymous	nonsynonymous	nonsynonymous	nonsynonymous
ZNF219	chr14	21560731	21560731	G	C	stopgain	stopgain	stopgain	stopgain
ZNF442	chr19	12461216	12461216	G	C	nonsynonymous	nonsynonymous	nonsynonymous	nonsynonymous
ZNF479	chr7	57187804	57187804	C	T	nonsynonymous	nonsynonymous	nonsynonymous	NA
ZNF568	chr19	37441182	37441182	C	A	nonsynonymous	nonsynonymous	nonsynonymous	nonsynonymous
ZNF609	chr15	64967404	64967404	G	A	nonsynonymous	nonsynonymous	nonsynonymous	nonsynonymous
ZNF91	chr19	23543851	23543851	C	T	nonsynonymous	NA	nonsynonymous	NA
ZNF91	chr19	23543859	23543859	T	C	nonsynonymous	NA	nonsynonymous	NA
BAZ2B	chr2	160193974	160193974	C	T	NA	NA	nonsynonymous	NA
DKK3	chr11	12030113	12030113	G	T	NA	NA	nonsynonymous	NA
ERVV-1	chr19	53518038	53518038	G	T	NA	NA	nonsynonymous	NA
PHKB	chr16	47545601	47545601	G	A	NA	NA	nonsynonymous	NA
SI	chr3	164733762	164733762	C	A	NA	NA	nonsynonymous	NA
XIRP1	chr3	39228173	39228173	C	T	NA	NA	nonsynonymous	nonsynonymous
FIBCD1	chr9	133787218	133787218	G	A	NA	NA	NA	stopgain
VN1R2	chr19	53761718	53761718	C	A	NA	NA	NA	stopgain

Gene	chromosome	Start	Stop	Ref	Alt	HK12-TB	HK12-TC	HK12-TD
ABCA13	chr7	48311550	48311550	C	G	nonsynonymous	nonsynonymous	nonsynonymous
AK9	chr6	109894777	109894777	C	G	nonsynonymous	NA	NA
ANOS1	chrX	8504893	8504893	C	T	nonsynonymous	nonsynonymous	nonsynonymous
ARHGEF38	chr4	106534544	106534544	G	C	nonsynonymous	nonsynonymous	nonsynonymous
ASIC1	chr12	50472303	50472303	G	A	nonsynonymous	NA	NA
ATP2A1	chr16	28912052	28912052	A	G	nonsynonymous	nonsynonymous	nonsynonymous
BIN2	chr12	51707620	51707620	A	G	nonsynonymous	nonsynonymous	nonsynonymous
BTAF1	chr10	93722332	93722332	G	A	nonsynonymous	nonsynonymous	nonsynonymous
C20orf96	chr20	256666	256666	G	A	nonsynonymous	nonsynonymous	nonsynonymous
CBR1	chr21	37444152	37444152	A	G	nonsynonymous	nonsynonymous	nonsynonymous
CCDC106	chr19	56163799	56163799	A	G	nonsynonymous	nonsynonymous	nonsynonymous
CCDC84	chr11	118886131	118886131	G	T	nonsynonymous	nonsynonymous	nonsynonymous
CHD1	chr5	98208191	98208191	C	G	nonsynonymous	nonsynonymous	nonsynonymous
CHD9	chr16	53283827	53283827	G	A	nonsynonymous	nonsynonymous	nonsynonymous
CHUK	chr10	101979055	101979055	G	T	nonsynonymous	NA	nonsynonymous
COL28A1	chr7	7472130	7472130	G	A	nonsynonymous	nonsynonymous	nonsynonymous
CORIN	chr4	47625702	47625702	C	T	nonsynonymous	nonsynonymous	nonsynonymous
DDX58	chr9	32466367	32466367	A	G	nonsynonymous	NA	NA
DGCR2	chr22	19109664	19109664	G	A	nonsynonymous	nonsynonymous	nonsynonymous
DNAH10	chr12	124285897	124285902	GTCTGT	-	nonframeshift_deletion	nonframeshift_deletion	nonframeshift_deletion
DNAH6	chr2	84881793	84881793	C	A	nonsynonymous	nonsynonymous	nonsynonymous
DNAJC3	chr13	96443196	96443196	-	C	frameshift_insertion	frameshift_insertion	frameshift_insertion
DOCK3	chr3	51393579	51393579	A	G	nonsynonymous	nonsynonymous	nonsynonymous
ELMO1	chr7	36917695	36917704	GGCGAAAGCC	-	frameshift_deletion	frameshift_deletion	frameshift_deletion
FILIP1	chr6	76022591	76022591	A	C	nonsynonymous	nonsynonymous	nonsynonymous
FOXK1	chr7	4798963	4798963	C	T	nonsynonymous	nonsynonymous	nonsynonymous
GCNT3	chr15	59911638	59911638	A	T	nonsynonymous	nonsynonymous	nonsynonymous
GGA1	chr22	38021869	38021869	G	A	nonsynonymous	nonsynonymous	nonsynonymous
GMEB1	chr1	29019517	29019517	G	T	nonsynonymous	nonsynonymous	nonsynonymous
GPR149	chr3	154139235	154139236	TT	-	frameshift_deletion	frameshift_deletion	frameshift_deletion
GRIA4	chr11	105845088	105845088	C	G	nonsynonymous	nonsynonymous	nonsynonymous
GRM7	chr3	7620638	7620638	G	A	nonsynonymous	nonsynonymous	nonsynonymous
HCFC1	chrX	153215011	153215011	G	C	nonsynonymous	nonsynonymous	nonsynonymous
HIST1H3C	chr6	26045639	26045639	A	G	nonsynonymous	nonsynonymous	nonsynonymous
HYDIN	chr16	70852298	70852298	G	A	nonsynonymous	nonsynonymous	nonsynonymous
IVL	chr1	152882529	152882529	G	A	nonsynonymous	nonsynonymous	nonsynonymous
JAG1	chr20	10653491	10653491	T	C	nonsynonymous	nonsynonymous	nonsynonymous
JAKMIP3	chr10	133981578	133981578	G	C	nonsynonymous	nonsynonymous	nonsynonymous
KIAA1217	chr10	24833138	24833138	G	A	nonsynonymous	nonsynonymous	nonsynonymous
KIAA1324L	chr7	86521053	86521053	G	T	nonsynonymous	nonsynonymous	nonsynonymous
KIF20A	chr5	137517152	137517152	G	C	nonsynonymous	nonsynonymous	nonsynonymous
LEPR	chr1	66075978	66075978	A	G	nonsynonymous	nonsynonymous	nonsynonymous
MED26	chr19	16688239	16688239	C	G	nonsynonymous	nonsynonymous	nonsynonymous
MEGF8	chr19	42858004	42858004	G	A	nonsynonymous	nonsynonymous	nonsynonymous
MUC4	chr3	195517174	195517174	G	C	stopgain	stopgain	stopgain
MYO16	chr13	109318370	109318370	G	T	nonsynonymous	nonsynonymous	nonsynonymous
MYO18B	chr22	26343724	26343724	T	C	nonsynonymous	NA	NA
NXPH1	chr7	8790948	8790948	G	C	nonsynonymous	nonsynonymous	nonsynonymous
OR3A1	chr17	3195486	3195486	G	A	nonsynonymous	nonsynonymous	nonsynonymous
OTX2	chr14	57272096	57272096	G	T	nonsynonymous	nonsynonymous	nonsynonymous
PANX2	chr22	50617363	50617363	A	T	nonsynonymous	nonsynonymous	nonsynonymous
PAXIP1	chr7	154738463	154738463	-	GA	frameshift_insertion	frameshift_insertion	frameshift_insertion
PCP4	chr21	41239434	41239434	G	T	stopgain	stopgain	stopgain
PKD1	chr16	2159730	2159730	C	T	nonsynonymous	nonsynonymous	nonsynonymous
POM121L1	chr7	53103683	53103683	G	A	nonsynonymous	NA	nonsynonymous
PRDM5	chr4	121631519	121631519	C	T	nonsynonymous	nonsynonymous	nonsynonymous
PRKD1	chr14	30108023	30108023	T	C	nonsynonymous	nonsynonymous	nonsynonymous
RASSF5	chr1	206711563	206711563	G	C	nonsynonymous	nonsynonymous	nonsynonymous
SFI1	chr22	32012799	32012809	CCCCCATCTAC	-	frameshift_deletion	frameshift_deletion	frameshift_deletion
SLFN12L	chr17	33806733	33806733	C	G	nonsynonymous	nonsynonymous	nonsynonymous
SMCO1	chr3	196242042	196242042	C	G	nonsynonymous	nonsynonymous	nonsynonymous
SOS1	chr2	39278356	39278356	C	T	nonsynonymous	nonsynonymous	nonsynonymous
TM9SF3	chr10	98321889	98321889	A	G	nonsynonymous	nonsynonymous	nonsynonymous
TMEM107	chr17	8077848	8077848	C	A	nonsynonymous	nonsynonymous	nonsynonymous
TMEM116	chr12	112374496	112374496	-	A	frameshift_insertion	frameshift_insertion	frameshift_insertion
TOP2A	chr17	38546301	38546301	C	G	nonsynonymous	nonsynonymous	nonsynonymous
TP53	chr17	7578406	7578406	C	T	nonsynonymous	nonsynonymous	nonsynonymous
TRHDE	chr12	73046127	73046127	G	A	nonsynonymous	nonsynonymous	nonsynonymous
TRIP12	chr2	230663624	230663624	C	G	nonsynonymous	nonsynonymous	nonsynonymous
UPK1B	chr3	118909111	118909111	T	C	nonsynonymous	NA	nonsynonymous

URB2	chr1	229770838	229770838	C	T	stopgain	stopgain	stopgain
VWA8	chr13	42249403	42249403	T	C	nonsynonymous	nonsynonymous	nonsynonymous
VWC2	chr7	49815552	49815552	C	T	nonsynonymous	NA	NA
WNK4	chr17	40936462	40936462	C	T	nonsynonymous	nonsynonymous	nonsynonymous
ZNF180	chr19	44981349	44981349	T	C	nonsynonymous	nonsynonymous	nonsynonymous
ZNF534	chr19	52941158	52941158	C	T	stopgain	stopgain	stopgain
ZNF658	chr9	40784529	40784529	C	G	nonsynonymous	nonsynonymous	nonsynonymous
ALS2	chr2	202632011	202632011	C	T	NA	nonsynonymous	NA
FBXO4	chr5	41941312	41941312	G	A	NA	nonsynonymous	NA
GON4L	chr1	155735092	155735092	G	A	NA	nonsynonymous	NA
KRT76	chr12	53170610	53170610	G	C	NA	nonsynonymous	NA
PAPPA	chr9	119106841	119106841	G	C	NA	nonsynonymous	NA
PLEKHG4	chr16	67314851	67314851	C	G	NA	nonsynonymous	NA
RBM26	chr13	79894836	79894836	C	G	NA	nonsynonymous	NA
TBX18	chr6	85453991	85453991	G	C	NA	nonsynonymous	NA
TNK1	chr17	7291904	7291904	C	T	NA	nonsynonymous	NA
TWF1	chr12	44193259	44193259	G	C	NA	nonsynonymous	NA
U2SURP	chr3	142775160	142775160	A	-	NA	frameshift_deletion	NA
ZNF358	chr19	7585177	7585177	C	T	NA	nonsynonymous	NA
ZNF470	chr19	57088086	57088086	G	C	NA	nonsynonymous	NA
ARL3	chr10	104445580	104445580	C	G	NA	NA	nonsynonymous
CDC42BPA	chr1	227261603	227261603	C	G	NA	NA	nonsynonymous
L2HGDH	chr14	50750748	50750748	G	C	NA	NA	nonsynonymous
PDIA3	chr15	44061741	44061756	AAATAGTGAATAATGA	-	NA	NA	frameshift_deletion
ZFHX4	chr8	77764123	77764123	G	T	NA	NA	nonsynonymous

Gene	chromosome	Start	Stop	Ref	Alt	HK13-TA	HK13-TB	HK13-TC	HK13-TD
ADAM20	chr14	70991528	70991528	G	C	nonsynonymous	NA	nonsynonymous	nonsynonymous
ADCY4	chr14	24798636	24798636	C	T	nonsynonymous	NA	nonsynonymous	NA
ADGRV1	chr5	89968489	89968489	T	A	nonsynonymous	NA	NA	NA
AHNAK2	chr14	105405047	105405047	C	T	nonsynonymous	NA	NA	NA
ALMS1	chr2	73800002	73800002	A	C	nonsynonymous	NA	NA	NA
ALPK3	chr15	85400106	85400106	G	C	nonsynonymous	NA	NA	NA
AMY2A	chr1	104160663	104160663	A	C	nonsynonymous	nonsynonymous	nonsynonymous	nonsynonymous
ANKRD30B	chr18	14843081	14843081	A	C	nonsynonymous	nonsynonymous	nonsynonymous	nonsynonymous
ASH1L	chr1	155450790	155450790	A	G	nonsynonymous	NA	NA	NA
ASXL1	chr20	31021697	31021697	G	T	stopgain	stopgain	stopgain	stopgain
ATP7A	chrX	77264621	77264621	C	A	nonsynonymous	NA	NA	NA
ATP8B4	chr15	50158564	50158564	G	A	nonsynonymous	NA	NA	NA
BAZ1A	chr14	35227951	35227951	C	T	nonsynonymous	NA	NA	NA
BCAS3	chr17	58756850	58756850	G	C	nonsynonymous	NA	NA	NA
BIRC6	chr2	32743450	32743450	C	T	stopgain	NA	NA	NA
BMS1	chr10	43285811	43285811	A	C	nonsynonymous	NA	NA	NA
C1orf131	chr1	231374820	231374820	G	C	nonsynonymous	NA	nonsynonymous	nonsynonymous
C1orf174	chr1	3807468	3807468	C	G	nonsynonymous	NA	nonsynonymous	nonsynonymous
C2orf16	chr2	27803241	27803241	G	C	nonsynonymous	NA	nonsynonymous	nonsynonymous
C5orf42	chr5	37153859	37153859	C	G	nonsynonymous	NA	NA	NA
CAD	chr2	27458260	27458262	AAG	-	nonframeshift_deletion	nonframeshift_deletion	nonframeshift_deletion	nonframeshift_deletion
CAMK2G	chr10	75574808	75574808	G	A	nonsynonymous	NA	NA	NA
CASS4	chr20	55027880	55027880	G	C	nonsynonymous	NA	NA	NA
CATSPERG	chr19	38851256	38851256	C	-	frameshift_deletion	NA	frameshift_deletion	NA
CBL	chr11	119155917	119155917	C	T	nonsynonymous	nonsynonymous	nonsynonymous	nonsynonymous
CCDC40	chr17	78023899	78023899	G	C	nonsynonymous	nonsynonymous	nonsynonymous	nonsynonymous
CCDC8	chr19	46914849	46914849	G	C	nonsynonymous	NA	NA	NA
CDH6	chr5	31323000	31323000	G	A	nonsynonymous	NA	NA	NA
CHD7	chr8	61741316	61741316	C	T	nonsynonymous	NA	nonsynonymous	nonsynonymous
CNKSR2	chrX	21581371	21581371	G	A	nonsynonymous	NA	nonsynonymous	NA
COL6A3	chr2	238233446	238233446	G	T	nonsynonymous	NA	NA	NA
COL6A3	chr2	238262013	238262013	C	G	nonsynonymous	NA	NA	NA
COL6A5	chr3	130119937	130119937	G	C	nonsynonymous	NA	NA	NA
CPAMD8	chr19	17058025	17058025	G	C	nonsynonymous	NA	NA	NA
CRAMP1	chr16	1716480	1716480	C	A	nonsynonymous	nonsynonymous	nonsynonymous	nonsynonymous
CRLF3	chr17	29111276	29111276	C	G	nonsynonymous	nonsynonymous	nonsynonymous	nonsynonymous
CSAD	chr12	53554924	53554924	C	G	nonsynonymous	NA	nonsynonymous	NA
CTSH	chr15	79228041	79228041	T	A	nonsynonymous	nonsynonymous	nonsynonymous	nonsynonymous
DCLRE1B	chr1	114454266	114454266	G	T	nonsynonymous	NA	NA	NA
DNAH10	chr12	124258705	124258705	C	G	nonsynonymous	NA	NA	NA
DYNC1H1	chr14	102467375	102467375	C	T	stopgain	NA	NA	NA
DYRK1A	chr21	38845130	38845130	A	G	nonsynonymous	NA	NA	NA
EHD1	chr11	64622841	64622841	C	T	nonsynonymous	NA	NA	NA
EIF3A	chr10	120797754	120797754	G	C	nonsynonymous	NA	NA	NA
ERBB2	chr17	37865575	37865575	C	G	nonsynonymous	nonsynonymous	nonsynonymous	nonsynonymous
ERCC2	chr19	45855784	45855784	G	A	nonsynonymous	nonsynonymous	nonsynonymous	nonsynonymous
F2RL2	chr5	75914392	75914392	G	T	nonsynonymous	nonsynonymous	nonsynonymous	nonsynonymous
F9	chrX	138619201	138619201	C	G	nonsynonymous	NA	NA	NA
FAM111A	chr11	58920626	58920626	G	C	nonsynonymous	NA	NA	NA
FAM208B	chr10	5782276	5782276	G	A	nonsynonymous	NA	nonsynonymous	nonsynonymous
FAT1	chr4	187516980	187516980	G	T	nonsynonymous	NA	NA	NA
FBXO5	chr6	153292321	153292321	T	C	nonsynonymous	NA	nonsynonymous	NA
FOXR2	chrX	55650710	55650710	C	T	nonsynonymous	NA	NA	NA
FRMD6	chr14	52186874	52186874	A	T	nonsynonymous	nonsynonymous	nonsynonymous	nonsynonymous
GADD45GI	chr19	13067710	13067710	T	A	nonsynonymous	NA	nonsynonymous	NA
GEM	chr8	95264450	95264450	C	T	nonsynonymous	NA	NA	NA
GLOD5	chrX	48629409	48629409	G	C	nonsynonymous	NA	NA	NA
GMNC	chr3	190573343	190573343	C	T	nonsynonymous	NA	NA	NA
GNS	chr12	65134417	65134417	T	C	nonsynonymous	NA	nonsynonymous	NA
GOT1L1	chr8	37791892	37791892	G	C	nonsynonymous	NA	NA	NA
GRID2	chr4	94137913	94137913	G	C	nonsynonymous	NA	NA	NA
GRM3	chr7	86416078	86416078	G	C	nonsynonymous	NA	nonsynonymous	nonsynonymous
GSC	chr14	95234863	95234863	C	G	nonsynonymous	NA	NA	NA
GUCY1A2	chr11	106617284	106617284	G	T	nonsynonymous	NA	NA	NA
GUCY2F	chrX	108718725	108718725	G	C	nonsynonymous	NA	NA	NA
HDAC10	chr22	50684380	50684380	G	A	nonsynonymous	NA	NA	NA
HDGF	chr1	156715134	156715134	G	C	stopgain	NA	stopgain	stopgain
HEPH	chrX	65479981	65479981	C	A	nonsynonymous	NA	NA	NA
HS3ST5	chr6	114378657	114378657	C	T	nonsynonymous	nonsynonymous	nonsynonymous	nonsynonymous
HSPA2	chr14	65008131	65008131	G	C	nonsynonymous	NA	nonsynonymous	nonsynonymous
IFIT5	chr10	91178070	91178070	G	A	nonsynonymous	NA	NA	NA
IGF1R	chr15	99452090	99452090	G	A	nonsynonymous	NA	NA	NA
IKZF1	chr7	50450367	50450367	G	A	nonsynonymous	nonsynonymous	nonsynonymous	nonsynonymous
IKZF3	chr17	37922649	37922649	G	C	nonsynonymous	NA	NA	NA
INHA	chr2	220439466	220439466	G	C	nonsynonymous	NA	NA	NA
INSIG2	chr2	118860884	118860884	A	G	nonsynonymous	nonsynonymous	nonsynonymous	nonsynonymous
INTS2	chr17	59947328	59947328	G	A	nonsynonymous	NA	NA	NA
IP6K2	chr3	48726108	48726108	G	T	nonsynonymous	NA	nonsynonymous	nonsynonymous
IRAK1	chrX	153278575	153278575	C	T	nonsynonymous	NA	nonsynonymous	nonsynonymous
ITGA11	chr15	68609668	68609668	C	A	stopgain	NA	stopgain	stopgain
ITGA8	chr10	15559161	15559161	G	T	nonsynonymous	nonsynonymous	nonsynonymous	nonsynonymous
KCNE2	chr21	35742907	35742907	G	C	nonsynonymous	NA	NA	NA
KCNK15	chr20	43374817	43374817	C	A	nonsynonymous	NA	NA	NA
KCNQ5	chr6	73713672	73713672	C	G	nonsynonymous	NA	NA	NA
KCNQ5	chr6	73879499	73879499	-	T	frameshift_insertion	NA	frameshift_insertion	frameshift_insertion

KDM4B	chr19	5131979	5131979	C	T	nonsynonymous	NA	NA	NA
KIAA1551	chr12	32137385	32137385	G	A	nonsynonymous	NA	NA	NA
KIF15	chr3	44816786	44816786	C	T	nonsynonymous	NA	nonsynonymous	nonsynonymous
KLHL23;PH	chr2	170591987	170591987	G	C	nonsynonymous	NA	NA	NA
KLHL34	chrX	21674319	21674319	C	G	nonsynonymous	NA	NA	NA
KMT2A	chr11	118343387	118343387	G	A	nonsynonymous	NA	nonsynonymous	nonsynonymous
KMT2B	chr19	36212021	36212021	C	G	nonsynonymous	NA	NA	NA
KMT2C	chr7	151845334	151845334	G	A	stopgain	NA	stopgain	stopgain
KMT2C	chr7	151873549	151873549	-	A	frameshift_insertion	NA	NA	NA
KMT2D	chr12	49433646	49433646	G	C	stopgain	stopgain	stopgain	stopgain
KMT2D	chr12	49444552	49444552	G	A	nonsynonymous	NA	NA	NA
LAMA3	chr18	21478721	21478721	G	A	nonsynonymous	NA	nonsynonymous	NA
LHCGR	chr2	48915210	48915210	A	T	nonsynonymous	NA	NA	NA
LMTK2	chr7	97822986	97822986	A	G	nonsynonymous	NA	NA	NA
MANEA	chr6	96034796	96034796	T	G	nonsynonymous	nonsynonymous	nonsynonymous	nonsynonymous
11-Mar	chr5	16067587	16067587	G	A	nonsynonymous	NA	nonsynonymous	NA
MAST3	chr19	18254766	18254766	G	T	nonsynonymous	NA	nonsynonymous	NA
MCM3AP	chr21	47693475	47693475	C	T	nonsynonymous	NA	nonsynonymous	NA
MED12L	chr3	150883644	150883644	A	T	nonsynonymous	NA	NA	NA
MED14OS	chrX	40594795	40594795	G	C	nonsynonymous	NA	nonsynonymous	nonsynonymous
MITD1	chr2	99787090	99787090	C	G	nonsynonymous	NA	NA	NA
MPHOSPH	chr13	20220673	20220673	A	C	nonsynonymous	nonsynonymous	nonsynonymous	nonsynonymous
MRFAP1	chr4	6642650	6642650	G	A	nonsynonymous	NA	nonsynonymous	nonsynonymous
MUC16	chr19	9065300	9065300	G	T	nonsynonymous	NA	NA	NA
MYH13	chr17	10235475	10235475	C	T	nonsynonymous	NA	NA	NA
MYH7B	chr20	33568475	33568475	G	A	nonsynonymous	nonsynonymous	nonsynonymous	nonsynonymous
MYH8	chr17	10317704	10317704	A	C	nonsynonymous	NA	NA	NA
MYO15A	chr17	18022578	18022578	C	A	nonsynonymous	NA	NA	NA
MYO18A	chr17	27445161	27445161	G	A	nonsynonymous	NA	NA	NA
MYO18B	chr22	26400763	26400763	G	T	nonsynonymous	NA	nonsynonymous	NA
MYO9A	chr15	72191222	72191222	C	T	nonsynonymous	NA	NA	NA
MYOCD	chr17	12642544	12642544	G	C	nonsynonymous	NA	nonsynonymous	nonsynonymous
NBR1	chr17	41346442	41346442	-	T	frameshift_insertion	frameshift_insertion	frameshift_insertion	frameshift_insertion
NCOR2	chr12	124826416	124826416	G	A	nonsynonymous	NA	nonsynonymous	nonsynonymous
NDUFA10	chr2	240951071	240951071	C	G	nonsynonymous	NA	NA	NA
NEGR1	chr1	72163819	72163819	T	C	nonsynonymous	nonsynonymous	nonsynonymous	nonsynonymous
NFIB	chr9	14155884	14155884	C	T	nonsynonymous	NA	NA	NA
NLRC3	chr16	3613965	3613965	T	A	nonsynonymous	NA	nonsynonymous	nonsynonymous
NME7	chr1	169102052	169102052	G	C	nonsynonymous	NA	NA	NA
NOMO2	chr16	18573231	18573231	C	A	nonsynonymous	NA	NA	NA
NOS2	chr17	26093567	26093567	A	T	nonsynonymous	NA	NA	NA
NUBP2	chr16	1838570	1838570	G	T	nonsynonymous	NA	NA	NA
NUP88	chr17	5308403	5308403	C	G	nonsynonymous	NA	NA	NA
NUP93	chr16	56873439	56873439	G	C	nonsynonymous	NA	nonsynonymous	NA
OLIG3	chr6	137814761	137814761	G	T	nonsynonymous	NA	NA	NA
OPLAH	chr8	145109762	145109762	G	C	nonsynonymous	nonsynonymous	nonsynonymous	nonsynonymous
OR2AG2	chr11	6789826	6789826	G	T	nonsynonymous	nonsynonymous	nonsynonymous	nonsynonymous
OR2F2	chr7	143632783	143632783	C	T	nonsynonymous	nonsynonymous	nonsynonymous	nonsynonymous
OR8H3	chr11	55890114	55890114	C	T	nonsynonymous	NA	NA	NA
P2RX7	chr12	121614963	121614964	AC	-	frameshift_deletion	frameshift_deletion	frameshift_deletion	frameshift_deletion
PANX2	chr22	50616466	50616466	C	T	nonsynonymous	nonsynonymous	nonsynonymous	nonsynonymous
PCDH11X	chrX	91873617	91873617	C	T	nonsynonymous	nonsynonymous	nonsynonymous	nonsynonymous
PCDH15	chr10	56424009	56424009	A	T	nonsynonymous	nonsynonymous	nonsynonymous	nonsynonymous
PCDHA7	chr5	140214740	140214740	G	A	nonsynonymous	NA	nonsynonymous	nonsynonymous
PCNT	chr21	47786980	47786980	C	T	nonsynonymous	NA	nonsynonymous	nonsynonymous
PCSK2	chr20	17462360	17462360	T	C	nonsynonymous	nonsynonymous	nonsynonymous	nonsynonymous
PKHD1L1	chr8	110535097	110535097	C	T	nonsynonymous	NA	NA	NA
PLEC	chr8	144991297	144991297	T	A	nonsynonymous	NA	NA	NA
PLEC	chr8	144995519	144995519	C	T	nonsynonymous	nonsynonymous	nonsynonymous	nonsynonymous
PMM2	chr16	8904949	8904949	G	A	nonsynonymous	NA	nonsynonymous	nonsynonymous
POU4F2	chr4	147560486	147560486	-	CGA	nonframeshift_insertion	nonframeshift_insertion	nonframeshift_insertion	nonframeshift_insertion
PPFIA2	chr12	82147942	82147942	G	A	nonsynonymous	NA	NA	NA
PPFIBP1	chr12	27802973	27802973	C	T	stopgain	NA	NA	NA
PPIP5K2	chr5	102526581	102526581	C	G	nonsynonymous	NA	NA	NA
PPOX	chr1	161138866	161138866	G	C	nonsynonymous	NA	NA	NA
PPP1R3A	chr7	113517791	113517791	A	G	nonsynonymous	NA	nonsynonymous	nonsynonymous
PRKAA1	chr5	40762930	40762930	T	C	nonsynonymous	NA	nonsynonymous	nonsynonymous
PRMT7	chr16	68358710	68358710	G	T	nonsynonymous	NA	NA	NA
PROC	chr2	128186487	128186487	G	A	nonsynonymous	nonsynonymous	nonsynonymous	nonsynonymous
PRR14	chr16	30666477	30666477	C	T	nonsynonymous	NA	NA	NA
PRRC2C	chr1	171510309	171510309	C	T	nonsynonymous	NA	NA	NA
PSMD6	chr3	63996587	63996587	A	G	nonsynonymous	nonsynonymous	nonsynonymous	nonsynonymous
PTPN7	chr1	202117745	202117745	G	T	nonsynonymous	NA	NA	NA
RAI14	chr5	34811195	34811195	G	A	nonsynonymous	NA	NA	NA
RARRES3	chr11	63313696	63313696	A	T	nonsynonymous	NA	NA	NA
RBAK	chr7	5104024	5104024	G	C	nonsynonymous	NA	NA	NA
REL	chr2	61121582	61121582	G	C	nonsynonymous	NA	NA	NA
RICTOR	chr5	38949979	38949979	G	A	nonsynonymous	NA	nonsynonymous	nonsynonymous
RIMBP2	chr12	130892273	130892273	C	G	nonsynonymous	NA	NA	NA
RIPOR1	chr16	67576305	67576305	C	T	nonsynonymous	NA	NA	NA
RNF8	chr6	37336587	37336587	C	G	nonsynonymous	NA	NA	NA
RNPS1	chr16	2305719	2305719	C	G	nonsynonymous	nonsynonymous	nonsynonymous	nonsynonymous
RP1	chr8	55537678	55537678	G	A	nonsynonymous	nonsynonymous	nonsynonymous	nonsynonymous
RTL9	chrX	109695401	109695401	C	A	nonsynonymous	NA	NA	NA
RUFY4	chr2	218939852	218939852	A	T	nonsynonymous	NA	NA	NA
RUNX2	chr6	45296506	45296506	C	A	nonsynonymous	NA	nonsynonymous	NA

SAMD12	chr8	119391813	119391813	T	C	nonsynonymous	NA	nonsynonymous	nonsynonymous
SAR1A	chr10	71917602	71917602	T	C	nonsynonymous	nonsynonymous	nonsynonymous	nonsynonymous
SARDH	chr9	136584095	136584095	C	G	nonsynonymous	NA	nonsynonymous	nonsynonymous
SEL1L2	chr20	13856737	13856737	C	T	nonsynonymous	NA	NA	NA
SEMA4F	chr2	74907005	74907005	C	T	nonsynonymous	NA	nonsynonymous	nonsynonymous
SERPINA9	chr14	94935782	94935782	C	A	nonsynonymous	NA	NA	NA
SERPING1	chr11	57367397	57367397	G	T	nonsynonymous	NA	NA	NA
SF1	chr11	64533543	64533543	C	G	nonsynonymous	NA	NA	NA
SIGLEC9	chr19	51628983	51628983	C	T	nonsynonymous	NA	NA	NA
SLC16A1	chr1	113460165	113460165	C	A	nonsynonymous	NA	NA	NA
SLC28A1	chr15	85448788	85448788	T	A	nonsynonymous	NA	NA	NA
SLC28A3	chr9	86900881	86900881	C	G	nonsynonymous	NA	nonsynonymous	nonsynonymous
SLC44A2	chr19	10748713	10748713	A	T	nonsynonymous	NA	NA	NA
SLC4A9	chr5	139742617	139742617	C	A	nonsynonymous	NA	nonsynonymous	NA
SLC9A5	chr16	67286567	67286567	C	A	nonsynonymous	NA	NA	NA
SNAP91	chr6	84350864	84350864	C	G	nonsynonymous	NA	nonsynonymous	nonsynonymous
SNX27	chr1	151584693	151584694	GG	-	frameshift_deletion	NA	NA	NA
SOX21	chr13	95364195	95364195	C	G	nonsynonymous	NA	nonsynonymous	nonsynonymous
SP7	chr12	53722014	53722014	C	G	nonsynonymous	NA	nonsynonymous	NA
SP7	chr12	53722490	53722490	C	A	nonsynonymous	NA	NA	NA
SPATA31E1	chr9	90501908	90501908	G	C	nonsynonymous	NA	NA	NA
SPG7	chr16	89616952	89616952	G	A	nonsynonymous	NA	NA	NA
SPIRE2	chr16	89916755	89916755	G	A	stopgain	NA	NA	NA
SPTBN5	chr15	42168349	42168349	A	T	nonsynonymous	nonsynonymous	nonsynonymous	nonsynonymous
SRGAP1	chr12	64456757	64456757	G	A	nonsynonymous	NA	NA	NA
SRGAP3	chr3	9094728	9094728	C	G	nonsynonymous	NA	NA	NA
SSH2	chr17	27958528	27958528	C	G	nonsynonymous	NA	NA	NA
STAG1	chr3	136152480	136152480	C	A	nonsynonymous	NA	NA	NA
SYNE1	chr6	152461104	152461104	A	T	nonsynonymous	NA	nonsynonymous	nonsynonymous
TAF7L	chrX	100533074	100533074	C	G	nonsynonymous	NA	NA	NA
TAGLN2	chr1	159889498	159889498	T	C	nonsynonymous	NA	nonsynonymous	nonsynonymous
TBC1D14	chr4	6925480	6925480	C	A	nonsynonymous	NA	NA	NA
TBC1D5	chr3	17208352	17208352	C	G	nonsynonymous	NA	NA	NA
TDG	chr12	104370719	104370719	C	T	nonsynonymous	NA	NA	NA
TENM2	chr5	167489270	167489270	G	C	nonsynonymous	NA	NA	NA
THAP11	chr16	67876779	67876787	CAGCAGCAG	-	nonframeshift_deletion	NA	nonframeshift_deletion	nonframeshift_deletion
THNSL1	chr10	25314218	25314218	C	G	stopgain	NA	stopgain	stopgain
TJP1	chr15	30008984	30008984	G	A	nonsynonymous	NA	nonsynonymous	nonsynonymous
TLCD1	chr17	27052378	27052378	T	-	frameshift_deletion	frameshift_deletion	frameshift_deletion	frameshift_deletion
TMPRSS4	chr11	117978572	117978572	G	A	nonsynonymous	NA	nonsynonymous	nonsynonymous
TP53	chr17	7578188	7578188	C	A	stopgain	stopgain	stopgain	stopgain
TRAPPC1	chr17	7834854	7834854	G	A	nonsynonymous	NA	nonsynonymous	NA
TRAPPC10	chr21	45522713	45522713	C	T	nonsynonymous	NA	NA	NA
TRAPPC8	chr18	29488281	29488281	T	C	nonsynonymous	nonsynonymous	nonsynonymous	nonsynonymous
TRIM41	chr5	180651477	180651477	-	T	frameshift_insertion	NA	NA	NA
TRIM9	chr14	51475909	51475909	G	A	stopgain	NA	NA	NA
TRPA1	chr8	72975073	72975073	T	G	nonsynonymous	NA	NA	NA
TRPM7	chr15	50884572	50884572	G	A	nonsynonymous	NA	NA	NA
TTC34	chr1	2576954	2576954	C	T	nonsynonymous	NA	NA	NA
UBA2	chr19	34922775	34922775	G	T	stopgain	NA	NA	NA
UVSSA	chr4	1345559	1345559	-	AA	frameshift_insertion	NA	NA	NA
VCPIP1	chr8	67576741	67576741	C	G	nonsynonymous	NA	NA	NA
VEZT	chr12	95656715	95656715	A	G	nonsynonymous	nonsynonymous	nonsynonymous	nonsynonymous
WDR59	chr16	74943722	74943722	C	G	nonsynonymous	NA	nonsynonymous	nonsynonymous
XPNPEP3	chr22	41277977	41277977	G	C	nonsynonymous	NA	NA	NA
ZBTB5	chr9	37441669	37441669	G	A	stopgain	NA	NA	NA
ZNF397	chr18	32825531	32825531	G	A	nonsynonymous	NA	nonsynonymous	nonsynonymous
ZNF608	chr5	123983180	123983180	G	A	nonsynonymous	NA	NA	NA
ZNF653	chr19	11616348	11616348	G	T	nonsynonymous	NA	NA	NA
ZNF704	chr8	81571894	81571894	C	A	nonsynonymous	NA	nonsynonymous	nonsynonymous
ZNF75A	chr16	3367693	3367693	C	G	nonsynonymous	NA	NA	NA
ZNF768	chr16	30537133	30537133	G	C	nonsynonymous	NA	NA	NA
ZNF831	chr20	57768820	57768820	-	A	frameshift_insertion	NA	NA	NA
ABCA13	chr7	48416105	48416105	G	C	NA	nonsynonymous	NA	nonsynonymous
ABHD11	chr7	73151401	73151401	C	G	NA	nonsynonymous	NA	NA
AMY2B	chr1	104120214	104120214	C	T	NA	stopgain	NA	stopgain
ARHGAP44	chr17	12877525	12877525	C	T	NA	nonsynonymous	NA	nonsynonymous
BAP1	chr3	52442557	52442557	G	C	NA	nonsynonymous	NA	nonsynonymous
BTBD9	chr6	38562050	38562050	A	G	NA	nonsynonymous	NA	nonsynonymous
CAMTA1	chr1	7806049	7806049	G	T	NA	nonsynonymous	NA	nonsynonymous
CCL13	chr17	32683585	32683585	A	G	NA	nonsynonymous	NA	nonsynonymous
CDKL3	chr5	133644339	133644339	C	G	NA	nonsynonymous	NA	NA
CEP78	chr9	80881599	80881599	C	A	NA	stopgain	NA	stopgain
CPSF1	chr8	145622967	145622967	G	C	NA	stopgain	NA	stopgain
DARS2	chr1	173814391	173814391	G	C	NA	nonsynonymous	NA	nonsynonymous
DIDO1	chr20	61542964	61542964	T	C	NA	nonsynonymous	NA	nonsynonymous
DNAAF2	chr14	50100723	50100723	G	A	NA	nonsynonymous	NA	nonsynonymous
DNMBP	chr10	101689673	101689673	C	T	NA	nonsynonymous	NA	nonsynonymous
DSP	chr6	7565643	7565643	C	T	NA	stopgain	NA	NA
DTNA	chr18	32462120	32462120	G	C	NA	nonsynonymous	NA	nonsynonymous
ECI2	chr6	4116258	4116258	C	G	NA	nonsynonymous	NA	nonsynonymous
EP400	chr12	132529228	132529228	G	A	NA	nonsynonymous	NA	nonsynonymous
EXOSC4	chr8	145134901	145134901	A	G	NA	nonsynonymous	NA	nonsynonymous
FCN2	chr9	137779137	137779137	C	T	NA	nonsynonymous	NA	nonsynonymous
GALNT13	chr2	155295230	155295230	G	A	NA	nonsynonymous	NA	nonsynonymous

GLDC	chr9	6558684	6558684	C	A	NA	nonsynonymous	NA	nonsynonymous
HSPG2	chr1	22156044	22156044	G	A	NA	nonsynonymous	NA	NA
HTRA2	chr2	74757176	74757176	C	T	NA	nonsynonymous	NA	nonsynonymous
IGSF10	chr3	151171333	151171333	G	C	NA	nonsynonymous	NA	nonsynonymous
IRX2	chr5	2749505	2749505	C	T	NA	nonsynonymous	NA	nonsynonymous
ITGA8	chr10	15761727	15761727	G	A	NA	nonsynonymous	nonsynonymous	nonsynonymous
IVD	chr15	40703771	40703771	A	-	NA	frameshift_deletion	NA	frameshift_deletion
JADE3	chrX	46884248	46884248	G	T	NA	nonsynonymous	NA	NA
KLF10	chr8	103663899	103663899	C	G	NA	nonsynonymous	NA	nonsynonymous
LAMB3	chr1	209796004	209796004	C	G	NA	nonsynonymous	NA	nonsynonymous
LAMB3	chr1	209796010	209796010	C	-	NA	frameshift_deletion	NA	frameshift_deletion
LONRF3	chrX	118109005	118109005	G	A	NA	nonsynonymous	NA	nonsynonymous
LOXL3	chr2	74760771	74760771	C	T	NA	nonsynonymous	NA	nonsynonymous
LOXL3	chr2	74761064	74761064	C	T	NA	nonsynonymous	NA	nonsynonymous
MAP4	chr3	47957738	47957738	C	G	NA	nonsynonymous	NA	nonsynonymous
MAT2B	chr5	162939069	162939069	G	A	NA	nonsynonymous	NA	nonsynonymous
MAT2B	chr5	162939071	162939071	G	C	NA	nonsynonymous	NA	nonsynonymous
METTL2B	chr7	128119220	128119220	G	C	NA	nonsynonymous	NA	nonsynonymous
MGA	chr15	42005367	42005367	C	G	NA	nonsynonymous	NA	nonsynonymous
MTOR	chr1	11204766	11204766	T	A	NA	nonsynonymous	NA	nonsynonymous
MYBBP1A	chr17	4448066	4448066	G	A	NA	nonsynonymous	NA	nonsynonymous
MYH15	chr3	108174653	108174653	C	T	NA	nonsynonymous	NA	nonsynonymous
MYO18B	chr22	26422845	26422845	C	T	NA	nonsynonymous	NA	nonsynonymous
MYO5B	chr18	47438495	47438495	C	G	NA	nonsynonymous	NA	NA
MYO5C	chr15	52506805	52506805	C	G	NA	nonsynonymous	NA	NA
NFATC2	chr20	50092079	50092079	A	T	NA	nonsynonymous	NA	nonsynonymous
NME8	chr7	37936633	37936633	C	T	NA	nonsynonymous	NA	nonsynonymous
NOTCH2	chr1	120512148	120512148	G	A	NA	nonsynonymous	NA	nonsynonymous
NSD1	chr5	176720862	176720862	G	A	NA	nonsynonymous	NA	nonsynonymous
P3H2	chr3	189690735	189690735	C	G	NA	nonsynonymous	NA	nonsynonymous
PCDHB4	chr5	140503528	140503528	C	T	NA	nonsynonymous	NA	nonsynonymous
PIK3C2A	chr11	17190912	17190912	G	C	NA	nonsynonymous	NA	nonsynonymous
PIK3CA	chr3	178936091	178936091	G	A	NA	nonsynonymous	NA	NA
PIK3CA	chr3	178952074	178952074	G	A	NA	nonsynonymous	NA	nonsynonymous
PRPF3	chr1	150321638	150321638	G	A	NA	nonsynonymous	NA	nonsynonymous
PTK7	chr6	43111288	43111288	G	C	NA	nonsynonymous	NA	nonsynonymous
RPN1	chr3	128356694	128356694	G	C	NA	nonsynonymous	NA	nonsynonymous
RRP8	chr11	6621996	6621996	C	T	NA	nonsynonymous	NA	nonsynonymous
SBNO2	chr19	1112190	1112190	G	C	NA	nonsynonymous	NA	nonsynonymous
SETD5	chr3	9483919	9483924	CAAATG	-	NA	nonframeshift_deletion	NA	nonframeshift_deletion
SF3A1	chr22	30734942	30734942	C	T	NA	nonsynonymous	NA	nonsynonymous
SH3TC1	chr4	8239278	8239278	G	C	NA	nonsynonymous	NA	nonsynonymous
SIDT1	chr3	113327298	113327298	C	G	NA	nonsynonymous	NA	NA
SYNE1	chr6	152771911	152771911	C	G	NA	nonsynonymous	NA	NA
SYNPO2L	chr10	75408165	75408165	G	T	NA	nonsynonymous	NA	NA
TCTE1	chr6	44253898	44253898	C	G	NA	nonsynonymous	NA	nonsynonymous
TESMIN	chr11	68506210	68506210	G	C	NA	nonsynonymous	NA	nonsynonymous
TMEM120I	chr12	122213016	122213016	G	C	NA	nonsynonymous	NA	nonsynonymous
UHRF2	chr9	6475422	6475422	A	G	NA	nonsynonymous	NA	nonsynonymous
VPS13B	chr8	100443833	100443833	G	C	NA	nonsynonymous	NA	nonsynonymous
VTI1B	chr14	68126581	68126581	A	C	NA	nonsynonymous	NA	nonsynonymous
WDR97	chr8	145170871	145170871	G	A	NA	nonsynonymous	NA	nonsynonymous
WFDC3	chr20	44405795	44405795	C	T	NA	nonsynonymous	NA	nonsynonymous
WWC2	chr4	184182425	184182425	C	T	NA	nonsynonymous	NA	NA
ZBED6	chr1	203767008	203767008	G	A	NA	nonsynonymous	NA	nonsynonymous
ZHX1	chr8	124267234	124267234	G	A	NA	nonsynonymous	NA	nonsynonymous
ZKSCAN2	chr16	25258319	25258319	G	C	NA	nonsynonymous	NA	nonsynonymous
ZNF276	chr16	89789058	89789058	G	C	NA	nonsynonymous	NA	NA
ZNF710	chr15	90611490	90611490	C	T	NA	nonsynonymous	NA	nonsynonymous
ZNF736	chr7	63797373	63797373	G	C	NA	stoploss	NA	stoploss
ZNF804B	chr7	88965234	88965234	T	A	NA	nonsynonymous	NA	NA
ZNF91	chr19	23545393	23545393	C	A	NA	nonsynonymous	NA	nonsynonymous
ABCA2	chr9	139915920	139915920	G	T	NA	NA	nonsynonymous	NA
AKAP8L	chr19	15512317	15512317	C	G	NA	NA	nonsynonymous	NA
ALOXE3	chr17	8013532	8013532	C	T	NA	NA	nonsynonymous	NA
AMFR	chr16	56396945	56396945	T	C	NA	NA	nonsynonymous	NA
APC2	chr19	1466363	1466363	C	G	NA	NA	nonsynonymous	NA
ARL14EP	chr11	30352673	30352673	C	T	NA	NA	nonsynonymous	NA
ARVCF	chr22	19968769	19968769	C	A	NA	NA	nonsynonymous	NA
ASXL2	chr2	25973208	25973208	G	C	NA	NA	nonsynonymous	NA
ATP10D	chr4	47583985	47583985	G	T	NA	NA	nonsynonymous	NA
BCAT1	chr12	25054785	25054785	C	A	NA	NA	nonsynonymous	NA
C16orf89	chr16	5112536	5112536	G	T	NA	NA	nonsynonymous	NA
CCDC168	chr13	103396856	103396856	G	A	NA	NA	nonsynonymous	NA
CD83	chr6	14133927	14133927	G	A	NA	NA	nonsynonymous	NA
CDK5RAP1	chr20	31967470	31967470	G	A	NA	NA	stopgain	NA
CELSR3	chr3	48685714	48685714	C	A	NA	NA	nonsynonymous	NA
CENPJ	chr13	25479998	25479998	G	C	NA	NA	nonsynonymous	NA
CEP250	chr20	34091466	34091466	C	T	NA	NA	stopgain	NA
CHAF1A	chr19	4409685	4409685	G	A	NA	NA	nonsynonymous	NA
CNNM2	chr10	104679528	104679528	G	T	NA	NA	stopgain	NA
DCAF15	chr19	14069936	14069936	C	G	NA	NA	nonsynonymous	NA
DISP3	chr1	11594520	11594520	C	A	NA	NA	nonsynonymous	NA
DNAH2	chr17	7708701	7708701	G	C	NA	NA	nonsynonymous	NA
DNAH7	chr2	196834805	196834805	C	T	NA	NA	nonsynonymous	NA
DNMT3A	chr2	25470477	25470477	C	G	NA	NA	nonsynonymous	NA

DOT1L	chr19	2211201	2211201	G	C	NA	NA	nonsynonymous	NA
DROSHA	chr5	31508842	31508842	G	C	NA	NA	nonsynonymous	NA
DYM	chr18	46956762	46956762	C	A	NA	NA	nonsynonymous	NA
FAM161B	chr14	74416887	74416887	C	T	NA	NA	nonsynonymous	NA
FAT3	chr11	92534544	92534544	C	A	NA	NA	nonsynonymous	NA
FAXC	chr6	99729120	99729120	C	G	NA	NA	nonsynonymous	NA
FBXO34	chr14	55818016	55818016	C	G	NA	NA	stopgain	NA
FOXB2	chr9	79635322	79635322	C	T	NA	NA	nonsynonymous	NA
GAB4	chr22	17450865	17450865	C	A	NA	NA	nonsynonymous	NA
GCGR	chr17	79770933	79770933	G	T	NA	NA	nonsynonymous	NA
GIGYF1	chr7	100282700	100282700	T	C	NA	NA	nonsynonymous	NA
GUCY2C	chr12	14849235	14849235	G	T	NA	NA	nonsynonymous	NA
KCND2	chr7	119915236	119915236	G	A	NA	NA	nonsynonymous	NA
KCND2	chr7	119915237	119915237	-	A	NA	NA	frameshift_insertion	NA
KIAA1109	chr4	123091817	123091817	G	C	NA	NA	nonsynonymous	NA
KIAA1324L	chr7	86541467	86541467	G	A	NA	NA	nonsynonymous	NA
KMT2D	chr12	49431346	49431346	G	T	NA	NA	nonsynonymous	NA
KSR2	chr12	117909083	117909083	C	G	NA	NA	nonsynonymous	NA
KSR2	chr12	117909085	117909085	T	G	NA	NA	nonsynonymous	NA
LINGO4	chr1	151774756	151774756	T	-	NA	NA	frameshift_deletion	NA
LPO	chr17	56342198	56342198	C	A	NA	NA	nonsynonymous	NA
MAEL	chr1	166985516	166985516	G	T	NA	NA	nonsynonymous	NA
MEP1A	chr6	46777237	46777237	G	A	NA	NA	nonsynonymous	NA
MMP25	chr16	3107591	3107591	G	C	NA	NA	nonsynonymous	NA
MORC2	chr22	31335957	31335957	G	A	NA	NA	nonsynonymous	nonsynonymous
MUC1	chr1	155160889	155160889	G	C	NA	NA	stopgain	NA
MYH4	chr17	10369962	10369962	G	A	NA	NA	nonsynonymous	NA
NDST4	chr4	115773881	115773881	C	T	NA	NA	nonsynonymous	NA
NSUN2	chr5	6632754	6632754	C	G	NA	NA	nonsynonymous	NA
OR2W3	chr1	248059396	248059396	G	A	NA	NA	nonsynonymous	NA
OVCH1	chr12	29614894	29614894	C	G	NA	NA	nonsynonymous	NA
PAK5	chr20	9523328	9523328	T	A	NA	NA	nonsynonymous	NA
PCNT	chr21	47775428	47775428	T	A	NA	NA	nonsynonymous	NA
PI3	chr20	43804645	43804645	T	C	NA	NA	nonsynonymous	NA
PINLYP	chr19	44081369	44081369	C	T	NA	NA	nonsynonymous	NA
PPP3CB	chr10	75239079	75239079	G	C	NA	NA	nonsynonymous	NA
PRM2	chr16	11369978	11369978	G	A	NA	NA	nonsynonymous	NA
PTPA	chr9	131890336	131890336	G	C	NA	NA	nonsynonymous	NA
PTPRB	chr12	70928645	70928645	C	G	NA	NA	nonsynonymous	NA
RAB11FIP5	chr2	73316207	73316207	C	A	NA	NA	nonsynonymous	NA
RNF150	chr4	141847208	141847208	A	T	NA	NA	nonsynonymous	NA
RTL6	chr22	44892765	44892765	G	-	NA	NA	frameshift_deletion	NA
SCN5A	chr3	38618251	38618251	C	G	NA	NA	nonsynonymous	NA
1-Sep	chr16	30393495	30393495	A	G	NA	NA	nonsynonymous	NA
SLC25A24	chr1	108697712	108697712	G	A	NA	NA	stopgain	NA
SLC2A2	chr3	170723209	170723209	C	T	NA	NA	nonsynonymous	NA
SLC35B3	chr6	8422853	8422853	G	C	NA	NA	nonsynonymous	NA
SLC43A1	chr11	57268673	57268673	C	T	NA	NA	nonsynonymous	NA
SLC4A1AP	chr2	27898470	27898470	G	C	NA	NA	nonsynonymous	NA
SLC5A9	chr1	48696322	48696322	C	A	NA	NA	nonsynonymous	NA
SLC7A1	chr13	30091745	30091745	G	C	NA	NA	nonsynonymous	NA
SOX17	chr8	55370901	55370901	T	A	NA	NA	nonsynonymous	NA
TGFB2	chr1	218536715	218536715	G	T	NA	NA	nonsynonymous	NA
TRIM42	chr3	140401376	140401376	T	G	NA	NA	nonsynonymous	NA
TSPAN12	chr7	120480132	120480132	G	T	NA	NA	nonsynonymous	NA
TTN	chr2	179638452	179638452	C	A	NA	NA	nonsynonymous	NA
UQCR10	chr22	30163496	30163496	G	C	NA	NA	nonsynonymous	NA
XDH	chr2	31565149	31565149	C	G	NA	NA	nonsynonymous	NA
ZKSCAN2	chr16	25268361	25268361	C	A	NA	NA	nonsynonymous	NA
ZNF670	chr1	247202765	247202765	C	G	NA	NA	nonsynonymous	NA
AASS	chr7	121773587	121773587	C	G	NA	NA	NA	nonsynonymous
ALDH1L1	chr3	125836906	125836906	C	T	NA	NA	NA	nonsynonymous
CDCA5	chr11	64851136	64851136	C	T	NA	NA	NA	nonsynonymous
CEBPZ	chr2	37439040	37439040	C	T	NA	NA	NA	nonsynonymous
CTDP1	chr18	77474644	77474644	C	T	NA	NA	NA	nonsynonymous
DSTYK	chr1	205133018	205133018	T	G	NA	NA	NA	nonsynonymous
FAM183A	chr1	43621928	43621928	G	A	NA	NA	NA	nonsynonymous
GGA3	chr17	73236075	73236075	G	C	NA	NA	NA	nonsynonymous
GPRIN1	chr5	176026777	176026777	G	T	NA	NA	NA	nonsynonymous
HES3	chr1	6305297	6305297	G	C	NA	NA	NA	nonsynonymous
KALRN	chr3	124132373	124132373	C	G	NA	NA	NA	nonsynonymous
MICAL3	chr22	18300332	18300332	T	A	NA	NA	NA	nonsynonymous
OR5A1	chr11	59211279	59211279	C	T	NA	NA	NA	nonsynonymous
PADI4	chr1	17634754	17634754	C	T	NA	NA	NA	stopgain
PRPF8	chr17	1558704	1558704	C	A	NA	NA	NA	nonsynonymous
RABEP1	chr17	5280498	5280498	G	C	NA	NA	NA	nonsynonymous
RASGRP4	chr19	38904057	38904057	C	G	NA	NA	NA	nonsynonymous
RPS2	chr16	2014325	2014325	C	T	NA	NA	NA	nonsynonymous
SCRN1	chr7	29980423	29980423	C	T	NA	NA	NA	nonsynonymous
SIAE	chr11	124506862	124506862	G	T	NA	NA	NA	nonsynonymous
STAB1	chr3	52539068	52539068	A	G	NA	NA	NA	nonsynonymous
TEX15	chr8	30705357	30705357	C	G	NA	NA	NA	nonsynonymous
TRAFD1	chr12	112572654	112572654	C	T	NA	NA	NA	nonsynonymous
TRO	chrX	54955965	54955965	T	G	NA	NA	NA	nonsynonymous
TXLNB	chr6	139583760	139583760	C	T	NA	NA	NA	nonsynonymous

Gene	chromosome	Start	Stop	Ref	Alt	HK14-TA	HK14-TB	HK14-TC	HK14-TD
AARD	chr8	117954933	117954933	C	T	nonsynonymous	nonsynonymous	nonsynonymous	nonsynonymous
ADGRV1	chr5	90261296	90261296	C	A	nonsynonymous	nonsynonymous	nonsynonymous	nonsynonymous
ANKRD24	chr19	4216668	4216668	C	T	nonsynonymous	nonsynonymous	nonsynonymous	nonsynonymous
ARFGEF3	chr6	138584580	138584580	C	T	stopgain	stopgain	stopgain	stopgain
C4orf17	chr4	100443822	100443822	C	G	nonsynonymous	nonsynonymous	nonsynonymous	nonsynonymous
C8orf76;ZH	chr8	124243832	124243832	G	C	nonsynonymous	nonsynonymous	nonsynonymous	nonsynonymous
CD163L1	chr12	7586008	7586008	T	C	nonsynonymous	nonsynonymous	nonsynonymous	nonsynonymous
CTSA	chr20	44520586	44520586	A	T	nonsynonymous	nonsynonymous	nonsynonymous	nonsynonymous
DMD	chrX	32509516	32509516	C	T	nonsynonymous	nonsynonymous	nonsynonymous	nonsynonymous
DRD2	chr11	113281481	113281481	G	A	nonsynonymous	nonsynonymous	nonsynonymous	nonsynonymous
EIF2AK4	chr15	40235651	40235651	C	T	nonsynonymous	NA	NA	NA
EPB41L1	chr20	34713438	34713438	C	T	nonsynonymous	nonsynonymous	nonsynonymous	nonsynonymous
ESR1	chr6	152265323	152265323	G	A	nonsynonymous	nonsynonymous	nonsynonymous	nonsynonymous
FAT3	chr11	92086741	92086741	G	C	nonsynonymous	nonsynonymous	nonsynonymous	nonsynonymous
FBXW7	chr4	153249393	153249393	G	A	nonsynonymous	nonsynonymous	nonsynonymous	nonsynonymous
FGF6	chr12	4554552	4554552	C	T	nonsynonymous	nonsynonymous	nonsynonymous	nonsynonymous
GIMAP7	chr7	150217566	150217566	C	A	nonsynonymous	NA	nonsynonymous	nonsynonymous
GRIK4	chr11	120833321	120833321	C	T	stopgain	stopgain	stopgain	stopgain
HS6ST2	chrX	132091041	132091041	G	A	nonsynonymous	nonsynonymous	nonsynonymous	nonsynonymous
IL18R1	chr2	103006566	103006566	G	A	nonsynonymous	nonsynonymous	nonsynonymous	nonsynonymous
INF2	chr14	105174271	105174271	G	A	nonsynonymous	nonsynonymous	nonsynonymous	nonsynonymous
ITGA11	chr15	68650822	68650822	A	C	nonsynonymous	NA	nonsynonymous	nonsynonymous
KCNV1	chr8	110984838	110984838	C	T	nonsynonymous	nonsynonymous	nonsynonymous	nonsynonymous
KIF26A	chr14	104642826	104642826	G	A	nonsynonymous	nonsynonymous	nonsynonymous	nonsynonymous
KIF2B	chr17	51901296	51901296	C	A	nonsynonymous	nonsynonymous	nonsynonymous	nonsynonymous
KLHL8	chr4	88084715	88084715	G	A	stopgain	stopgain	stopgain	stopgain
KRT15	chr17	39674835	39674846	CCAAAGC CTCCA	-	nonframeshift_deletion	nonframeshift_deletion	nonframeshift_deletion	nonframeshift_deletion
LAMA1	chr18	7037660	7037660	C	G	nonsynonymous	nonsynonymous	nonsynonymous	nonsynonymous
LGR6	chr1	202245623	202245623	C	-	frameshift_deletion	frameshift_deletion	frameshift_deletion	frameshift_deletion
MAGI2	chr7	77797326	77797326	C	T	nonsynonymous	nonsynonymous	nonsynonymous	NA
MDGA2	chr14	47770669	47770669	C	T	nonsynonymous	nonsynonymous	nonsynonymous	nonsynonymous
MUC5B	chr11	1269917	1269917	C	A	nonsynonymous	nonsynonymous	nonsynonymous	nonsynonymous
MYH2	chr17	10432234	10432234	G	A	nonsynonymous	nonsynonymous	nonsynonymous	nonsynonymous
NFASC	chr1	204943900	204943900	G	A	nonsynonymous	nonsynonymous	nonsynonymous	nonsynonymous
OR10R2	chr1	158450491	158450491	C	A	nonsynonymous	nonsynonymous	nonsynonymous	nonsynonymous
OR56A4	chr11	6023781	6023781	C	T	nonsynonymous	nonsynonymous	nonsynonymous	nonsynonymous
PDCD11	chr10	105173844	105173844	G	A	nonsynonymous	nonsynonymous	nonsynonymous	nonsynonymous
PIK3CA	chr3	178928076	178928084	TTAGAAG AT	-	nonframeshift_deletion	nonframeshift_deletion	nonframeshift_deletion	nonframeshift_deletion
PPP3CB	chr10	75230684	75230684	A	C	nonsynonymous	nonsynonymous	nonsynonymous	nonsynonymous
PPP6R3	chr11	68343437	68343437	G	A	nonsynonymous	nonsynonymous	nonsynonymous	nonsynonymous
PTPN3	chr9	112153824	112153824	-	T	frameshift_insertion	frameshift_insertion	frameshift_insertion	frameshift_insertion
SAMD4A	chr14	55227107	55227107	G	A	nonsynonymous	nonsynonymous	nonsynonymous	NA
SIM1	chr6	100841414	100841414	C	G	nonsynonymous	nonsynonymous	nonsynonymous	nonsynonymous
SIMC1	chr5	175740736	175740736	G	A	nonsynonymous	nonsynonymous	nonsynonymous	nonsynonymous
SMCHD1	chr18	2700638	2700638	C	A	nonsynonymous	nonsynonymous	nonsynonymous	nonsynonymous
SMCHD1	chr18	2728472	2728472	-	AAGTG	frameshift_insertion	frameshift_insertion	frameshift_insertion	frameshift_insertion
SORCS3	chr10	106849582	106849582	C	T	nonsynonymous	nonsynonymous	nonsynonymous	nonsynonymous
SRRM2	chr16	2816369	2816369	-	C	frameshift_insertion	NA	frameshift_insertion	frameshift_insertion
SRRM2	chr16	2816374	2816382	AGAACTC CA	-	nonframeshift_deletion	NA	nonframeshift_deletion	nonframeshift_deletion
ST6GALNA(	chr17	74625381	74625381	-	T	frameshift_insertion	frameshift_insertion	frameshift_insertion	frameshift_insertion
SVEP1	chr9	113221366	113221366	C	T	nonsynonymous	nonsynonymous	nonsynonymous	nonsynonymous
TBXT	chr6	166581055	166581055	C	T	nonsynonymous	nonsynonymous	nonsynonymous	nonsynonymous
TEAD1	chr11	12958705	12958708	TGTT	-	frameshift_deletion	NA	frameshift_deletion	frameshift_deletion
TEAD3	chr6	35454354	35454365	TTGTCCA GCCCC	-	nonframeshift_deletion	nonframeshift_deletion	nonframeshift_deletion	nonframeshift_deletion
TNFSF14	chr19	6664954	6664954	C	T	nonsynonymous	nonsynonymous	NA	nonsynonymous
TPST1	chr7	65705513	65705513	G	A	nonsynonymous	nonsynonymous	nonsynonymous	nonsynonymous
TTN	chr2	179484974	179484974	C	T	nonsynonymous	nonsynonymous	nonsynonymous	nonsynonymous
USH2A	chr1	215972357	215972357	C	A	nonsynonymous	nonsynonymous	nonsynonymous	nonsynonymous
WFDC5	chr20	43739055	43739055	C	T	nonsynonymous	nonsynonymous	nonsynonymous	nonsynonymous
XRN1	chr3	142030460	142030460	A	C	nonsynonymous	nonsynonymous	nonsynonymous	nonsynonymous
KIAA1257	chr3	128711936	128711936	T	C	NA	nonsynonymous	NA	NA
MYO7A	chr11	76885856	76885856	A	G	NA	nonsynonymous	nonsynonymous	nonsynonymous
SEC14L6	chr22	30921495	30921495	G	A	NA	nonsynonymous	NA	NA
XKR4	chr8	56270263	56270263	A	G	NA	nonsynonymous	NA	NA
ZNF93	chr19	20044278	20044278	C	A	NA	nonsynonymous	NA	NA
EMID1	chr22	29654917	29654917	G	T	NA	NA	nonsynonymous	NA
NKX2-5	chr5	172660452	172660452	C	T	NA	NA	nonsynonymous	nonsynonymous
CCDC178	chr18	30795599	30795599	A	-	NA	NA	NA	frameshift_deletion

Gene	chromosome	Start	Stop	Ref	Alt	HK15-TA	HK15-TB	HK15-TC	HK15-TD
ABCA1	chr9	107593321	107593321	A	G	nonsynonymous	NA	NA	NA
ABCC2	chr10	101569949	101569949	C	G	nonsynonymous	nonsynonymous	NA	nonsynonymous
ACSM4	chr12	7463248	7463248	G	T	stopgain	stopgain	NA	stopgain
ADAD2	chr16	84229907	84229907	G	A	nonsynonymous	nonsynonymous	NA	nonsynonymous
ADAM11	chr17	42854285	42854285	C	T	nonsynonymous	nonsynonymous	nonsynonymous	nonsynonymous
ADAMTS5	chr21	28338641	28338641	C	T	nonsynonymous	nonsynonymous	nonsynonymous	nonsynonymous
ADCK1	chr14	78374152	78374152	C	T	stopgain	stopgain	stopgain	stopgain
ADGRE5	chr19	14507264	14507264	G	C	nonsynonymous	nonsynonymous	nonsynonymous	nonsynonymous
ADPGK	chr15	73048712	73048712	C	T	nonsynonymous	nonsynonymous	NA	nonsynonymous
AFF3	chr2	100210291	100210291	G	A	nonsynonymous	nonsynonymous	nonsynonymous	nonsynonymous
AGTR1	chr3	148459805	148459805	C	T	nonsynonymous	nonsynonymous	nonsynonymous	nonsynonymous
AHCYL1	chr1	110561698	110561698	C	T	nonsynonymous	nonsynonymous	nonsynonymous	nonsynonymous
AIM2	chr1	159038366	159038366	G	A	nonsynonymous	nonsynonymous	nonsynonymous	nonsynonymous
AKAP6	chr14	32902965	32902965	A	G	nonsynonymous	nonsynonymous	nonsynonymous	nonsynonymous
ALKBH4	chr7	102098029	102098029	G	C	nonsynonymous	nonsynonymous	nonsynonymous	nonsynonymous
ANKDD1A	chr15	65235771	65235771	G	C	nonsynonymous	nonsynonymous	nonsynonymous	nonsynonymous
ANP32A	chr15	69079733	69079758	GAGGGTC CGAATGA CTTACCA GTGGC	-	frameshift_deletion	frameshift_deletion	NA	frameshift_deletion
AP1M1	chr19	16317206	16317206	A	G	nonsynonymous	nonsynonymous	nonsynonymous	nonsynonymous
APOBR	chr16	28509153	28509153	G	C	nonsynonymous	nonsynonymous	nonsynonymous	nonsynonymous
ARFGAP3	chr22	43243586	43243586	C	G	nonsynonymous	nonsynonymous	nonsynonymous	nonsynonymous
ARHGEF15	chr17	8215587	8215587	C	T	nonsynonymous	nonsynonymous	nonsynonymous	nonsynonymous
ARL6IP4	chr12	123465031	123465031	G	C	nonsynonymous	nonsynonymous	NA	nonsynonymous
ASCC2	chr22	30209468	30209468	T	C	nonsynonymous	nonsynonymous	nonsynonymous	nonsynonymous
ATP12A	chr13	25280464	25280464	G	A	nonsynonymous	nonsynonymous	nonsynonymous	nonsynonymous
ATP2C2	chr16	84495386	84495386	G	A	nonsynonymous	nonsynonymous	nonsynonymous	nonsynonymous
ATRN	chr20	3452043	3452043	G	A	nonsynonymous	nonsynonymous	NA	nonsynonymous
BIN3	chr8	22487992	22487992	G	A	nonsynonymous	nonsynonymous	nonsynonymous	nonsynonymous
BLZF1	chr1	169349732	169349732	G	A	nonsynonymous	nonsynonymous	nonsynonymous	nonsynonymous
BRCA1	chr17	41246810	41246810	C	G	nonsynonymous	nonsynonymous	NA	nonsynonymous
BRIP1	chr17	59821871	59821871	G	T	nonsynonymous	nonsynonymous	nonsynonymous	nonsynonymous
C16orf96	chr16	4638303	4638303	G	C	nonsynonymous	nonsynonymous	nonsynonymous	nonsynonymous
C1orf52	chr1	85725307	85725307	C	A	stopgain	stopgain	NA	stopgain
C3	chr19	6694559	6694559	G	T	nonsynonymous	nonsynonymous	nonsynonymous	nonsynonymous
C6orf120	chr6	170103093	170103093	A	G	nonsynonymous	nonsynonymous	nonsynonymous	nonsynonymous
CASP8	chr2	202136277	202136277	C	G	stopgain	stopgain	stopgain	stopgain
CD160	chr1	145704257	145704257	C	T	nonsynonymous	nonsynonymous	nonsynonymous	nonsynonymous
CD44	chr11	35218394	35218394	C	G	nonsynonymous	nonsynonymous	nonsynonymous	nonsynonymous
CDKL2	chr4	76522194	76522194	G	T	nonsynonymous	nonsynonymous	nonsynonymous	nonsynonymous
CEACAM20	chr19	45029263	45029263	C	T	nonsynonymous	nonsynonymous	nonsynonymous	nonsynonymous
CHFR	chr12	133463835	133463835	C	T	nonsynonymous	NA	NA	nonsynonymous
CHMP2A	chr19	59063805	59063805	C	G	nonsynonymous	nonsynonymous	nonsynonymous	nonsynonymous
CHRNA1	chr2	175613330	175613330	T	G	nonsynonymous	nonsynonymous	NA	nonsynonymous
CLEC1B	chr12	10149466	10149466	C	G	nonsynonymous	nonsynonymous	nonsynonymous	nonsynonymous
CLP1	chr11	57428744	57428744	G	C	nonsynonymous	nonsynonymous	NA	nonsynonymous
CMYA5	chr5	79028394	79028394	G	C	nonsynonymous	nonsynonymous	nonsynonymous	nonsynonymous
COL24A1	chr1	86426958	86426958	C	G	nonsynonymous	nonsynonymous	nonsynonymous	nonsynonymous
CSMD1	chr8	3205531	3205531	C	T	nonsynonymous	nonsynonymous	NA	nonsynonymous
CUL7	chr6	43014110	43014110	C	T	nonsynonymous	nonsynonymous	nonsynonymous	nonsynonymous
DCXR	chr17	79994491	79994491	C	G	nonsynonymous	NA	NA	NA
DMD	chrX	31496249	31496249	G	C	nonsynonymous	nonsynonymous	nonsynonymous	nonsynonymous
DNAH14	chr1	225267081	225267081	C	T	nonsynonymous	nonsynonymous	nonsynonymous	nonsynonymous
DNAH5	chr5	13866343	13866343	G	T	nonsynonymous	nonsynonymous	NA	nonsynonymous
DNAH5	chr5	13901455	13901455	G	A	nonsynonymous	nonsynonymous	nonsynonymous	nonsynonymous
DNAH7	chr2	196741339	196741339	G	C	nonsynonymous	nonsynonymous	NA	nonsynonymous
DNAJB5	chr9	34990754	34990754	C	T	stopgain	stopgain	stopgain	stopgain
DNAJB5	chr9	34996514	34996514	G	A	nonsynonymous	nonsynonymous	nonsynonymous	nonsynonymous
DNAJC6	chr1	65830441	65830441	C	T	nonsynonymous	nonsynonymous	nonsynonymous	nonsynonymous
DOPEY1	chr6	83839919	83839919	C	T	stopgain	stopgain	stopgain	stopgain
DSC3	chr18	28602418	28602418	T	C	nonsynonymous	nonsynonymous	nonsynonymous	nonsynonymous
DTNB	chr2	25705696	25705696	T	C	nonsynonymous	nonsynonymous	nonsynonymous	nonsynonymous
DZIP1L	chr3	137800591	137800591	G	C	nonsynonymous	nonsynonymous	nonsynonymous	nonsynonymous
E4F1	chr16	2283931	2283931	C	T	stopgain	stopgain	stopgain	stopgain
EGFLAM	chr5	38337630	38337630	G	C	nonsynonymous	nonsynonymous	NA	nonsynonymous
EHMT1	chr9	140708918	140708918	C	G	nonsynonymous	nonsynonymous	nonsynonymous	nonsynonymous
EIF4E1B	chr5	176071437	176071437	C	G	nonsynonymous	nonsynonymous	nonsynonymous	nonsynonymous
ELP2	chr18	33750155	33750155	C	G	nonsynonymous	nonsynonymous	NA	nonsynonymous
EME1	chr17	48458130	48458130	C	G	nonsynonymous	nonsynonymous	nonsynonymous	nonsynonymous
ENDOD1	chr11	94861807	94861807	G	T	nonsynonymous	nonsynonymous	nonsynonymous	nonsynonymous
ENOSF1	chr18	677352	677352	C	G	nonsynonymous	nonsynonymous	nonsynonymous	nonsynonymous
EPHA2	chr1	16475273	16475273	C	G	nonsynonymous	nonsynonymous	NA	nonsynonymous
ERICH6B	chr13	46135584	46135584	C	A	stopgain	stopgain	stopgain	stopgain
ESRRG	chr1	216850689	216850689	G	T	nonsynonymous	nonsynonymous	nonsynonymous	nonsynonymous
ESS2	chr22	19124893	19124893	C	G	nonsynonymous	nonsynonymous	nonsynonymous	nonsynonymous
FAM214B	chr9	35107992	35107992	C	T	nonsynonymous	nonsynonymous	nonsynonymous	nonsynonymous
FANCC	chr9	97864103	97864103	C	G	nonsynonymous	nonsynonymous	nonsynonymous	nonsynonymous
FARP1	chr13	99076875	99076875	G	A	nonsynonymous	nonsynonymous	nonsynonymous	nonsynonymous
FAT1	chr4	187549845	187549845	-	A	frameshift_insertion	frameshift_insertion	frameshift_insertion	frameshift_insertion
FBN1	chr15	48733944	48733944	A	G	nonsynonymous	nonsynonymous	NA	nonsynonymous
FBXL20	chr17	37420440	37420440	G	C	nonsynonymous	nonsynonymous	nonsynonymous	nonsynonymous
FCRL6	chr1	159779007	159779007	G	C	nonsynonymous	nonsynonymous	nonsynonymous	nonsynonymous
FCRLB	chr1	161697121	161697121	G	C	nonsynonymous	nonsynonymous	nonsynonymous	nonsynonymous

FICD	chr12	108912382	108912382	C	G	nonsynonymous	nonsynonymous	nonsynonymous	nonsynonymous
FLNC	chr7	128482740	128482740	G	A	nonsynonymous	nonsynonymous	nonsynonymous	nonsynonymous
FOXD3	chr1	63788805	63788805	G	C	nonsynonymous	nonsynonymous	NA	nonsynonymous
G6PC3	chr17	42151565	42151565	G	C	nonsynonymous	nonsynonymous	nonsynonymous	nonsynonymous
GABRG1	chr4	46067466	46067466	A	C	nonsynonymous	nonsynonymous	nonsynonymous	nonsynonymous
GAL3ST4	chr7	99757893	99757893	G	C	nonsynonymous	nonsynonymous	nonsynonymous	nonsynonymous
GAREM1	chr18	29847925	29847925	G	A	nonsynonymous	nonsynonymous	nonsynonymous	nonsynonymous
GEMIN5	chr5	154308214	154308214	T	A	nonsynonymous	nonsynonymous	nonsynonymous	nonsynonymous
GGCX	chr2	85785696	85785696	G	A	nonsynonymous	nonsynonymous	nonsynonymous	nonsynonymous
GLG1	chr16	74640700	74640700	G	A	nonsynonymous	nonsynonymous	NA	nonsynonymous
GOLIM4	chr3	167762627	167762627	G	C	nonsynonymous	nonsynonymous	nonsynonymous	nonsynonymous
GOSR2	chr17	45008521	45008521	C	G	nonsynonymous	nonsynonymous	nonsynonymous	nonsynonymous
GPR179	chr17	36482700	36482700	G	A	nonsynonymous	nonsynonymous	NA	nonsynonymous
GRID1	chr10	87484301	87484303	AGA	-	nonframeshift_deletion	nonframeshift_deletion	nonframeshift_deletion	nonframeshift_deletion
GRM6	chr5	178413391	178413391	C	T	nonsynonymous	nonsynonymous	nonsynonymous	nonsynonymous
GUCD1	chr22	24951747	24951747	C	G	nonsynonymous	nonsynonymous	nonsynonymous	nonsynonymous
HIST1H2BF	chr6	26199996	26199996	C	G	nonsynonymous	nonsynonymous	nonsynonymous	nonsynonymous
HIST1H2BG	chr6	26216692	26216692	C	T	nonsynonymous	nonsynonymous	NA	nonsynonymous
HIST1H4K	chr6	27798995	27798995	C	G	stoploss	stoploss	stoploss	stoploss
HOXD9	chr2	176987680	176987680	G	T	stopgain	stopgain	stopgain	stopgain
HSPA14	chr10	14909289	14909289	G	C	nonsynonymous	nonsynonymous	NA	nonsynonymous
HUWE1	chrX	53600793	53600793	C	G	nonsynonymous	nonsynonymous	nonsynonymous	nonsynonymous
IGFN1	chr1	201179173	201179173	G	T	stopgain	stopgain	stopgain	stopgain
IGSF10	chr3	151156003	151156003	C	G	nonsynonymous	nonsynonymous	nonsynonymous	nonsynonymous
IGSF5	chr21	41137622	41137622	C	G	nonsynonymous	nonsynonymous	nonsynonymous	nonsynonymous
IL18BP	chr11	71712660	71712660	G	T	nonsynonymous	nonsynonymous	nonsynonymous	nonsynonymous
IRAK1BP1	chr6	79607570	79607570	G	-	frameshift_deletion	frameshift_deletion	frameshift_deletion	frameshift_deletion
JPH3	chr16	87723536	87723536	G	A	nonsynonymous	nonsynonymous	NA	nonsynonymous
KCNG1	chr20	49621055	49621055	G	A	nonsynonymous	nonsynonymous	nonsynonymous	nonsynonymous
KIAA0825	chr5	93805704	93805704	C	T	nonsynonymous	nonsynonymous	nonsynonymous	nonsynonymous
KIF26A	chr14	104642460	104642460	C	T	nonsynonymous	nonsynonymous	NA	nonsynonymous
KIF4B	chr5	154396579	154396579	G	C	nonsynonymous	nonsynonymous	nonsynonymous	nonsynonymous
KMT2E	chr7	104752569	104752577	CCTCACACA	-	nonframeshift_deletion	nonframeshift_deletion	nonframeshift_deletion	nonframeshift_deletion
KMT2E	chr7	104752582	104752588	TACAGCA	-	frameshift_deletion	frameshift_deletion	frameshift_deletion	frameshift_deletion
LIPT1	chr2	99778565	99778565	G	C	nonsynonymous	nonsynonymous	NA	nonsynonymous
LRP1	chr12	57578943	57578943	G	A	nonsynonymous	nonsynonymous	nonsynonymous	nonsynonymous
LRP2	chr2	170094609	170094609	T	A	stopgain	stopgain	stopgain	stopgain
LRRC66	chr4	52861615	52861615	G	C	nonsynonymous	nonsynonymous	NA	nonsynonymous
LYN	chr8	56879403	56879403	C	T	nonsynonymous	nonsynonymous	nonsynonymous	nonsynonymous
MAP3K9	chr14	71267608	71267608	T	C	nonsynonymous	nonsynonymous	nonsynonymous	nonsynonymous
MBLAC1	chr7	99725409	99725409	C	G	nonsynonymous	nonsynonymous	nonsynonymous	nonsynonymous
MED16	chr19	868885	868885	C	T	nonsynonymous	NA	nonsynonymous	nonsynonymous
MFSD6	chr2	191300779	191300779	C	G	nonsynonymous	nonsynonymous	NA	nonsynonymous
MIGA2	chr9	131812240	131812240	G	C	nonsynonymous	nonsynonymous	nonsynonymous	nonsynonymous
MINDY1	chr1	150972995	150972995	C	T	nonsynonymous	nonsynonymous	nonsynonymous	nonsynonymous
MTERF2	chr12	107372404	107372404	A	-	stopgain	stopgain	stopgain	stopgain
MYF5	chr12	81111053	81111053	G	A	nonsynonymous	nonsynonymous	nonsynonymous	nonsynonymous
MYH4	chr17	10355471	10355471	G	C	nonsynonymous	nonsynonymous	nonsynonymous	nonsynonymous
MYOD1	chr11	17741717	17741717	G	A	nonsynonymous	nonsynonymous	nonsynonymous	nonsynonymous
NAV2	chr11	20127131	20127131	C	G	nonsynonymous	nonsynonymous	NA	nonsynonymous
NEK5	chr13	52661581	52661581	G	C	nonsynonymous	nonsynonymous	nonsynonymous	nonsynonymous
NMBR	chr6	142409507	142409507	C	A	nonsynonymous	nonsynonymous	nonsynonymous	nonsynonymous
NOTCH1	chr9	139404213	139404214	CA	-	frameshift_deletion	frameshift_deletion	frameshift_deletion	frameshift_deletion
NOTCH1	chr9	139409055	139409055	G	A	nonsynonymous	nonsynonymous	nonsynonymous	nonsynonymous
NOX3	chr6	155717970	155717970	C	G	stoploss	stoploss	stoploss	stoploss
OR10J1	chr1	159409634	159409634	C	G	nonsynonymous	nonsynonymous	nonsynonymous	nonsynonymous
OR4K14	chr14	20482734	20482734	G	C	nonsynonymous	nonsynonymous	nonsynonymous	nonsynonymous
OR5K2	chr3	98216555	98216555	G	C	nonsynonymous	nonsynonymous	nonsynonymous	nonsynonymous
OR5K2	chr3	98216786	98216786	G	C	nonsynonymous	nonsynonymous	nonsynonymous	nonsynonymous
PDCD11	chr10	105174056	105174056	A	G	nonsynonymous	nonsynonymous	nonsynonymous	nonsynonymous
PDE4DIP	chr1	144930670	144930670	T	C	nonsynonymous	nonsynonymous	NA	nonsynonymous
PDZD2	chr5	32089255	32089255	C	T	nonsynonymous	nonsynonymous	nonsynonymous	nonsynonymous
PIGC	chr1	172411349	172411368	CTTCAGCACTGGTGAAAACC	-	frameshift_deletion	frameshift_deletion	frameshift_deletion	frameshift_deletion
PIK3C2G	chr12	18435649	18435649	C	T	nonsynonymous	nonsynonymous	nonsynonymous	nonsynonymous
PKHD1L1	chr8	110477300	110477300	G	C	nonsynonymous	nonsynonymous	NA	nonsynonymous
PLD5	chr1	242271130	242271130	G	C	nonsynonymous	nonsynonymous	NA	nonsynonymous
PLEC	chr8	144997418	144997418	C	A	stopgain	stopgain	stopgain	stopgain
PLPPR3	chr19	814693	814693	G	A	nonsynonymous	nonsynonymous	nonsynonymous	nonsynonymous
PLXNA3	chrX	153696779	153696779	C	T	nonsynonymous	nonsynonymous	nonsynonymous	nonsynonymous
PNPT1	chr2	55912099	55912099	T	C	nonsynonymous	nonsynonymous	nonsynonymous	nonsynonymous
PTPRO	chr12	15652456	15652456	G	C	nonsynonymous	nonsynonymous	nonsynonymous	nonsynonymous
RAP1GAP2	chr17	2888283	2888283	C	G	nonsynonymous	nonsynonymous	nonsynonymous	nonsynonymous
RAP1GAP2	chr17	2898752	2898752	G	A	nonsynonymous	nonsynonymous	nonsynonymous	nonsynonymous
RASGEF1C	chr5	179529117	179529117	C	A	stopgain	stopgain	stopgain	stopgain
RBM27	chr5	145649089	145649089	A	G	nonsynonymous	nonsynonymous	NA	nonsynonymous
REV1	chr2	100055386	100055386	G	T	nonsynonymous	nonsynonymous	nonsynonymous	nonsynonymous
RGS9	chr17	63221411	63221411	C	G	nonsynonymous	nonsynonymous	nonsynonymous	nonsynonymous
RLF	chr1	40661412	40661412	C	A	nonsynonymous	nonsynonymous	nonsynonymous	nonsynonymous
RNF213	chr17	78343632	78343632	C	G	nonsynonymous	nonsynonymous	nonsynonymous	nonsynonymous
RNF34	chr12	121858084	121858084	G	A	nonsynonymous	nonsynonymous	nonsynonymous	nonsynonymous
RORB	chr9	77286800	77286800	G	A	nonsynonymous	nonsynonymous	nonsynonymous	nonsynonymous

RPF2	chr6	111318422	111318422	A	T	nonsynonymous	nonsynonymous	nonsynonymous	nonsynonymous
RRP12	chr10	99160190	99160190	C	T	nonsynonymous	nonsynonymous	nonsynonymous	nonsynonymous
RRP7A	chr22	42914058	42914058	G	T	nonsynonymous	nonsynonymous	nonsynonymous	nonsynonymous
RYR2	chr1	237753261	237753261	C	A	nonsynonymous	nonsynonymous	nonsynonymous	nonsynonymous
SAMD8	chr10	76936369	76936369	A	T	nonsynonymous	nonsynonymous	nonsynonymous	nonsynonymous
SEC23IP	chr10	121662457	121662457	C	G	stopgain	stopgain	stopgain	stopgain
SEMA3A	chr7	83634847	83634847	C	G	nonsynonymous	nonsynonymous	nonsynonymous	nonsynonymous
SEZ6L	chr22	26776239	26776239	G	C	nonsynonymous	nonsynonymous	nonsynonymous	nonsynonymous
SFMBT2	chr10	7262435	7262435	C	T	nonsynonymous	nonsynonymous	nonsynonymous	nonsynonymous
SGK1	chr6	134583102	134583102	G	C	stopgain	stopgain	stopgain	stopgain
SIPA1L1	chr14	72090951	72090951	G	A	nonsynonymous	nonsynonymous	nonsynonymous	nonsynonymous
SIPA1L2	chr1	232649613	232649613	G	C	nonsynonymous	nonsynonymous	nonsynonymous	nonsynonymous
SLC18A2	chr10	119036769	119036769	A	C	nonsynonymous	nonsynonymous	nonsynonymous	nonsynonymous
SLC30A10	chr1	220100407	220100407	C	G	nonsynonymous	nonsynonymous	NA	nonsynonymous
SLC38A4	chr12	47172337	47172337	C	T	nonsynonymous	nonsynonymous	nonsynonymous	nonsynonymous
SLC39A10	chr2	196545546	196545546	G	T	nonsynonymous	nonsynonymous	nonsynonymous	nonsynonymous
SLC39A6	chr18	33702021	33702021	C	G	nonsynonymous	nonsynonymous	nonsynonymous	nonsynonymous
SLC9A8	chr20	48466195	48466195	G	C	nonsynonymous	nonsynonymous	nonsynonymous	nonsynonymous
SMC5	chr9	72892379	72892379	T	G	nonsynonymous	nonsynonymous	nonsynonymous	nonsynonymous
SNTG1	chr8	51415341	51415341	C	G	nonsynonymous	nonsynonymous	nonsynonymous	nonsynonymous
SP4	chr7	21550647	21550647	G	C	nonsynonymous	nonsynonymous	nonsynonymous	nonsynonymous
SPATS1	chr6	44320490	44320490	C	G	nonsynonymous	nonsynonymous	NA	nonsynonymous
SPEN	chr1	16257786	16257786	A	C	nonsynonymous	nonsynonymous	NA	nonsynonymous
SRPRB	chr3	133524825	133524825	C	T	nonsynonymous	nonsynonymous	nonsynonymous	nonsynonymous
STAC	chr3	36524564	36524564	C	T	stopgain	stopgain	stopgain	stopgain
STAU2	chr8	74334886	74334886	C	G	nonsynonymous	nonsynonymous	nonsynonymous	nonsynonymous
STIP1	chr11	63960601	63960601	G	A	nonsynonymous	nonsynonymous	nonsynonymous	nonsynonymous
STRA8	chr7	134925352	134925352	G	C	nonsynonymous	nonsynonymous	NA	nonsynonymous
SUMO4	chr6	149721789	149721789	C	T	stopgain	stopgain	stopgain	stopgain
SUN1	chr7	899853	899853	C	T	nonsynonymous	nonsynonymous	NA	nonsynonymous
SYT14	chr1	210334172	210334172	G	A	nonsynonymous	nonsynonymous	nonsynonymous	nonsynonymous
TBX18	chr6	85473796	85473796	T	C	nonsynonymous	nonsynonymous	NA	nonsynonymous
TCERG1	chr5	145859463	145859463	C	G	nonsynonymous	nonsynonymous	nonsynonymous	nonsynonymous
TCERG1L	chr10	133058651	133058651	C	T	nonsynonymous	nonsynonymous	nonsynonymous	nonsynonymous
TENM4	chr11	78399182	78399182	A	G	nonsynonymous	nonsynonymous	nonsynonymous	nonsynonymous
THSD7A	chr7	11486896	11486896	C	G	nonsynonymous	nonsynonymous	nonsynonymous	nonsynonymous
TM9SF1	chr14	24661529	24661529	T	C	nonsynonymous	nonsynonymous	nonsynonymous	nonsynonymous
TMCO3	chr13	114154408	114154408	A	G	nonsynonymous	nonsynonymous	nonsynonymous	nonsynonymous
TNKS1BP1	chr11	57077673	57077673	C	A	stopgain	stopgain	stopgain	stopgain
TNRC18	chr7	5352313	5352313	G	C	nonsynonymous	nonsynonymous	NA	nonsynonymous
TP53	chr17	7578212	7578212	G	A	stopgain	stopgain	stopgain	stopgain
TPP2	chr13	103299647	103299647	G	C	nonsynonymous	nonsynonymous	nonsynonymous	nonsynonymous
TRIM44	chr11	35747709	35747709	G	C	nonsynonymous	nonsynonymous	NA	nonsynonymous
TSPYL2	chrX	53114419	53114419	A	T	nonsynonymous	nonsynonymous	nonsynonymous	nonsynonymous
TTN	chr2	179474644	179474644	T	G	nonsynonymous	nonsynonymous	NA	nonsynonymous
USH2A	chr1	216062325	216062325	G	A	nonsynonymous	nonsynonymous	nonsynonymous	nonsynonymous
VN1R1	chr19	57966804	57966804	C	A	nonsynonymous	nonsynonymous	nonsynonymous	nonsynonymous
VN1R4	chr19	53770518	53770518	C	T	nonsynonymous	nonsynonymous	NA	nonsynonymous
VPS16	chr20	2843320	2843320	T	G	nonsynonymous	nonsynonymous	NA	nonsynonymous
WDR5B	chr3	122134323	122134323	G	T	stopgain	stopgain	stopgain	stopgain
WDR62	chr19	36593872	36593872	C	T	nonsynonymous	nonsynonymous	nonsynonymous	nonsynonymous
XPNPEP2	chrX	128886172	128886172	C	G	nonsynonymous	nonsynonymous	nonsynonymous	nonsynonymous
XRN2	chr20	21284237	21284237	C	T	nonsynonymous	nonsynonymous	NA	nonsynonymous
ZBTB18	chr1	244217282	244217282	G	C	nonsynonymous	nonsynonymous	nonsynonymous	nonsynonymous
ZBTB24	chr6	109797399	109797399	T	A	nonsynonymous	nonsynonymous	nonsynonymous	nonsynonymous
ZNF257	chr19	22271918	22271918	G	C	nonsynonymous	nonsynonymous	nonsynonymous	nonsynonymous
ZNF280C	chrX	129377622	129377622	G	C	stopgain	stopgain	stopgain	stopgain
ZNF316	chr7	6682182	6682182	G	C	nonsynonymous	nonsynonymous	NA	nonsynonymous
ZNF514	chr2	95818509	95818509	G	C	nonsynonymous	nonsynonymous	nonsynonymous	nonsynonymous
ZNF536	chr19	30935385	30935385	G	A	nonsynonymous	nonsynonymous	nonsynonymous	nonsynonymous
ZNF577	chr19	52381653	52381653	A	G	nonsynonymous	nonsynonymous	NA	nonsynonymous
ZNF768	chr16	30536901	30536901	T	A	nonsynonymous	nonsynonymous	nonsynonymous	nonsynonymous
ZSCAN32	chr16	3434609	3434609	C	G	nonsynonymous	nonsynonymous	nonsynonymous	nonsynonymous
ABCC10	chr6	43416925	43416925	C	G	NA	nonsynonymous	NA	NA
ALG6	chr1	63867927	63867927	A	G	NA	nonsynonymous	nonsynonymous	nonsynonymous
CACNA2D1	chr7	81746383	81746383	A	T	NA	nonsynonymous	NA	nonsynonymous
CATSPERE	chr1	244773557	244773557	G	A	NA	nonsynonymous	nonsynonymous	nonsynonymous
CD5L	chr1	157803081	157803081	G	A	NA	nonsynonymous	NA	NA
CDCA7	chr2	174223548	174223548	G	C	NA	nonsynonymous	nonsynonymous	nonsynonymous
CFAP221	chr2	120362837	120362837	C	T	NA	nonsynonymous	nonsynonymous	nonsynonymous
CHADL	chr22	41634205	41634205	C	G	NA	nonsynonymous	NA	nonsynonymous
DNAH5	chr5	13824412	13824412	G	C	NA	nonsynonymous	nonsynonymous	nonsynonymous
EML2	chr19	46148591	46148591	C	T	NA	nonsynonymous	nonsynonymous	nonsynonymous
EML3	chr11	62370680	62370680	T	C	NA	nonsynonymous	NA	nonsynonymous
FBXO17	chr19	39440752	39440752	C	T	NA	nonsynonymous	NA	NA
FNBP4	chr11	47744569	47744569	T	C	NA	nonsynonymous	nonsynonymous	nonsynonymous
FSIP2	chr2	186665713	186665713	G	A	NA	nonsynonymous	NA	nonsynonymous
IL12RB1	chr19	18180450	18180450	C	T	NA	nonsynonymous	nonsynonymous	nonsynonymous
ILDR1	chr3	121712403	121712403	G	A	NA	nonsynonymous	NA	nonsynonymous
KAT6B	chr10	76788261	76788261	G	C	NA	nonsynonymous	nonsynonymous	nonsynonymous
KLF15	chr3	126062712	126062712	C	T	NA	nonsynonymous	NA	NA
LAMA1	chr18	7049186	7049186	G	A	NA	nonsynonymous	NA	NA
LUZP1	chr1	23417960	23417960	G	C	NA	nonsynonymous	NA	NA
MFN1	chr3	179103475	179103475	G	C	NA	nonsynonymous	nonsynonymous	nonsynonymous
MYLK	chr3	123451866	123451866	C	T	NA	nonsynonymous	nonsynonymous	nonsynonymous
NECTIN4	chr1	161043062	161043062	C	T	NA	nonsynonymous	NA	nonsynonymous

OR8B8	chr11	124310497	124310497	C	T	NA	nonsynonymous	NA	NA
PEG10	chr7	94293422	94293422	C	G	NA	nonsynonymous	NA	nonsynonymous
PRAG1	chr8	8234013	8234013	G	A	NA	nonsynonymous	NA	NA
PTPN13	chr4	87622879	87622879	A	G	NA	nonsynonymous	NA	NA
RABEP2	chr16	28922244	28922244	C	T	NA	nonsynonymous	NA	nonsynonymous
RAD54L2	chr3	51661736	51661736	G	A	NA	nonsynonymous	NA	nonsynonymous
RECQL4	chr8	145741379	145741379	C	A	NA	nonsynonymous	NA	NA
RHBDF2	chr17	74473301	74473301	G	A	NA	nonsynonymous	NA	nonsynonymous
ROBO1	chr3	78766428	78766428	G	C	NA	nonsynonymous	NA	NA
RPL10L	chr14	47120842	47120842	A	T	NA	nonsynonymous	nonsynonymous	nonsynonymous
RSF1	chr11	77475726	77475726	C	A	NA	stopgain	stopgain	stopgain
SCN7A	chr2	167334062	167334062	T	G	NA	nonsynonymous	NA	nonsynonymous
SDE2	chr1	226173021	226173021	C	G	NA	nonsynonymous	NA	NA
SPAG11B	chr8	7308347	7308347	G	A	NA	nonsynonymous	nonsynonymous	nonsynonymous
SPEF1	chr20	3760321	3760321	G	A	NA	nonsynonymous	NA	nonsynonymous
TMEM68	chr8	56668863	56668863	A	T	NA	nonsynonymous	nonsynonymous	nonsynonymous
TMPRSS3	chr21	43795968	43795968	C	T	NA	nonsynonymous	NA	NA
ZNF343	chr20	2474438	2474438	T	C	NA	nonsynonymous	nonsynonymous	nonsynonymous
ZSCAN22	chr19	58850261	58850261	G	A	NA	nonsynonymous	nonsynonymous	nonsynonymous
CROCC2	chr2	241903904	241903904	G	C	NA	NA	nonsynonymous	nonsynonymous
GRIN2C	chr17	72851086	72851086	C	T	NA	NA	NA	nonsynonymous
OSBPL7	chr17	45895876	45895876	C	T	NA	NA	NA	nonsynonymous
RCN1	chr11	32125953	32126017	ATGTTTGTCGGAAGCCAAGCTACCAATTACGGGGAAGATCTCACAAAAAATCATGATGAGCTTTG	-	NA	NA	NA	frameshift_deletion
