# Supplementary material for: Clonal relationship and alcohol consumption-associated mutational signature in synchronous hypopharyngeal tumours and oesophageal squamous cell carcinoma
Source: Br J Cancer. 2022 Oct 19;127(12):2166–74. doi: 10.1038/s41416-022-01995-0 (PMC9726980; doi:10.1038/s41416-022-01995-0)
Supplement: Supplementary file 3 — Supplementory Table 6 [file 41416_2022_1995_MOESM3_ESM.pdf]

Shanghai cohort

	Clonality				Errors			
Clone	SH1-H	SH1-N1	SH1-E	SH1-N2	SH1-H	SH1-N1	SH1-E	SH1-N2
1	0.996	0.001	0.001	0.001	0.015	0.008	0.005	0.006
2	0.000	0.001	0.926	0.000	0.003	0.006	0.018	0.004
3	0.833	0.001	0.000	0.001	0.009	0.004	0.002	0.003
4	0.599	0.000	0.001	0.002	0.042	0.011	0.008	0.008
5	0.392	0.001	0.000	0.000	0.010	0.004	0.002	0.003
6	0.001	0.001	0.733	0.000	0.002	0.003	0.006	0.002
7	0.000	0.001	0.558	0.000	0.004	0.007	0.021	0.005
8	0.000	0.000	0.360	0.000	0.003	0.005	0.012	0.004
9	0.004	0.000	0.077	0.000	0.014	0.018	0.033	0.013
	Clonality				Errors			
Clone	SH2-H	SH2-N1	SH2-E	SH2-N2	SH2-H	SH2-N1	SH2-E	SH2-N2
1	0.075	0.003	0.950	0.003	0.005	0.004	0.004	0.003
2	0.357	0.003	0.000	0.000	0.078	0.019	0.017	0.015
3	0.007	0.000	0.801	0.000	0.011	0.012	0.021	0.009
4	0.033	0.000	0.467	0.000	0.014	0.010	0.034	0.007
	Clonality				Errors			
Clone	SH3-H	SH3-N1	SH3-E	SH3-N2	SH3-H	SH3-N1	SH3-E	SH3-N2
1	0.000	0.000	0.917	0.000	0.003	0.005	0.016	0.006
2	0.869	0.001	0.001	0.002	0.009	0.004	0.003	0.005
3	0.001	0.001	0.733	0.001	0.002	0.003	0.006	0.003
4	0.000	0.000	0.470	0.001	0.002	0.003	0.011	0.004
5	0.000	0.000	0.252	0.000	0.003	0.004	0.012	0.005
6	0.638	0.004	0.000	0.005	0.038	0.014	0.008	0.015
7	0.394	0.003	0.002	0.002	0.012	0.006	0.004	0.007

Hong Kong cohort

	Clonality						Errors					
Clone	HK1-EA	HK1-EB	HK1-EC	HK1-HA	HK1-HC	HK1-blood	HK1-EA	HK1-EB	HK1-EC	HK1-HA	HK1-HC	HK1-blood
1	0.936	0.884	0.946	0.000	0.000	0.000	0.057	0.078	0.042	0.012	0.034	0.024
2	0.000	0.000	0.000	0.937	0.558	0.000	0.005	0.005	0.005	0.013	0.022	0.006
3	0.730	0.640	0.931	0.000	0.000	0.000	0.044	0.032	0.029	0.018	0.018	0.017
4	0.632	0.611	0.498	0.001	0.000	0.000	0.029	0.044	0.029	0.008	0.015	0.010
5	0.525	0.353	0.481	0.001	0.001	0.001	0.011	0.013	0.011	0.003	0.006	0.005
6	0.094	0.345	0.253	0.000	0.000	0.000	0.051	0.076	0.071	0.018	0.028	0.023
7	0.374	0.002	0.006	0.000	0.000	0.000	0.051	0.016	0.016	0.010	0.022	0.015
8	0.000	0.000	0.369	0.005	0.000	0.000	0.025	0.027	0.093	0.023	0.036	0.033
9	0.000	0.000	0.001	0.429	0.408	0.000	0.006	0.006	0.006	0.023	0.028	0.007
10	0.002	0.000	0.001	0.541	0.224	0.008	0.010	0.012	0.017	0.050	0.022	0.016
11	0.018	0.003	0.002	0.000	0.454	0.006	0.013	0.012	0.014	0.065	0.008	0.012
12	0.007	0.000	0.000	0.019	0.360	0.000	0.032	0.032	0.012	0.101	0.012	0.006

	Clonality					Errors				
Clone	HK2-blood	HK2-EA	HK2-HA	HK2-HB	HK2-HC	HK2-blood	HK2-EA	HK2-HA	HK2-HB	HK2-HC
1	0.000	1.000	1.000	1.000	1.000	0.193	0.362	0.181	0.126	0.122
2	0.000	0.000	0.999	0.952	0.993	0.015	0.011	0.005	0.023	0.012
3	0.000	0.924	0.003	0.008	0.005	0.013	0.032	0.016	0.020	0.016
4	0.000	0.000	0.657	0.561	0.727	0.009	0.006	0.046	0.032	0.035
5	0.000	0.001	0.405	0.514	0.432	0.005	0.004	0.021	0.022	0.017
6	0.005	0.009	0.400	0.141	0.402	0.012	0.009	0.010	0.039	0.013
7	0.000	0.000	0.000	0.049	0.430	0.020	0.016	0.029	0.056	0.092
8	0.000	0.000	0.331	0.010	0.008	0.018	0.015	0.029	0.036	0.028
9	0.000	0.000	0.000	0.250	0.000	0.067	0.092	0.062	0.051	0.048
10	0.000	0.694	0.001	0.000	0.001	0.004	0.010	0.005	0.006	0.005
11	0.000	0.421	0.000	0.006	0.003	0.024	0.054	0.028	0.039	0.028

	Clonality			Errors		
Clone	HK3-blood	HK3-EB	HK3-HA	HK3-blood	HK3-EB	HK3-HA
1	0.000	1.000	1.000	0.209	0.116	0.090
2	0.000	0.001	0.996	0.010	0.007	0.017
3	0.001	0.957	0.000	0.005	0.006	0.005
4	0.001	0.000	0.708	0.003	0.002	0.006
5	0.001	0.000	0.538	0.004	0.003	0.015

6	0.000	0.712	0.000	0.007	0.024	0.008
7	0.001	0.438	0.001	0.005	0.006	0.006
8	0.000	0.238	0.046	0.010	0.052	0.038

	Clonality			Errors		
Clone	HK4-blood	HK4-E	HK4-H	HK4-blood	HK4-E	HK4-H
1	0.000	0.001	0.997	0.005	0.009	0.004
2	0.000	0.980	0.001	0.007	0.009	0.009
3	0.001	0.001	0.739	0.006	0.007	0.010
4	0.001	0.000	0.489	0.008	0.013	0.026
5	0.001	0.007	0.187	0.004	0.036	0.006
6	0.003	0.743	0.000	0.007	0.010	0.008
7	0.000	0.524	0.008	0.010	0.013	0.015
8	0.000	0.343	0.001	0.006	0.015	0.018

	Clonality					Errors				
Clone	HK5-blood	HK5-EA	HK5-EB	HK5-EC	HK5-ED	HK5-blood	HK5-EA	HK5-EB	HK5-EC	HK5-ED
1	0.000	0.810	0.003	0.367	0.808	0.008	0.018	0.006	0.022	0.029
2	0.000	0.001	0.947	0.002	0.000	0.029	0.015	0.042	0.014	0.018
3	0.000	0.412	0.002	0.340	0.575	0.012	0.034	0.008	0.032	0.053
4	0.000	0.440	0.000	0.004	0.358	0.014	0.050	0.009	0.007	0.055
5	0.006	0.116	0.014	0.240	0.332	0.015	0.030	0.011	0.035	0.020
6	0.001	0.444	0.005	0.002	0.004	0.011	0.025	0.007	0.005	0.010
7	0.003	0.040	0.001	0.000	0.355	0.008	0.014	0.007	0.006	0.011
8	0.000	0.217	0.000	0.001	0.005	0.010	0.027	0.007	0.004	0.012
9	0.005	0.011	0.000	0.000	0.243	0.014	0.023	0.009	0.011	0.025
10	0.000	0.001	0.001	0.256	0.006	0.010	0.007	0.008	0.014	0.012
11	0.000	0.000	0.525	0.000	0.001	0.003	0.002	0.009	0.001	0.003

	Clonality				Errors			
Clone	HK6-blood	HK6-EB	HK6-ED	HK6-EE	HK6-blood	HK6-EB	HK6-ED	HK6-EE
1	0.000	0.785	0.976	0.941	0.012	0.031	0.020	0.017
2	0.000	0.798	0.508	0.684	0.044	0.088	0.112	0.086
3	0.002	0.406	0.601	0.822	0.027	0.024	0.034	0.024
4	0.001	0.300	0.452	0.521	0.006	0.011	0.011	0.010
5	0.000	0.266	0.399	0.013	0.006	0.022	0.023	0.010
6	0.000	0.004	0.006	0.507	0.009	0.010	0.011	0.029
7	0.000	0.000	0.418	0.000	0.029	0.031	0.092	0.035

	Clonality				Errors			
Clone	HK7-blood	HK7-EB	HK7-EC	HK7-ED	HK7-blood	HK7-EB	HK7-EC	HK7-ED
1	0.000	0.976	1.000	0.892	0.015	0.021	0.004	0.045
2	0.007	0.559	0.952	0.605	0.024	0.034	0.025	0.044
3	0.000	0.425	0.662	0.679	0.020	0.026	0.034	0.059
4	0.001	0.442	0.688	0.478	0.003	0.008	0.009	0.007
5	0.009	0.438	0.188	0.498	0.048	0.057	0.040	0.027
6	0.000	0.002	0.503	0.000	0.012	0.016	0.063	0.017
7	0.001	0.401	0.269	0.001	0.009	0.034	0.036	0.014
8	0.000	0.002	0.004	0.409	0.009	0.012	0.014	0.036

	Clonality						Errors					
Clone	HK8-blood	HK8-EA	HK8-EB	HK8-EC	HK8-ED	HK8-EE	HK8-blood	HK8-EA	HK8-EB	HK8-EC	HK8-ED	HK8-EE
1	0.001	0.769	0.786	0.839	0.862	0.840	0.006	0.007	0.009	0.007	0.006	0.005
2	0.000	0.460	0.550	0.663	0.703	0.712	0.020	0.045	0.034	0.048	0.049	0.035
3	0.000	0.395	0.406	0.425	0.435	0.435	0.006	0.013	0.014	0.010	0.012	0.013
4	0.000	0.000	0.000	0.421	0.439	0.418	0.017	0.008	0.008	0.017	0.032	0.027
5	0.003	0.411	0.401	0.006	0.000	0.001	0.017	0.018	0.025	0.008	0.009	0.010
6	0.000	0.000	0.000	0.302	0.214	0.062	0.024	0.012	0.011	0.057	0.051	0.037
7	0.000	0.012	0.153	0.000	0.000	0.000	0.017	0.018	0.047	0.009	0.014	0.009

	Clonality				Errors			
Clone	HK9-blood	HK9-EA	HK9-EB	HK9-EC	HK9-blood	HK9-EA	HK9-EB	HK9-EC
1	0.001	0.463	0.858	0.885	0.006	0.030	0.016	0.017
2	0.001	0.270	0.713	0.761	0.002	0.006	0.006	0.006
3	0.000	0.162	0.672	0.788	0.005	0.011	0.016	0.014
4	0.000	0.007	0.766	0.715	0.016	0.022	0.077	0.078

5	0.000	0.001	0.583	0.000	0.011	0.011	0.041	0.012
6	0.000	0.009	0.018	0.400	0.032	0.038	0.029	0.065

	Clonality				Errors			
Clone	HK10-blood	HK10-EA	HK10-EB	HK10-EC	HK10-blood	HK10-EA	HK10-EB	HK10-EC
1	0.000	0.999	0.992	0.990	0.021	0.043	0.022	0.028
2	0.000	0.716	0.953	0.741	0.009	0.032	0.025	0.041
3	0.001	0.618	0.788	0.441	0.008	0.021	0.015	0.020
4	0.002	0.299	0.754	0.355	0.013	0.026	0.014	0.025
5	0.005	0.001	0.749	0.337	0.012	0.008	0.013	0.021
6	0.001	0.264	0.002	0.001	0.009	0.016	0.009	0.009
7	0.001	0.001	0.398	0.174	0.004	0.002	0.009	0.007
8	0.000	0.000	0.358	0.000	0.011	0.008	0.056	0.014

	Clonality					Errors				
Clone	HK11-blood	HK11-EA	HK11-EB	HK11-EC	HK11-ED	HK11-blood	HK11-EA	HK11-EB	HK11-EC	HK11-ED
1	9.2E-05	0.988	0.747	0.797	0.833	0.017	0.026	0.039	0.043	0.039
2	0.005	0.859	0.496	0.700	0.566	0.025	0.043	0.033	0.029	0.044
3	0.001	0.507	0.502	0.454	0.445	0.009	0.026	0.030	0.025	0.026
4	0.002	0.510	0.313	0.409	0.337	0.005	0.009	0.008	0.009	0.008
5	0.024	0.249	0.285	0.088	0.350	0.028	0.053	0.018	0.067	0.037
6	0.000	0.004	0.041	0.385	0.006	0.023	0.022	0.040	0.081	0.020
7	0.000	0.371	0.000	0.010	0.000	0.019	0.073	0.016	0.021	0.015

	Clonality					Errors				
Clone	HK12-blood	HK12-EB	HK12-EC	HK12-ED	HK12-EA	HK12-blood	HK12-EB	HK12-EC	HK12-ED	HK12-EA
1	0	0.613	0.983	0.994	0.991	0.017	0.046	0.055	0.048	0.019
2	5.9228E-05	0.286	0.970	0.652	0.975	0.014	0.038	0.034	0.045	0.044
3	0.006	0.181	0.367	0.289	0.944	0.033	0.040	0.078	0.064	0.076
4	0.035	0.319	0.550	0.654	0.000	0.079	0.066	0.105	0.102	0.188
5	0.000	0.214	0.252	0.298	0.528	0.014	0.038	0.043	0.042	0.049
6	0.000	0.028	0.000	0.365	0.030	0.025	0.020	0.015	0.038	0.031
7	0.000	0.314	0.006	0.000	0.004	0.019	0.067	0.020	0.014	0.016
8	0.000	0.000	0.001	0.002	0.313	0.019	0.010	0.013	0.014	0.043
9	0.000	0.000	0.887	0.000	0.000	0.029	0.011	0.137	0.020	0.019
10	6.2161E-05	0.001	0.537	0.000	0.000	0.008	0.005	0.020	0.006	0.005

	Clonality					Errors				
Clone	HK13-blood	HK13-EA	HK13-EB	HK13-EC	HK13-ED	HK13-blood	HK13-EA	HK13-EB	HK13-EC	HK13-ED
1	0.002	0.978	0.711	0.999	0.816	0.012	0.019	0.042	0.008	0.038
2	0.000	0.864	0.703	0.786	0.677	0.008	0.013	0.018	0.011	0.009
3	0.001	0.853	0.002	0.793	0.242	0.007	0.018	0.005	0.024	0.020
4	0.000	0.000	0.611	0.000	0.365	0.005	0.003	0.021	0.007	0.018
5	0.000	0.410	0.000	0.795	0.111	0.009	0.028	0.007	0.032	0.019
6	0.003	0.002	0.001	0.431	0.033	0.012	0.006	0.006	0.023	0.009
7	0.000	0.389	0.001	0.002	0.000	0.005	0.016	0.004	0.007	0.003
8	0.000	0.000	0.167	0.000	0.260	0.009	0.006	0.025	0.011	0.029

	Clonality					Errors				
Clone	HK14-blood	HK14-EA	HK14-EB	HK14-EC	HK14-ED	HK14-blood	HK14-EA	HK14-EB	HK14-EC	HK14-ED
1	0.000	0.774	0.995	0.962	0.931	0.032	0.077	0.035	0.070	0.076
2	0.000	1.000	0.636	0.546	0.929	0.025	0.076	0.183	0.063	0.124
3	0.002	0.574	0.725	0.491	0.562	0.007	0.008	0.010	0.010	0.010
4	0.001	0.349	0.675	0.263	0.293	0.013	0.013	0.040	0.018	0.026
5	0.000	0.173	0.629	0.062	0.194	0.018	0.048	0.050	0.019	0.043
6	0.000	0.000	0.411	0.005	0.000	0.017	0.009	0.047	0.009	0.007

	Clonality					Errors				
Clone	HK15-blood	HK15-EA	HK15-EB	HK15-EC	HK15-ED	HK15-blood	HK15-EA	HK15-EB	HK15-EC	HK15-ED
1	0.000	0.995	1.000	0.996	1.000	0.031	0.011	0.004	0.012	0.003
2	0.001	0.353	0.770	0.264	0.753	0.006	0.017	0.013	0.014	0.014
3	0.000	0.313	0.634	0.249	0.698	0.002	0.005	0.005	0.005	0.005
4	0.000	0.330	0.665	0.018	0.746	0.011	0.029	0.033	0.008	0.032
5	0.001	0.001	0.000	0.230	0.000	0.025	0.018	0.017	0.021	0.018
6	0.000	0.005	0.360	0.002	0.000	0.015	0.014	0.050	0.009	0.012
