# Supplementary material for: Clonal relationship and alcohol consumption-associated mutational signature in synchronous hypopharyngeal tumours and oesophageal squamous cell carcinoma
Source: Br J Cancer. 2022 Oct 19;127(12):2166–74. doi: 10.1038/s41416-022-01995-0 (PMC9726980; doi:10.1038/s41416-022-01995-0)
Supplement: Supplementary file 4 — Supplementory Table 7 [file 41416_2022_1995_MOESM4_ESM.pdf]

chr	start	end	name	clone	clonality SH2-HT	clonality SH2-HN	clonality SH2-ET	clonality SH2-EN	error SH2-HT	error SH2-HN	error SH2-ET	error SH2-EN	severity	type	AA pos	AA before	AA after	isCosmic Census
1	16268467	16268467	ZBTB17 (1) frameshift	1	0.25	0.00	0.92	0.00	0.30	0.07	0.23	0.06	5	frameshift	803			FALSE
1	26629661	26629661	UBXN11 (1) intron	1	0.07	0.00	0.83	0.00	0.12	0.05	0.30	0.04	22	intron				FALSE
1	26801691	26801691	HMGN2 (1) threeUTR	1	0.07	0.00	0.84	0.00	0.12	0.10	0.36	0.07	20	threeUTR				FALSE
1	28422925	28422925	SPCS2P4 (1) intergenic	1	0.10	0.00	1.00	0.00	0.12	0.05	0.37	0.04	30	intergenic				FALSE
1	29030551	29030551	GMEB1 (1) intron	1	0.00	0.00	1.00	0.00	0.12	0.12	0.10	0.11	22	intron				FALSE
1	31973167	31973167	NST00000418618 (1) nonsynonymo	1	0.19	0.00	1.00	0.00	0.22	0.10	0.28	0.09	10	nonsynonymous	75	A	T	FALSE
1	43830263	43830263	ELOVL1 (1) nonsynonymous	1	0.10	0.00	0.95	0.00	0.13	0.03	0.13	0.03	10	nonsynonymous	144	H	R	FALSE
1	72163884	72163884	NEGR1 (1) intron	1	0.09	0.00	0.97	0.00	0.11	0.05	0.06	0.03	22	intron				FALSE
1	98348998	98348998	DPYD (1) intron	1	0.15	0.00	0.98	0.00	0.16	0.12	0.07	0.06	22	intron				FALSE
1	102268675	102268675	OLFM3 (1) threeUTR	1	0.10	0.00	0.96	0.00	0.06	0.02	0.04	0.01	20	threeUTR				FALSE
1	115590807	115590807	TSPAN2 (1) threeUTR	1	0.14	0.00	0.88	0.00	0.15	0.10	0.13	0.06	20	threeUTR				FALSE
1	149949669	149949669	OTUD7B (1) intron	1	0.22	0.00	0.92	0.00	0.36	0.21	0.30	0.13	22	intron				FALSE
1	151259132	151259132	ZNF687 (1) nonsynonymous	1	0.20	0.00	0.89	0.00	0.23	0.06	0.13	0.05	10	nonsynonymous	131	R	Q	FALSE
1	162473486	162473486	UHMK1 (1) intron	1	0.15	0.00	1.00	0.00	0.13	0.03	0.26	0.03	22	intron				FALSE
1	162473487	162473487	UHMK1 (1) intron	1	0.15	0.00	1.00	0.00	0.13	0.04	0.05	0.03	22	intron				FALSE
1	169390720	169390720	CCDC181 (1) nonsynonymous	1	0.09	0.04	1.00	0.05	0.11	0.08	0.23	0.06	10	nonsynonymous	317	S	A	FALSE
1	172017687	172017687	DNM3 (1) intron	1	0.16	0.00	0.99	0.00	0.16	0.04	0.25	0.04	22	intron				FALSE
1	174980521	174980521	MRPS14 (1) threeUTR	1	0.16	0.00	1.00	0.00	0.12	0.03	0.21	0.02	20	threeUTR				FALSE
1	179312640	179312640	SOAT1 (1) intron	1	0.17	0.07	1.00	0.00	0.27	0.12	0.06	0.06	22	intron				FALSE
1	186342422	186342422	TPR (1) intron	1	0.43	0.00	1.00	0.04	0.27	0.09	0.06	0.08	22	intron				TRUE
1	202154046	202154046	PTPRVP (1) promoter	1	0.00	0.00	0.91	0.00	1.00	0.16	0.49	0.12	19	promoter				FALSE
1	220191982	220191982	EPRS (1) intron	1	0.12	0.00	0.96	0.00	0.15	0.10	0.35	0.06	22	intron				FALSE
1	226549106	226549106	PARP1 (1) threeUTR	1	0.26	0.00	1.00	0.00	0.13	0.02	0.03	0.02	20	threeUTR				FALSE
1	242012254	242012254	EXO1 (1) fiveUTR	1	0.05	0.03	0.95	0.00	0.09	0.05	0.19	0.02	21	fiveUTR				FALSE
2	11780715	11780715	GREB1 (2) threeUTR	1	0.05	0.00	0.98	0.00	0.09	0.04	0.22	0.03	20	threeUTR				FALSE
2	12882027	12882027	TRIB2 (2) threeUTR	1	0.19	0.02	1.00	0.00	0.11	0.04	0.17	0.02	20	threeUTR				FALSE
2	17912501	17912501	SMC6 (2) intron	1	0.26	0.00	0.99	0.00	0.15	0.05	0.25	0.04	22	intron				FALSE
2	24413974	24413974	FAM228A (2) threeUTR	1	0.18	0.00	1.00	0.00	0.16	0.06	0.05	0.04	20	threeUTR				FALSE
2	27361745	27361745	C2orf53 (2) promoter	1	0.00	0.00	1.00	0.00	0.19	0.09	0.44	0.07	19	promoter				FALSE
2	40657352	40657352	SLC8A1 (2) synonymous	1	0.22	0.00	1.00	0.00	0.11	0.03	0.04	0.02	16	synonymous	23	V	V	FALSE
2	46803840	46803840	RHOQ (2) intron	1	0.20	0.00	0.80	0.00	0.13	0.03	0.17	0.03	22	intron				FALSE
2	55874657	55874657	PNPT1 (2) intron	1	0.39	0.00	0.95	0.00	0.30	0.17	0.32	0.08	22	intron				FALSE
2	86769208	86769208	RNF103-CHMP3 (2) intron	1	0.19	0.00	1.00	0.00	0.19	0.06	0.37	0.06	22	intron				FALSE
2	87225035	87225035	RGPD1 (2) intron	1	0.20	0.00	0.82	0.00	0.30	0.11	0.43	0.09	22	intron				FALSE
2	89100709	89100709	ANKRD36BP2 (2) nonsynonymous	1	0.15	0.02	0.90	0.02	0.08	0.04	0.15	0.03	10	nonsynonymous	125	A	T	FALSE
2	89998816	89998816	IGKV2D-28 (2) intron	1	0.12	0.00	0.83	0.00	0.14	0.06	0.25	0.04	22	intron				FALSE
2	99206794	99206794	INPP4A (2) threeUTR	1	0.29	0.03	0.84	0.00	0.13	0.05	0.18	0.02	20	threeUTR				FALSE
2	131132773	131132773	PTPN18 (2) threeUTR	1	0.23	0.00	0.91	0.00	0.19	0.03	0.21	0.03	20	threeUTR				FALSE
2	170929815	170929815	UBR3 (2) intron	1	0.10	0.00	1.00	0.00	0.17	0.11	0.42	0.12	22	intron				FALSE
2	175304552	175304552	GPR155 (2) intron	1	0.19	0.04	1.00	0.00	0.16	0.08	0.31	0.04	22	intron				FALSE
2	179576607	179576607	TTN-AS1 (2) intron	1	0.04	0.00	1.00	0.03	0.08	0.05	0.27	0.06	22	intron				FALSE
2	197000824	197000824	STK17B (2) threeUTR	1	0.06	0.00	1.00	0.00	0.07	0.03	0.23	0.02	20	threeUTR				FALSE
2	201486249	201486249	AOX1 (2) intron	1	0.25	0.00	1.00	0.00	0.36	0.19	0.47	0.18	22	intron				FALSE
2	201721599	201721599	CLK1 (2) intron	1	0.05	0.00	1.00	0.00	0.09	0.04	0.30	0.03	22	intron				FALSE
2	203142373	203142373	NOP58 (2) promoter	1	0.36	0.00	1.00	0.00	0.46	0.21	1.32	0.23	19	promoter				FALSE
2	204259810	204259810	RAPH1 (2) intron	1	0.03	0.00	1.00	0.00	0.06	0.03	0.27	0.02	22	intron				FALSE
2	210847310	210847310	UNC80 (2) threeUTR	1	0.12	0.00	0.84	0.00	0.09	0.02	0.11	0.02	20	threeUTR				FALSE
2	226377873	226377873	NYAP2 (2) intron	1	0.00	0.00	1.00	0.00	0.18	0.19	0.45	0.16	22	intron				FALSE
2	233630446	233630446	KCNJ13 (2) intron	1	0.20	0.00	0.99	0.05	0.18	0.07	0.21	0.09	22	intron				FALSE
2	233712017	233712017	GIGYF2 (2) intron	1	0.06	0.00	0.98	0.00	0.10	0.05	0.15	0.04	22	intron				FALSE
2	237416286	237416286	IQCA1 (2) promoter	1	0.00	0.00	1.00	0.00	0.26	0.19	0.51	0.12	19	promoter				FALSE
2	242033239	242033239	SNED1 (2) threeUTR	1	0.10	0.00	0.85	0.00	0.11	0.03	0.15	0.02	20	threeUTR				FALSE
3	42915647	42915647	KRBOX1 (3) threeUTR	1	0.00	0.00	0.96	0.00	0.07	0.04	0.07	0.04	20	threeUTR				FALSE
3	48015541	48015541	MAP4 (3) threeUTR	1	0.11	0.08	0.97	0.03	0.12	0.10	0.04	0.05	20	threeUTR				FALSE
3	56916472	56916472	ARHGEF3 (3) intron	1	0.07	0.00	0.99	0.00	0.08	0.04	0.04	0.03	22	intron				FALSE
3	67450899	67450899	SUCLG2 (3) intron	1	0.27	0.00	1.00	0.00	0.43	0.16	0.17	0.19	22	intron				FALSE
3	69084029	69084029	TMF1 (3) intron	1	0.11	0.00	0.93	0.00	0.11	0.07	0.07	0.04	22	intron				FALSE
3	69105209	69105209	UBA3 (3) nonsynonymous	1	0.11	0.00	0.96	0.00	0.08	0.03	0.04	0.02	10	nonsynonymous	397	M	T	FALSE
3	98298448	98298448	CPOX (3) threeUTR	1	0.45	0.00	1.00	0.00	0.20	0.05	0.20	0.03	20	threeUTR				FALSE
3	100498926	100498926	ABI3BP (3) intron	1	0.29	0.00	0.93	0.00	0.22	0.09	0.07	0.04	22	intron				FALSE
3	108229353	108229353	MYH15 (3) nonsynonymous	1	0.16	0.00	0.72	0.00	0.16	0.09	0.34	0.05	10	nonsynonymous	29	A	T	FALSE
3	108475877	108475877	RETNLB (3) intron	1	0.17	0.07	1.00	0.00	0.19	0.12	0.65	0.04	22	intron				FALSE
3	109027239	109027239	DPPA2 (3) intron	1	0.00	0.00	0.97	0.00	0.15	0.15	0.06	0.08	22	intron				FALSE
3	119135539	119135539	ARHGAP31 (3) threeUTR	1	0.12	0.00	1.00	0.00	0.12	0.03	0.33	0.02	20	threeUTR				FALSE
3	123426520	123426520	MYLK (3) intron	1	0.36	0.00	0.83	0.00	0.33	0.09	0.53	0.05	22	intron				FALSE
3	129225368	129225368	IFT122 (3) frameshift	1	0.12	0.00	1.00	0.00	0.14	0.06	0.47	0.04	5	frameshift	763			FALSE
3	138456811	138456811	PIK3CB (3) intron	1	0.15	0.00	0.71	0.00	0.23	0.15	0.64	0.08	22	intron				FALSE
3	142984387	142984387	SLC9A9 (3) threeUTR	1	0.23	0.00	1.00	0.00	0.13	0.04	0.02	0.02	20	threeUTR				FALSE
3	155628563	155628563	GMPS (3) synonymous	1	0.22	0.00	0.92	0.00	0.15	0.05	0.34	0.03	16	synonymous	203	L	L	TRUE
3	160225913	160225913	KPNA4 (3) nonsynonymous	1	0.11	0.00	1.00	0.00	0.11	0.05	0.54	0.02	10	nonsynonymous	452	L	V	FALSE
3	168825559	168825559	MECOM (3) intron	1	0.00	0.00	0.93	0.00	0.10	0.10	0.07	0.07	22	intron				TRUE
3	178952085	178952085	PIK3CA (3) nonsynonymous	1	0.14	0.00	1.00	0.00	0.12	0.05	0.45	0.03	10	nonsynonymous	1047	H	R	TRUE
3	185798998	185798998	ETV5-AS1 (3) intron	1	0.15	0.00	0.96	0.00	0.16	0.09	0.05	0.05	22	intron				FALSE
3	196434704	196434704	PIGX (3) synonymous	1	0.23	0.00	1.00	0.00	0.12	0.03	0.37	0.02	16	synonymous	74	L	L	FALSE
4	3433077	3433077	RGS12 (4) threeUTR	1	0.06	0.00	0.96	0.00	0.11	0.05	0.05	0.04	20	threeUTR				FALSE
4	3768437	3768437	ADRA2C (4) nonsynonymous	1	0.31	0.00	0.95	0.00	0.48	0.19	0.15	0.16	10	nonsynonymous	35	S	L	FALSE
4	46976018	46976018	GABRA4 (4) intron	1	0.00	0.00	1.00	0.00	0.15	0.23	0.14	0.14	22	intron				FALSE
4	49643002	49643002	? (4) intergenic	1	0.00	0.00	1.00	0.00	1.00	0.46	0.60	0.25	30	intergenic				FALSE
4	68604184	68604184	UBA6-AS1 (4) threeUTR	1	0.08	0.00	0.97	0.02	0.07	0.04	0.04	0.04	20	threeUTR				FALSE
4	80246435	80246435	NAA11 (4) synonymous	1	0.15	0.00	0.94	0.00	0.11	0.03	0.05	0.04	16	synonymous	199	T	T	FALSE
4	90646765	90646765	SNCA (4) threeUTR	1	0.10	0.00	0.98	0.00	0.09	0.03	0.03	0.02	20	threeUTR				FALSE
4	101342515	101342515	EMCN (4) synonymous	1	0.10	0.00	1.00	0.00	0.08	0.03	0.12	0.02	16	synonymous	196	V	V	FALSE
4	106182991	106182991	TET2 (4) nonsynonymous	1	0.15	0.00	0.98	0.00	0.10	0.04	0.04	0.03	10	nonsynonymous	1344	A	T	TRUE
4	145579946	145579946	HHIP-AS1 (4) nonsynonymous	1	0.18	0.00	1.00	0.00	0.11	0.04	0.04	0.04	10	nonsynonymous	159	F	L	FALSE
4	146076780	146076780	OTUD4 (4) nonsynonymous	1	0.13	0.00	0.97	0.02	0.07	0.02	0.03	0.03	10	nonsynonymous	185	S	L	FALSE
4	153247226	153247226	FBXW7 (4) nonsynonymous	1	0.12	0.00	0.98	0.00	0.07	0.03	0.03	0.02	10	nonsynonymous	350	W	R	FALSE
4	153802598	153802598	ARFIP1 (4) intron	1	0.00	0.00	1.00	0.00	0.28	0.46	0.23	0.38	22	intron				FALSE
4	153882043	153882043	FHDC1 (4) intron	1	0.04	0.00	1.00	0.00	0.06	0.06	0.19	0.05	22	intron				FALSE
4	186083181	186083181	KIAA1430 (4) threeUTR	1	0.17	0.00	0.02	0.00	0.07	0.02	0.98	0.02	20	threeUTR				FALSE
5	34687918	34687918	RAI14 (5) intron	1	0.11	0.00	0.92	0.00	0.13	0.04	0.15	0.04	22	intron				FALSE
5	38918931	38918931	OSMR (5) intron	1	0.08	0.00	1.00	0.00	0.10	0.04	0.35	0.04	22	intron				FALSE
5	40715884	40715884	TTC33 (5) threeUTR	1	0.10	0.00	1.00	0.00	0.07	0.03	0.23	0.02	20	threeUTR				FALSE
5	45353372	45353372	HCN1 (5) intron	1	0.21	0.00	1.00	0.07	0.24	0.10	0.54	0.13	22	intron				FALSE
5	52095871	52095871	PELO (5) fiveUTR	1	0.15	0.00	0.91	0.00	0.24	0.08	0.08	0.06	21	fiveUTR				FALSE
5	56180785	56180785	MAP3K1 (5) intron	1	0.00	0.00	1.00	0.07	0.11	0.10	0.10	0.13	22	intron				FALSE
5	68667951	68667951	RAD17 (5) fiveUTR	1	0.19	0.00	0.95	0.02	0.13	0.03	0.05	0.04	21	fiveUTR				FALSE
5	79960935	79960935	MSH3 (5) intron	1	0.15	0.00	0.95	0.00	0.23	0.13	0.16	0.10	22	intron				FALSE
5	80109480	80109480	MSH3 (5) nonsynonymous	1	0.14	0.00	0.97	0.00	0.13	0.03	0.04	0.03	10	nonsynonymous	911	L	F	FALSE
5	95195867	95195867	C5orf27 (5) intergenic	1	0.15	0.00	1.00	0.00	0.13	0.04	0.14	0.02	30	intergenic				FALSE
5	96119660	96119660	ERAP1 (5) nonsynonymous	1	0.20	0.03	0.94	0.00	0.15	0.06	0.05	0.02	10	nonsynonymous	690	R	G	FALSE
5	106712653	106712653	EFNA5 (5) threeUTR	1	0.08	0.00	0.94	0.00	0.10	0.04	0.30	0.03	20	threeUTR				FALSE
5	112197045	112197045	SRP19 (5) promoter	1	0.29	0.00	1.00	0.00	0.24	0.04	0.34	0.04	19	promoter				FALSE
5	139725975	139725975	HBEGF (5) fiveUTR	1	0.32	0.00	0.93	0.00	0.34	0.15	0.59	0.12	21	fiveUTR				FALSE
5	140558171	140558171	PCDHB8 (5) nonsynonymous	1	0.05	0.00	0.97	0.00	0.08	0.03	0.20	0.02	10	nonsynonymous	186	R	C	FALSE
5	150923332	150923332	FAT2 (5) nonsense	1	0.06	0.00	1.00	0.00	0.10	0.04	0.31	0.03	4	nonsense	2450	YNL	*	FALSE
5	156915989	156915989	ADAM19 (5) intron	1	0.00	0.00	0.92	0.00	0.17	0.15	0.24	0.11	22	intron				FALSE
5	171764378	171764378	SH3PXD2B (5) threeUTR	1	0.05	0.00	0.98	0.00	0.09	0.04	0.26	0.03	20	threeUTR				FALSE
5	175992391	175992391	CDHR2 (5) nonsynonymous	1	0.12	0.00	0.88	0.00	0.19	0.05	0.12	0.05	10	nonsynonymous	13	A	V	FALSE
5	178418456	178418456	GRM6 (5) nonsynonymous	1	0.16	0.00	0.86	0.00	0.25	0.06	0.39	0.06	10	nonsynonymous	276	I	L	FALSE
5	179315305	179315305	TBC1D9B (5) nonsynonymous	1	0.14	0.00	1.00	0.00	0.22	0.07	0.38	0.06	10	nonsynonymous	351	I	T	FALSE
6	7393771	7393771	RIOK1 (6) intron	1	0.00	0.00	1.00	0.00	0.19	0.08	0.11	0.14	22	intron				FALSE
6	16299878	16299878	ATXN1 (6) threeUTR	1	0.19	0.00	1.00	0.03	0.13	0.03	0.05	0.05	20	threeUTR				FALSE

6	25492513	25492513	LRRC16A (6) intron	1	0.00	0.00	1.00	0.00	0.17	0.17	0.11	0.17	22	intron				FALSE
6	25868702	25868702	SLC17A3 (6) promoter	1	0.00	0.00	1.00	0.00	0.07	0.06	0.06	0.06	19	promoter				FALSE
6	27861105	27861105	HIST1H2BO (6) promoter	1	0.14	0.00	0.99	0.00	0.22	0.07	0.30	0.05	19	promoter				FALSE
6	46188950	46188950	RCAN2 (6) threeUTR	1	0.25	0.00	0.91	0.00	0.14	0.04	0.19	0.04	20	threeUTR				FALSE
6	56494301	56494301	DST (6) intron	1	0.06	0.00	1.00	0.00	0.10	0.08	0.08	0.07	22	intron				FALSE
6	66416964	66416964	EYS (6) promoter	1	0.00	0.00	1.00	0.00	0.11	0.25	0.13	0.09	19	promoter				FALSE
6	74534342	74534342	CD109 (6) threeUTR	1	0.09	0.00	1.00	0.00	0.10	0.03	0.04	0.04	20	threeUTR				FALSE
6	76426841	76426841	SENP6 (6) threeUTR	1	0.05	0.00	0.85	0.00	0.07	0.03	0.45	0.04	20	threeUTR				FALSE
6	84303186	84303186	SNAP91 (6) intron	1	0.14	0.00	1.00	0.00	0.09	0.03	0.05	0.03	22	intron				FALSE
6	109871385	109871385	AK9 (6) nonsense	1	0.09	0.00	0.81	0.00	0.09	0.02	1.09	0.02	4	nonsense	958	R	*	FALSE
6	127770884	127770884	SOGA3 (6) intron	1	0.00	0.00	0.99	0.00	0.09	0.05	0.11	0.07	22	intron				FALSE
6	128176056	128176056	THEMIS (6) intron	1	0.06	0.00	1.00	0.00	0.11	0.07	0.08	0.08	22	intron				FALSE
6	128411153	128411153	PTPRK (6) intron	1	0.08	0.00	0.90	0.00	0.10	0.04	0.46	0.04	22	intron				TRUE
6	131926682	131926682	MED23 (6) intron	1	0.15	0.00	0.75	0.00	0.17	0.07	0.43	0.07	22	intron				FALSE
6	132643998	132643998	MOXD1 (6) synonymous	1	0.09	0.00	0.84	0.00	0.11	0.06	0.45	0.05	16	synonymous	307	A	A	FALSE
7	1542490	1542490	INTS1 (7) intron	1	0.29	0.00	1.00	0.00	0.32	0.10	0.40	0.06	22	intron				FALSE
7	24881625	24881625	OSBPL3 (7) intron	1	0.00	0.00	1.00	0.00	0.23	0.21	0.11	0.18	22	intron				FALSE
7	30796287	30796287	INMT-FAM188B (7) threeUTR	1	0.10	0.00	1.00	0.00	0.17	0.08	0.41	0.05	20	threeUTR				FALSE
7	33102343	33102343	NT5C3A (7) promoter	1	0.00	0.00	1.00	0.00	0.30	0.16	0.74	0.14	19	promoter				FALSE
7	33102344	33102344	NT5C3A (7) promoter	1	0.00	0.00	1.00	0.00	0.33	0.16	0.09	0.15	19	promoter				FALSE
7	72671603	72671603	GTF2IRD2P1 (7) intron	1	0.10	0.00	1.00	0.00	0.17	0.07	0.32	0.06	22	intron				FALSE
7	83592732	83592732	SEMA3A (7) intron	1	0.18	0.00	1.00	0.03	0.15	0.10	0.31	0.06	22	intron				FALSE
7	96654390	96654390	DLX5 (7) promoter	1	0.36	0.00	0.91	0.00	0.34	0.11	0.32	0.08	19	promoter				FALSE
7	99951722	99951722	TAG3L5P-PVRIG2P-PILRB (7) intro	1	0.00	0.00	0.96	0.00	0.47	0.14	0.52	0.21	22	intron				FALSE
7	105108953	105108953	PUS7 (7) intron	1	0.32	0.00	1.00	0.00	0.21	0.05	0.28	0.04	22	intron				FALSE
7	123334757	123334757	WASL (7) intron	1	0.23	0.00	1.00	0.00	0.11	0.04	0.25	0.02	22	intron				FALSE
7	138437303	138437303	ATP6V0A4 (7) intron	1	0.00	0.00	0.94	0.00	0.33	0.21	0.56	0.11	22	intron				FALSE
7	138957242	138957242	UBN2 (7) intron	1	0.44	0.11	1.00	0.00	0.30	0.19	0.40	0.05	22	intron				FALSE
7	139786051	139786051	KDM7A (7) threeUTR	1	0.15	0.03	1.00	0.00	0.10	0.06	0.19	0.02	20	threeUTR				FALSE
7	141806159	141806159	MGAM (7) threeUTR	1	0.10	0.00	0.99	0.00	0.08	0.03	0.16	0.02	20	threeUTR				FALSE
7	143088841	143088841	EPHA1 (7) nonsynonymous	1	0.27	0.07	1.00	0.00	0.30	0.12	0.07	0.09	10	nonsynonymous	908	S	R	FALSE
8	21988221	21988221	HR (8) fiveUTR	1	0.00	0.00	1.00	0.00	1.00	0.30	1.00	0.23	21	fiveUTR				FALSE
8	48648622	48648622	SPIDR (8) intron	1	0.18	0.00	0.89	0.00	0.11	0.04	0.11	0.03	22	intron				FALSE
8	52321351	52321351	PXDNL (8) nonsynonymous	1	0.00	0.00	0.92	0.00	0.14	0.10	0.08	0.08	10	nonsynonymous	945	A	T	FALSE
8	54963497	54963497	LYPLA1 (8) intron	1	0.14	0.00	0.90	0.00	0.16	0.09	0.10	0.07	22	intron				FALSE
8	67067949	67067949	TRIM55 (8) intron	1	0.15	0.00	0.87	0.00	0.15	0.08	0.13	0.05	22	intron				FALSE
8	68150764	68150764	ARFGEF1 (8) intron	1	0.05	0.00	0.87	0.00	0.09	0.06	0.13	0.05	22	intron				FALSE
8	69028379	69028379	PREX2 (8) intron	1	0.00	0.00	0.91	0.00	0.30	0.24	0.09	0.18	22	intron				FALSE
8	73979532	73979532	SBSPON (8) threeUTR	1	0.11	0.00	0.91	0.00	0.08	0.05	0.03	0.03	20	threeUTR				FALSE
8	93027007	93027007	RUNX1T1 (8) nonsynonymous	1	0.00	0.00	0.92	0.00	0.07	0.07	0.08	0.06	10	nonsynonymous	53	A	T	FALSE
8	100146744	100146744	VPS13B (8) intron	1	0.10	0.00	1.00	0.00	0.17	0.15	0.75	0.11	22	intron				FALSE
8	104433519	104433519	DCAF13 (8) intron	1	0.11	0.00	0.84	0.00	0.12	0.05	0.16	0.05	22	intron				FALSE
8	134250550	134250550	NDRG1 (8) threeUTR	1	0.25	0.03	0.93	0.00	0.13	0.06	0.02	0.02	20	threeUTR				TRUE
8	141678360	141678360	PTK2 (8) intron	1	0.17	0.00	0.89	0.00	0.11	0.05	0.11	0.05	22	intron				FALSE
8	146280342	146280342	C8orf33 (8) threeUTR	1	0.14	0.00	0.79	0.00	0.10	0.03	0.21	0.03	20	threeUTR				FALSE
9	5768725	5768725	KIAA1432 (9) intron	1	0.00	0.00	1.00	0.00	0.19	0.28	0.17	0.17	22	intron				FALSE
9	10341114	10341114	PTPRD (9) intron	1	0.12	0.00	0.86	0.00	0.20	0.12	0.16	0.19	22	intron				FALSE
9	14398884	14398884	NFIB (9) promoter	1	0.05	0.00	0.94	0.00	0.08	0.06	0.07	0.04	19	promoter				TRUE
9	21974707	21974707	CDKN2A (9) nonsynonymous	1	0.17	0.00	1.00	0.00	0.27	0.05	0.24	0.04	10	nonsynonymous	30	EAGALF	A	TRUE
9	33057519	33057519	SMU1 (9) intron	1	0.06	0.00	0.97	0.00	0.10	0.06	0.08	0.09	22	intron				FALSE
9	91619812	91619812	S1PR3 (9) threeUTR	1	0.14	0.00	1.00	0.00	0.09	0.03	0.22	0.02	20	threeUTR				FALSE
9	111778281	111778281	TMEM245 (9) threeUTR	1	0.24	0.00	0.99	0.02	0.09	0.02	0.11	0.03	20	threeUTR				FALSE
9	129740222	129740222	RALGPS1 (9) intron	1	0.00	0.00	1.00	0.00	0.33	0.12	0.86	0.38	22	intron				FALSE
9	131493203	131493203	ZER1 (9) threeUTR	1	0.11	0.00	0.78	0.00	0.13	0.04	0.25	0.03	20	threeUTR				FALSE
9	131834088	131834088	FAM73B (9) threeUTR	1	0.12	0.00	0.93	0.00	0.21	0.07	0.20	0.07	20	threeUTR				FALSE
9	136218018	136218018	SNORD36C (9) intron	1	0.24	0.00	1.00	0.00	0.14	0.05	0.15	0.03	22	intron				FALSE
10	871906	871906	LARP4B (10) intron	1	0.15	0.00	1.00	0.00	0.18	0.07	0.10	0.06	22	intron				FALSE
10	10989390	10989390	LINC00710 (10) intron	1	0.08	0.00	0.99	0.00	0.14	0.08	0.23	0.06	22	intron				FALSE
10	12178237	12178237	SEC61A2 (10) intron	1	0.00	0.00	1.00	0.00	0.10	0.09	0.23	0.05	22	intron				FALSE
10	13152465	13152465	OPTN (10) nonsynonymous	1	0.30	0.00	0.98	0.00	0.29	0.08	0.06	0.05	10	nonsynonymous	120	R	G	FALSE
10	37433716	37433716	ANKRD30A (10) intron	1	0.00	0.00	0.92	0.00	0.18	0.33	0.21	0.17	22	intron				FALSE
10	45799827	45799827	OR13A1 (10) nonsynonymous	1	0.00	0.00	0.93	0.00	0.10	0.07	0.07	0.04	10	nonsynonymous	15	R	H	FALSE
10	55587258	55587258	PCDH15 (10) nonsynonymous	1	0.17	0.00	0.94	0.00	0.12	0.03	0.06	0.03	10	nonsynonymous	1426	A	V	FALSE
10	58118406	58118406	ZWINT (10) nonsynonymous	1	0.17	0.00	0.92	0.02	0.18	0.04	0.05	0.04	10	nonsynonymous	235	L	F	FALSE
10	68858609	68858609	LRRTM3 (10) threeUTR	1	0.15	0.00	0.97	0.00	0.07	0.02	0.03	0.01	20	threeUTR				FALSE
10	74834502	74834502	P4HA1 (10) promoter	1	0.17	0.10	0.91	0.00	0.20	0.18	0.11	0.06	19	promoter				FALSE
10	94214227	94214227	IDE (10) nonsynonymous	1	0.23	0.00	0.96	0.00	0.16	0.04	0.04	0.03	10	nonsynonymous	457	I	V	FALSE
10	105160337	105160337	PDCD11 (10) intron	1	0.10	0.00	1.00	0.00	0.11	0.03	0.05	0.02	22	intron				FALSE
10	122659464	122659464	WDR11 (10) intron	1	0.10	0.00	1.00	0.00	0.17	0.07	0.03	0.06	22	intron				FALSE
10	124377512	124377512	DMBT1 (10) intron	1	0.14	0.14	1.00	0.09	0.23	0.23	0.13	0.16	22	intron				FALSE
10	132890860	132890860	TCERG1L (10) threeUTR	1	0.17	0.00	1.00	0.00	0.18	0.04	0.02	0.03	20	threeUTR				FALSE
11	1251049	1251049	MUC5B (11) intron	1	0.20	0.00	1.00	0.00	0.30	0.08	0.25	0.08	22	intron				FALSE
11	10830818	10830818	EIF4G2 (11) promoter	1	0.00	0.00	1.00	0.00	0.18	0.23	0.47	0.19	19	promoter				FALSE
11	15993677	15993677	SOX6 (11) threeUTR	1	0.10	0.07	1.00	0.02	0.09	0.09	0.07	0.04	20	threeUTR				FALSE
11	34162955	34162955	NAT10 (11) intron	1	0.36	0.00	1.00	0.00	0.46	0.38	1.25	0.28	22	intron				FALSE
11	49197183	49197183	FOLH1 (11) intron	1	0.22	0.00	1.00	0.00	0.32	0.11	0.66	0.17	22	intron				FALSE
11	62483201	62483201	HNRNPUL2-BSCL2 (11) intron	1	0.00	0.00	0.94	0.00	0.17	0.09	0.33	0.06	22	intron				FALSE
11	64570301	64570301	MAP4K2 (11) intron	1	0.00	0.00	1.00	0.00	0.37	0.18	0.09	0.16	22	intron				FALSE
11	64680659	64680659	ATG2A (11) intron	1	0.00	0.00	1.00	0.00	0.18	0.10	0.49	0.16	22	intron				FALSE
11	65789154	65789154	CATSPER1 (11) intron	1	0.18	0.00	1.00	0.00	0.28	0.10	0.08	0.06	22	intron				FALSE
11	68363844	68363844	PPP6R3 (11) intron	1	0.00	0.00	1.00	0.00	0.24	0.14	0.08	0.08	22	intron				FALSE
11	71668279	71668279	RNF121 (11) nonsynonymous	1	0.25	0.00	0.87	0.04	0.19	0.06	0.25	0.06	10	nonsynonymous	24	M	V	FALSE
11	71809007	71809007	LRTOMT (11) intron	1	0.28	0.00	0.87	0.00	0.28	0.07	0.26	0.05	22	intron				FALSE
11	92917845	92917845	SLC36A4 (11) intron	1	0.14	0.00	1.00	0.00	0.22	0.09	0.61	0.13	22	intron				FALSE
11	93457267	93457267	KIAA1731 (11) promoter	1	0.40	0.00	0.99	0.02	0.19	0.03	0.16	0.03	19	promoter				FALSE
11	105851567	105851567	GRIA4 (11) threeUTR	1	0.31	0.00	1.00	0.00	0.16	0.02	0.16	0.02	20	threeUTR				FALSE
11	110040907	110040907	ZC3H12C (11) threeUTR	1	0.23	0.00	0.90	0.00	0.09	0.02	0.20	0.02	20	threeUTR				FALSE
11	112064551	112064551	BCO2 (11) fiveUTR	1	0.12	0.00	1.00	0.00	0.09	0.03	0.21	0.02	21	fiveUTR				FALSE
11	118894949	118894949	TRAPPC4 (11) intergenic	1	0.21	0.00	0.76	0.00	0.15	0.06	0.26	0.04	30	intergenic				FALSE
12	6623913	6623913	NCAPD2 (12) intron	1	0.14	0.00	1.00	0.00	0.12	0.05	0.24	0.03	22	intron				FALSE
12	22610023	22610023	C2CD5 (12) nonsynonymous	1	0.16	0.00	1.00	0.02	0.10	0.03	0.04	0.04	10	nonsynonymous	725	N	S	FALSE
12	32898688	32898688	YARS2 (12) intergenic	1	0.08	0.00	1.00	0.00	0.14	0.07	0.31	0.05	30	intergenic				FALSE
12	42499690	42499690	GXYLT1 (12) nonsynonymous	1	0.13	0.03	0.18	0.02	0.09	0.05	1.00	0.04	10	nonsynonymous	265	Y	C	FALSE
12	42499692	42499692	GXYLT1 (12) nonsense	1	0.14	0.03	0.19	0.02	0.09	0.05	1.00	0.04	4	nonsense	264	Y	*	FALSE
12	42499701	42499701	GXYLT1 (12) nonsynonymous	1	0.16	0.03	0.21	0.02	0.10	0.05	1.00	0.04	10	nonsynonymous	261	R	S	FALSE
12	42499711	42499711	GXYLT1 (12) nonsynonymous	1	0.16	0.03	0.21	0.02	0.10	0.06	1.00	0.04	10	nonsynonymous	258	R	L	FALSE
12	42499714	42499714	GXYLT1 (12) nonsynonymous	1	0.16	0.03	0.24	0.02	0.09	0.06	1.00	0.04	10	nonsynonymous	257	N	S	FALSE
12	42499730	42499730	GXYLT1 (12) synonymous	1	0.16	0.03	0.28	0.04	0.10	0.05	1.00	0.05	16	synonymous	252	R	R	FALSE
12	42499738	42499738	GXYLT1 (12) nonsynonymous	1	0.16	0.03	0.27	0.04	0.10	0.05	1.00	0.05	10	nonsynonymous	249	E	G	FALSE
12	42499763	42499763	GXYLT1 (12) nonsynonymous	1	0.15	0.03	0.29	0.04	0.09	0.05	1.00	0.05	10	nonsynonymous	241	I	V	FALSE
12	42552321	42552321	YAF2 (12) threeUTR	1	0.14	0.00	0.95	0.00	0.09	0.03	0.11	0.02	20	threeUTR				FALSE
12	44152687	44152687	PUS7L (12) promoter	1	0.07	0.00	1.00	0.00	0.12	0.05	0.08	0.05	19	promoter				FALSE
12	70213837	70213837	RAB3IP (12) threeUTR	1	0.12	0.00	1.00	0.00	0.08	0.02	0.06	0.01	20	threeUTR				FALSE
12	77243171	77243171	ZDHHC17 (12) nonsynonymous	1	0.12	0.00	1.00	0.00	0.15	0.05	0.09	0.04	10	nonsynonymous	561	I	V	FALSE
12	81102667	81102667	MYF6 (12) synonymous	1	0.00	0.00	1.00	0.00	0.08	0.03	0.07	0.02	16	synonymous	219	L	L	FALSE
12	99097522	99097522	APAF1 (12) intron	1	0.00	0.00	1.00	0.00	0.33	0.21	0.12	0.15	22	intron				FALSE
12	120149836	120149836	CIT (12) intron	1	0.13	0.00	1.00	0.00	0.21	0.06	0.04	0.05	22	intron				FALSE
12	122277778	122277778	HPD (12) intron	1	0.24	0.00	1.00	0.00	0.18	0.03	0.04	0.03	22	intron				FALSE
13	20660134	20660134	ZMYM2 (13) nonsynonymous	1	0.18	0.00	0.95	0.00	0.11	0.04	0.05	0.03	10	nonsynonymous	1372	L	V	TRUE
13	28840726	28840726	PAN3 (13) intron	1	0.07	0.00	1.00	0.00	0.13	0.18	0.13	0.08	22	intron				FALSE
13	30054448	30054448	MTUS2-AS1 (13) nonsynonymous	1	0.00	0.17	0.96	0.00	0.16	0.28	0.12	0.09	10	nonsynonymous	1126	Q	K	FALSE
13	46288415	46288415	SPERT (13) nonsynonymous	1	0.15	0.00	1.00	0.00	0.26	0.09	0.08	0.07	10	nonsynonymous	419	E	K	FALSE
13	48985815	48985815	RB1 (13) nonsynonymous	1	0.14	0.00	0.96	0.02	0.07	0.03	0.04	0.04	10	nonsynonymous	249	L	F	TRUE
13	99515363	99515363	DOCK9 (13) synonymous	1	0.21	0.00	0.99	0.03	0.15	0.06	0.03	0.06	16	synonymous	1163	T	T	FALSE
13	103493165	103493165	BIVM-ERCC5 (13) threeUTR	1	0.16	0.00	0.97	0.03	0.08	0.03	0.03	0.04	20	threeUTR				FALSE

13	113741987	113741987	MCF2L (13) intron	1	0.00	0.00	1.00	0.00	0.12	0.07	0.05	0.05	22	intron				FALSE
13	113741988	113741988	MCF2L (13) intron	1	0.11	0.00	1.00	0.00	0.19	0.08	0.05	0.05	22	intron				FALSE
14	21464420	21464420	METTL17 (14) frameshift	1	0.14	0.00	0.87	0.00	0.11	0.03	0.17	0.02	5	frameshift	350			FALSE
14	23826434	23826434	EFS (14) threeUTR	1	0.10	0.00	1.00	0.00	0.13	0.06	0.06	0.04	20	threeUTR				FALSE
14	39531020	39531020	SEC23A (14) nonsynonymous	1	0.31	0.00	1.00	0.00	0.23	0.09	0.07	0.05	10	nonsynonymous	485	Y	C	FALSE
14	64619669	64619669	SYNE2 (14) intron	1	0.00	0.00	1.00	0.00	0.17	0.33	0.11	0.21	22	intron				FALSE
14	64886473	64886473	MTHFD1 (14) intron	1	0.13	0.00	0.98	0.00	0.15	0.07	0.29	0.06	22	intron				FALSE
14	65007453	65007453	HSPA2 (14) fiveUTR	1	0.40	0.08	1.00	0.09	0.23	0.10	0.05	0.09	21	fiveUTR				FALSE
14	70078322	70078322	KIAA0247 (14) promoter	1	0.00	0.00	1.00	0.00	0.37	0.38	0.12	0.28	19	promoter				FALSE
14	81557383	81557383	TSHR (14) intron	1	0.18	0.00	1.00	0.00	0.21	0.10	0.37	0.07	22	intron				TRUE
14	90432366	90432366	TDP1 (14) intron	1	0.29	0.00	1.00	0.00	0.24	0.08	0.38	0.06	22	intron				FALSE
14	90455848	90455848	TDP1 (14) intron	1	0.23	0.00	1.00	0.00	0.34	0.25	1.62	0.14	22	intron				FALSE
14	99931898	99931898	SETD3 (14) intron	1	0.00	0.00	1.00	0.00	0.16	0.23	0.12	0.18	22	intron				FALSE
14	104380483	104380483	C14orf2 (14) intron	1	0.11	0.00	0.85	0.00	0.20	0.11	0.36	0.10	22	intron				FALSE
15	31775262	31775262	OTUD7A (15) intergenic	1	0.09	0.00	0.87	0.00	0.11	0.03	0.24	0.03	30	intergenic				FALSE
15	32929026	32929026	ARHGAP11A (15) synonymous	1	0.16	0.00	1.00	0.00	0.09	0.03	0.03	0.04	16	synonymous	495	R	R	FALSE
15	33915962	33915962	RYR3 (15) intron	1	0.08	0.00	1.00	0.00	0.15	0.07	0.02	0.07	22	intron				FALSE
15	37101920	37101920	CSNK1A1P1 (15) threeUTR	1	0.08	0.00	1.00	0.00	0.06	0.02	0.26	0.02	20	threeUTR				FALSE
15	37101921	37101921	CSNK1A1P1 (15) threeUTR	1	0.08	0.00	1.00	0.00	0.06	0.02	0.24	0.02	20	threeUTR				FALSE
15	43771781	43771781	TP53BP1 (15) intron	1	0.17	0.00	0.75	0.00	0.11	0.04	0.23	0.03	22	intron				FALSE
15	65848798	65848798	PTPLAD1 (15) intron	1	0.61	0.00	1.00	0.00	0.63	0.23	0.66	0.46	22	intron				FALSE
15	72957764	72957764	GOLGA6B (15) promoter	1	0.00	0.00	1.00	0.00	0.28	0.10	0.15	0.12	19	promoter				FALSE
15	83488252	83488252	WHAMM (15) intron	1	0.18	0.00	1.00	0.00	0.07	0.02	0.09	0.01	22	intron				FALSE
15	91461431	91461431	MAN2A2 (15) nonsynonymous	1	0.11	0.00	1.00	0.00	0.14	0.04	0.16	0.03	10	nonsynonymous	1001	D	G	FALSE
15	99677758	99677758	TTC23 (15) threeUTR	1	0.07	0.00	0.92	0.00	0.09	0.04	0.13	0.03	20	threeUTR				FALSE
16	3777356	3777356	CREBBP (16) threeUTR	1	0.17	0.00	0.99	0.01	0.08	0.02	0.09	0.02	20	threeUTR				TRUE
16	23684582	23684582	ENST00000566996 (16) threeUTR	1	0.07	0.00	1.00	0.00	0.13	0.06	0.53	0.06	20	threeUTR				FALSE
16	24674276	24674276	ENST00000414816 (16) threeUTR	1	0.21	0.00	0.97	0.00	0.15	0.06	0.20	0.05	20	threeUTR				FALSE
16	29548770	29548770	ENST00000566127 (16) intron	1	0.03	0.00	1.00	0.00	0.02	0.01	0.13	0.01	22	intron				FALSE
16	33750810	33750810	ENST00000567603 (16) intergenic	1	0.04	0.00	1.00	0.00	0.03	0.02	0.21	0.02	30	intergenic				FALSE
16	53524562	53524562	RBL2 (16) threeUTR	1	0.08	0.00	0.98	0.00	0.06	0.02	0.11	0.02	20	threeUTR				FALSE
16	66946358	66946358	CDH16 (16) intron	1	0.00	0.00	0.92	0.00	0.11	0.05	0.18	0.04	22	intron				FALSE
16	67197388	67197388	HSF4 (16) nonsynonymous	1	0.00	0.00	1.00	0.00	0.23	0.21	0.44	0.17	10	nonsynonymous	264	A	S	FALSE
16	73165079	73165079	C16orf47 (16) intergenic	1	0.00	0.00	1.00	0.00	0.13	0.07	0.28	0.10	30	intergenic				FALSE
16	74949925	74949925	WDR59 (16) promoter	1	0.11	0.00	0.98	0.00	0.10	0.03	0.25	0.03	19	promoter				FALSE
16	84209574	84209574	DNAAF1 (16) synonymous	1	0.25	0.00	1.00	0.00	0.18	0.04	0.16	0.03	16	synonymous	578	S	S	FALSE
17	4622735	4622735	ARRB2 (17) intron	1	0.17	0.00	0.96	0.00	0.21	0.08	0.09	0.06	22	intron				FALSE
17	4726688	4726688	PLD2 (17) threeUTR	1	0.11	0.00	0.96	0.00	0.11	0.05	0.06	0.03	20	threeUTR				FALSE
17	7574021	7574021	TP53 (17) nonsense	1	0.46	0.00	1.00	0.00	0.41	0.33	0.27	0.19	4	nonsense	177	E	*	TRUE
17	10302311	10302311	MYH8 (17) intron	1	0.25	0.00	0.93	0.00	0.30	0.28	0.15	0.12	22	intron				FALSE
17	16344510	16344510	FAM211A-AS1 (17) promoter	1	0.20	0.00	0.95	0.00	0.17	0.07	0.08	0.07	19	promoter				FALSE
17	19698847	19698847	ULK2 (17) intron	1	0.22	0.00	0.97	0.00	0.14	0.06	0.07	0.04	22	intron				FALSE
17	27249016	27249016	PHF12 (17) intron	1	0.00	0.00	1.00	0.00	0.25	0.12	0.11	0.10	22	intron				FALSE
17	34500162	34500162	TBC1D3B (17) promoter	1	0.00	0.00	1.00	0.00	1.00	0.38	0.18	0.46	19	promoter				FALSE
17	39211182	39211182	KRTAP2-2 (17) nonsense	1	0.18	0.00	0.96	0.00	0.30	0.28	0.20	0.11	4	nonsense	94	W	*	FALSE
17	39432007	39432007	KRTAP9-7 (17) nonsynonymous	1	0.12	0.00	0.75	0.08	0.15	0.07	0.34	0.10	10	nonsynonymous	20	K	Q	FALSE
17	40453591	40453591	STAT5A (17) intron	1	0.00	0.00	0.91	0.00	0.21	0.13	0.12	0.11	22	intron				FALSE
17	40619911	40619911	ATP6V0A1 (17) intron	1	0.15	0.00	1.00	0.00	0.18	0.13	0.07	0.10	22	intron				FALSE
17	42949718	42949718	EFTUD2 (17) intron	1	0.10	0.00	0.95	0.00	0.17	0.10	0.10	0.11	22	intron				FALSE
17	47301655	47301655	PHOSPHO1 (17) nonsynonymous	1	0.08	0.00	0.98	0.00	0.15	0.08	0.07	0.05	10	nonsynonymous	278	A	T	FALSE
17	49043275	49043275	SPAG9 (17) threeUTR	1	0.13	0.00	1.00	0.01	0.06	0.02	0.04	0.02	20	threeUTR				FALSE
17	53040488	53040488	COX11 (17) threeUTR	1	0.05	0.00	0.98	0.00	0.09	0.06	0.07	0.04	20	threeUTR				FALSE
17	57094454	57094454	TRIM37 (17) intron	1	0.09	0.10	1.00	0.00	0.17	0.18	0.09	0.11	22	intron				FALSE
17	65822516	65822516	BPTF (17) intron	1	0.00	0.00	0.93	0.00	0.17	0.12	0.19	0.17	22	intron				FALSE
17	79612283	79612283	TSPAN10 (17) nonsynonymous	1	0.00	0.00	1.00	0.00	0.15	0.07	0.23	0.07	10	nonsynonymous	139	G	D	FALSE
18	263889	263889	THOC1 (18) intron	1	0.17	0.06	0.85	0.00	0.17	0.10	0.33	0.06	22	intron				FALSE
18	5289970	5289970	ZBTB14 (18) threeUTR	1	0.05	0.00	0.98	0.00	0.06	0.02	0.22	0.02	20	threeUTR				FALSE
18	10797387	10797387	PIEZO2 (18) synonymous	1	0.12	0.00	0.82	0.00	0.09	0.03	0.19	0.02	16	synonymous	504	A	A	FALSE
18	45361031	45361031	SMAD2 (18) threeUTR	1	0.04	0.00	0.95	0.00	0.05	0.03	0.14	0.02	20	threeUTR				FALSE
18	47432937	47432937	MYO5B (18) nonsynonymous	1	0.04	0.00	0.90	0.00	0.07	0.05	0.18	0.04	10	nonsynonymous	756	L	V	FALSE
18	61018283	61018283	KDSR (18) synonymous	1	0.19	0.00	0.83	0.00	0.16	0.07	0.20	0.05	16	synonymous	149	S	S	TRUE
19	920210	920210	KISS1R (19) intron	1	0.31	0.00	0.77	0.00	0.48	0.28	0.45	0.28	22	intron				FALSE
19	6379859	6379859	GTF2F1 (19) threeUTR	1	0.13	0.00	1.00	0.00	0.15	0.06	0.07	0.06	20	threeUTR				FALSE
19	8954182	8954182	MBD3L1 (19) intergenic	1	0.14	0.00	1.00	0.00	0.23	0.16	0.69	0.13	30	intergenic				FALSE
19	10218552	10218552	SNORD105 (19) promoter	1	0.00	0.00	1.00	0.00	0.19	0.17	0.08	0.09	19	promoter				FALSE
19	12126096	12126096	ZNF433 (19) nonsynonymous	1	0.16	0.00	0.98	0.00	0.09	0.02	0.15	0.01	10	nonsynonymous	529	T	I	FALSE
19	16952721	16952721	SIN3B (19) nonsynonymous	1	0.13	0.00	1.00	0.02	0.11	0.03	0.24	0.04	10	nonsynonymous	175	R	L	FALSE
19	21350593	21350593	ZNF431 (19) intron	1	0.00	0.00	1.00	0.00	0.08	0.19	0.09	0.10	22	intron				FALSE
19	21838431	21838431	? (19) intergenic	1	0.08	0.00	1.00	0.00	0.08	0.04	0.05	0.03	30	intergenic				FALSE
19	22000875	22000875	ZNF43 (19) intron	1	0.00	0.00	1.00	0.00	0.04	0.07	0.06	0.04	22	intron				FALSE
19	49838976	49838976	CD37 (19) synonymous	1	0.12	0.00	0.86	0.00	0.15	0.06	0.22	0.05	16	synonymous	25	L	L	FALSE
19	51128285	51128285	SYT3 (19) intron	1	0.00	0.00	1.00	0.00	0.33	0.28	0.39	0.23	22	intron				FALSE
19	51129043	51129043	SYT3 (19) intron	1	0.00	0.00	1.00	0.00	0.38	0.46	0.46	0.19	22	intron				FALSE
19	58511505	58511505	ZNF606 (19) intron	1	0.00	0.00	1.00	0.00	0.33	0.25	0.50	0.38	22	intron				FALSE
20	209880	209880	DEFB129 (20) intron	1	0.15	0.00	0.82	0.00	0.11	0.03	0.15	0.02	22	intron				FALSE
20	16721937	16721937	SNRPB2 (20) threeUTR	1	0.19	0.00	1.00	0.01	0.09	0.02	0.16	0.03	20	threeUTR				FALSE
20	23666306	23666306	CST4 (20) threeUTR	1	0.00	0.00	1.00	0.00	0.10	0.05	0.27	0.04	20	threeUTR				FALSE
20	40710266	40710266	PTPRT (20) intron	1	0.00	0.00	1.00	0.18	1.00	0.21	1.74	0.30	22	intron				FALSE
20	47865426	47865426	ZNFX1 (20) nonsynonymous	1	0.05	0.00	1.00	0.00	0.08	0.03	0.29	0.03	10	nonsynonymous	1379	S	P	FALSE
20	49197907	49197907	PTPN1 (20) synonymous	1	0.15	0.00	0.90	0.00	0.18	0.07	0.12	0.05	16	synonymous	398	D	D	FALSE
20	54573555	54573555	CBLN4 (20) threeUTR	1	0.07	0.00	0.98	0.00	0.07	0.03	0.14	0.02	20	threeUTR				FALSE
20	58449379	58449379	SYCP2 (20) intron	1	0.00	0.00	1.00	0.00	0.30	0.33	0.56	0.46	22	intron				FALSE
21	15981613	15981613	ENST00000435732 (21) intron	1	0.27	0.00	1.00	0.00	0.38	0.25	0.13	0.19	22	intron				FALSE
21	31015564	31015564	GRIK1 (21) intron	1	0.20	0.00	1.00	0.00	0.30	0.10	0.11	0.12	22	intron				FALSE
21	43307671	43307671	C2CD2 (21) threeUTR	1	0.17	0.00	1.00	0.02	0.12	0.03	0.05	0.03	20	threeUTR				FALSE
21	46888290	46888290	COL18A1 (21) nonsynonymous	1	0.18	0.00	0.97	0.00	0.21	0.06	0.06	0.04	10	nonsynonymous	81	R	W	FALSE
21	47860819	47860819	PCNT (21) nonsynonymous	1	0.07	0.00	0.98	0.00	0.11	0.05	0.05	0.03	10	nonsynonymous	3149	L	V	FALSE
22	23213668	23213668	IGLV4-3 (22) intron	1	0.28	0.06	1.00	0.00	0.19	0.11	0.06	0.03	22	intron				FALSE
22	28194390	28194390	MN1 (22) synonymous	1	0.00	0.00	1.00	0.00	0.47	0.23	1.20	0.25	16	synonymous	714	S	S	TRUE
22	29182912	29182912	CCDC117 (22) threeUTR	1	0.20	0.00	0.90	0.00	0.11	0.04	0.16	0.02	20	threeUTR				FALSE
22	30892255	30892255	SEC14L4 (22) intron	1	0.00	0.00	0.95	0.00	1.00	1.00	0.86	0.28	22	intron				FALSE
22	42481512	42481512	NDUFA6 (22) intergenic	1	0.18	0.00	1.00	0.00	0.21	0.11	0.34	0.05	30	intergenic				FALSE
22	46640929	46640929	CDPF1 (22) threeUTR	1	0.17	0.00	0.84	0.00	0.20	0.08	0.24	0.03	20	threeUTR				FALSE
22	50943916	50943916	LMF2 (22) nonsynonymous	1	0.23	0.00	0.87	0.00	0.34	0.15	0.30	0.09	10	nonsynonymous	298	V	I	FALSE
X	13795309	13795309	GPM6B (X) intron	1	0.00	0.00	0.96	0.00	1.00	1.00	0.11	1.00	22	intron				FALSE
X	69725361	69725361	DLG3 (X) intergenic	1	0.15	0.00	1.00	0.00	0.85	1.00	10.00	1.00	30	intergenic				FALSE
X	79926082	79926082	BRWD3 (X) threeUTR	1	0.04	0.05	0.92	0.00	0.96	0.95	0.35	1.00	20	threeUTR				FALSE
Y	7246289	7246289	RNU6-941P (Y)	1	0.00	0.00	0.94	0.00	1.00	1.00	0.10	1.00	100					FALSE
1	50513686	121309999	71Mbp AA (1)	1	0.00	0.07	0.94	0.06	0.06	0.12	0.02	0.12						
1	158368312	249213345	91Mbp AAB (1)	1	0.14	0.01	0.00	0.00	0.10	0.10	1.57	0.08						
1	158368312	249213345	91Mbp AABB (1)	1	0.07	0.00	0.87	0.00	0.02	0.10	0.03	0.06						
2	38814	196602851	197Mbp AABB (2)	1	0.14	0.01	0.91	0.00	0.09	0.06	0.05	0.10						
2	217122585	243102469	26Mbp AAB (2)	1	0.16	0.00	0.91	0.00	0.10	0.08	0.10	0.08						
3	237441	89531284	89Mbp AA (3)	1	0.14	0.00	0.95	0.00	0.04	0.07	0.02	0.06						
3	93591881	156878482	63Mbp 4ABB (3)	1	0.14	0.06	0.88	0.02	0.09	0.10	0.10	0.10						
4	53227	187644987	188Mbp AA (4)	1	0.10	0.07	0.95	0.00	0.06	0.09	0.04	0.11						
5	140373	45696220	46Mbp AAB (5)	1	0.16	0.02	0.89	0.00	0.10	0.05	0.10	0.07						
5	49692031	59787092	10Mbp AAB (5)	1	0.19	0.04	0.00	0.02	0.11	0.10	8.61	0.10						
5	49692031	59787092	10Mbp AA (5)	1	0.09	0.06	0.94	0.01	0.08	0.06	0.05	0.09						
5	61601989	98860018	37Mbp AAB (5)	1	0.16	0.04	0.00	0.01	0.10	0.03	22.22	0.03						
5	61601989	98860018	37Mbp AA (5)	1	0.07	0.07	0.94	0.00	0.10	0.07	0.03	0.08						
5	99871124	180795226	81Mbp AAB (5)	1	0.13	0.02	0.90	0.00	0.10	0.10	0.11	0.04						
7	149597	100611619	100Mbp AABB (7)	1	0.13	0.00	0.90	0.00	0.03	0.01	0.03	0.01						
7	100701284	158937649	58Mbp AABB (7)	1	0.14	0.00	0.93	0.00	0.02	0.10	0.03	0.10						
9	14511	32573182	33Mbp AA (9)	1	0.07	0.07	0.95	0.18	0.12	0.08	0.02	0.10						

9	65487273	84303596	19Mbp AAB (9)	1	0.09	0.04	0.94	0.04	0.07	0.09	0.10	0.43						
9	85597315	141134172	56Mbp AAB (9)	1	0.00	0.00	0.97	0.03	0.10	0.08	0.11	0.54						
10	92828	46411621	46Mbp AAB (10)	1	0.17	0.00	0.00	0.00	0.10	0.10	20.56	0.10						
10	92828	46411621	46Mbp AA (10)	1	0.06	0.06	0.93	0.04	0.10	0.11	0.04	0.11						
10	47658234	99790585	52Mbp AAB (10)	1	0.19	0.01	0.00	0.02	0.10	0.03	21.73	0.10						
10	47658234	99790585	52Mbp AA (10)	1	0.07	0.00	0.93	0.06	0.12	0.07	0.09	0.08						
10	99894381	135233096	35Mbp BBA (10)	1	0.18	0.00	0.00	0.00	0.07	0.10	27.07	0.10						
11	123328	50379802	50Mbp AAB (11)	1	0.16	0.00	0.93	0.00	0.04	0.04	0.10	0.06						
11	51411378	79151695	28Mbp AAB (11)	1	0.12	0.00	0.00	0.00	0.10	0.04	1.50	0.03						
11	81601783	92715948	11Mbp AABB (11)	1	0.17	0.01	0.92	0.01	0.05	0.10	0.03	0.09						
11	92880850	110583451	18Mbp AAABB (11)	1	0.00	0.00	0.91	0.00	0.10	0.09	0.10	0.05						
12	87984	34181237	34Mbp AAB (12)	1	0.19	0.00	0.00	0.00	0.10	0.08	1.72	0.07						
12	87984	34181237	34Mbp AABB (12)	1	0.09	0.00	0.93	0.00	0.02	0.06	0.07	0.05						
12	37935858	41968392	4Mbp AAB (12)	1	0.26	0.00	0.99	0.00	0.10	0.10	0.11	0.09						
12	42550906	46384401	4Mbp AAB (12)	1	0.26	0.02	1.00	0.00	0.09	0.10	0.05	0.04						
13	19408543	103719196	84Mbp AA (13)	1	0.06	0.08	0.95	0.06	0.08	0.08	0.07	0.08						
13	106118216	106143383	25kbp CL (13)	1	0.00	0.04	0.93	0.06	0.07	0.06	0.08	0.07						
14	29236287	97347951	68Mbp AABB (14)	1	0.09	0.00	0.93	0.01	0.02	0.07	0.06	0.10						
15	34159696	45983479	12Mbp AAABB (15)	1	0.05	0.00	0.90	0.00	0.01	0.03	0.10	0.01						
15	48010686	59815751	12Mbp AAB (15)	1	0.18	0.02	0.99	0.02	0.10	0.08	0.10	0.06						
15	59903982	60690185	786kbp 4AB (15)	1	0.08	0.00	0.91	0.01	0.02	0.02	0.03	0.05						
15	60711808	102519296	42Mbp AAB (15)	1	0.07	0.00	0.97	0.00	0.10	0.06	0.10	0.05						
16	61555	5147789	5Mbp A (16)	1	0.13	0.05	0.00	0.05	0.10	0.10	1.34	0.05						
16	61555	5147789	5Mbp AAB (16)	1	0.00	0.00	0.89	0.00	0.12	0.03	0.10	0.03						
16	6069132	28223190	22Mbp AAB (16)	1	0.00	0.02	0.96	0.01	0.10	0.06	0.04	0.07						
16	46503249	70099851	24Mbp AAB (16)	1	0.01	0.00	0.95	0.01	0.04	0.04	0.11	0.03						
16	70253484	90242304	20Mbp AAB (16)	1	0.00	0.00	0.93	0.00	0.06	0.07	0.06	0.03						
17	260118	16557167	16Mbp AAAA (17)	1	0.02	0.00	0.90	0.00	0.08	0.10	0.09	0.13						
17	16828644	18328647	2Mbp AAAA (17)	1	0.04	0.00	0.91	0.01	0.03	0.05	0.06	0.03						
17	18486722	21156578	3Mbp AAAA (17)	1	0.08	0.00	0.94	0.00	0.09	0.08	0.10	0.05						
17	21431571	61021673	40Mbp AAAA (17)	1	0.05	0.00	0.92	0.01	0.10	0.09	0.03	0.08						
18	109065	19782227	20Mbp AAB (18)	1	0.10	0.00	1.00	0.00	0.11	0.10	0.09	0.05						
19	28281401	50316567	22Mbp AA (19)	1	0.08	0.07	0.93	0.07	0.08	0.08	0.05	0.07						
20	68351	33413433	33Mbp AABB (20)	1	0.18	0.05	0.91	0.01	0.05	0.10	0.05	0.02						
20	33421378	50808524	17Mbp AAB (20)	1	0.24	0.12	0.97	0.03	0.10	0.10	0.06	0.07						
21	9907194	48084863	38Mbp AAB (21)	1	0.14	0.01	0.00	0.07	0.10	0.09	23.13	0.10						
21	9907194	48084863	38Mbp AA (21)	1	0.07	0.06	0.94	0.07	0.09	0.11	0.04	0.07						

chr	start	end	name	clone	clonality.HK2-Blood	clonality.HK2-EA	clonality.HK2-HA	clonality.HK2-HB	clonality.HK2-HC	error.HK2-Blood	error.HK2-EA	error.HK2-HA	error.HK2-HB	error.HK2-HC	severity	type	AApos	AAbefore	AAafter	isCosmicCensus
1	39927463	39927463	MACF1 (1) promoter	1	0.00	0.71	0.64	0.76	0.87	0.11	0.15	0.61	0.35	0.77	19	promoter				FALSE
1	45140619	45140619	TMEM53 (1) promoter	1	0.11	0.98	0.78	0.61	0.86	0.19	0.07	0.32	0.34	0.34	19	promoter				FALSE
1	228538968	228538968	OBSCN (1) promoter	1	0.14	0.74	0.52	0.70	0.78	0.23	0.27	0.45	0.70	0.37	19	promoter				FALSE
6	18163377	18163377	KDM1B (6) intron	1	0.00	0.63	0.90	0.46	0.61	0.21	0.18	0.68	0.54	1.02	22	intron				FALSE
8	6420073	6420073	MCPH1 (8) intron	1	0.00	0.98	0.82	1.00	0.77	0.21	0.36	0.33	1.75	0.35	22	intron				FALSE
9	95039956	95039956	IARS (9) intron	1	0.00	0.52	0.79	1.00	0.31	0.18	0.20	0.54	2.55	0.69	22	intron				FALSE
10	29784297	29784297	SVIL (10) intron	1	0.00	0.62	0.87	0.82	0.84	0.23	0.37	1.69	0.49	0.38	22	intron				FALSE
10	71851429	71851429	H2AFY2 (10) intron	1	0.00	0.77	0.71	0.88	0.84	0.19	0.32	0.34	0.37	0.63	22	intron				FALSE
10	105218360	105218360	CALHM1 (10) nonsynonymous	1	0.00	1.00	0.92	0.70	0.60	0.17	0.28	0.39	0.70	0.40	10	nsynonymo	50	A	V	FALSE
11	73850973	73850973	C2CD3 (11) intron	1	0.00	0.69	1.00	0.82	1.00	0.19	0.18	0.19	0.71	0.18	22	intron				FALSE
15	30385056	30385056	GOLGA8J (15) promoter	1	0.13	0.65	0.89	0.84	0.73	0.16	0.11	0.38	0.44	0.36	19	promoter				FALSE
15	78486551	78486551	ACSBG1 (15) intron	1	0.00	1.00	0.81	1.00	1.00	0.23	6.18	0.39	0.66	0.70	22	intron				FALSE
19	18420704	18420704	LSM4 (19) intron	1	0.00	1.00	0.85	1.00	0.62	0.19	0.30	0.68	0.15	1.09	22	intron				FALSE
19	49642746	49642746	PPFIA3 (19) intron	1	0.00	1.00	0.71	0.95	0.71	0.19	0.37	0.56	0.38	0.56	22	intron				FALSE
19	51957556	51957556	SIGLEC8 (19) nonsynonymous	1	0.12	1.00	1.00	0.95	0.95	0.21	0.18	0.25	0.10	0.26	10	nsynonymo	279	R	G	FALSE
20	44243376	44243376	WFDC9 (20) intron	1	0.00	0.77	1.00	1.00	0.87	0.14	0.27	0.21	2.42	0.98	22	intron				FALSE
20	51869840	51869840	TSHZ2 (20) intron	1	0.00	0.46	0.62	0.72	1.00	0.19	0.31	0.67	0.39	0.44	22	intron				FALSE
21	42830399	42830399	MX1 (21) intron	1	0.00	0.65	0.55	0.75	0.40	0.13	0.29	0.45	0.96	0.60	22	intron				FALSE
21	46929467	46929467	SLC19A1 (21) synonymous	1	0.00	1.00	1.00	1.00	1.00	0.19	0.36	0.18	0.13	0.12	16	synonymous	1149	A	A	FALSE
22	22979748	22979748	? (22) promoter	1	0.00	0.71	0.98	0.37	0.46	0.15	0.29	0.30	0.38	0.34	19	promoter				FALSE
3	237441	3221401	3Mbp AAB (3)	1	0.04	0.00	0.00	0.82	0.00	0.10	2.11	0.84	0.11	0.74						
5	723709	3601517	3Mbp AAB (5)	1	0.00	1.00	0.97	1.00	0.74	0.11	0.07	0.11	0.97	0.11						
5	16451628	23528706	7Mbp AAB (5)	1	0.01	1.00	0.00	0.76	0.00	0.11	0.17	0.92	0.05	0.97						
5	24487209	28927420	4Mbp AAAB (5)	1	0.01	0.66	0.92	1.00	0.70	0.07	0.04	0.08	0.07	0.42						
6	16129317	34250571	18Mbp AAB (6)	1	0.00	0.00	0.09	0.81	0.00	0.07	2.75	1.10	0.11	1.04						
9	65487273	96717608	31Mbp AAAB (9)	1	0.01	0.00	0.34	0.78	0.38	0.17	2.11	0.67	0.10	0.62						
9	97861336	103115863	5Mbp AAAB (9)	1	0.01	0.00	0.35	0.79	0.36	0.20	1.98	0.61	0.10	0.64						
11	69624736	69634192	9kbp 13AB (11)	1	0.00	0.39	0.84	1.00	0.96	0.08	0.10	0.11	0.08	0.12						
11	69831789	70053508	222kbp 12ABB (11)	1	0.00	0.46	0.80	0.98	0.86	0.02	0.18	0.04	0.05	0.03						
15	20083807	48635570	29Mbp AAB (15)	1	0.00	0.00	0.04	0.86	0.03	0.22	2.65	0.91	0.05	1.00						
15	48700503	48944213	244kbp AAB (15)	1	0.05	0.00	0.12	0.86	0.04	0.11	3.34	0.81	0.10	1.01						
15	49030135	102519296	53Mbp AAB (15)	1	0.00	0.00	0.07	0.86	0.06	0.08	2.54	0.99	0.04	1.04						
21	47518033	47717665	200kbp AAB (21)	1	0.05	0.62	0.36	1.00	0.44	0.11	0.11	0.84	0.07	0.93						

chr	start	end	name	clone	clonality.HK3_Blood	clonality.HK3-EB	clonality.HK3-HA	error.HK3_Blood	error.HK3-EB	error.HK3-HA	severity	type	AApos	AAbefore	AAafter	isCosmicCensus
2	85510388	85510388	TCF7L1 (2) intron	1	0.00	0.81	0.57	0.18	0.80	0.51	22	intron				FALSE
2	211471792	211471792	CPS1 (2) intron	1	0.00	0.76	0.91	0.19	0.65	0.25	22	intron				FALSE
3	42907411	42907411	KRBOX1 (3) threeUTR	1	0.18	0.89	0.72	0.18	0.20	0.56	20	threeUTR				FALSE
6	122792608	122792608	SERINC1 (6) promoter	1	0.00	0.90	0.79	0.18	0.64	0.58	19	promoter				FALSE
6	124125152	124125152	NKAIN2 (6) promoter	1	0.00	0.51	1.00	0.18	0.49	0.44	19	promoter				FALSE
6	152469331	152469331	SYNE1 (6) synonymous	1	0.14	1.00	1.00	0.25	1.30	0.34	16	synonymous	477	P	P	FALSE
7	44798697	44798697	ZMIZ2 (7) promoter	1	0.00	0.96	0.79	0.19	0.49	0.87	19	promoter				FALSE
9	141070630	141070630	TUBBP5 (9) intron	1	0.08	0.85	1.00	0.15	0.16	0.31	22	intron				FALSE
12	104519627	104519627	NFYB (12) intron	1	0.00	1.00	0.60	0.23	0.47	0.40	22	intron				FALSE
12	124835352	124835352	NCOR2 (12) intron	1	0.00	0.84	0.77	0.19	0.39	0.23	22	intron				FALSE
16	1138795	1138795	? (16) threeUTR	1	0.08	1.00	1.00	0.14	0.72	0.22	20	threeUTR				FALSE
16	1138797	1138797	? (16) threeUTR	1	0.16	1.00	1.00	0.19	0.90	0.23	20	threeUTR				FALSE
16	2446021	2446021	ABCA17P (16) intron	1	0.00	1.00	0.77	0.21	0.12	0.23	22	intron				FALSE
16	11348211	11348211	SOCS1 (16) intron	1	0.00	1.00	1.00	0.21	0.12	0.09	22	intron				TRUE
16	22539282	22539282	NPIPB5 (16) nonsynonymous	1	0.14	0.94	0.62	0.25	0.17	0.21	10	nonsynonymous	178	T	S	FALSE
16	31733698	31733698	ZNF720 (16) intron	1	0.00	1.00	0.65	0.23	0.08	0.58	22	intron				FALSE
16	55798778	55798778	CES1P1 (16) fiveUTR	1	0.00	0.84	0.78	0.11	0.22	0.35	21	fiveUTR				FALSE
16	55798783	55798783	CES1P1 (16) fiveUTR	1	0.00	0.77	0.84	0.12	0.30	0.34	21	fiveUTR				FALSE
17	40760875	40760875	TUBG1 (17) promoter	1	0.00	0.91	0.86	0.23	0.15	0.38	19	promoter				FALSE
17	47132519	47132519	IGF2BP1 (17) threeUTR	1	0.06	0.06	0.85	0.08	1.00	0.08	20	threeUTR				FALSE
18	29784585	29784585	MEP1B (18) intron	1	0.00	0.93	1.00	0.21	0.19	0.39	22	intron				FALSE
19	3009770	3009770	TLE2 (19) intron	1	0.00	1.00	1.00	0.23	125.01	1.37	22	intron				FALSE
22	16158087	16158087	ENST00000447898 (22) intron	1	0.00	1.00	0.74	0.21	0.39	0.34	22	intron				FALSE
22	17265124	17265124	XKR3 (22) nonsynonymous	1	0.08	1.00	0.90	0.11	0.39	0.33	10	nonsynonymous	255	F	L	FALSE
22	20457383	20457383	RIMBP3 (22) nonsynonymous	1	0.06	0.86	0.50	0.07	0.32	0.50	10	nonsynonymous	1307	P	T	FALSE
22	37964417	37964417	CDC42EP1 (22) nonsynonymous	1	0.15	1.00	0.90	0.18	0.31	0.25	10	nonsynonymous	256	A	T	TRUE
1	11006530	12269277	1Mbp A (1)	1	0.12	0.71	0.00	0.10	0.52	2.81						
1	12290113	12838049	548kbp A (1)	1	0.09	0.69	0.00	0.07	0.56	2.26						
1	43613594	48714316	5Mbp AAAB (1)	1	0.00	0.00	0.91	0.04	6.71	0.08						
3	51812577	52248343	436kbp AA (3)	1	0.13	0.87	0.80	0.18	0.22	0.21						
9	140201348	141134172	933kbp A (9)	1	0.15	0.82	0.18	0.11	0.07	1.46						
14	54416454	57397550	3Mbp AAB (14)	1	0.00	0.83	0.00	0.11	0.08	3.93						
16	11348274	12668146	1Mbp A (16)	1	0.09	0.92	0.00	0.11	0.04	2.33						
17	2496923	3337135	840kbp A (17)	1	0.03	0.87	0.66	0.05	0.09	4.24						
17	21431571	22023991	592kbp A (17)	1	0.04	0.96	0.79	0.12	0.09	0.10						
